# Site-selective decarbonylative [4 + 2] annulation of carboxylic acids with terminal alkynes by C–C/C–H activation strategy and cluster catalysis†

**DOI:** 10.1039/d4sc05429f

**Published:** 2024-11-14

**Authors:** Mengjie Cen, Xinyue Ma, Xi Yang, Shangshang Zhang, Long Liu, Michal Szostak, Tieqiao Chen

**Affiliations:** a School of Chemistry and Chemical Engineering, Hainan University Haikou 570228 China chentieqiao@hnu.edu.cn; b Hainan Research Academy of Environmental Sciences Haikou 571127 PR China; c Department of Chemistry, Rutgers University 73 Warren Street Newark NJ 07102 USA michal.szostak@rutgers.edu

## Abstract

Cycloaddition and annulation strategies are among the most powerful methods for creating molecular complexity in organic molecules. In this manuscript, we report a highly site-selective palladium-catalyzed decarbonylative [4 + 2] cyclization of carboxylic acids with terminal alkynes by a sequential C–C/C–H bond activation. Most notably, this method represents the first use of carboxylic acids as the ubiquitous and underdeveloped synthons for intramolecular cycloadditions by decarbonylative C–C bond cleavage. The method provides a solution to the long-standing challenge of the regioselective synthesis of substituted naphthalenes by cycloaddition. Mechanistic studies show that this reaction occurs through a sequential process involving the formation of key palladacycle by a sequential C–C/C–H bond activation and highly regioselective alkyne insertion enabled by cluster catalysis. Wide substrate scope for both carboxylic acids and terminal alkynes is demonstrated with high functional group tolerance. Moreover, this reaction is scalable and applicable to the synthesis of functionalized molecules featuring bioactive fragments. This reaction advances the toolbox of redox-neutral carboxylic acid interconversion to cycloaddition processes. We anticipate that this approach will find broad application in organic synthesis, drug discovery and functionalized material research fields.

## Abstract 2

A highly site-selective decarbonylative [4 + 2] cyclization of carboxylic acids with terminal alkynes forming naphthalenes is enabled by palladium cluster catalysis.

## Graphical abstract

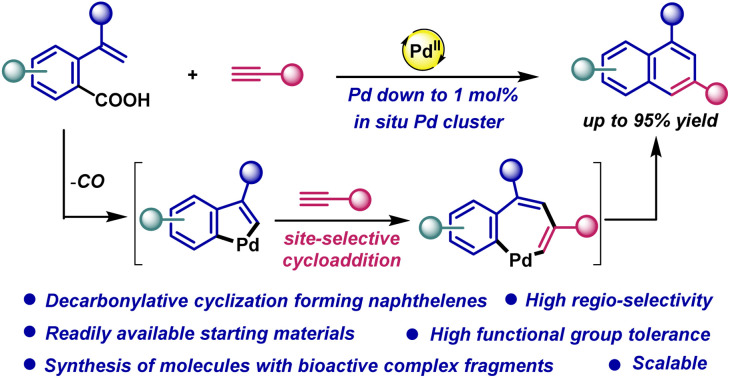

## Introduction

Cycloaddition and annulation strategies are among the most powerful methods in organic synthesis and an area of intense interest from academic and industrial perspectives.^1,2^ In particular, [4 + 2] cycloadditions, such as the venerable Diels–Alder reaction, are of major importance in organic synthesis, drug discovery and functional material science. Catalytic cycloadditions involving regioselective activation of inert bonds are of particular value since they provide nonclassical methods for the creation of molecular complexity by cycloaddition processes. In this context, the naphthalene ring represents a prevalent structural motif found in a plethora of drugs, natural products and advanced materials. Naphthalenes show unique optoelectronic properties and thus are widely used as optical and electronic materials.^3^ Over the years, the efficient construction of rings has received significant attention.^4–7^ Although straightforward and atom-economic methods for the synthesis of naphthalenes have been established, at present, there are major challenges in deploying this method for the site-selective synthesis of the naphthalene ring. In general, acid-catalyzed electrophilic cyclizations are limited by the site-selectivity at the original aryl ring and restricted to electron-rich arenes.^5^ In contrast, the transition-metal-catalyzed approach is one of the most straightforward methods for the construction of carbocycles; however, in the case of naphthalene rings, this approach is limited to symmetrical coupling partners to avoid regio-selectivity issues at the newly formed benzene ring and only limited functional groups can be introduced by this process.^6^ Furthermore, the method since intramolecular cyclization approach does not represent an efficient the preparation of functionalized substrates is a major limitation.^7^ Thus, despite their significant utility, general and modular methods for the synthesis of polyfunctionalized naphthalene rings with high site-specificity of the introduction of various functional groups from the readily available starting materials are underdeveloped.

Carboxylic acids are among the most ubiquitous, naturally abundant and commercially available substrates in organic synthesis.^8^ Recent years have witnessed an explosion of interest in the utilization of the carboxylic acid functional group by metal catalysis to facilitate the synthesis of functionalized molecules.^9–12^ Thus far, carboxylic acids have been widely used as the acyl source in organic synthesis after the selective oxidative addition of the acyl bond to transition metals.^10^ Significant advances have also been made in the cross-coupling of carboxylic acids by orthogonal activation pathways by decarboxylation^11^ and decarbonylation.^12^ However, the use of carboxylic acids as modular synthons for intermolecular site-selective cycloadditions to rapidly build-up molecular complexity is underdeveloped, despite their untapped utility as broadly available precursors for this class of transformations.

**Scheme 1:**
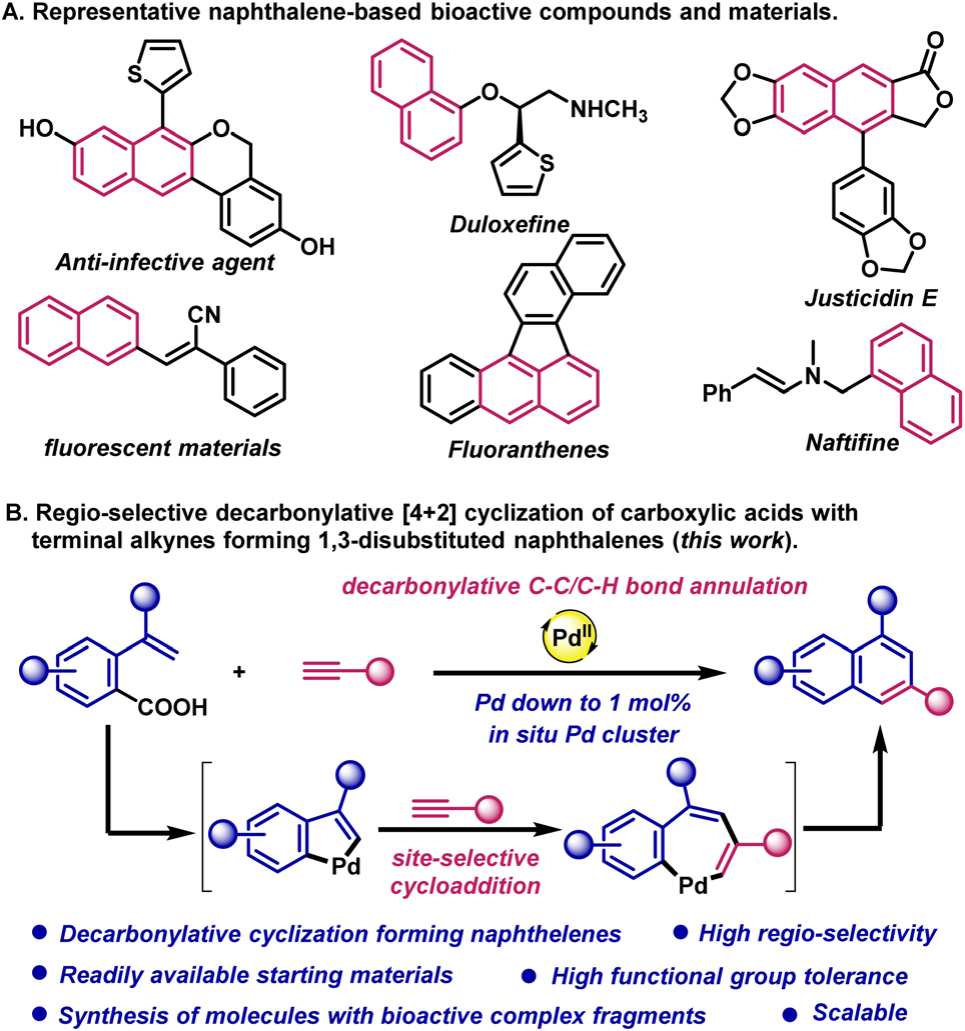
(A) Representative naphthalene-based bioactive natural products and materials. (B) Decarbonylative site-selective C–C/C–H cycloaddition of carboxylic acids forming functionalized naphthalenes.

Herein, we report a highly site-selective palladium-catalyzed decarbonylative [4 + 2] cyclization of carboxylic acids with terminal alkynes by a sequential C–C/C–H bond activation. Most notably, this method represents the first use of carboxylic acids as the ubiquitous and underdeveloped synthons for intramolecular cycloadditions by decarbonylative C–C bond cleavage. The method provides a solution to the long-standing challenge of the regioselective synthesis of substituted naphthalenes by cycloaddition. Mechanistic studies show that this reaction occurs through a sequential process involving the formation of key palladacycle by a sequential C–C/C–H bond activation and highly regioselective alkyne insertion enabled by cluster catalysis. Wide substrate scope for both carboxylic acids and terminal alkynes is demonstrated with high functional group tolerance (>60 examples) (Scheme 1).

Valuable functional group, such as ethers, thioethers, halides, fluorinated functional groups, esters, ketones, aldehydes, nitriles, various heterocycles, silicon protecting groups, alkenes and even organoboranes are well tolerated under the reaction conditions. Moreover, this reaction is scalable and applicable to the smooth synthesis of functionalized molecules featuring bioactive fragments. This reaction advances the toolbox of redox-neutral carboxylic acid interconversion to cycloaddition processes and enables the site-selective synthesis of functionalized naphthalenes from readily available carboxylic acids. We anticipate that this approach will find broad application in organic synthesis, drug discovery and functionalized material research fields.

## Results and discussion

### Reaction optimization

We initiated our investigation by examining the cycloaddition of 2-(1-phenylvinyl)benzoic acid 1a and phenylacetylene 2a as a model system as summarized in Table 1. We are delighted to find that reacting the mixture of 1a (0.2 mmol), 2a (0.4 mmol), PdCl_2_ (5 mol%), TFP (10 mol%), Piv_2_O (1.4 equiv.), DMAP (4-dimethylaminopyridine, 1.0 equiv.) and LiBr (0.5 equiv.) in dioxane at 160 °C for 12 h, affords the cyclization product 3a with full site-specificity in 84% yield (Table 1, entry 1). Control reactions demonstrate that palladium catalyst, anhydride activator and base are essential to this reaction, with no or only a trace amount of 3a being detected in their absence (Table 1, entries 2–4). Other Pd(ii) catalysts, such as Pd(acac)_2_ or Pd(OAc)_2_, can also promote the reaction (Table 1, entry 5). Pd(0) catalysts, such as Pd_2_(dba)_3_, affords 3a in 50% yield under similar reaction conditions (Table 1, entry 6). Interestingly, NiCl_2_ is also a capable catalyst, providing a promising lead for future studies (Table 1, entry 7). Furthermore, when Boc_2_O and Ac_2_O are used instead of Piv_2_O as the activating reagent, the yield of 3a decreases (Table 1, entry 8). The yield is also lower when pyridine, Et_3_N or DBU are used instead of DMAP (Table 1, entry 9). The results might be ascribed to their weak nucleophilicity.^13^ Interestingly, LiBr plays an important role in this reaction;^14^ with only 34% yield of 3a being observed in its absence (Table 1, entry 10). LiF, LiCl and LiFePO_4_ can also serve as promoters, but their efficiency is lower (Table 1, entry 11). Furthermore, we established that KBr has a positive effect, but NaBr is ineffective (Table 1, entries 12). Next, we extensively investigated the phosphine ligand. In its absence, only 63% yield of 3a is obtained (Table 1, entry 13). Bidentate Xantphos and DPE-phos show good catalytic efficiency (Table 1, entry 14), while monodentate phosphines, such as (4-F-Ph)_3_P and (4-Me-Ph)_3_P also afford lower yields (Table 1, entries 14 and 15). Importantly, the reaction can take place in several ether solvents such as THF, DME and DEE (Table 1, entry 16), while toluene and hexane also afford similar yields (Table 1, entry 17). It should be noted that the reaction shows excellent regioselectivity; the byproduct 1,4-disubstituted naphthalene is not detected in the crude reaction mixtures by GC-MS. Additional optimization details are included in the ESI.†

**Table 1:** Reaction. Optimization^a^

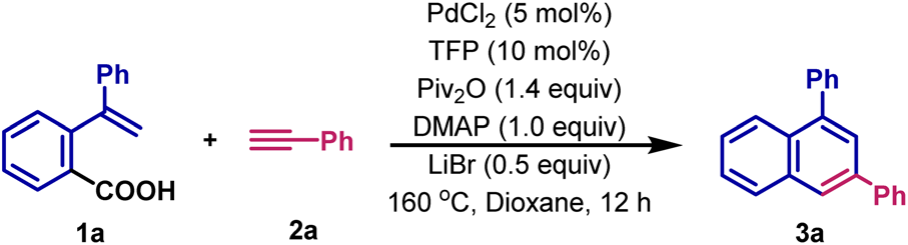		
Entry	Variation from the standard conditions	Yield^b^ of 3a (%)
1	None	84
2	Without Pd catalyst	N.D.
3	Without anhydride Piv_2_O	N.D.
4	Without base DMAP	Trace
5	Pd(acac)_2_ or Pd(OAc)_2_ instead of PdCl_2_	70/42
6	Pd2(dba)_3_ instead of PdCl_2_	50
6	NiCl_2_ instead of PdC1_2_	Trace
7	Boc_2_O, Ac_2_0 instead of Piv_2_O	29/56
9	Pyridine, Et_3_N or DBU instead of DMAP	Trace/12/43
10	Without LiBr	34
11	LiF, LiCl and LiFePO_4_ instead of LiBr	41/70/75
12	NaBr and KBr instead of LiBr	35/55
13	Without phosphine ligand	63
14	Xantphos and DPE-phos instead of TFP	73/68
15	(4-F-Ph)_3_P and (4-Me-Ph)_3_P instead of TFP	67/64
16	THF, DME and DEE instead of dioxane	49/50/52
17	Toluene and hexane instead of dioxane	53/52
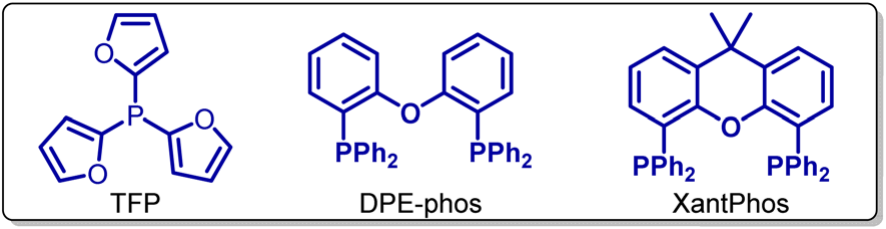		

a Reaction conditions: 2-(1-phenylvinyl)benzoic acid 1a (0.2 mmol), phenylacetylene 2a (0.4 mmol, 2.0 equiv. based on 1a), cat. [Pd] (5 mol%), ligand (10 mol%), anhydride (1.4 equiv.), DMAP (1.0 equiv.), solvent (2.0 mL), N_2_, 160 °C, 12 h.

b GC yield using dodecane as an internal standard.

### Scope studies

With the optimal reaction conditions in hand, the substrate scope for both carboxylic acids and terminal alkynes was then investigated. Importantly, we establish that the reaction shows excellent generality with respect to both reaction components. In terms of alkynes, both aromatic and aliphatic terminal alkynes including those with various functional groups work well under the reaction conditions. After isolation and purification, 3a is obtained in 77% yield. Substrates with electron-donating groups, such as alkyl (–Me, –^*t*^Bu), alkoxy (–OMe, –OPh), thiomethyl (–SMe) and phenyl (–Ph) at the benzene ring are readily converted into the corresponding naphthalenes in 57–89% yields (Table 2, 3b–3j). Halogen groups (F, Cl and Br) survive well to furnish the halogenated products in 53–73% yields (Table 2, 3k–3p). These products provide functional handles that can be easily further functionalized by traditional cross-coupling reactions, demonstrating orthogonality of the carboxylic acid electrophile. Likewise, high yields are also obtained from substrates containing a range of electron-withdrawing groups, such as OCF_3_, CF_3_, ester, acetyl, CN or formyl at the benzene ring (Table 2, 3q–3v). To our delight, the π-extended terminal alkynes, such as 2-ethynylnaphthalene and ethylnylferrocene can produce the cyclized products in good yields (Table 2, 3w–3x). It is further worth noting that chelating heterocycles that can coordinate with transition-metals are compatible under the reaction conditions, including thiophenes (Table 2, 3y–3z), pyridine (Table 2, 3aa), quinoline (Table 2, 3ab), *N*-methyl-2-pyridinone (Table 2, 3ac) and thiochromane (Table 2, 3ad), all of these substrates are smoothly transformed into the corresponding cyclizing products in good to high yields, furnishing π-conjugates naphthalene products that are relevant in the synthesis of advanced materials.

**Table 2:** Substrate scope of carboxylic acids and terminal alkynes^a^

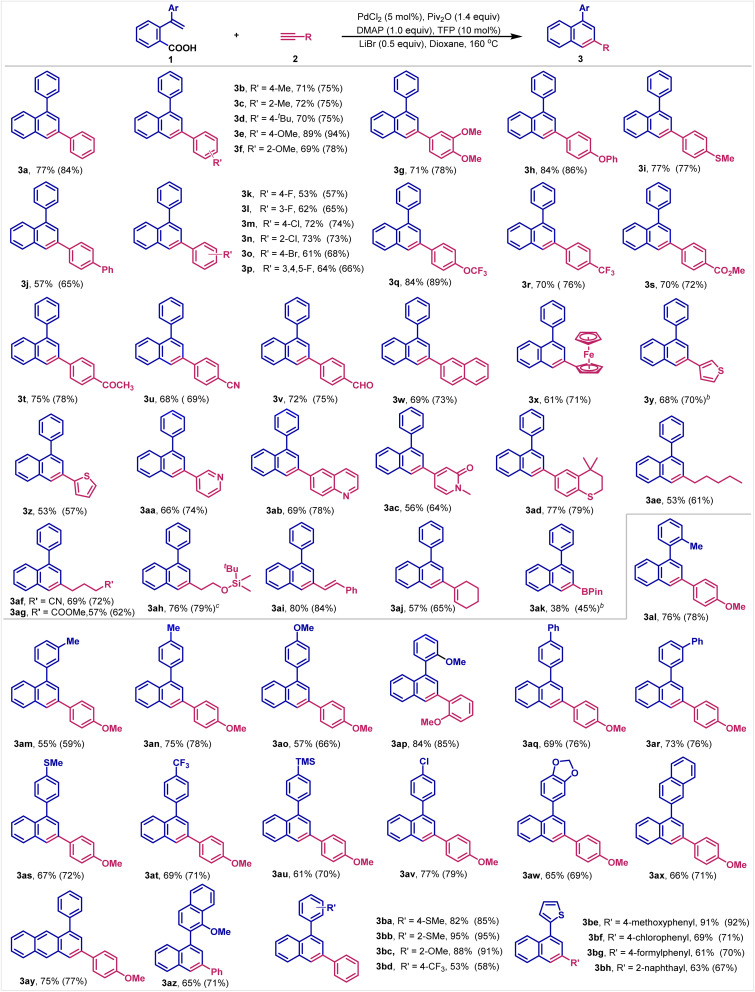

a Reaction conditions: carboxylic acid 1 (0.2 mmol), terminal alkyne 2 (0.4 mmol, 2.0 equiv.), PdCl_2_ (5 mol%), TFP (10 mol%), Piv_2_O (0.28 mmol, 1.4 equiv.), dioxane (2.0 mL), N_2_, 160 °C, 12 h. Isolated yield. GC yield is shown in parentheses.

b Terminal alkyne 2 (4.0 equiv.) is used.

c LiI instead of LiBr.

Furthermore, we are pleased to learn that in addition to aromatic terminal alkynes, aliphatic alkynes are also applicable to this reaction and afford the corresponding products with full site-selectivity. For example, 1-heptyne reacts well with 1a to give the desired 3-alkyl-naphthalene (Table 2, 3ae). Alkynes decorated with cyano, ester and siloxy groups prove to be effective substrates (Table 2, 3af–3ah). It is worth noting that alkenyl double bonds are well-tolerated under the reaction conditions (Table 2, 3ai and 3aj). Furthermore, the reaction enables to introduce even an organoboronic ester into the naphthalene framework, demonstrating the functional group orthogonality of the carboxylic acid decarbonylative annulation approach (Table 2, 3ak). However, ethyl propiolate and ethoxyethyne did not work under the reaction conditions. The results might be ascribed to their low boiling points. Internal alkynes like diphenylacetylene and prop-1-yn-1-ylbenzene were not suitable to this reaction either, since the corresponding products were only detected in less than 10% yields.

With respect to the carboxylic acid component, the reaction also shows high functional group tolerance. Thus, alkyl, methoxy, phenyl, methylthio, trimethylsilyl, chloro and trifluoromethyl groups are well-tolerated under the reaction conditions (Table 2, 3al–3av). To our delight, large conjugated π-systems can be efficiently constructed by the strategy as exemplified by the anthracenyl and binaphthyl rings (Table 2, 3aw–3az). These products have found wide applications in photo-material chemistry. Finally, selectivity studies with different terminal alkynes and carboxylic acids are conducted, affording the corresponding naphthalenes in 53–95% yields with full site-selectivity, further demonstrating the generality of this new reaction (Table 2, 3ba–3bh).

### Synthesis of molecules bearing bioactive fragment

**Scheme 2:**
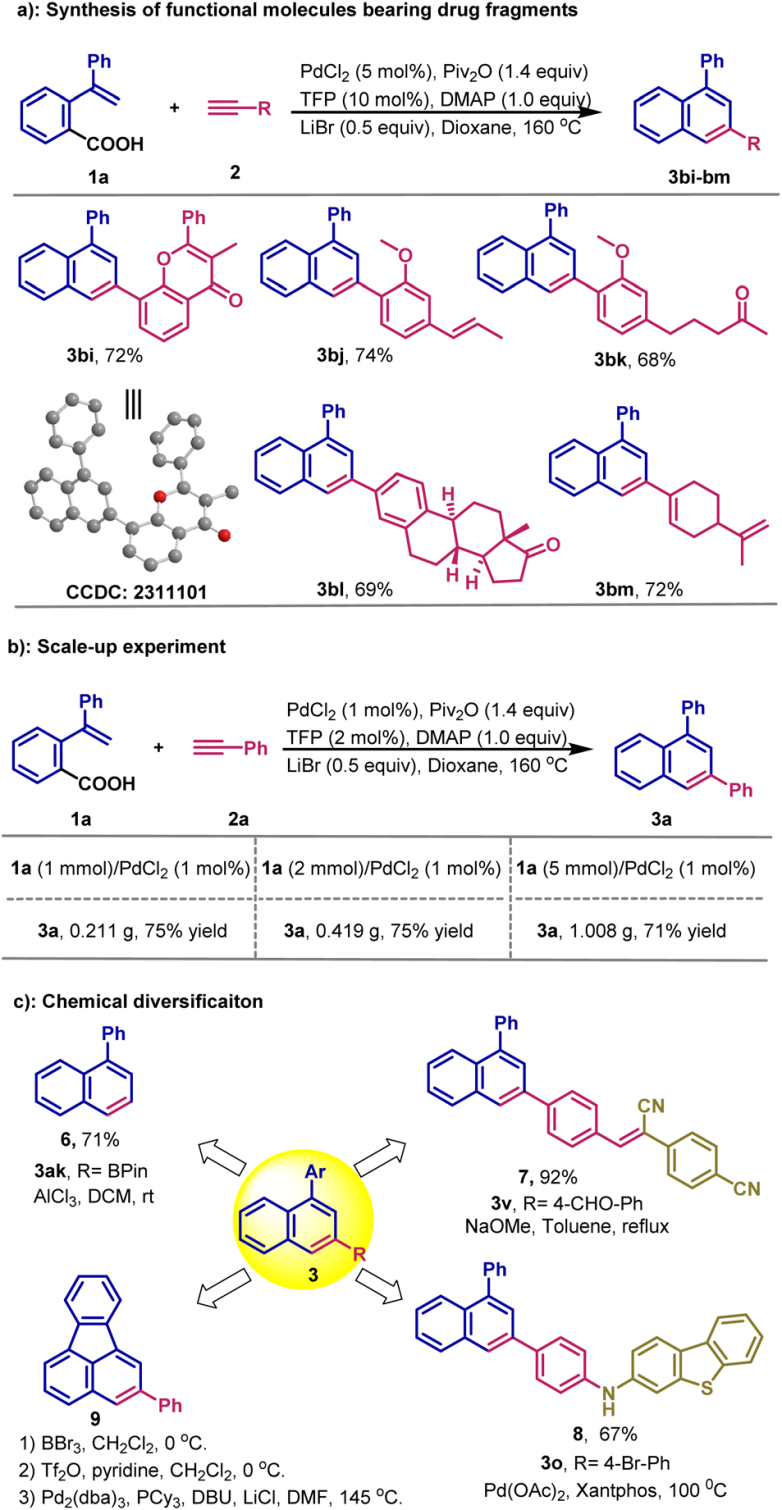
Synthetic applications. (a) Scale-up experiments. (b) Synthesis of functional molecules bearing drug fragments. (c) Chemical diversification.

We demonstrate that this novel method enables facile introduction of bioactive molecular fragments into the naphthalene skeleton. For example, naphthalene with an antioxidant flavone fragment is generated in 72% yield under the standard reaction conditions (Scheme 2b, 3bi). The molecular structure is determined by X-ray analysis (CCDC number: 2311101), unambiguously confirming the site-selectivity of the cycloaddition. By the strategy, estrone, isoeugenol, flavone, zingiberone and perillaldehyde fragments are also smoothly introduced (Scheme 2b, 3bj–3bm), demonstrating the potential of this new method in medicinal chemistry and natural products campaigns.

### Scale-up

To further evaluate the practicality of this new method, a scale-up reaction was conducted. As shown in Scheme 2a, when the reaction of 1a and 2a is performed on a 1 mmol scale, 3a is produced in 75% yield (0.211 g). High yields are also obtained at 2 mmol and 5 mmol scales. It should be noted that only 1 mol% PdCl_2_ is required in these reactions, demonstrating efficiency of the decarbonylative process.

### Chemical diversification

As demonstrated by the scope studies, we find that this novel reaction tolerates a variety of electrophilic groups, which could facilitate further derivatization to synthesize target functional molecules not easily available by other methods (Scheme 2c). For example, we show that 3ak can readily undergo protodeborylation to produce the corresponding 1-substituted naphthalene 6. Furthermore, product 3v with a formyl group readily reacts with benzeneacetonitrile to afford the fluorescent photochromic 7, which exhibits distinct light-triggered changes in the emission colors.^15^ Similarly, product 3o with a Br substituent can produce the diarylamine product 8 through a conventional Buchwald–Hartwig cross-coupling. It is worth noting is that compound 8 is a structural segment of organic electroluminescent devices.^16^ Furthermore, dibenzofluoranthenes, such as 9, can be readily synthesized from product 3ap.^17^ These results highlight the practical value of this new site-selective decarbonylative cycloaddition of carboxylic acids in organic synthesis.

### Mechanistic studies

**Scheme 3:**
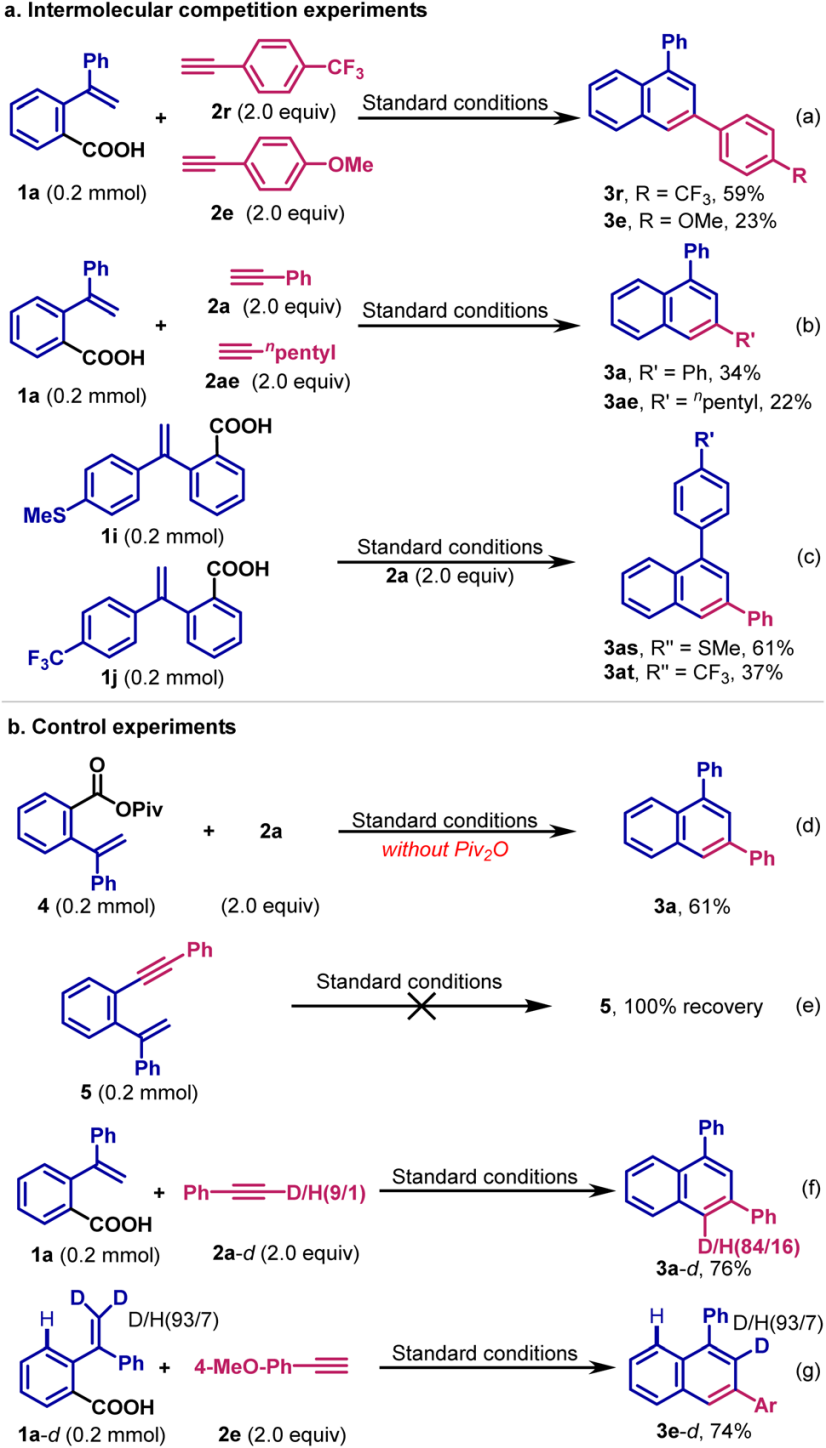
Competition experiments, control experiments and deuterium experiments.

To gain mechanistic insight into the reaction, control experiments are performed (Scheme 3). First, to probe the selectivity of this reaction, intermolecular competition experiments are conducted. When 4-trifluoromethyl phenyl acetylene 2r and 4-methoxyl phenyl acetylene 2e react with carboxylic acid 1a, the corresponding products 3r and 3e are produced in 59% and 23% yields, respectively (Scheme 3a). When phenylacetylene 2a is allowed to compete with hept-1-yne 2ae, 3a is obtained in 34% yield, and 3ae is generated in 22% yield (Scheme 3b). These results indicate that the reaction favors electron-deficient terminal alkynes. Furthermore, when phenylacetylene 2a is reacted with carboxylic acids 1i and 1j, the product 3as is produced in 61% yield, and 3at in 37% yield, indicating that electron-rich carboxylic acids are favored (Scheme 3c).

Next, we hypothesize that carboxylic acid is firstly activated *in situ* to produce the active mixed anhydride. Indeed, we have synthesized the mixed anhydride 4 and allowed it to react with phenyl acetylene 2a, resulting in 65% yield of 3a under similar reaction conditions (Scheme 3d). During the reaction of 1a with 2a, the *ipso*-decarbonylative product 5 is detected by GC-MS. To probe the role of 5 as the intermediate, which could undergo intramolecular cyclization to produce the target product, we synthesized 5 and subjected it to the reaction conditions (Scheme 3e). However, we find that 3a is not generated and 5 is quantitatively recovered. When carboxylic acid 1a reacts with deuterated 2a-*d*, 3a-*d* is produced in 76% yield with 84% deuterium incorporation at the 4-position (Scheme 3f). We also synthesized deuterated carboxylic acid 1a-*d*, and reacted it with 4-methoxyl phenyl acetylene 2e to produce 3e-*d* in 74% yield under the reaction conditions (Scheme 3g). In compound 3e-*d*, the C2-position is fully deuterated, while no deuterium is detected at the C4-position. These results indicate that 5 is not the intermediate of this decarbonylative cycloaddition. Furthermore, the *ortho*-vinyl group remained intact during the reaction, while the decrease of deuterium incorporation at the C4-position in 3a-*d* could be ascribed to the hydrogen–deuterium exchange of terminal alkyne under the reaction conditions.

### Kinetic studies

**Fig. 1:**
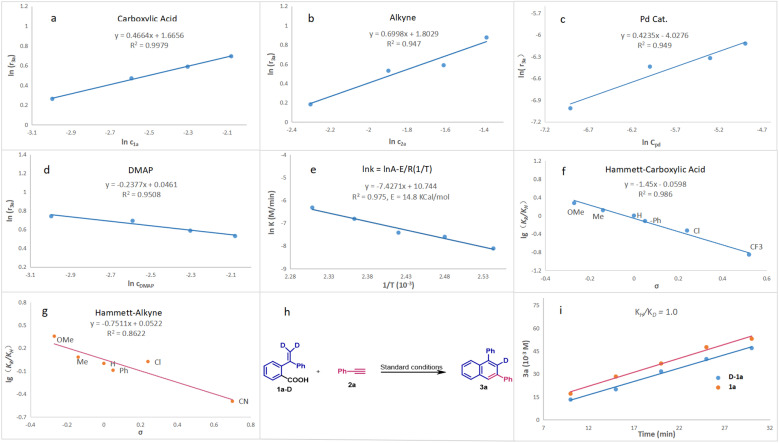
Kinetic analysis, Hammett analysis and KIE experiments. (a) Carboxylic acid *vs.* initial rate. (b) Alkyne *vs.* initial rate. (c) Pd-cat *vs.* initial rate. (d) DMAP *vs.* initial rate. (e) Arrhenius plots: plot of ln *k vs.* 1/*T*. (f) *para*-Substituted carboxylic acids with alkyne. (g) *para*-Substituted alkynes with carboxylic acid. (h and i) Kinetic isotopic effects (KIE).

**Scheme 4:**
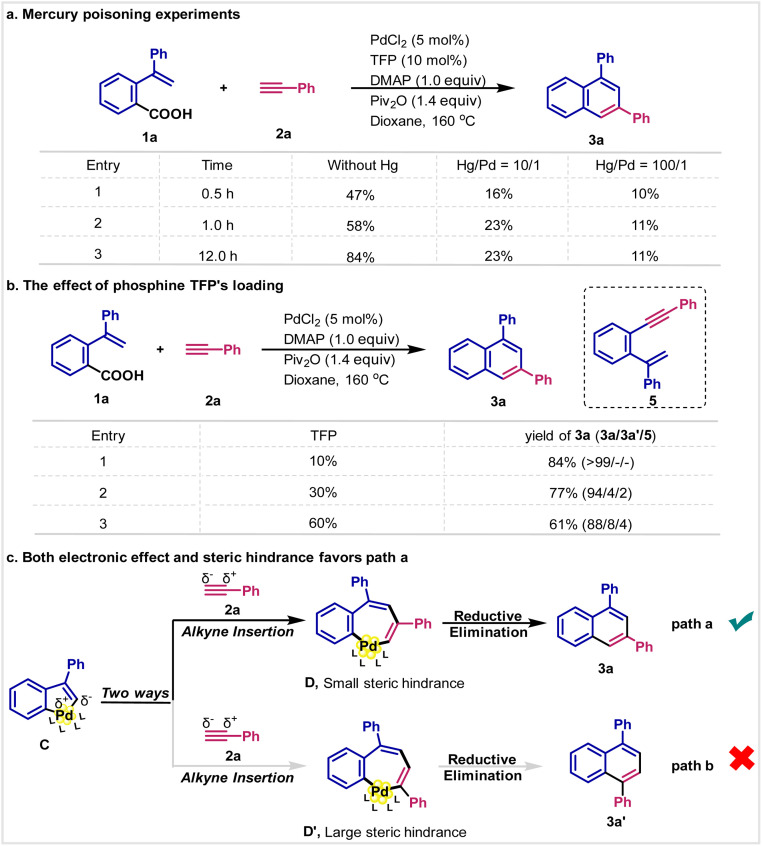
The investigation of reaction selectivity.

The kinetic analysis was subsequently conducted (Fig. 1). We find that the reaction shows half-order dependence on carboxylic acid and Pd-catalyst. For terminal alkynes and DMAP, the rate is 0.7-order and zero-order, respectively (Fig. 1a–d). The activation energy is also calculated based on the reaction temperature and the value is 14.8 kcal mol^−1^. Since the reaction is performed at 160 °C, the intrinsic activation energy would usually be more than 30 kcal mol^−1^. This suggests that the reaction might be mediated by palladium clusters, leading to a difference by diffusion. To validate this hypothesis, the mercury poisoning experiments are conducted (Scheme 4a). We find that mercury can inhibit the reaction; in particular, when 100 equiv. mercury (calculated on the basis of palladium) is used, the reaction is almost completely inhibited. These results show that the reaction involves a heterogeneous process.^18^ Moreover, we analyzed the reaction mixture by MALDI-TOF MS and found some fragments such Pd2, Pd4 and Pd5, further supporting the formation of palladium clusters. Subsequently, we investigated the effect of ligand's loading. It is found that the yield of 3a decreased as the increase of phosphine TFP's loading (Scheme 4b). Worth noting is that the selectivity also decreased. Under the standard reaction conditions, the isomer 3a′ and the *ipso*-coupling product 5 almost could not be detected by GC-MS, while both 3a′ and 5 were generated when more TFP was added. It is known that ligand would stabilize the molecular palladium complexes, stopping the formation of palladium cluster. Thus, those results might be ascribed to the delayed formation of Pd cluster.^19^

As described below, complex C would be the key intermediate in this reaction (Scheme 4c). Considering from the steric hindrance, compared with C_aryl_–Pd bond, it would be easier for terminal alkynes to insert into C_vinyl_–Pd bond. In addition, both electronic and steric factors would favor path a to give isomer 3a. In this reaction, the steric hindrance is further increased due to the formation of palladium clusters, thus very high selectivity to 3a is observed. Finally, we performed Hammett analysis (Fig. 1f–h). A negative slope (*ρ* = −1.45) is observed for the reaction of *para*-substituted carboxylic acids 1 with phenylacetylene 2a (Fig. 1f). A comparatively smaller value (*ρ* = −0.75) is found for the reaction of *para*-substituted phenylacetylenes 2 with carboxylic acid 1a (Fig. 1g). These results indicate that the reaction is more affected by the electronic properties of the carboxylic acid. Furthermore, kinetic isotope experiments (KIE) of 2a-*d* with 3a are performed (Fig. 1h). A small kinetic isotope effect (*k*_H_/*k*_D_ = 1.0) is obtained, indicating that the C–H cleavage is not the rate-determining step in this reaction.

### Proposed mechanism

**Scheme 5:**
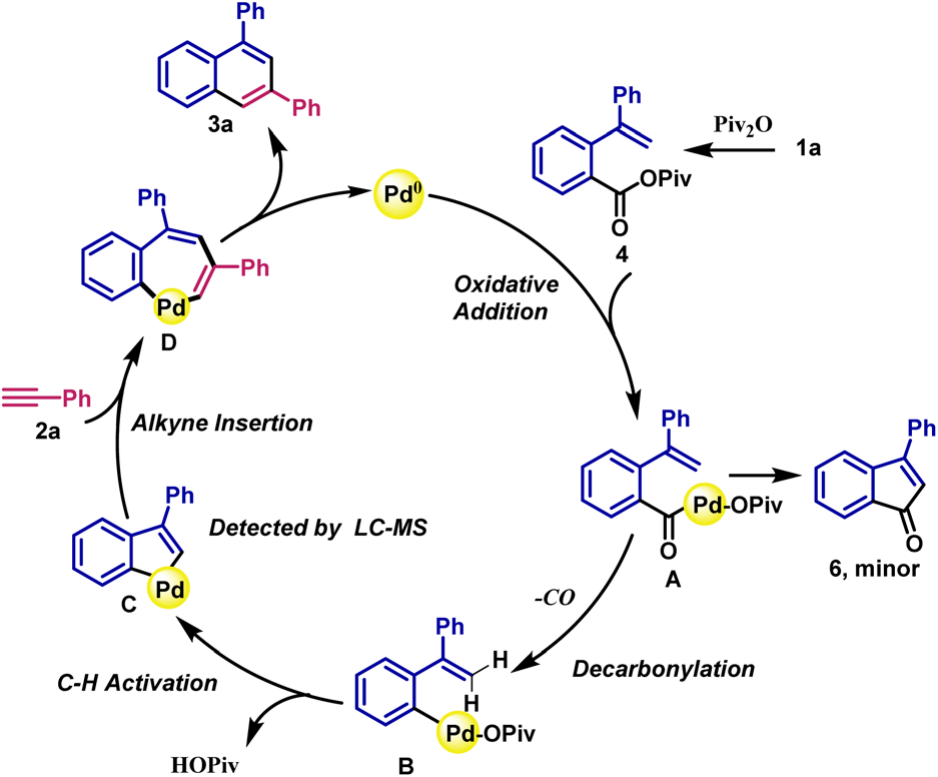
Proposed mechanism. For clarity, the ligand is omitted.

On the basis of mechanistic studies, a plausible mechanism involving palladacycle formation by a sequential C–C/C–H activation and a subsequent regioselective insertion of terminal alkynes is proposed (Scheme 5). As shown in Scheme 5, carboxylic acid 1a is firstly activated by anhydride Piv_2_O to produce the mixed anhydride 4. Then, the acyl C–O bond oxidatively adds to the active Pd(0) catalyst generated *in situ* to afford A. This acyl–Pd intermediate undergoes decarbonylation and vinnyl C–H cleavage by the assistance of PivO^−^ anion forming the five-membered palladacycle C.^20^ The subsequent insertion of terminal alkyne proceeds with exquisite regioselectivity to generate the seven-membered arylpalladium D. Finally, reductive elimination of D produces the target product 3 and regenerates the active Pd(0) catalyst.^21^ The byproduct 6 obtained from intermediate A through intramolecular C–H activative cyclization can be observed by GC-MS. It should be noted that fragment of intermediates B, C and D have been detected by MALDI-TOF MS (see ESI†), further supporting the proposed decarbonylative C–C/C–H catalytic cycle. It should be mentioned that the detected fragment of intermediates B, C and D did not contain phosphine ligand. The result might be ascribed to the relatively weak coordination between Pd and phosphine ligand as well as the weak Pd–Pd bonds in cluster.

In conclusion, we have disclosed an exquisitely site-selective decarbonylative cycloaddition of carboxylic acids with terminal alkynes by sequential C–C/C–H bond activation. Most notably, this method represents the first use of carboxylic acids as the ubiquitous and underdeveloped synthons for intramolecular cycloadditions by decarbonylative C–C bond cleavage. Furthermore, the method provides a solution to the challenge of the regioselective synthesis of substituted naphthalenes by cycloaddition. Wide substrate scope for both carboxylic acids and terminal alkynes is demonstrated with excellent functional group tolerance. In addition, the reaction utility is demonstrated in the construction of complex molecules bearing bioactive fragments and product derivatization through readily tolerated active groups. Extensive mechanistic studies show this reaction takes place through formation of a key five-membered palladacycle and regioselective transfer insertion of terminal alkynes determining the high regio-selectivity of this process by the formation of palladium clusters. This reaction advances the toolbox of redox-neutral carboxylic acid interconversion to cycloaddition processes. This reactivity platform is likely to find wide application in organic synthesis, drug discovery and functional material research fields.

## Data availability

The ESI† includes all experimental details, including optimization of the synthetic method, synthesis and characterization of all starting materials and products reported in this study, and mechanistic studies. NMR spectra of all products reported are included as well.

## Author contributions

M. C. performed the experiments. M. S. and T. C. conceived the project and wrote the manuscript. X. M., X. Y., S. Z. and L. L. provided crucial suggestions to the main concept of the project and revised the manuscript.

## Conflicts of interest

There are no conflicts to declare.

## Supplementary Material

SC-OLF-D4SC05429F-s001

SC-OLF-D4SC05429F-s002
