# Supplementary material for: Site-selective decarbonylative [4 + 2] annulation of carboxylic acids with terminal alkynes by C–C/C–H activation strategy and cluster catalysis
Source: Chem Sci. 2024 Nov 14;15(48):20346–54. doi: 10.1039/d4sc05429f (PMC11577269; doi:10.1039/d4sc05429f)

Supplementary Information (SI) for Chemical Science.
This journal is © The Royal Society of Chemistry 2024

Electronic Supplementary Information

Site-Selective Decarbonylative [4+2] Annulation of Carboxylic Acids with Terminal Alkynes by C−C/C−H Activation Strategy and Cluster Catalysis

Mengjie Cen,[a,b] Xinyue Ma,[a] Xi Yang,[a] Shangshang Zhang,[a] Long Liu,[a] Michal Szostak,*[,c] and Tieqiao Chen*[,a]

[a]School of Chemistry and Chemical Engineering, Hainan University, Haikou, 570228, China

[b]Hainan Research Academy of Environmental Sciences, Haikou, 571127, PR China

[c]Department of Chemistry, Rutgers University, 73 Warren Street, Newark, NJ 07102, United States

Table of Contents

1. General Information······ S3

2. Experimental Procedure······ S3

2.1 Optimization of Reaction Conditions······ S3

2.2 Preparation of the Starting Material······ S6

2.3 Control Experiments······ S9

2.4 Intermolecular Competition Experiments······ S10

2.5 Substituent Effect Experiments······ S11

2.6 Reaction Progress Kinetic Analysis······ S12

2.7 Mercury Poisoning Experiments······ S14

2.8 General Procedure for Preparation of Product 3······ S14

2.9 Derivatization Experiments······ S15

2.10 Measurement of Intermediate by MALDI-TOF MS······ S16

3. X-ray Crystallographic Data······ S18

4. Characterization Data for the Products······ S31

5. References······ S76

6. Copies of ^{1}H, ^{13}C and ^{19}F NMR Spectra of the Products······ S77

1. General Information

All experiments were carried out under nitrogen atmosphere using standard Schlenk techniques. Solvents were dried over Na or CaH_2, and were distilled under nitrogen prior to use. Reagents were of analytical grade, obtained from commercial suppliers and used without further purification. Column chromatography was performed using Silica Gel (200–300 mesh). The reactions were monitored by GC and GC-MS. GC-MS results were recorded on GC-MS QP 2010 plus, and GC analysis was performed on GC 2014. The ^{1}H, ^{13}C, and ^{19}F NMR spectra were recorded on a Bruker ADVANCE III spectrometer at 400 MHz, 100 MHz, and 376 MHz respectively, and chemical shifts were reported in parts per million (ppm). All solvents and reagents were purchased from Energy Chemical, Alfa Aesar, Heowns, Meryer and Aladdin.

2. Experimental Procedure

2.1 Optimization of Reaction Conditions

Table **S1** Reaction Optimization

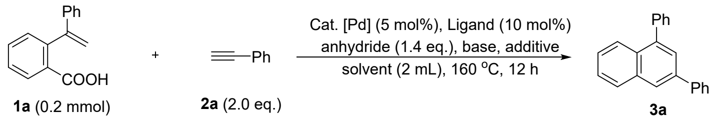

entry	Pd-Cat.	Ligand	additive	base	solvent	yield
1[a]	$Pd(OAc)_2$	$(4\text{-Cl-Ph})_3P$	-	DMAP (1.5 eq.)	THF	trace
2[a]	$Pd(acac)_2$	$(4\text{-Cl-Ph})_3P$	-	DMAP (1.5 eq.)	THF	trace
3[a]	$Pd(TFA)_2$	$(4\text{-Cl-Ph})_3P$	-	DMAP (1.5 eq.)	THF	trace
4[a]	$PdCl_2$	$(4\text{-Cl-Ph})_3P$	-	DMAP (1.5 eq.)	THF	7%
5[a]	$Pd(dba)_2$	$(4\text{-Cl-Ph})_3P$	-	DMAP (1.5 eq.)	THF	trace
6[a]	$Pd_2(dba)_3$	$(4\text{-Cl-Ph})_3P$	-	DMAP (1.5 eq.)	THF	trace
7[a]	$Pd(MeCN)_2Cl_2$	$(4\text{-Cl-Ph})_3P$	-	DMAP (1.5 eq.)	THF	4%
8[a]	$Pd(PPh_3)_2Cl_2$	$(4\text{-Cl-Ph})_3P$	-	DMAP (1.5 eq.)	THF	5%
9[a]	$Pd(dppp)_2Cl_2$	$(4\text{-Cl-Ph})_3P$	-	DMAP (1.5 eq.)	THF	trace
10[a]	$Pd(PhCN)_2Cl_2$	$(4\text{-Cl-Ph})_3P$	-	DMAP (1.5 eq.)	THF	5%
11[a]	$Pd(cod)_2Cl_2$	$(4\text{-Cl-Ph})_3P$	-	DMAP (1.5 eq.)	THF	7%
12[a]	$Pd(PPh_3)_4$	$(4\text{-Cl-Ph})_3P$	-	DMAP (1.5 eq.)	THF	trace

13[a]	$Pd(cod)_2Cl_2$	$(4\text{-}Cl\text{-}Ph)_3P$	-	Et_3N (1.5 eq.)	THF	3%
14[a]	$Pd(cod)_2Cl_2$	$(4\text{-}Cl\text{-}Ph)_3P$	-	Li_2CO_3 (1.5 eq.)	THF	15%
15[a]	$Pd(cod)_2Cl_2$	$(4\text{-}Cl\text{-}Ph)_3P$	-	Na_2CO_3 (1.5 eq.)	THF	trace
16[a]	$Pd(cod)_2Cl_2$	$(4\text{-}Cl\text{-}Ph)_3P$	-	K_2CO_3 (1.5 eq.)	THF	n.d.
17[a]	$Pd(cod)_2Cl_2$	$(4\text{-}Cl\text{-}Ph)_3P$	-	$NaHCO_3$ (1.5 eq.)	THF	n.d.
18[a]	$Pd(cod)_2Cl_2$	$(4\text{-}Cl\text{-}Ph)_3P$	-	$KHCO_3$ (1.5 eq.)	THF	n.d.
19[a]	$Pd(cod)_2Cl_2$	$(4\text{-}Cl\text{-}Ph)_3P$	-	HCOOLi (1.5 eq.)	THF	trace
20[a]	$Pd(cod)_2Cl_2$	PPh_3	Li_2CO_3 (1.5 eq.)		THF	11%
21[a]	$Pd(cod)_2Cl_2$	$(4\text{-}Me\text{-}Ph)_3P$	Li_2CO_3 (1.5 eq.)		THF	4%
22[a]	$Pd(cod)_2Cl_2$	TFP	Li_2CO_3 (1.5 eq.)		THF	16%
23[a]	$Pd(cod)_2Cl_2$	Ph_2P(2-Py)	Li_2CO_3 (1.5 eq.)	-	THF	8%
24[a]	$Pd(cod)_2Cl_2$	dppf	Li_2CO_3 (1.5 eq.)	-	THF	6%
25[a]	$Pd(cod)_2Cl_2$	dppm	Li_2CO_3 (1.5 eq.)	-	THF	12%
26[a]	$Pd(cod)_2Cl_2$	dppe	Li_2CO_3 (1.5 eq.)	-	THF	8%
27[a]	$Pd(cod)_2Cl_2$	dppp	Li_2CO_3 (1.5 eq.)	-	THF	3%
28[a]	$Pd(cod)_2Cl_2$	dppb	Li_2CO_3 (1.5 eq.)	-	THF	trace
29[a]	$Pd(cod)_2Cl_2$	dpph	Li_2CO_3 (1.5 eq.)	-	THF	4%
30[a]	$Pd(cod)_2Cl_2$	dppbenz	Li_2CO_3 (1.5 eq.)	-	THF	trace
31[a]	$Pd(cod)_2Cl_2$	XantPhos	Li_2CO_3 (1.5 eq.)	-	THF	13%
32[a]	$Pd(cod)_2Cl_2$	Bpy	Li_2CO_3 (1.5 eq.)	-	THF	8%
33[a]	$Pd(cod)_2Cl_2$	Tpy	Li_2CO_3 (1.5 eq.)	-	THF	7%
34[a]	$Pd(cod)_2Cl_2$	1,10-phen	Li_2CO_3 (1.5 eq.)	-	THF	16%
35[a]	$Pd(cod)_2Cl_2$	-	Li_2CO_3 (1.0 eq.)	-	THF	10%
36[a]	$Pd(cod)_2Cl_2$	TFP	LiCl (0.5 eq.)	-	THF	27%
37[a]	$Pd(cod)_2Cl_2$	TFP	LiCl (1.0 eq.)	-	THF	27%
38[a]	$Pd(cod)_2Cl_2$	TFP	LiCl (2.0 eq.)	-	THF	28%
39[a]	$Pd(cod)_2Cl_2$	TFP	LiCl (3.0 eq.)	-	THF	26%
40[a]	$Pd(cod)_2Cl_2$	TFP	LiCl (2.0 eq.)	Et_3N (1.0 eq.)	THF	14%
41[a]	$Pd(cod)_2Cl_2$	TFP	LiCl (2.0 eq.)	DMAP (1.0 eq.)	THF	33%

42[a]	$Pd(cod)_2Cl_2$	TFP	LiCl (2.0 eq.)	DBU (1.0 eq.)	THF	13%
43[a]	$Pd(cod)_2Cl_2$	TFP	LiCl (2.0 eq.)	BDMA (1.0 eq.)	THF	16%
44[a]	$Pd(cod)_2Cl_2$	TFP	LiCl (2.0 eq.)	TMEDA (1.0 eq.)	THF	trace
45[a]	$Pd(cod)_2Cl_2$	TFP	LiCl (2.0 eq.)	1,10-phen (1.0 eq.)	THF	n.d.
46[a]	$Pd(cod)_2Cl_2$	TFP	LiCl (2.0 eq.)	TEDA (1.0 eq.)	THF	trace
47[a]	$Pd(cod)_2Cl_2$	TFP	LiCl (2.0 eq.)	Bpy (1.0 eq.)	THF	6%
48[a]	$Pd(cod)_2Cl_2$	TFP	LiCl (2.0 eq.)	DMAP (1.0 eq.)	THF	13%
49[a]	$Pd(cod)_2Cl_2$	TFP	LiCl (2.0 eq.)	DMAP ,(1.0 eq.)	Cy	6%
50[a]	$Pd(cod)_2Cl_2$	TFP	LiCl (2.0 eq.)	DMAP (1.0 eq.)	Toluene	6%
51[a]	$Pd(cod)_2Cl_2$	TFP	LiCl (2.0 eq.)	DMAP (1.0 eq.)	dioxane	38%
52[a]	$Pd(cod)_2Cl_2$	TFP	LiCl (2.0 eq.)	DMAP (1.0 eq.)	DME	18%
53[a]	$Pd(cod)_2Cl_2$	TFP	LiCl (2.0 eq.)	DMAP (1.0 eq.)	DEE	8%
54[a]	$Pd(cod)_2Cl_2$	TFP	LiCl (2.0 eq.)	DMAP (1.0 eq.)	2-Me-THF	13%
55[a]	$Pd(cod)_2Cl_2$	TFP	LiBr (3.0 eq.)	DMAP (1.0 eq.)	dioxane	61%
56[a]	$Pd(cod)_2Cl_2$	TFP	LiBr (2.0 eq.)	DMAP (1.0 eq.)	dioxane	65%
57[a]	$Pd(cod)_2Cl_2$	TFP	LiBr (1.5 eq.)	DMAP (1.0 eq.)	dioxane	65%
58[a]	$Pd(cod)_2Cl_2$	TFP	LiBr (1.0 eq.)	DMAP (1.0 eq.)	dioxane	70%
59[a]	$Pd(cod)_2Cl_2$	TFP	LiBr (0.5eq.)	DMAP (1.0 eq.)	dioxane	74%
60[a]	$Pd(cod)_2Cl_2$	TFP	LiBr (0.25eq.)	DMAP (1.0 eq.)	dioxane	65%
61[a]	$Pd(cod)_2Cl_2$	TFP	LiBr (0.1 eq.)	DMAP (1.0 eq.)	dioxane	74%
62[b]	$Pd(cod)_2Cl_2$	TFP	LiBr (0.5 eq.)	DMAP (1.0 eq.)	dioxane	78%
63[c]	$Pd(cod)_2Cl_2$	TFP	LiBr (0.5 eq.)	DMAP (1.0 eq.)	dioxane	79%
64	$Pd(cod)_2Cl_2$	TFP	LiBr (0.5 eq.)	DMAP (1.0 eq.)	dioxane	79%
65	$PdCl_2$	TFP	LiBr (0.5 eq.)	DMAP (1.0 eq.)	dioxane	84%
66	$Pd(OAc)_2$	TFP	LiBr (0.5 eq.)	DMAP (1.0 eq.)	dioxane	42%
67	$Pd(acac)_2$	TFP	LiBr (0.5 eq.)	DMAP (1.0 eq.)	dioxane	70%
68	$NiCl_2$	TFP	LiBr (0.5 eq.)	DMAP (1.0 eq.)	dioxane	trace
69	$PdCl_2$	TFP	HCO_2Li (0.5 eq.)	DMAP (1.0 eq.)	dioxane	18%
70	$PdCl_2$	TFP	Li_3PO_4 (0.5 eq.)	DMAP (1.0 eq.)	dioxane	66%

71	$PdCl_2$	TFP	LiH_2PO_4 (0.5 eq.)	DMAP (1.0 eq.)	dioxane	62%
72	$PdCl_2$	TFP	$LiPO_3$ (0.5 eq.)	DMAP (1.0 eq.)	dioxane	74%
73	$PdCl_2$	TFP	$LiFePO_4$ (0.5 eq.)	DMAP (1.0 eq.)	dioxane	75%
74	$PdCl_2$	TFP	LiF (0.5 eq.)	DMAP (1.0 eq.)	dioxane	41%
75	$PdCl_2$	TFP	LiCl (0.5 eq.)	DMAP (1.0 eq.)	dioxane	70%
76	$PdCl_2$	TFP	LiI (0.5 eq.)	DMAP (1.0 eq.)	dioxane	75%
77	$PdCl_2$	DPE-phos	LiBr (0.5 eq.)	DMAP (1.0 eq.)	dioxane	68%
78	$PdCl_2$	Xantphos	LiBr (0.5 eq.)	DMAP (1.0 eq.)	dioxane	73%
79	$PdCl_2$	(4-Me-Ph)$_3$P	LiBr (0.5 eq.)	DMAP (1.0 eq.)	dioxane	64%
80	$PdCl_2$	Ph_2P(2-Py)	LiBr (0.5 eq.)	DMAP (1.0 eq.)	dioxane	45%
81	$PdCl_2$	TFP	NaBr (0.5 eq.)	DMAP (1.0 eq.)	dioxane	35%
82	$PdCl_2$	TFP	KBr (0.5 eq.)	DMAP (1.0 eq.)	dioxane	55%

[a]Terminal alkyne **2a** (1.2 eq.). [b]Terminal alkyne **2a** (1.6 eq.). [c]Terminal alkyne **2a** (1.8 eq.). Abbr.: DMAP (4-dimethylaminopyridine), dppm (Bis(diphenylphosphino)methane), dppe (1,2-Bis(diphenylphosphino)ethane), dppp (1,3-Bis(diphenylphosphino)propane), dppb (1,4-Bis(diphenylphosphino)butane), dppbenz (1,2-Bis(diphenylphosphanyl)benzene), dpph (1,6-Bis(diphenylphosphino)hexane), dppf (1,1'-Bis(diphenylphosphino)ferrocene), BDMA (N,N-Dimethylbenzylamine), TEDA (Triethylenediamine), TOA (Trioctyl amine), Cy (cyclohexane), DME (1,2-Dimethoxyethane), DEE (1,2-Diethoxyethene).

2.1 Preparation of the Starting Materials

Procedure I for the synthesis of carboxylic acids

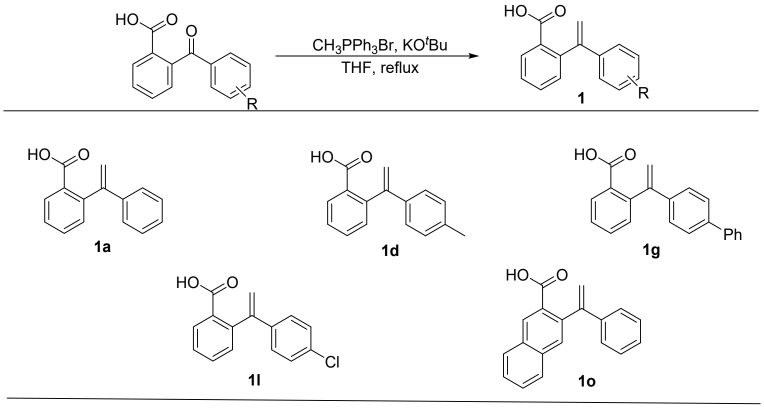

Step: To a stirred suspension of methyltriphenylphosphonium bromide (2.0 eq.) in THF (1M) Potassium *tert*-butoxide (3.0 eq.) was added at 0 °C and stirred for 0.5 hours under N_2. A solution of 2-

ketobenzoic acid (10 mmol, 1.0 eq.) in THF (3 M) was added dropwise. Until the reaction was completed, the resulting mixture was quenched with water, acidified with aqueous HCl (2.0 M) until pH 1-2 and extracted twice with ethyl acetate. The combined organic layers were washed with water and brine, dried over Na_2SO_4, filtered and concentrated in vacuo. The resulting crude product was purified by flash column chromatography to afford the desired unsaturated benzoic acid derivative in 78-95% yield.

Procedure II for the synthesis of carboxylic acids

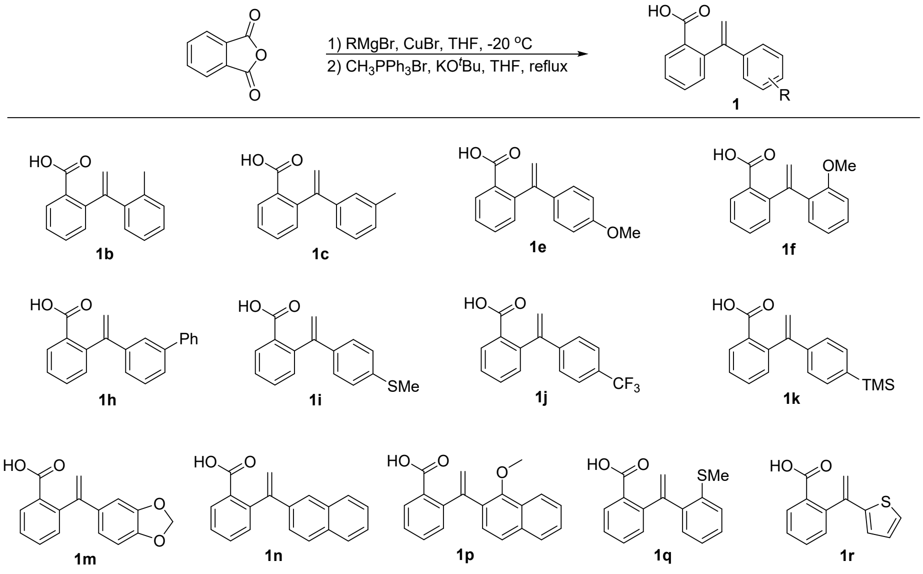

Step: To a flame-dried flask under nitrogen atmosphere were added isobenzofuran-1,3-dione (1.0 eq.), cuprous bromide (0.07 eq.) and anhydrous THF (1.0 M). The resulting suspension was cooled down to -20 °C and a solution of RMgBr (1.1 eq.) dissolved in THF was added dropwise over 1 h. The reaction mixture was stirred overnight at -20 °C, then allowed to warm to room temperature, quenched with water, basified with aqueous NaOH (3.0 M) until pH 12-14 and washed with diethyl ether. The resulting aqueous phase was acidified with aqueous HCl (2.0 M) until pH 1-2 and extracted twice with ethyl acetate. The combined organic layers were washed with water, brine, dried over Na_2SO_4, filtered and concentrated in vacuo. The resulting crude product was used for the next step without further purification.

To a stirred suspension of Methyltriphenylphosphonium bromide (2.0 eq.) in THF (1.0 M) Potassium *tert*-butoxide (3.0 eq.) was added at 0 °C and stirred for 0.5 hours under N_2. A solution of 2-ketobenzoic acid (10 mmol, 1.0 eq.) in THF (3.0 M) was added dropwise. Until the reaction was completed, as monitored by TLC, the resulting mixture was quenched with water, basified with

aqueous NaOH (3.0 M) and washed with diethyl ether. The resulting aqueous phase was acidified with aqueous HCl (2.0 M) until pH 1-2 and extracted twice with ethyl acetate. The combined organic layers were washed with water and brine, dried over Na_2SO_4, filtered and concentrated in vacuo. The resulting crude product was purified by flash column chromatography to afford the desired unsaturated benzoic acid derivative in 40-90% yield.

Procedure III for the synthesis of carboxylic acids 1a-*d*

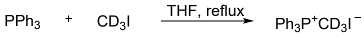

Step: To a solution of triphenylphosphine (5.62 g, 21.4 mmol) in THF (40 mL), CD_3I (1.6 mL, 25.7 mmol) was added dropwise. The solution was refluxed for 1.0 hour and then cooled to room temperature and filtered. The precipitate was washed with xylene and dried under high vacuum to provide the title compound as a white solid in 100% yield.

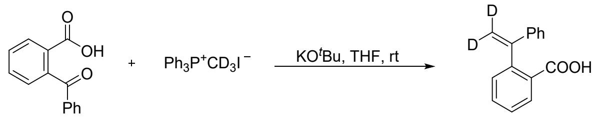

1a-*d*

Step: In an oven dried flask, methyl triphenylphosphonium iodide (1.2 eq.) was suspended in THF (1.6 M). At 0 °C, KO*t*Bu (1.2 eq.) was added and resulted yellow suspension was stirred for another 45 min. After that, a solution of benzophenone (1.0 eq.) in THF (3.0 M) was added dropwise and then warmed gradually to RT and stirred for 16 hours. After reaction was finished, quenched with water, basified with aqueous NaOH (3.0 M) until pH 12-14 and washed with diethyl ether. The resulting aqueous phase was acidified with aqueous HCl (2.0 M) until pH 1-2 and extracted twice with ethyl acetate. The combined organic layers were washed with water and brine, dried over Na_2SO_4, filtered and concentrated in vacuo. The resulting product was purified by flash column chromatography to afford the desired unsaturated benzoic acid derivative in 80% yield.

Procedure I for the synthesis of terminal alkynes (2bh)

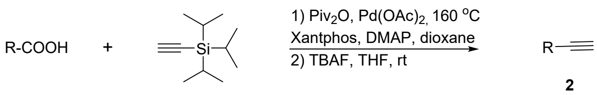

2

Step: An oven-dried flask was charged with carboxylic acid (2.0 mmol), alkyne (4.0 eq.), $Pd(OAc)_2$ (5 mol%), XantPhos (10 mol%), 4-dimethylaminopyridine (1.5 eq.) and trimethylacetic anhydride (1.5 eq.), placed under a positive pressure of N_2, dioxane (0.2 M) was added at room temperature, and then reaction mixture was stirred 10 hours at 160 °C. Until the reaction was completedthe, the reaction

mixture was cooled down to room temperature. The resulting product was purified by flash column chromatography to afford the desired terminal alkynes in 67% yield.

Procedure II for the synthesis of terminal alkynes (2bi-2bk)

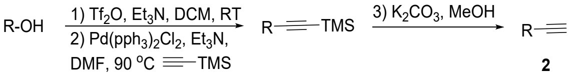

Step: Under N_2, phenols (10.0 mmol) and Et_3N (1.5 eq.) were dissolved in CH_2Cl_2 (20 mL). The mixture was cooled in an ice bath at 0 °C and a solution of Tf_2O (1.5 eq.) in CH_2Cl_2 (20 mL) was added dropwise. The reaction mixture was stirred for another 2.0 h and allowed to warm up to rt during this period. H_2O (50 mL) was added and the product was extracted with CH_2Cl_2 (2×30 mL). The combined organic layers were washed with H_2O (30 mL) and brine (20 mL), dried over Na_2SO_4 and then the solvent was removed in vacuo. The brown residue was purified by flash column chromatography afford the triflate.

The triflate was dissolved in THF (20 mL) and Et_3N (20 mL). $Pd(PPh_3)_2Cl_2$ (1 mol%), CuI (2 mol%) and TMS-acetylene (1.0 eq.) were added to the reaction mixture. The mixture was heated at 90 °C . The reaction was monitored by TLC. The solvents were removed in vacuo and sat. NH_4Cl (30 mL) and EtOAc (50 mL) were added. The layers were partitioned and the aqueous layer was extracted with EtOAc (50 mL). The combined organic layers were washed with H_2O (50 mL) and brine (50 mL), dried over Na_2SO_4 and solvent was removed in vacuo, and purified by silica gel flash column chromatograph.

To a methanol (20 mL) containing silicylene, was added K_2CO_3 (5.0 eq.). The mixture was stirred overnight at room temperature. The product was filtered off, washed with water and dried to give the products in 56-87% yield, and purified by silica gel flash column chromatograph.

Table S2 Failed Examples

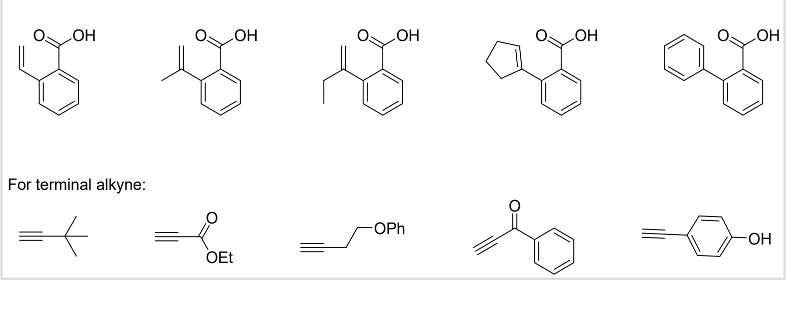

2.3 Control Experiments

Synthesis of intermediates

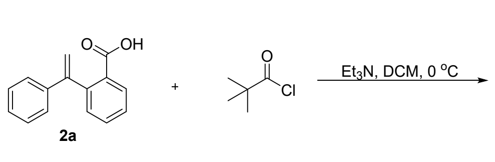

Step: Under N_2, benzoic acid **2a** (0.34 mmol, 1.0 eq.) was dissolved in DCM (20 mL). The mixture was cooled in an ice bath at 0 °C and a solution of triethylamine (1.5 eq.) and pivaloyl chloride (1.0 eq.) in DCM (20 mL) was added dropwise. Stir the mixture at the same temperature for 1.0 h. The TCL plate was used to detect the end of the reaction, and the reaction mixture was washed three times with 5% $NaHCO_3$ solution. The organic phase was dried over anhydrous Na_2SO_4. The solvent was evaporated under reduced pressure to obtain the corresponding anhydride in 97% yield.

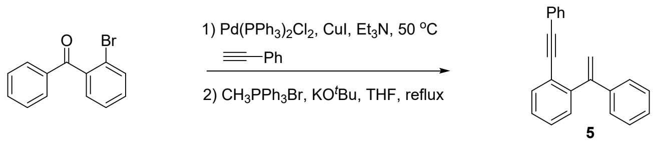

Step: To a solution of the 2-bromobenzophenone (10 mmol), $Pd(PPh_3)_2Cl_2$ (2.0 mol%), and CuI (1.0 mol%) in NEt_3 (0.25 M) was added the appropriate acetylene (1.2 eq.). The resulting mixture was stirred under nitrogen atmosphere at 50 °C overnight. After the reaction was finished, the mixture was filtered by short silica, then the solvent was evaporated under reduced pressure and the residue was purified by flash chromatography on silica gel to afford the desired product.

A solution of methyltriphenylphosphonium bromide (2.0 eq.) and KO*t*Bu (3.0 eq.) in THF (5.0 mL) was stirred at 70 °C under N_2 for 2 h. Afterwards phenyl(2-(phenylethynyl)phenyl)methanone (8.0 mmol) in THF (5.0 mL) was added and the reaction solution was stirred at 70 °C for another 4 h. Upon completion, the reaction was cooled to room temperature and the mixture was filtered. The filtrate was concentrated under reduced pressure and the residue was purified by flash chromatography (eluent: petroleum ether) to afford the products in 88% yield.

2.4 Intermolecular Competition Experiments

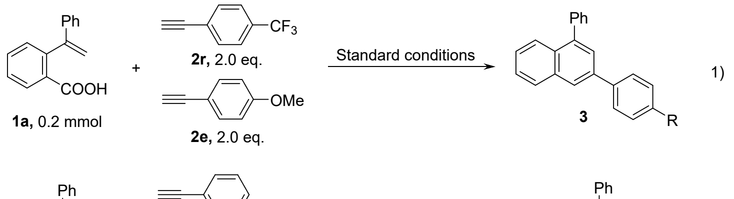

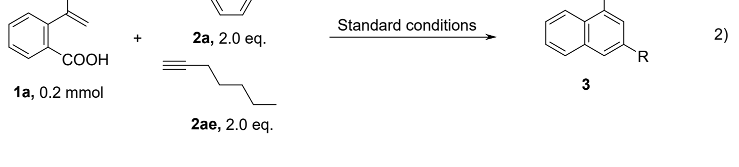

Step: An oven dried 25 mL Schlenk tube was charged with 2-(1-phenylvinyl)benzoic acid **1a** (0.2 mmol), DMAP (1.0 eq.), LiBr (0.5 eq.), $PdCl_2$ (5 mol%) and TFP (10 mol%). Subsequently, Alkynes (2.0 eq.), Piv_2O (1.4 eq.) and cyclohexane (2 mL) were added under N_2, the reaction mixture was allowed to react at 160 °C for 12 h. After completion of the reaction, the reaction mixture was cooled down to RT, and yields were determined by GC using dodecane as an internal standard.

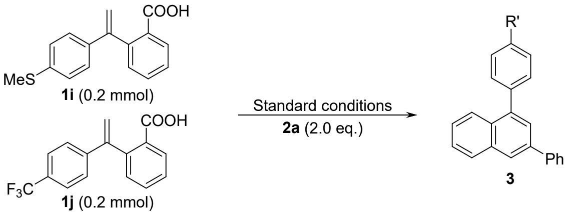

3)

Step: An oven dried 25 mL Schlenk tube was charged with **1i** (0.2 mmol), **1j** (0.2 mmol), DMAP (1.0 eq.), LiBr (0.5 eq.), $PdCl_2$ (5 mol%) and TFP (10 mol%). Subsequently, phenylacetylene (2.0 eq.), Piv_2O (1.4 eq.) and cyclohexane (2 mL) were added under N_2, the reaction mixture was allowed to react at 160 °C for 12 h. After completion of the reaction, the reaction mixture was cooled down to RT, and yields were determined by GC using dodecane as an internal standard.

2.5 Substituent Effect Experiments

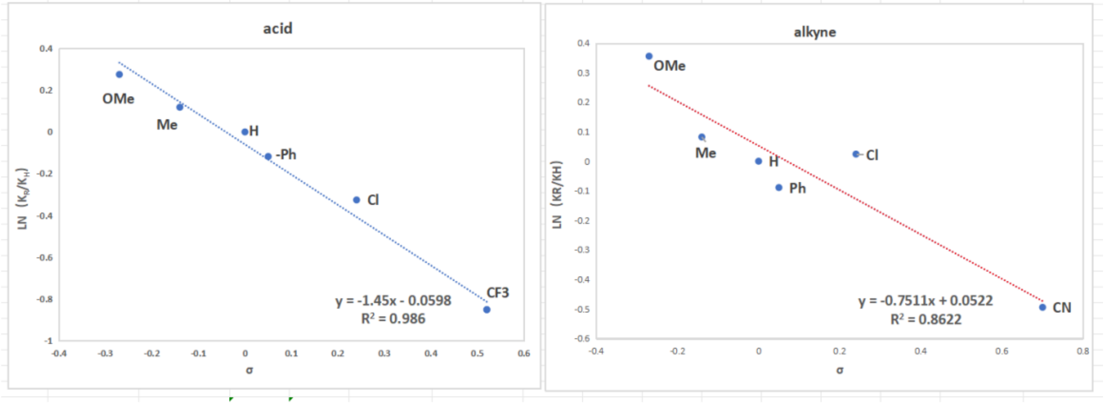

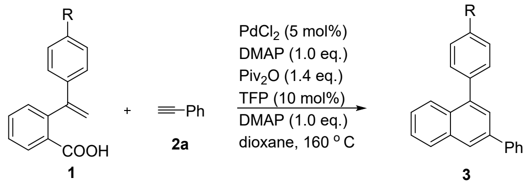

R	OMe	Me	H	Ph	Cl	CF_3
Yield	54%	46%	41%	36%	30%	18%

Step: An oven dried 25 mL Schlenk tube was charged with Carboxylic acid **1a** (0.2 mmol), DMAP (1.0 eq.), LiBr (0.5 eq.), $PdCl_2$ (5 mol%) and TFP (10 mol%). Subsequently, Alkynes (2.0 eq.), Piv_2O

(1.4 eq.) and cyclohexane (2 mL) were added under N_2, the reaction mixture was allowed to react at 160 °C for 45 min. After completion of the reaction, the reaction mixture was cooled down to RT, and yields were determined by GC using dodecane as an internal standard.

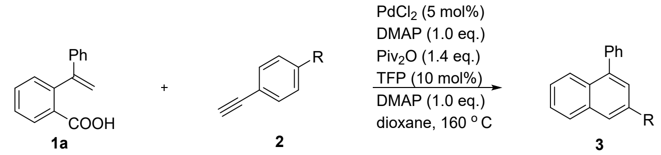

R	OMe	Me	H	Ph	Cl	CN
Yield	58%	45%	41%	37%	42%	25%

Step: An oven dried 25 mL Schlenk tube was charged with 2-(1-phenylvinyl)benzoic acid **1a** (0.2 mmol), DMAP (1.0 eq.), LiBr (0.5 eq.), $PdCl_2$ (5 mol%) and TFP (10 mol%). Subsequently, Alkynes **2** (2.0 eq.), Piv_2O (1.4 eq.) and cyclohexane (2 mL) were added under N_2, the reaction mixture was allowed to react at 160 °C for 45 min. After completion of the reaction, the reaction mixture was cooled down to RT, and yields were determined by GC using dodecane as an internal standard.

2.6 Reaction Progress Kinetic Analysis

To provide further information about the reaction mechanism, kinetic experiments were performed to establish the rate laws of the esterification using the reaction of **1a** and **2a** as a model reaction. All the kinetic data were taken at the time points in the early stage of reaction. With the help of GC tracking on product formation, we evaluated the dependence of the average rate after the initiation period on the **1a**, **2a**, $Pd(OAc)_2$ and DMAP concentrations in each case. (Note: Every indicated times represents one independent experiment).

The rate on the concentration of 1a

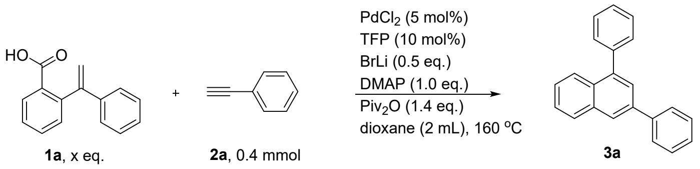

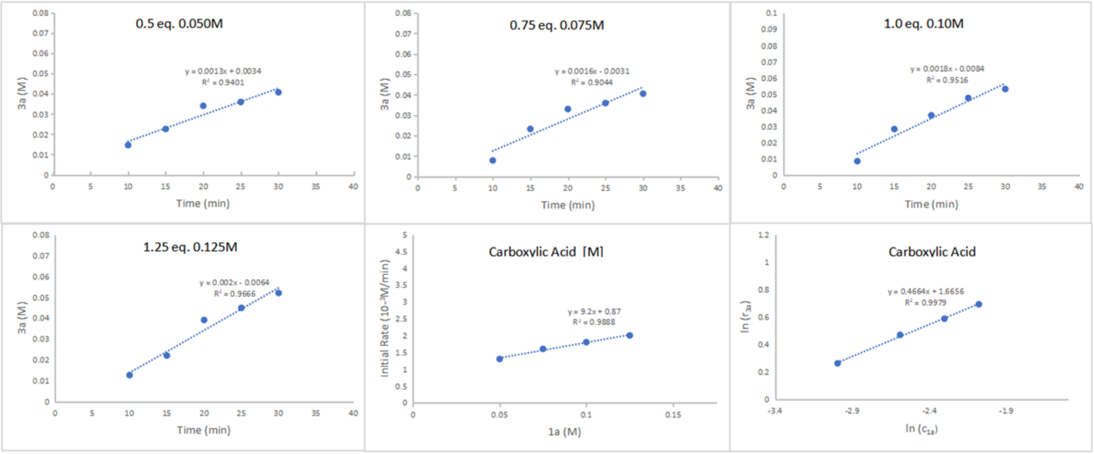

Fig. S1 Plot of the rise of product and the rate on the concentration of **1a** from the reaction of **2a** (0. 4 mmol), Piv_2O (0.28 mmol), DMAP (0.2 mmol), BrLi (0.1 mmol), $PdCl_2$ (5 mol%), TFP (10 mol%) with 0.1 mmol, 0.15 mmol, 0.2 mmol, 0.25 mmol of **1a**.

The rate on the concentration of 2a

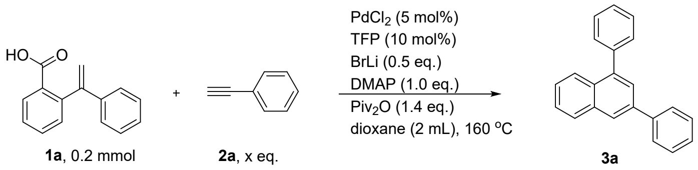

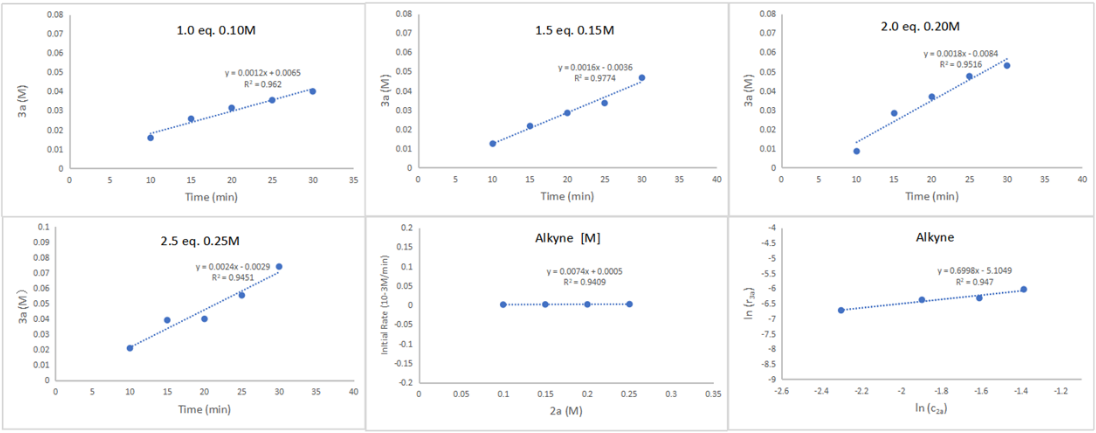

Fig. S2 Plot of the rise of product and the rate on the concentration of **2a** from the reaction of **1a** (0. 2 mmol), Piv_2O (0.28 mmol), DMAP (0.2 mmol), BrLi (0.1 mmol), $PdCl_2$ (5 mol%), TFP (10 mol%) with 0.2 mmol, 0.3 mmol, 0.4 mmol, 0. 5 mmol of **1a**.

The rate on the concentration of Pd cat.

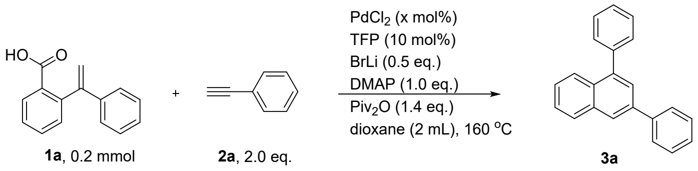

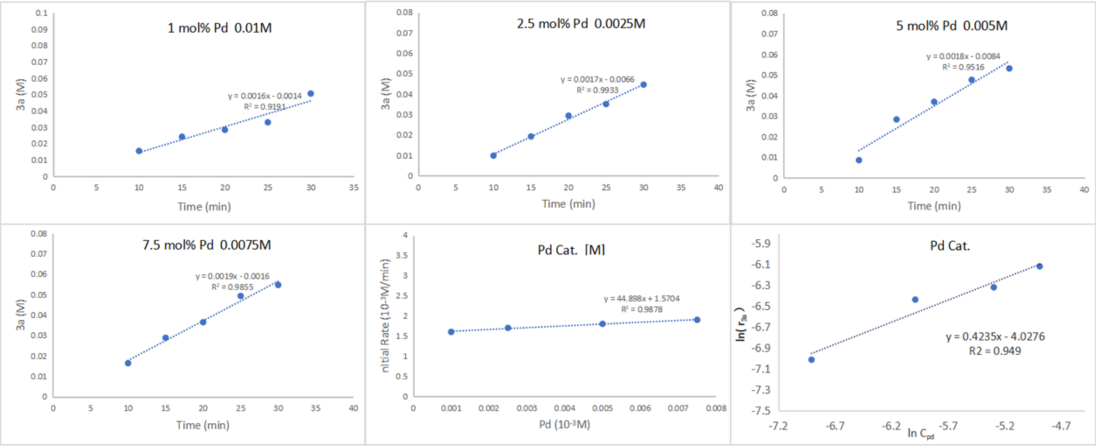

Fig. S3 Plot of the rise of product and the rate on the concentration of $PdCl_2$ from the reaction of **1a** (0. 2 mmol), **2a** (0. 4 mmol), Piv_2O (0.28 mmol), DMAP (0.2 mmol), BrLi (0.1 mmol) with , 1 mol%, 2.5 mol%, 5 mol%, 7.5 mol% of $PdCl_2$.

The rate on the concentration of DMAP

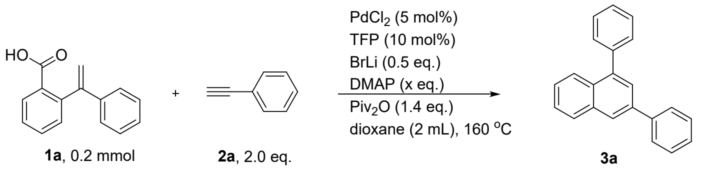

1a, 0.2 mmol **2a**, 2.0 eq. **3a**

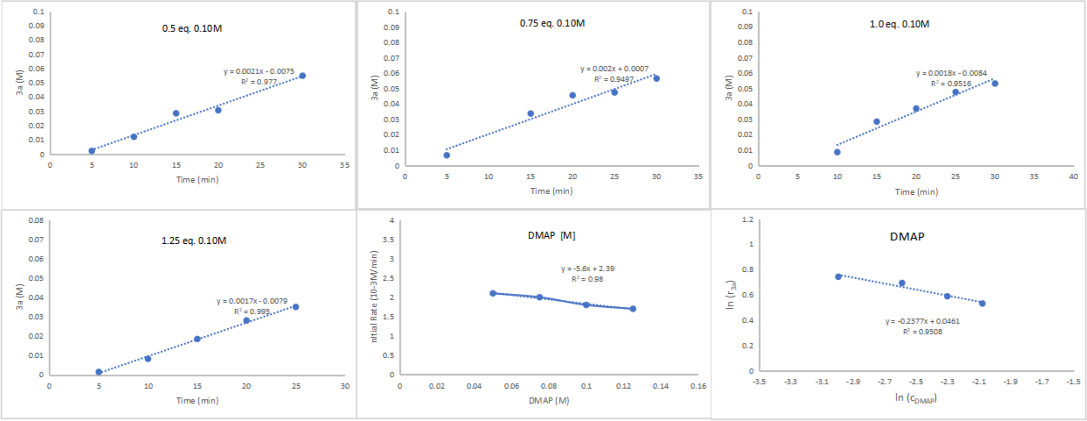

Fig. S4 Plot of the rise of product and the rate on the concentration of DMAP from the reaction of **1a** (0. 2 mmol), **2a** (0. 4 mmol), Piv_2O (0.28 mmol), DMAP (0.2 mmol), BrLi (0.1 mmol), $PdCl_2$ (5 mol%), TFP (10 mol%) with 0.1 mmol, 0.15 mmol, 0.2 mmol, 0. 25 mmol of DMAP.

2.7 Mercury Poisoning Experiments

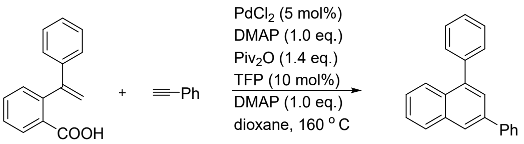

1a **2a** **3a**

Time	Without Hg	Hg/Pd = 10/1	Hg/Pd = 100/1
0.5 h	47%	16%	10%
1 h	58%	23%	11%
12 h	84%	23%	11%

Step: An oven-dried 25 mL Schlenk tube was charged with **1a** (0.2 mmol), $PdCl_2$ (5 mol%), TFP (10 mol%), BrLi (0.5 eq.) and DMAP (1.0 eq.), pumped with nitrogen and finally added Piv_2O (1.4 eq.), **2a** (2.0 eq.) , Hg and dioxane (2 mL). The reaction mixture was reacted at 160 °C.

2.8 General Procedure for Preparation of Product 3

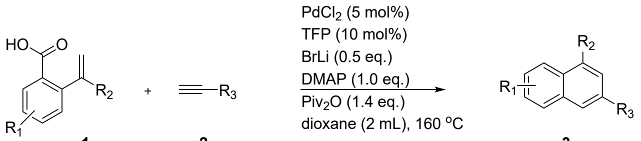

1 2 3

Step: An oven-dried 25 mL Schlenk tube was charged with **1** (0.2 mmol), $PdCl_2$ (5 mol%), TFP (10 mol%), BrLi (0.5 eq.) and DMAP (1.0 eq.), pumped with nitrogen and finally added Piv_2O (1.4 eq.), **2** (2.0 eq.) and dioxane (2 mL). The reaction mixture was reacted at 160 °C for 12 h. The mixture was concentrated under reduced pressure and purified by flash chromatography to afford the products.

2.9 Derivatization Experiments

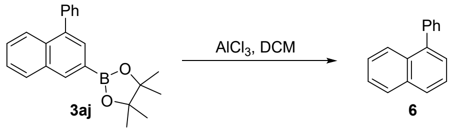

3aj 6

Step: A solution of aryl boronic esters **3ak** (1.0 mmol, 1.0 eq.) and $AlCl_3$ (1.5 eq.) in DCM (20 mL) was stirred at room temperature for 1 h until TLC analysis indicating the complete exhaustion of starting material. Then the reaction mixture was quenched with 1 mol/L HCl aqueous solution, and diluted with DCM. The organic layer was washed with water, 1 mol/L $NaHCO_3$ aqueous solution, and brine, sequentially. After that, the organic layer was dried over Na_2SO_4, and filtered. The filtrate was concentrated under reduced pressure and the residue was purified by flash chromatography (eluent: petroleum ether) to afford the products in 71% yield.

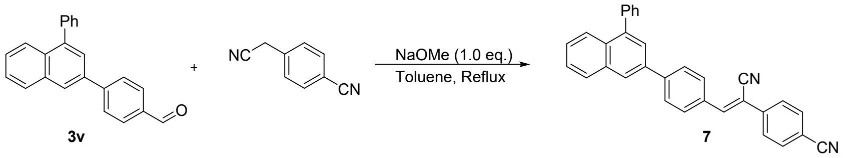

3v 7

Step:To a solution of the arylacetonitrile (1.0 mmol) and **3v** (1.0 mmol) in toluene (4 mL) was added

in one portion 1 mmol $NaOCH_3$. The reaction mixture was stirred at reflux temperature overnight. Acetic ether was added to dissolve the material and this solution was then worked with H_2O (30 mL) three times. After that, the organic layer was dried over Na_2SO_4, and filtered. The filtrate was concentrated under reduced pressure and the residue was purified by flash chromatography (eluent: PE/EA=20/1) to afford the products in 92% yield.

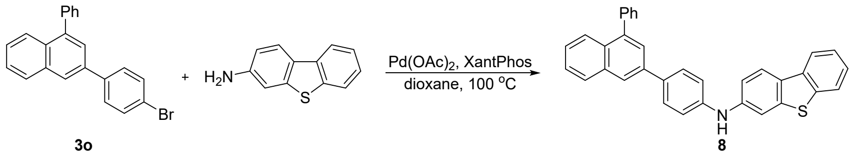

Step: *N*-(4-(4-phenylnaphthalen-2-yl)phenyl)dibenzo[*b,d*]thiophen-3-amine was synthesized by reacting dibenzo[*b,d*]thiophen-3-amine (0.6 mmol) with **3o** (0.5 mmol) in the presence of $Pd(OAc)_2$ (0.05 mmol), Xantphos (0.025 mmol), NaO^tBu (0.5 mmol) at 100 °C for 24 h using dioxane (2 mL) as solvent. The mixture was concentrated under reduced pressure and purified by flash chromatography (eluent: PE/EA=20/1) to afford the products in 67% yield.

2.10 Measurement of Intermediate by MALDI-TOF MS

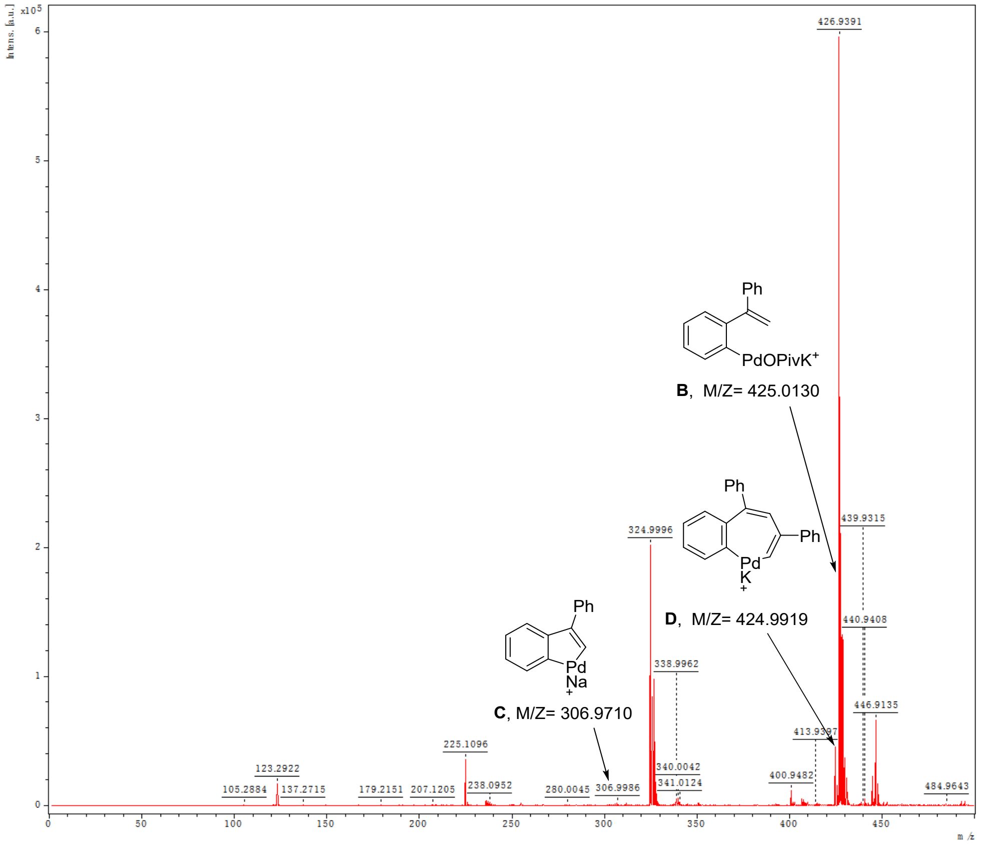

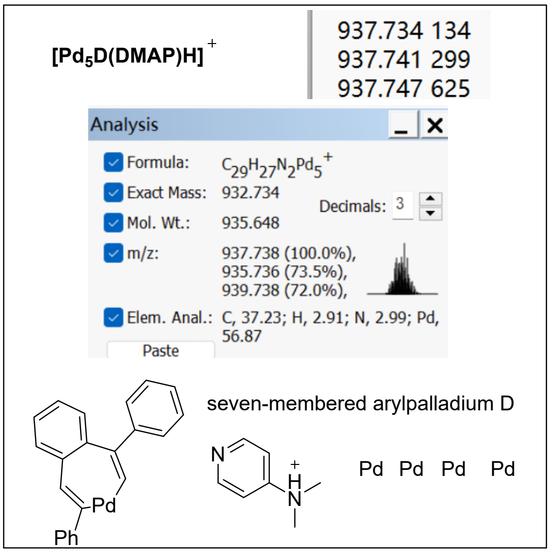
[Pd5D(DMAP)H]+
937.734 134
937.741 299
937.747 625
Analysis
Formula: C29H27N2Pd5+
Exact Mass: 932.734
Decimals: 3
Mol. Wt.: 935.648
m/z: 937.738 (100.0%), 935.736 (73.5%), 939.738 (72.0%),
Elem. Anal.: C, 37.23; H, 2.91; N, 2.99; Pd, 56.87
Paste
seven-membered arylpalladium D
Pd
Ph
N
H
N
+
Pd Pd Pd Pd

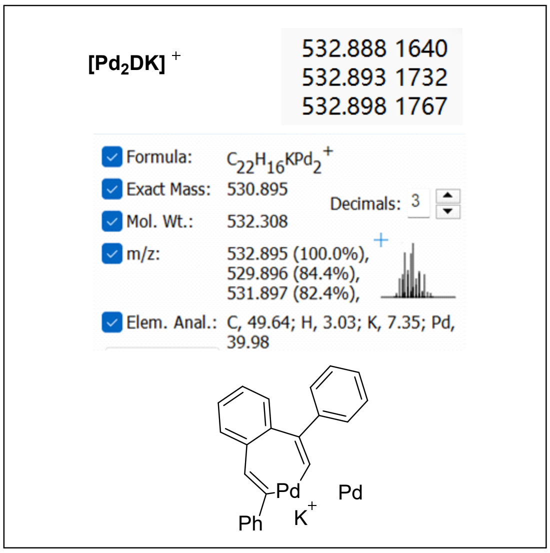
[Pd2DK]+
532.888 1640
532.893 1732
532.898 1767
Formula: C22H16KPd2+
Exact Mass: 530.895
Decimals: 3
Mol. Wt.: 532.308
m/z: 532.895 (100.0%), 529.896 (84.4%), 531.897 (82.4%),
Elem. Anal.: C, 49.64; H, 3.03; K, 7.35; Pd, 39.98
Pd
Ph
K+
Pd

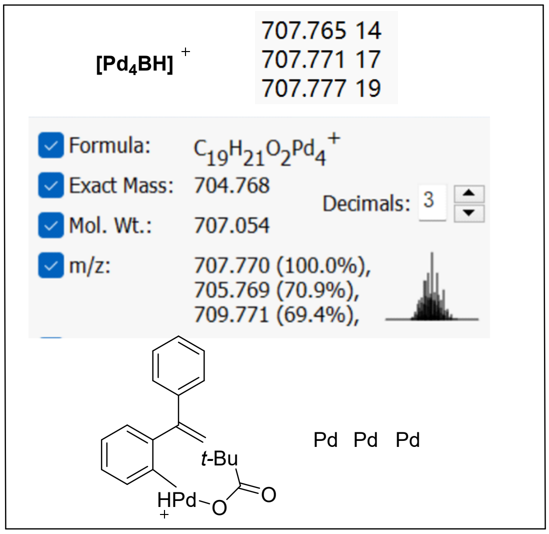
[Pd4BH]+
707.765 14
707.771 17
707.777 19
Formula: C19H21O2Pd4+
Exact Mass: 704.768
Decimals: 3
Mol. Wt.: 707.054
m/z: 707.770 (100.0%), 705.769 (70.9%), 709.771 (69.4%),
t-Bu
HPd
O
O
+
Pd Pd Pd

3 X-ray Crystallographic Data

Check CIF/PLATON report

Compounds **3bi** was collected at 298 K on a Rigaku Oxford Diffraction Supernova Dual Source, Cu at Zero equipped with an AtlasS2 CCD using Cu Kα radiation. Data reduction was carried out with the diffractometer's software.s1 The structures were solved by direct methods using Olex2 softwares2 and the non-hydrogen atoms were located from the trial structure and then refined anisotropically with SHELXL-2014s3 using a full-matrix least squares procedure based on F2. The weighted R factor, wR and S values were obtained based on F2.The hydrogen atom positions were fixed geometrically at the calculated distances and allowed to ride on their parent atoms. Crystallographic data for the structure reported in this paper have been deposited at the Cambridge Crystallographic Data Center and allocated with the deposition numbers: CCDC 2311101.

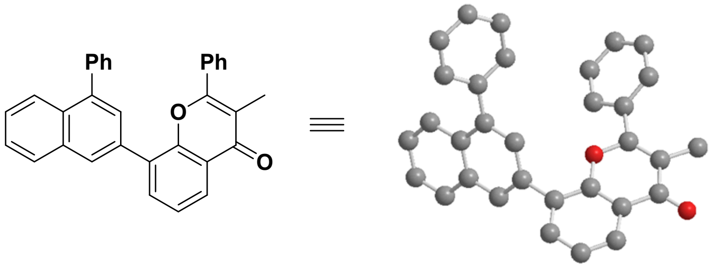

Fig. S5 ORTEP drawing of **3bi**

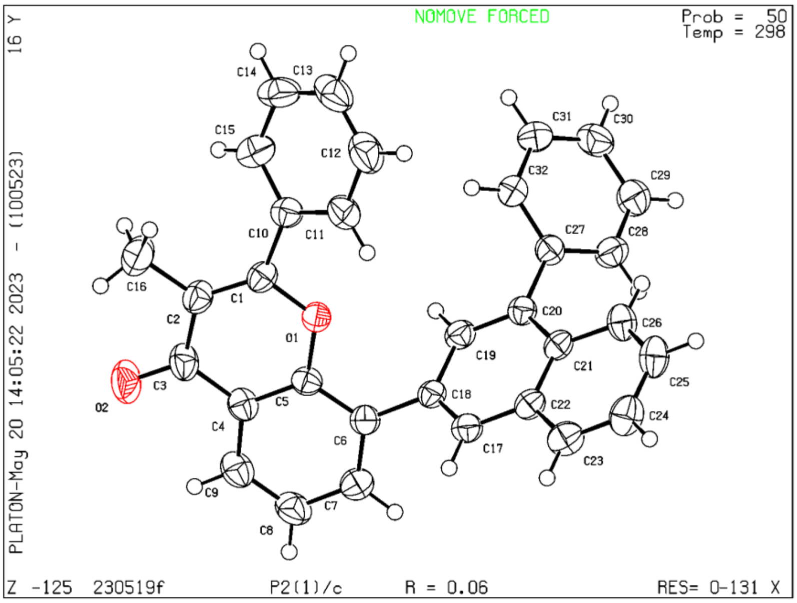

The ellipsoid contour % probability levels in the caption for the image of the structure is 50%.

Table S3 Crystal data and structure refinement for **3bi**.

Empirical formula	$C_{32}H_{22}O_2$
Formula weight	438.50
Temperature	298(2) K
Wavelength	0.71073 A
Crystal system, space group	Monoclinic, P2(1)/c
	a = 10.2726(11) A alpha = 90 deg.
Unit cell dimensions	b = 23.866(2)A beta = 118.405(6) deg.
	c = 10.2726(11) A gamma = 90 deg.
Volume	2215.3(4) A^3
Z, Calculated density	4, 1.315 Mg/m^3
Absorption coefficient	0.081 mm^-1
F(000)	920
Crystal size	0.43 x 0.41 x 0.25 mm
Theta range for data collection	2.41 to 25.02 deg.
Limiting indices	-12<=h<=11, -28<=k<=16, -12<=l<=12
Reflections collected / uniqu	10651 / 3891 [R(int) = 0.0553]
Completeness to theta = 25.02	99.4 %
Absorption correction	Semi-empirical from equivalents
Max. and min. transmission	0.9801 and 0.9661
Refinement method	Full-matrix least-squares on F^2
Data / restraints / parameters	3891 / 0 / 308
Goodness-of-fit on F^2	1.035
Final R indices [I>2sigma(I)]	R1 = 0.0564, wR2 = 0.1349
R indices (all data)	R1 = 0.0977, wR2 = 0.1516
Largest diff. peak and hole	0.173 and -0.214 e.A^-3

Table S4 Atomic coordinates (x 10^4) and equivalent isotropic displacement parameters (A^2 x 10^3) for **3bi** U_{eq} is defined as one third of the trace of the orthogonalized U_{ij} tensor.

**Atom**	**x**	**y**	**z**	**U(eq.)**
O(1)	2386(2)	598(1)	3335(2)	39(1)
O(2)	2067(2)	-769(1)	925(3)	64(1)
C(1)	3493(3)	461(1)	3032(3)	37(1)
C(2)	3415(3)	22(1)	2193(3)	41(1)
C(3)	2163(3)	-351(1)	1651(3)	45(1)
C(4)	983(3)	-189(1)	1969(3)	38(1)
C(5)	1123(3)	288(1)	2768(3)	34(1)
C(6)	14(3)	493(1)	3030(3)	36(1)
C(7)	-1258(3)	185(1)	2489(3)	46(1)
C(8)	-1416(3)	-311(1)	1727(3)	51(1)
C(9)	-317(3)	-491(1)	1459(3)	46(1)
C(10)	4682(3)	871(1)	3733(3)	38(1)
C(11)	4337(3)	1424(1)	3809(3)	49(1)
C(12)	5432(4)	1817(1)	4409(4)	62(1)
C(13)	6867(4)	1673(2)	4956(4)	66(1)
C(14)	7226(3)	1126(2)	4949(3)	61(1)
C(15)	6152(3)	725(1)	4345(3)	51(1)
C(16)	4565(3)	-101(1)	1734(4)	65(1)
C(17)	-784(3)	1458(1)	3087(3)	37(1)
C(18)	179(3)	1036(1)	3787(3)	33(1)
C(19)	1318(3)	1125(1)	5228(3)	37(1)
C(20)	1473(3)	1616(1)	5958(3)	33(1)
C(21)	493(3)	2063(1)	5221(3)	34(1)
C(22)	-641(3)	1976(1)	3770(3)	36(1)
C(23)	-1618(3)	2421(1)	3026(3)	46(1)
C(24)	-1478(3)	2924(1)	3684(3)	51(1)
C(25)	-353(3)	3007(1)	5112(3)	48(1)
C(26)	604(3)	2593(1)	5870(3)	42(1)

C(27)	2653(3)	1662(1)	7510(3)	36(1)
C(28)	2328(3)	1791(1)	8632(3)	45(1)
C(29)	3406(3)	1779(1)	10074(3)	53(1)
C(30)	4825(3)	1643(1)	10434(3)	54(1)
C(31)	5164(3)	1525(1)	9338(3)	53(1)
C(32)	4090(3)	1538(1)	7885(3)	44(1)

Table S5 Bond lengths [A] and angles [deg] for **3bi**.

Atom	Bond lengths	Atom	Bond angles
O(1)-C(1)	1.354(3)	C(1)-O(1)-C(5)	120.3(2)
O(1)-C(5)	1.360(3)	C(2)-C(1)-O(1)	122.6(2)
O(2)-C(3)	1.221(3)	C(2)-C(1)-C(10)	128.7(2)
C(1)-C(2)	1.334(4)	O(1)-C(1)-C(10)	108.7(2)
C(1)-C(10)	1.461(4)	C(1)-C(2)-C(3)	120.2(2)
C(2)-C(3)	1.440(4)	C(1)-C(2)-C(16)	123.0(3)
C(2)-C(16)	1.495(4)	C(3)-C(2)-C(16)	116.8(2)
C(3)-C(4)	1.451(4)	O(2)-C(3)-C(2)	122.7(3)
C(4)-C(5)	1.370(4)	O(2)-C(3)-C(4)	121.5(3)
C(4)-C(9)	1.382(3)	C(2)-C(3)-C(4)	115.8(2)
C(5)-C(6)	1.378(3)	C(5)-C(4)-C(9)	117.9(2)
C(6)-C(7)	1.366(3)	C(5)-C(4)-C(3)	119.7(2)
C(6)-C(18)	1.479(4)	C(9)-C(4)-C(3)	122.4(2)
C(7)-C(8)	1.385(4)	O(1)-C(5)-C(4)	121.0(2)
C(7)-H(7)	0.9300	O(1)-C(5)-C(6)	115.7(2)
C(8)-C(9)	1.353(4)	C(4)-C(5)-C(6)	123.3(2)
C(8)-H(8)	0.9300	C(7)-C(6)-C(5)	116.8(2)
C(9)-H(9)	0.9300	C(7)-C(6)-C(18)	122.2(2)
C(10)-C(15)	1.376(4)	C(5)-C(6)-C(18)	120.9(2)
C(10)-C(11)	1.378(4)	C(6)-C(7)-C(8)	121.4(3)

C(11)-C(12)	1.366(4)	C(6)-C(7)-H(7)	119.3
C(11)-H(11)	0.9300	C(8)-C(7)-H(7)	119.3
C(12)-C(13)	1.349(5)	C(9)-C(8)-C(7)	120.1(3)
C(12)-H(12)	0.9300	C(9)-C(8)-H(8)	120.0
C(13)-C(14)	1.359(5)	C(7)-C(8)-H(8)	120.0
C(13)-H(13)	0.9300	C(8)-C(9)-C(4)	120.4(3)
C(14)-C(15)	1.366(4)	C(8)-C(9)-H(9)	119.8
C(14)-H(14)	0.9300	C(4)-C(9)-H(9)	119.8
C(15)-H(15)	0.9300	C(15)-C(10)-C(11)	118.2(3)
C(16)-H(16A)	0.9600	C(15)-C(10)-C(1)	122.2(3)
C(16)-H(16B)	0.9600	C(11)-C(10)-C(1)	119.6(2)
C(16)-H(16C)	0.9600	C(12)-C(11)-C(10)	120.3(3)
C(17)-C(18)	1.354(3)	C(12)-C(11)-H(11)	119.8
C(17)-C(22)	1.394(4)	C(10)-C(11)-H(11)	119.8
C(17)-H(17)	0.9300	C(13)-C(12)-C(11)	121.0(3)
C(18)-C(19)	1.399(3)	C(13)-C(12)-H(12)	119.5
C(19)-C(20)	1.361(3)	C(12)-C(13)-C(14)	119.2(3)
C(19)-H(19)	0.9300	C(12)-C(13)-H(13)	120.4
C(20)-C(21)	1.413(3)	C(14)-C(13)-H(13)	120.4
C(20)-C(27)	1.477(3)	C(13)-C(14)-C(15)	120.9(3)
C(21)-C(22)	1.403(3)	C(13)-C(14)-H(14)	119.6
C(21)-C(26)	1.409(4)	C(15)-C(14)-H(14)	119.6
C(22)-C(23)	1.410(3)	C(14)-C(15)-C(10)	120.2(3)
C(23)-C(24)	1.351(4)	C(14)-C(15)-H(15)	119.9
C(23)-H(23)	0.9300	C(10)-C(15)-H(15)	119.9
C(24)-C(25)	1.384(4)	C(2)-C(16)-H(16A)	109.5
C(24)-H(24)	0.9300	C(2)-C(16)-H(16B)	109.5
C(25)-C(26)	1.349(4)	H(16A)-C(16)-H(16B)	109.5
C(25)-H(25)	0.9300	C(2)-C(16)-H(16C)	109.5

C(26)-H(26)	0.9300	H(16A)-C(16)-H(16C)	109.5
C(27)-C(32)	1.369(3)	H(16B)-C(16)-H(16C)	109.5
C(27)-C(28)	1.379(4)	C(18)-C(17)-C(22)	121.1(2)
C(28)-C(29)	1.364(4)	C(18)-C(17)-H(17)	119.4
C(28)-H(28)	0.9300	C(22)-C(17)-H(17)	119.4
C(29)-C(30)	1.362(4)	C(17)-C(18)-C(19)	118.7(2)
C(29)-H(29)	0.9300	C(17)-C(18)-C(6)	120.0(2)
C(30)-C(31)	1.356(4)	C(19)-C(18)-C(6)	121.2(2)
C(30)-H(30)	0.9300	C(20)-C(19)-C(18)	122.3(2)
C(31)-C(32)	1.371(4)	C(20)-C(19)-H(19)	118.9
C(31)-H(31)	0.9300	C(18)-C(19)-H(19)	118.9
C(32)-H(32)	0.9300	C(19)-C(20)-C(21)	119.2(2)
		C(19)-C(20)-C(27)	118.6(2)
		C(21)-C(20)-C(27)	122.2(2)
		C(22)-C(21)-C(26)	118.4(2)
		C(22)-C(21)-C(20)	118.5(2)
		C(26)-C(21)-C(20)	123.1(2)
		C(17)-C(22)-C(21)	120.1(2)
		C(17)-C(22)-C(23)	121.1(2)
		C(21)-C(22)-C(23)	118.8(2)
		C(24)-C(23)-C(22)	121.2(3)
		C(24)-C(23)-H(23)	119.4
		C(22)-C(23)-H(23)	119.4
		C(23)-C(24)-C(25)	119.6(3)
		C(23)-C(24)-H(24)	120.2
		C(25)-C(24)-H(24)	120.2
		C(26)-C(25)-C(24)	121.2(3)
		C(26)-C(25)-H(25)	119.4
		C(24)-C(25)-H(25)	119.4

C(25)-C(26)-C(21)	120.8(3)
C(25)-C(26)-H(26)	119.6
C(21)-C(26)-H(26)	119.6
C(32)-C(27)-C(28)	118.0(2)
C(32)-C(27)-C(20)	120.7(2)
C(28)-C(27)-C(20)	121.2(2)
C(29)-C(28)-C(27)	120.6(3)
C(29)-C(28)-H(28)	119.7
C(27)-C(28)-H(28)	119.7
C(30)-C(29)-C(28)	120.7(3)
C(30)-C(29)-H(29)	119.6
C(28)-C(29)-H(29)	119.6
C(31)-C(30)-C(29)	119.2(3)
C(31)-C(30)-H(30)	120.4
C(29)-C(30)-H(30)	120.4
C(30)-C(31)-C(32)	120.5(3)
C(30)-C(31)-H(31)	119.8
C(32)-C(31)-H(31)	119.8
C(27)-C(32)-C(31)	120.9(3)
C(27)-C(32)-H(32)	119.6
C(31)-C(32)-H(32)	119.6

Table S6 Anisotropic displacement parameters (A^2 x 10^3) for **3bi**. The anisotropic displacement factor exponent takes the form: -2 pi^2 [h^2 a*^2 U11 + ... + 2 h k a* b* U12]

Atom	U11	U22	U33	U23	U13	U12
O(1)	36(1)	37(1)	49(1)	-10(1)	24(1)	-6(1)
O(2)	78(2)	39(1)	91(2)	-23(1)	54(1)	-9(1)
C(1)	37(1)	37(2)	42(2)	4(1)	24(1)	2(1)
C(2)	42(2)	34(2)	54(2)	0(1)	28(1)	1(1)
C(3)	57(2)	31(2)	55(2)	-6(1)	32(2)	-2(1)

C(4)	46(2)	31(2)	40(2)	0(1)	22(1)	-4(1)
C(5)	34(1)	34(2)	35(2)	1(1)	17(1)	-4(1)
C(6)	35(1)	39(2)	34(2)	0(1)	17(1)	-1(1)
C(7)	41(2)	52(2)	51(2)	-5(2)	26(1)	-7(1)
C(8)	50(2)	49(2)	53(2)	-11(2)	25(2)	-18(2)
C(9)	57(2)	35(2)	49(2)	-5(1)	27(2)	-10(1)
C(10)	37(2)	44(2)	35(2)	1(1)	18(1)	-3(1)
C(11)	51(2)	42(2)	56(2)	-12(1)	26(2)	-9(1)
C(12)	77(2)	53(2)	70(2)	-20(2)	45(2)	-20(2)
C(13)	70(2)	89(3)	54(2)	-29(2)	41(2)	-40(2)
C(14)	38(2)	102(3)	44(2)	-3(2)	20(2)	-14(2)
C(15)	39(2)	63(2)	52(2)	8(2)	22(1)	2(2)
C(16)	59(2)	59(2)	93(3)	-16(2)	49(2)	0(2)
C(17)	33(1)	43(2)	34(2)	1(1)	16(1)	-1(1)
C(18)	30(1)	39(2)	32(2)	-2(1)	18(1)	-3(1)
C(19)	32(1)	39(2)	38(2)	2(1)	15(1)	5(1)
C(20)	33(1)	37(2)	32(2)	1(1)	17(1)	-1(1)
C(21)	33(1)	39(2)	36(2)	4(1)	23(1)	1(1)
C(22)	35(1)	38(2)	38(2)	5(1)	21(1)	1(1)
C(23)	44(2)	48(2)	43(2)	11(1)	18(1)	7(1)
C(24)	60(2)	42(2)	54(2)	12(2)	29(2)	16(2)
C(25)	68(2)	34(2)	55(2)	3(1)	40(2)	7(2)
C(26)	55(2)	37(2)	40(2)	0(1)	28(2)	1(1)
C(27)	36(1)	33(2)	35(2)	1(1)	15(1)	3(1)
C(28)	36(2)	58(2)	40(2)	0(1)	17(1)	5(1)
C(29)	53(2)	70(2)	37(2)	-5(2)	22(2)	3(2)
C(30)	46(2)	60(2)	40(2)	-6(2)	8(1)	1(2)
C(31)	35(2)	65(2)	48(2)	-7(2)	10(2)	7(1)

C(32)	37(2)	50(2)	43(2)	-7(1)	19(1)	2(1)

Table S7 Hydrogen coordinates (x 10^4) and isotropic displacement parameters (A^2 x 10^3) for **3bi**.

**Atom**	**x**	**y**	**z**	**U(eq.)**
H(7)	-2036	312	2635	55
H(8)	-2279	-521	1400	61
H(9)	-437	-819	928	56
H(11)	3354	1530	3449	59
H(12)	5184	2190	4441	75
H(13)	7603	1946	5333	80
H(14)	8216	1022	5361	74
H(15)	6414	352	4347	62
H(16A)	5000	244	1648	98
H(16B)	4115	-290	796	98
H(16C)	5318	-336	2462	98
H(17)	-1553	1402	2134	44
H(19)	1992	838	5704	44
H(23)	-2371	2367	2063	56
H(24)	-2135	3212	3181	62
H(25)	-254	3355	5558	58
H(26)	1346	2658	6832	50
H(28)	1365	1888	8404	54
H(29)	3169	1864	10820	64
H(30)	5554	1630	11419	64
H(31)	6132	1434	9574	64
H(32)	4341	1462	7145	52

Table S8 Torsion angles [deg] for **3bi**.

Atom	Torsion angles
C(5)-O(1)-C(1)-C(2)	-0.7(4)
C(5)-O(1)-C(1)-C(10)	178.0(2)
O(1)-C(1)-C(2)-C(3)	-4.2(4)
C(10)-C(1)-C(2)-C(3)	177.4(3)
O(1)-C(1)-C(2)-C(16)	174.0(3)
C(10)-C(1)-C(2)-C(16)	-4.4(5)
C(1)-C(2)-C(3)-O(2)	-177.3(3)
C(16)-C(2)-C(3)-O(2)	4.4(4)
C(1)-C(2)-C(3)-C(4)	5.0(4)
C(16)-C(2)-C(3)-C(4)	-173.3(3)
O(2)-C(3)-C(4)-C(5)	-179.0(3)
C(2)-C(3)-C(4)-C(5)	-1.2(4)
O(2)-C(3)-C(4)-C(9)	-0.9(4)
C(2)-C(3)-C(4)-C(9)	176.9(3)
C(1)-O(1)-C(5)-C(4)	4.6(4)
C(1)-O(1)-C(5)-C(6)	-174.3(2)
C(9)-C(4)-C(5)-O(1)	178.3(2)
C(3)-C(4)-C(5)-O(1)	-3.5(4)
C(9)-C(4)-C(5)-C(6)	-2.8(4)
C(3)-C(4)-C(5)-C(6)	175.3(2)
O(1)-C(5)-C(6)-C(7)	-179.1(2)
C(4)-C(5)-C(6)-C(7)	2.0(4)
O(1)-C(5)-C(6)-C(18)	4.3(4)
C(4)-C(5)-C(6)-C(18)	-174.6(2)
C(5)-C(6)-C(7)-C(8)	0.6(4)
C(18)-C(6)-C(7)-C(8)	177.2(3)
C(6)-C(7)-C(8)-C(9)	-2.4(4)
C(7)-C(8)-C(9)-C(4)	1.5(4)

C(5)-C(4)-C(9)-C(8)	1.0(4)
C(3)-C(4)-C(9)-C(8)	-177.1(3)
C(2)-C(1)-C(10)-C(15)	-37.9(4)
O(1)-C(1)-C(10)-C(15)	143.5(3)
C(2)-C(1)-C(10)-C(11)	142.9(3)
O(1)-C(1)-C(10)-C(11)	-35.7(3)
C(15)-C(10)-C(11)-C(12)	3.4(4)
C(1)-C(10)-C(11)-C(12)	-177.5(3)
C(10)-C(11)-C(12)-C(13)	-0.9(5)
C(11)-C(12)-C(13)-C(14)	-2.1(5)
C(12)-C(13)-C(14)-C(15)	2.6(5)
C(13)-C(14)-C(15)-C(10)	-0.1(5)
C(11)-C(10)-C(15)-C(14)	-2.9(4)
C(1)-C(10)-C(15)-C(14)	178.0(3)
C(22)-C(17)-C(18)-C(19)	0.9(4)
C(22)-C(17)-C(18)-C(6)	-179.2(2)
C(7)-C(6)-C(18)-C(17)	-57.0(4)
C(5)-C(6)-C(18)-C(17)	119.4(3)
C(7)-C(6)-C(18)-C(19)	122.8(3)
C(5)-C(6)-C(18)-C(19)	-60.7(3)
C(17)-C(18)-C(19)-C(20)	1.3(4)
C(6)-C(18)-C(19)-C(20)	-178.6(2)
C(18)-C(19)-C(20)-C(21)	-2.8(4)
C(18)-C(19)-C(20)-C(27)	176.4(2)
C(19)-C(20)-C(21)-C(22)	2.1(3)
C(27)-C(20)-C(21)-C(22)	-177.0(2)
C(19)-C(20)-C(21)-C(26)	-177.6(2)
C(27)-C(20)-C(21)-C(26)	3.3(4)
C(18)-C(17)-C(22)-C(21)	-1.5(4)

C(18)-C(17)-C(22)-C(23)	178.1(2)
C(26)-C(21)-C(22)-C(17)	179.7(2)
C(20)-C(21)-C(22)-C(17)	0.0(3)
C(26)-C(21)-C(22)-C(23)	0.0(3)
C(20)-C(21)-C(22)-C(23)	-179.7(2)
C(17)-C(22)-C(23)-C(24)	-179.8(3)
C(21)-C(22)-C(23)-C(24)	-0.1(4)
C(22)-C(23)-C(24)-C(25)	0.4(4)
C(23)-C(24)-C(25)-C(26)	-0.7(4)
C(24)-C(25)-C(26)-C(21)	0.6(4)
C(22)-C(21)-C(26)-C(25)	-0.3(4)
C(20)-C(21)-C(26)-C(25)	179.4(2)
C(19)-C(20)-C(27)-C(32)	53.7(3)
C(21)-C(20)-C(27)-C(32)	-127.2(3)
C(19)-C(20)-C(27)-C(28)	-121.7(3)
C(21)-C(20)-C(27)-C(28)	57.4(4)
C(32)-C(27)-C(28)-C(29)	-1.9(4)
C(20)-C(27)-C(28)-C(29)	173.6(3)
C(27)-C(28)-C(29)-C(30)	0.5(5)
C(28)-C(29)-C(30)-C(31)	0.8(5)
C(29)-C(30)-C(31)-C(32)	-0.5(5)
C(28)-C(27)-C(32)-C(31)	2.1(4)
C(20)-C(27)-C(32)-C(31)	-173.4(3)
C(30)-C(31)-C(32)-C(27)	-1.0(5)

```
The following ALERTS were generated. Each ALERT has the format
       test-name_ALERT_alert-type_alert-level.
Click on the hyperlinks for more details of the test.
```

Alert level C

```
PLAT331_ALERT_2_C Small Aver Phenyl C-C Dist C10      --C15      .      1.37 Ang.
PLAT331_ALERT_2_C Small Aver Phenyl C-C Dist C27      --C32      .      1.37 Ang.
PLAT334_ALERT_2_C Small <C-C> Benzene Dist.   C4       -C9       .      1.37 Ang.
PLAT340_ALERT_3_C Low Bond Precision on  C-C Bonds ..............   0.00436 Ang.
```

Alert level G

```
PLAT005_ALERT_5_G No Embedded Refinement Details Found  in the CIF     Please Do !
PLAT066_ALERT_1_G Predicted and Reported Tmin&Tmax Range Identical          ? Check
PLAT093_ALERT_1_G No s.u.'s on H-positions, Refinement Reported as      mixed Check
PLAT899_ALERT_4_G SHELXL-97  is Deprecated and Succeeded by SHELXL     2019/3 Note
```

```
   0 ALERT level A = Most likely a serious problem - resolve or explain
   0 ALERT level B = A potentially serious problem, consider carefully
   4 ALERT level C = Check. Ensure it is not caused by an omission or oversight
   4 ALERT level G = General information/check it is not something unexpected

   2 ALERT type 1 CIF construction/syntax error, inconsistent or missing data
   3 ALERT type 2 Indicator that the structure model may be wrong or deficient
   1 ALERT type 3 Indicator that the structure quality may be low
   1 ALERT type 4 Improvement, methodology, query or suggestion
   1 ALERT type 5 Informative message, check
```

It is advisable to attempt to resolve as many as possible of the alerts in all categories. Often the minor alerts point to easily fixed oversights, errors and omissions in your CIF or refinement strategy, so attention to these fine details can be worthwhile. In order to resolve some of the more serious problems it may be necessary to carry out additional measurements or structure refinements. However, the purpose of your study may justify the reported deviations and the more serious of these should normally be commented upon in the discussion or experimental section of a paper or in the "special_details" fields of the CIF. checkCIF was carefully designed to identify outliers and unusual parameters, but every test has its limitations and alerts that are not important in a particular case may appear. Conversely, the absence of alerts does not guarantee there are no aspects of the results needing attention. It is up to the individual to critically assess their own results and, if necessary, seek expert advice.

Publication of your CIF in IUCr journals

A basic structural check has been run on your CIF. These basic checks will be run on all CIFs submitted for publication in IUCr journals (*Acta Crystallographica*, *Journal of Applied Crystallography*, *Journal of Synchrotron Radiation*); however, if you intend to submit to *Acta Crystallographica Section C* or *E* or *IUCrData*, you should make sure that full publication checks are run on the final version of your CIF prior to submission.

Publication of your CIF in other journals

Please refer to the *Notes for Authors* of the relevant journal for any special instructions relating to CIF submission.

PLATON version of 10/05/2023; check.def file version of 10/05/2023

Preparation of single crystal:

3bi (50 mg) was dissolved in ethyl acetate (5.0 mL) in a 25 mL round bottom, and small holes were poked in the membrane after sealing with a sealing membrane. The solvent evaporated at room temperature and single crystals precipitated after a week.

4. Characterization Data for the Products

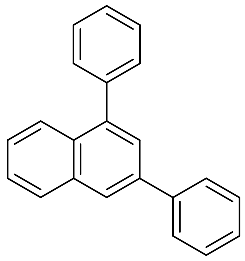

1,3-diphenylnaphthalene (3a): The representative general procedure mentioned above was followed. An oven dried 25 mL Schlenk tube was charged with carboxylic acid **1a** (0.2 mmol), DMAP (0.2 mmol, 1.0 eq.), LiBr (0.1 mmol, 0.5 eq.), $PdCl_2$ (5 mol%) and TFP (10 mol%). Subsequently, phenylacetylene **2a** (0.4 mmol, 2.0 eq.), Piv_2O (1.4 eq.) and dioxane (2 mL) was added under N_2. The reaction mixture was allowed to react at 160 °C for 12 h. After completion of the reaction, the reaction mixture was then cooled to room temperature, and then the reaction mixture was transferred to a round-bottomed bottle and concentrated under reduced pressure. The crude residue was purified by flash chromatography on silica gel (PE) to give the desired **3a** as yellow oil in 77% yield (43.1 mg). ^1H NMR (400 MHz, $CDCl_3$) δ 8.04 (s, 1H), 7.94 – 7.88 (m, 2H), 7.75 – 7.69 (m, 3H), 7.56 – 7.52 (m, 2H), 7.49 – 7.48 (m, 2H), 7.46 – 7.50 (m, 2H), 7.50 – 7.40 (m, 3H), 7.37 – 7.33 (m, 1H); ^{13}C NMR (100 MHz, $CDCl_3$) δ 140.9 (s), 140.8 (s), 140.6 (s), 138.0 (s), 134.1 (s), 130.8 (s), 130.0 (s), 128.8 (s), 128.6 (s), 128.3 (s), 127.4 (s), 127.3 (s), 126.7 (s), 126.2 (s), 126.1 (s), 125.9 (s), 125.4 (s). This compound is known. [1]

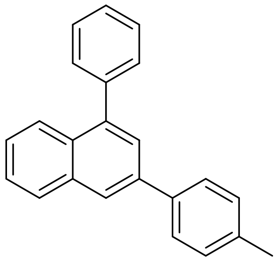

1-phenyl-3-(p-tolyl)naphthalene (3b): The representative general procedure mentioned above was followed. An oven dried 25 mL Schlenk tube was charged with carboxylic acid **1a** (0.2 mmol), DMAP (0.2 mmol, 1.0 eq.), LiBr (0.1 mmol, 0.5 eq.), $PdCl_2$ (5 mol%) and TFP (10 mol%). Subsequently, 4-methylphenylene acetylene **2b** (0.4 mmol, 2.0 eq.), Piv_2O (1.4 eq.) and dioxane (2 mL) was added under N_2. The reaction mixture was allowed to react at 160 °C for 12 h. After completion of the reaction, the reaction mixture was then cooled to room temperature, and then the reaction mixture was

transferred to a round-bottomed bottle and concentrated under reduced pressure. The crude residue was purified by flash chromatography on silica gel (PE) to give the desired **3b** as yellow oil in 71% yield (41.7 mg). ^{1}H NMR (400 MHz, $CDCl_3$) δ 8.11 (s, 1H), 8.01 –7.95 (m, 2H), 7.77 –7.76 (m, 1H), 7.73 – 7.71 (m, 2H), 7.64 – 7.59 (m, 2H), 7.59 – 7.54 (m, 3H), 7.52 – 7.45 (m, 2H), 7.34 (m, 2H), 2.47 (s, 3H); ^{13}C NMR (100 MHz, $CDCl_3$) δ 140.7 (s), 137.9 (s), 137.8 (s), 137.2 (s), 134.2 (s), 130.7 (s), 130.1 (s), 129.6 (s), 128.5 (s), 128.3 (s), 127.3 (s), 127.2 (s), 126.6 (s), 126.1 (s), 125.9 (s), 125.8 (s), 125.0 (s), 21.1 (s). This compound is known. [1]

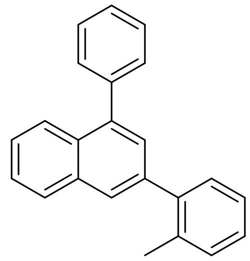

1-phenyl-3-(o-tolyl)naphthalene (3c): The representative general procedure mentioned above was followed. An oven dried 25 mL Schlenk tube was charged with carboxylic acid **1a** (0.2 mmol), DMAP (0.2 mmol, 1.0 eq.), LiBr (0.1 mmol, 0.5 eq.), $PdCl_2$ (5 mol%) and TFP (10 mol%). Subsequently, 2-methylphenylene **2c** (0.4 mmol, 2.0 eq.), Piv_2O (1.4 eq.) and dioxane (2 mL) was added under N_2. The reaction mixture was allowed to react at 160 °C for 12 h. After completion of the reaction, the reaction mixture was then cooled to room temperature, and then the reaction mixture was transferred to a round-bottomed bottle and concentrated under reduced pressure. The crude residue was purified by flash chromatography on silica gel (PE) to give the desired **3c** as yellow oil in 72% yield (42.3 mg). ^{1}H NMR (400 MHz, $CDCl_3$) δ 8.00 – 7.98 (m, 1H), 7.96 – 7.94 (m, 1H), 7.84 (s, 1H), 7.60 – 7.54 (m, 3H), 7.52 (m, 3H), 7.49 – 7.43 (m, 2H), 7.41 (m, 1H), 7.35 – 7.30 (m, 3H), 2.40 (s, 3H); ^{13}C NMR (100 MHz, $CDCl_3$) δ 141.6 (s), 140.6 (s), 139.9 (s), 139.0 (s), 135.6 (s), 133.8 (s), 130.4 (s), 130.3 (s), 130.1 (s), 130.0 (s), 128.8 (s), 128.4 (s), 128.3 (s), 127.5 (s), 127.4 (s), 127.3 (s), 126.1 (s), 126.0 (s), 125.9 (s), 125.8 (s), 20.6 (s). This compound is known. [1]

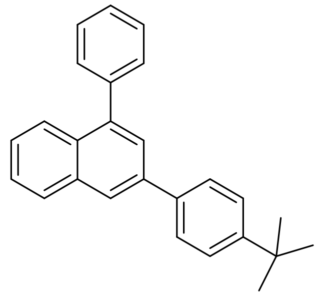

1-(4-(*tert*-butyl)phenyl)-1-phenylnaphthalene (3d): The representative general procedure mentioned above was followed. An oven dried 25 mL Schlenk tube was charged with carboxylic acid **1a** (0.2 mmol), DMAP (0.2 mmol, 1.0 eq.), LiBr (0.1 mmol, 0.5 eq.), $PdCl_2$ (5 mol%) and TFP (10 mol%). Subsequently, 4-*tert*-butylphenylacetylene **2d** (0.4 mmol, 2.0 eq.), Piv_2O (1.4 eq.) and dioxane (2 mL) was added under N_2. The reaction mixture was allowed to react at 160 °C for 12 h. After completion of the reaction, the reaction mixture was then cooled to room temperature, and then the reaction mixture was transferred to a round-bottomed bottle and concentrated under reduced pressure. The crude residue was purified by flash chromatography on silica gel (PE) to give the desired **3d** as yellow oil in 70% yield (47.0 mg). ^1H NMR (400 MHz, $CDCl_3$) δ 8.09 (s, 1H), 7.99 – 7.97 (m, 1H), 7.95 – 7.93 (m, 1H), 7.76 – 7.75 (m, 1H), 7.75 – 7.71 (m, 2H), 7.60 – 7.57 (m, 2H), 7.56 – 7.55 (m, 2H), 7.54 – 7.51 (m, 3H), 7.50 – 7.42 (m, 2H), 1.41 (s, 9H); ^{13}C NMR (100 MHz, $CDCl_3$) δ 150.5 (s), 140.7 (s), 140.6 (s), 137.9 (s), 137.8 (s), 134.2 (s), 130.7 (s), 130.1 (s), 128.5 (s), 128.3 (s), 127.3 (s), 127.1 (s), 126.7 (s), 126.1 (s), 126.0 (s), 125.9 (s), 125.8 (s), 125.1 (s), 34.6 (s), 31.4 (s). HRMS (APCI-POS) m/z: $[M+H]^+$ calcd. $C_{26}H_{25}^+$: 337.1951, found: 337.1942.

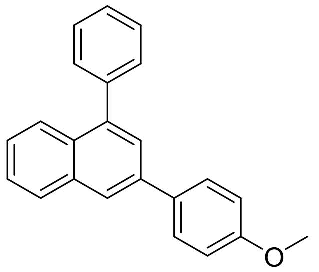

3-(4-methoxyphenyl)-1-phenylnaphthalene (3e): The representative general procedure mentioned above was followed. An oven dried 25 mL Schlenk tube was charged with carboxylic acid **1a** (0.2 mmol), DMAP (0.2 mmol, 1.0 eq.), LiBr (0.1 mmol, 0.5 eq.), $PdCl_2$ (5 mol%) and TFP (10 mol%). Subsequently, 4-methoxyphenylene **2e** (0.4 mmol, 2.0 eq.), Piv_2O (1.4 eq.) and dioxane (2 mL) was added under N_2. The reaction mixture was allowed to react at 160 °C for 12 h. After completion of the reaction, the reaction mixture was then cooled to room temperature, and then the reaction mixture was transferred to a round-bottomed bottle and concentrated under reduced pressure. The crude residue was purified by flash chromatography on silica gel (PE) to give the desired **3e** as yellow oil in 89% yield (55.2 mg). ^1H NMR (400 MHz, $CDCl_3$) δ 8.02 (s, 1H), 7.96 – 7.94 (m, 1H), 7.91 – 7.89 (m, 1H), 7.73 – 7.67 (m, 3H), 7.58 – 7.54 (m, 2H), 7.54 – 7.49 (m, 3H), 7.47 – 7.46 (m, 1H), 7.44 – 7.40 (m, 1H), 7.04 –7.02 (m, 2H), 3.88 (s, 3H); ^{13}C NMR (100 MHz, $CDCl_3$) δ 159.3 (s), 140.7 (s), 137.4 (s), 134.2

(s), 133.4 (s), 130.5 (s), 130.1 (s), 128.4 (s), 128.3 (s), 127.3 (s), 126.5 (s), 126.1 (s), 125.9 (s), 125.8 (s), 124.6 (s), 114.3 (s), 55.4 (s). This compound is known.[1]

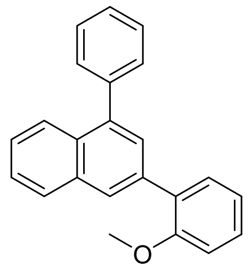

3-(2-methoxyphenyl)-1-phenylnaphthalene (3f): The representative general procedure mentioned above was followed. An oven dried 25 mL Schlenk tube was charged with carboxylic acid **1a** (0.2 mmol), DMAP (0.2 mmol, 1.0 eq.), LiBr (0.1 mmol, 0.5 eq.), $PdCl_2$ (5 mol%) and TFP (10 mol%). Subsequently, 2-methoxyphenylene **2f** (0.4 mmol, 2.0 eq.), Piv_2O (1.4 eq.) and dioxane (2 mL) was added under N_2. The reaction mixture was allowed to react at 160 °C for 12 h. After completion of the reaction, the reaction mixture was then cooled to room temperature, and then the reaction mixture was transferred to a round-bottomed bottle and concentrated under reduced pressure. The crude residue was purified by flash chromatography on silica gel (PE) to give the desired **3f** as yellow oil in 69% yield (42.8 mg). ^{1}H NMR (400 MHz, $CDCl_3$) δ 8.05 – 7.96 (m, 3H), 7.70 – 7. 60 (m, 2H), 7.60 – 7. 52 (m, 4H), 7.40 – 7. 26 (m, 4H), 7.11 – 7.05 (m, 2H), 3.87 (s, 3H); ^{13}C NMR (100 MHz, $CDCl_3$) δ 156.7 (s), 140.8 (s), 139.6 (s), 135.6 (s), 133.8 (s), 131.2 (s), 130.6 (s), 130.4 (s), 130.2 (s), 129.1 (s), 128.8 (s), 128.5 (s), 128.2 (s), 127.8 (s), 127.2 (s), 125.9 (s), 125.8 (s), 125.8 (s), 120.9 (s), 111.3 (s), 55.6 (s). HRMS (APCI-POS) m/z: $[M+H]^+$ calcd. for $C_{23}H_{19}O^+$: 311.1430, found: 311.1426.

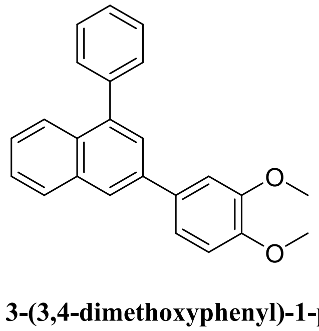

3-(3,4-dimethoxyphenyl)-1-phenylnaphthalene (3g): The representative general procedure mentioned above was followed. An oven dried 25 mL Schlenk tube was charged with carboxylic acid **1a** (0.2 mmol), DMAP (0.2 mmol, 1.0 eq.), LiBr (0.1 mmol, 0.5 eq.), $PdCl_2$ (5 mol%) and TFP (10 mol%). Subsequently, 3, 4-dimethoxy-phenylacetylene **2g** (0.4 mmol, 2.0 eq.), Piv_2O (1.4 eq.) and dioxane (2 mL) was added under N_2. The reaction mixture was allowed to react at 160 °C for 12 h.

After completion of the reaction, the reaction mixture was then cooled to room temperature, and then the reaction mixture was transferred to a round-bottomed bottle and concentrated under reduced pressure. The crude residue was purified by flash chromatography on silica gel (PE) to give the desired **3g** as yellow oil in 71% yield (48.1 mg). ^{1}H NMR (400 MHz, $CDCl_3$) δ 8.03 (s, 1H), 7.98 – 7.90 (m, 2H), 7.70 (d, J = 1.6 Hz, 1H), 7.60 – 7.59 (m, 2H), 7.56 – 7.52 (m, 3H), 7.48– 7.41 (m, 2H), 7.34 – 7.28 (m, 2H), 7.00 (d, J = 8.0 Hz, 1H), 4.00 (s, 3H), 3.96 (s, 3H); ^{13}C NMR (100 MHz, $CDCl_3$) δ 149.3 (s), 148.8 (s), 140.7 (s), 140.6 (s), 137. 8 (s), 134.2 (s), 133.8 (s), 130.6 (s), 130.1 (s), 128.4 (s), 128.3 (s), 127.3 (s), 126.5 (s), 126.2 (s), 125.9 (s), 124.8 (s), 119.7 (s), 111.5 (s), 110.6 (s), 56.0 (s), 55.9 (s). HRMS (APCI-POS) m/z: $[M+H]^+$ calcd. for $C_{24}H_{21}O_2^+$: 341.1536, found: 341.1532.

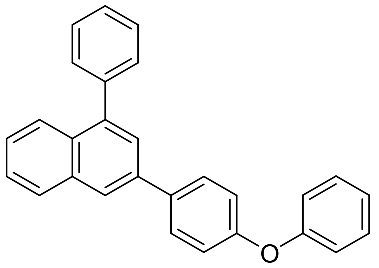

3-(4-phenoxyphenyl)-1-phenylnaphthalene (3h): The representative general procedure mentioned above was followed. An oven dried 25 mL Schlenk tube was charged with carboxylic acid **1a** (0.2 mmol), DMAP (0.2 mmol, 1.0 eq.), LiBr (0.1 mmol, 0.5 eq.), $PdCl_2$ (5 mol%) and TFP (10 mol%). Subsequently, 4-phenoxyphenylacetylene **2h** (0.4 mmol, 2.0 eq.), Piv_2O (1.4 eq.) and dioxane (2 mL) was added under N_2. The reaction mixture was allowed to react at 160 °C for 12 h. After completion of the reaction, the reaction mixture was then cooled to room temperature, and then the reaction mixture was transferred to a round-bottomed bottle and concentrated under reduced pressure. The crude residue was purified by flash chromatography on silica gel (PE) to give the desired **3h** as yellow oil in 84% yield (62.4 mg). ^{1}H NMR (400 MHz, $CDCl_3$) δ 8.06 (s, 1H), 7.99 – 7.93 (m, 2H), 7.76 – 7.72 (m, 3H), 7.60 – 7.58 (m, 3H), 7.56 – 7.52 (m, 2H), 7.50 – 7.47 (m, 2H), 7.42 – 7.38 (m, 2H), 7.20 – 7.17 (m, 3H), 7.12– 7.10 (m, 2H); ^{13}C NMR (100 MHz, $CDCl_3$) δ 157.1 (s), 156.9 (s), 140.9 (s), 140.6 (s), 137.3 (s), 135.9 (s), 134.2 (s), 130.7 (s), 130.1 (s), 129.8 (s), 128.7 (s), 128.5 (s), 128.3 (s), 127.4 (s), 126.5 (s), 126.2 (s), 126.0 (s), 125.9 (s), 124.9 (s), 123.4 (s), 119.2 (s), 118.9 (s). HRMS (APCI-POS) m/z: $[M+H]^+$ calcd. $C_{23}H_{19}O^+$: 373.1587, found: 373.1590.

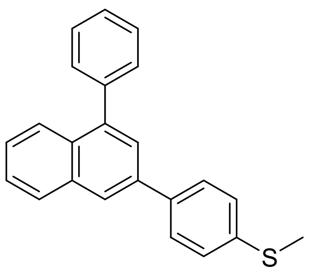

methyl(4-(4-phenylnaphthalen-2-yl)phenyl)sulfane (3i): The representative general procedure mentioned above was followed. An oven dried 25 mL Schlenk tube was charged with carboxylic acid **1a** (0.2 mmol), DMAP (0.2 mmol, 1.0 eq.), LiBr (0.1 mmol, 0.5 eq.), $PdCl_2$ (5 mol%) and TFP (10 mol%). Subsequently, (4-ethynylphenyl)(methyl)sulfane **2i** (0.4 mmol, 2.0 eq.), Piv_2O (1.4 eq.) and dioxane (2 mL) was added under N_2. The reaction mixture was allowed to react at 160 °C for 12 h. After completion of the reaction, the reaction mixture was then cooled to room temperature, and then the reaction mixture was transferred to a round-bottomed bottle and concentrated under reduced pressure. The crude residue was purified by flash chromatography on silica gel (PE) to give the desired **3i** as yellow oil in 77% yield (50.1 mg). ^1H NMR (400 MHz, $CDCl_3$) δ 8.07 (s, 1H), 7.98 – 7.93 (m, 2H), 7.73 – 7.72 (m, 2H), 7.71 – 7.70 (m, 1H), 7.59 – 7.56 (m, 3H), 7.54 – 7.50 (m, 2H), 7.48 – 7.43 (m, 2H), 7.40 – 7.38 (m, 2H), 2.56 (s, 3H); ^{13}C NMR (100 MHz, $CDCl_3$) δ 140.8 (s), 140.6 (s), 137.8 (s), 137.5 (s), 137.2 (s), 134.1 (s), 130.7 (s), 130.0 (s), 128.5 (s), 128.3 (s), 127.7 (s), 127.3 (s), 126.9 (s), 126.3 (s), 126.2 (s), 126.0 (s), 125.8 (s), 124.9 (s), 15.8 (s). HRMS (APCI-POS) m/z: $[M+H]^+$ calcd. $C_{23}H_{18}S$: 327.1202, found: 327.1201.

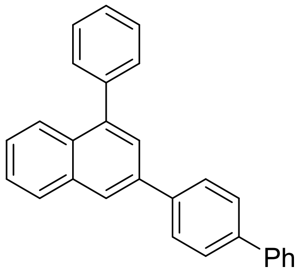

1-([1,1'-biphenyl]-4-yl)-1-phenylnaphthalene (3j): The representative general procedure mentioned above was followed. An oven dried 25 mL Schlenk tube was charged with carboxylic acid **1a** (0.2 mmol), DMAP (0.2 mmol, 1.0 eq.), LiBr (0.1 mmol, 0.5 eq.), $PdCl_2$ (5 mol%) and TFP (10 mol%). Subsequently, 4-phenylphenylene **2j** (0.4 mmol, 2.0 eq.), Piv_2O (1.4 eq.) and dioxane (2 mL) was added under N_2. The reaction mixture was allowed to react at 160 °C for 12 h. After completion of the reaction, the reaction mixture was then cooled to room temperature, and then the reaction mixture

was transferred to a round-bottomed bottle and concentrated under reduced pressure. The crude residue was purified by flash chromatography on silica gel (PE) to give the desired **3j** as a yellow solid in 57% yield (40.5 mg). mp: 127-128 °C; ^{1}H NMR (400 MHz, $CDCl_3$) δ 8.14 (s, 1H), 8.01 – 7.94 (m, 2H), 7.87 – 7.77 (m, 2H), 7.79 – 7.78 (m, 1H), 7.75 – 7.73 (m, 2H), 7.70 – 7.68 (m, 2H), 7.60 – 7.58 (m, 3H), 7.55 – 7.53 (m, 2H), 7.51 – 7.48 (m, 4H), 7.41 – 7.38 (m, 1H); ^{13}C NMR (100 MHz, $CDCl_3$) δ 140.9 (s), 140.7 (s), 140.3 (s), 139.8 (s), 137.5 (s), 134.3 (s), 130.9 (s), 130.2 (s), 128.9 (s), 128.7 (s), 128.4 (s), 127.8 (s), 127.7 (s), 127.5 (s), 127.1 (s), 126.6 (s), 126.3 (s), 126.2 (s), 125.9 (s), 125.2 (s). HRMS (APCI-POS) m/z: $[M]^+$ calcd. For $C_{28}H_{19}^+$: 355.1482, found: 355.1487.

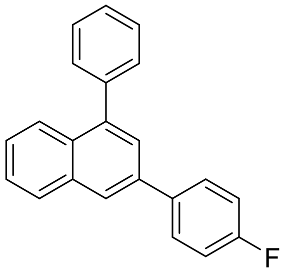

1-(4-fluorophenyl)-1-phenylnaphthalene (3k): The representative general procedure mentioned above was followed. An oven dried 25 mL Schlenk tube was charged with carboxylic acid **1a** (0.2 mmol), DMAP (0.2 mmol, 1.0 eq.), LiBr (0.1 mmol, 0.5 eq.), $PdCl_2$ (5 mol%) and TFP (10 mol%). Subsequently, 4-fluorophenylene **2k** (0.4 mmol, 2.0 eq.), Piv_2O (1.4 eq.) and dioxane (2 mL) was added under N_2. The reaction mixture was allowed to react at 160 °C for 12 h. After completion of the reaction, the reaction mixture was then cooled to room temperature, and then the reaction mixture was transferred to a round-bottomed bottle and concentrated under reduced pressure. The crude residue was purified by flash chromatography on silica gel (PE) to give the desired **3k** as yellow oil in 53% yield (31.6 mg). ^{1}H NMR (400 MHz, $CDCl_3$) δ 8.03 (s, 1H), 7.98 – 7.93 (m, 2H), 7.74 – 7.71 (m, 2H), 7.69 – 7.68 (dm, 1H), 7.58 – 7.56 (m, 3H), 7.54 – 7.53 (m, 2H), 7.50 – 7.44 (m, 2H), 7.22 – 7.16 (m, 2H); ^{13}C NMR (100 MHz, $CDCl_3$) δ 162.5 (d, J = 245.2 Hz), 140.7 (d, J = 42.2 Hz), 136.9 (s), 134.1 (s), 130.8 (s), 130.0 (s), 128.9 (s), 128.9 (s), 128.5 (s), 128.3 (s), 127.4 (s), 126.5 (s), 126.3 (s), 126.1 (s), 125.9 (s), 125.2 (s), 115.7 (d, J = 21.3 Hz); ^{19}F NMR (376 MHz, $CDCl_3$) δ -115.4 (s). This compound is known.[2]

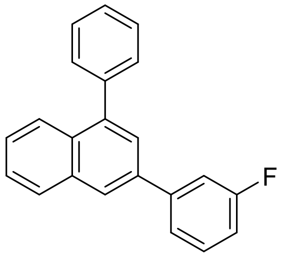

1-(3-fluorophenyl)-1-phenylnaphthalene (3l): The representative general procedure mentioned above was followed. An oven dried 25 mL Schlenk tube was charged with carboxylic acid **1a** (0.2 mmol), DMAP (0.2 mmol, 1.0 eq.), LiBr (0.1 mmol, 0.5 eq.), $PdCl_2$ (5 mol%) and TFP (10 mol%). Subsequently, 3-fluorophenylene **2l** (0.4 mmol, 2.0 eq.), Piv_2O (1.4 eq.) and dioxane (2 mL) was added under N_2. The reaction mixture was allowed to react at 160 °C for 12 h. After completion of the reaction, the reaction mixture was then cooled to room temperature, and then the reaction mixture was transferred to a round-bottomed bottle and concentrated under reduced pressure. The crude residue was purified by flash chromatography on silica gel (PE) to give the desired **3l** as yellow oil in 65% yield (36.9 mg). ^{1}H NMR (400 MHz, $CDCl_3$) δ 8.10 (s, 1H), 8.01 (td, *J* = 7.0, 0.8 Hz, 2H), 7.76 – 7.75 (m, 1H), 7.63 – 7.61 (m, 2H), 7.60 – 7.58 (m, 3H), 7.57 – 7.56 (m, 1H), 7.55 – 7.50 (m, 3H), 7.48 – 7.44 (m, 1H), 7.17 – 7.10 (m, 1H); ^{13}C NMR (100 MHz, $CDCl_3$) δ 163.2 (d, *J* = 244.2 Hz), 143.1 (d, *J* = 7.6 Hz), 141.1 (d, *J* = 7.6 Hz), 136.6 (s), 134.0 (s), 131.1 (s), 130.3 (d, *J* = 8.4 Hz), 130.01 (s), 128.6 (s), 128.3 (s), 127.4 (s), 126.4 (s), 126.3 (s), 126.2 (s), 125.9 (s), 125.5 (s), 122.98 (d, *J* = 2.4 Hz), 114.3 (d, *J* = 5.1 Hz), 114.1 (d, *J* = 4.2 Hz); ^{19}F NMR (376 MHz, $CDCl_3$) δ -112.9 (s).[6] This compound is known.[3]

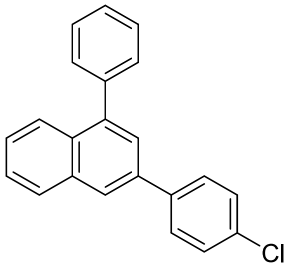

3-(4-chlorophenyl)-1-phenylnaphthalene (3m): The representative general procedure mentioned above was followed. An oven dried 25 mL Schlenk tube was charged with carboxylic acid **1a** (0.2 mmol), DMAP (0.2 mmol, 1.0 eq.), LiBr (0.1 mmol, 0.5 eq.), $PdCl_2$ (5 mol%) and TFP (10 mol%). Subsequently, 4-chlorophenylene **2m** (0.4 mmol, 2.0 eq.), Piv_2O (1.4 eq.) and dioxane (2 mL) was added under N_2. The reaction mixture was allowed to react at 160 °C for 12 h. After completion of the

reaction, the reaction mixture was then cooled to room temperature, and then the reaction mixture was transferred to a round-bottomed bottle and concentrated under reduced pressure. The crude residue was purified by flash chromatography on silica gel (PE) to give the desired **3m** as yellow oil in 72% yield (45.2 mg). ^{1}H NMR (400 MHz, $CDCl_3$) δ 8.04 (s, 1H), 7.98 – 7.92 (m, 2H), 7.70 – 7.67 (m, 3H), 7.57 – 7.54 (m, 3H), 7.53 – 7.51 (m, 2H), 7.50 – 7.44 (m, 4H); ^{13}C NMR (100 MHz, $CDCl_3$) δ 141.0 (s), 140.5 (s), 139.3 (s), 136.7 (s), 134.1 (s), 133.5 (s), 130.9 (s), 130.0 (s), 128.9 (s), 128.6 (s), 128.6 (s), 128.3 (s), 127.4 (s), 126.4 (s), 126.3 (s), 126.2 (s), 125.9 (s), 125.2 (s). This compound is known. [1]

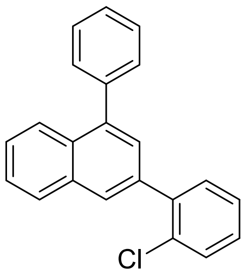

1-(2-chlorophenyl)-1-phenylnaphthalene (3n): The representative general procedure mentioned above was followed. An oven dried 25 mL Schlenk tube was charged with carboxylic acid **1a** (0.2 mmol), DMAP (0.2 mmol, 1.0 eq.), LiBr (0.1 mmol, 0.5 eq.), $PdCl_2$ (5 mol%) and TFP (10 mol%). Subsequently, 2-chlorophenylene **2n** (0.4 mmol, 2.0 eq.), Piv_2O (1.4 eq.) and dioxane (2 mL) was added under N_2. The reaction mixture was allowed to react at 160 °C for 12 h. After completion of the reaction, the reaction mixture was then cooled to room temperature, and then the reaction mixture was transferred to a round-bottomed bottle and concentrated under reduced pressure. The crude residue was purified by flash chromatography on silica gel (PE) to give the desired **3n** as a yellow solid in 73% yield (45.8 mg). mp: 104-105 °C; ^{1}H NMR (400 MHz, $CDCl_3$) δ 7.96 – 7.90 (m, 3H), 7.58 – 7.53 (m, 3H), 7.51 – 7.50 (m, 1H), 7.50 – 7.46 (m, 4H), 7.45 – 7.38 (m, 2H), 7.36 – 7.19 (m, 3H); ^{13}C NMR (100 MHz, $CDCl_3$) δ 140.4 (s), 140.3 (s), 139.9 (s), 136.4 (s), 134.4 (s), 133.6 (s), 132.7 (s), 131.6 (s), 130.8 (s), 130.1 (s), 130.0 (s), 128.7 (s), 128.6 (s), 128.3 (s), 127.9 (s), 127.3 (s), 126.9 (s), 126.4 (s), 126.1 (s), 125.9 (s). HRMS (APCI-POS) m/z: $[M+H]^+$ calcd. for $C_{22}H_{16}Cl^+$: 315.0936, found: 315.0936.

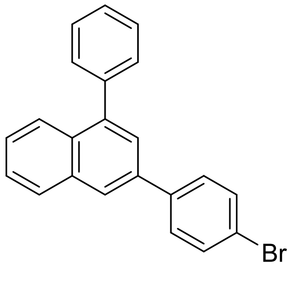

3-(4-bromophenyl)-1-phenylnaphthalene (3o): The representative general procedure mentioned above was followed. An oven dried 25 mL Schlenk tube was charged with carboxylic acid **1a** (0.2 mmol), DMAP (0.2 mmol, 1.0 eq.), LiBr (0.1 mmol, 0.5 eq.), $PdCl_2$ (5 mol%) and TFP (10 mol%). Subsequently, 4-bromophenylacetylene **2o** (0.4 mmol, 2.0 eq.), Piv_2O (1.4 eq.) and dioxane (2 mL) was added under N_2. The reaction mixture was allowed to react at 160 °C for 12 h. After completion of the reaction, the reaction mixture was then cooled to room temperature, and then the reaction mixture was transferred to a round-bottomed bottle and concentrated under reduced pressure. The crude residue was purified by flash chromatography on silica gel (PE) to give the desired **3o** as a yellow solid in 61% yield (43.7 mg). ^1H NMR (400 MHz, $CDCl_3$) δ 8.04 (s, 1H), 7.98 – 7.91 (m, 2H), 7.67 – 7.66 (m, 1H), 7.62 – 7.61 (m, 3H), 7.56 – 7.55 (m, 3H), 7.53 – 7.51 (m, 2H), 7.49 – 7.44 (m, 2H); ^{13}C NMR (100 MHz, $CDCl_3$) δ 141.1 (s), 140.4 (s), 139.8 (s), 136.7 (s), 134.1 (s), 131.9 (s), 130.9 (s), 130.0 (s), 128.9 (s), 128.6 (s), 128.3 (s), 127.5 (s), 126.4 (s), 126.3 (s), 126.2 (s), 125.9 (s), 125.3 (s), 121.7 (s). This compound is known.[2]

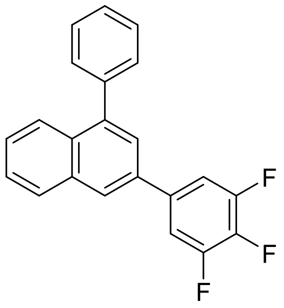

1-phenyl-3-(3,4,5-trifluorophenyl)naphthalene (3p): The representative general procedure mentioned above was followed. An oven dried 25 mL Schlenk tube was charged with carboxylic acid **1a** (0.2 mmol), DMAP (0.2 mmol, 1.0 eq.), LiBr (0.1 mmol, 0.5 eq.), $PdCl_2$ (5 mol%) and TFP (10 mol%). Subsequently, 3, 4, 5-trifluorophenylene **2p** (0.4 mmol, 2.0 eq.), Piv_2O (1.4 eq.) and dioxane (2 mL) was added under N_2. The reaction mixture was allowed to react at 160 °C for 12 h. After completion of the reaction, the reaction mixture was then cooled to room temperature, and then the reaction mixture was transferred to a round-bottomed bottle and concentrated under reduced pressure. The crude residue was purified by flash chromatography on silica gel (PE) to give the desired **3p** as yellow oil in 64% yield (42.7 mg). ^1H NMR (400 MHz, $CDCl_3$) δ 7.98 – 7.95 (m, 2H), 7.93 – 7.91 (m, 1H), 7.59 – 7.56 (m, 2H), 7.54 – 7.53 (m, 4H), 7.50 – 7.48 (m, 2H), 7.36 (dd, J = 8.8, 6.4 Hz, 2H); ^{13}C NMR (100 MHz, $CDCl_3$) δ 151.51 (ddd, J = 248.0, 10.0, 4.2 Hz), 141.5 (s), 140.1 (s), 139.3 (td, J =

250.5, 15.3 Hz), 137.32 – 136.79 (m), 134.9 (s), 133.9 (s), 131.3 (s), 129.9 (s), 128.6 (s), 128.4 (s), 127.6 (s), 126.7 (s), 126.7 (s), 126.0 (s), 125.6 (s), 125.5 (s), 111.25 (dd, *J* = 15.8, 5.9 Hz); ^{19}F NMR (376 MHz, $CDCl_3$) δ -134.2 (dd, *J* = 20.4, 8.0 Hz), -162.5 – -162.6 (m). HRMS (APCI-POS) m/z: $[M+Na]^+$ calcd. for $C_{22}H_{13}F_3Na^+$: 362.1253, found: 362.1248.

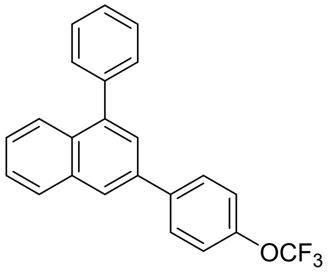

1-phenyl-3-(4-(trifluoromethoxy)phenyl)naphthalene (3q): The representative general procedure mentioned above was followed. An oven dried 25 mL Schlenk tube was charged with carboxylic acid **1a** (0.2 mmol), DMAP (0.2 mmol, 1.0 eq.), LiBr (0.1 mmol, 0.5 eq.), $PdCl_2$ (5 mol%) and TFP (10 mol%). Subsequently, 4-trifluoromethoxyphenylene **2q** (0.4 mmol, 2.0 eq.), Piv_2O (1.4 eq.) and dioxane (2 mL) was added under N_2. The reaction mixture was allowed to react at 160 °C for 12 h. After completion of the reaction, the reaction mixture was then cooled to room temperature, and then the reaction mixture was transferred to a round-bottomed bottle and concentrated under reduced pressure. The crude residue was purified by flash chromatography on silica gel (PE) to give the desired **3q** as yellow oil in 84% yield (61.1 mg). ^1H NMR (400 MHz, $CDCl_3$) δ 8.05 (s, 1H), 7.99 – 7.93 (m, 2H), 7.78 – 7.76 (m, 2H), 7.68 (d, *J* = 1.6 Hz, 1H), 7.58 – 7.55 (m, 3H), 7.54 – 7.52 (m, 2H), 7.50 – 7.44 (m, 2H), 7.36 – 7.34 (m, 2H); ^{13}C NMR (100 MHz, $CDCl_3$) δ 148.8 (s), 141.1 (s), 140.5 (s), 139.7 (s), 136.6 (s), 134.1 (s), 130.9 (s), 130.0 (s), 128.7 (s), 128.6 (s), 128.4 (s), 127.5 (s), 126.4 (s), 126.4 (s), 126.3 (s), 125.9 (s), 125.5 (s), 121.3 (s); ^{19}F NMR (376 MHz, $CDCl_3$) δ -57.7 (s). HRMS (APCI-POS) m/z: $[M+H]^+$ calcd. for $C_{24}H_{18}F_3O^+$: 379.1305, found: 379.1305.

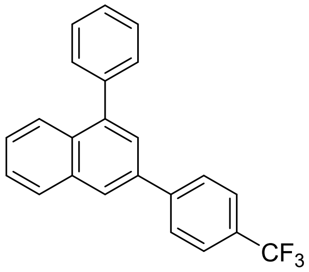

1-phenyl-3-(4-(trifluoromethyl)phenyl)naphthalene (3r): The representative general procedure mentioned above was followed. An oven dried 25 mL Schlenk tube was charged with carboxylic acid

1a (0.2 mmol), DMAP (0.2 mmol, 1.0 eq.), LiBr (0.1 mmol, 0.5 eq.), $PdCl_2$ (5 mol%) and TFP (10 mol%). Subsequently, 4-trifluoromethyl phenylacetylene **2r** (0.4 mmol, 2.0 eq.), Piv_2O (1.4 eq.) and dioxane (2 mL) was added under N_2. The reaction mixture was allowed to react at 160 °C for 12 h. After completion of the reaction, the reaction mixture was then cooled to room temperature, and then the reaction mixture was transferred to a round-bottomed bottle and concentrated under reduced pressure. The crude residue was purified by flash chromatography on silica gel (PE) to give the desired **3r** as yellow oil in 70% yield (48.7 mg). ^1H NMR (400 MHz, $CDCl_3$) δ 8.09 (s, 1H), 8.00 – 7.94 (m, 2H), 7.88 – 7.86 (m, 2H), 7.76 – 7.74 (m, 2H), 7.71 (d, *J* = 1.8 Hz, 1H), 7.58 – 7.56 (m, 3H), 7.54 – 7.53 (m, 2H), 7.50 – 7.47 (m, 2H). ^{13}C NMR (100 MHz, $CDCl_3$) δ 144.4 (s), 141.3 (s), 140.4 (s), 136.5 (s), 134.0 (s), 131.2 (s), 130.0 (s), 128.7 (s), 128.4 (s), 127.7 (s), 127.4 (s), 126.6 (s), 126.5 (s), 126.2 (s), 125.9 (s), 125.9 (s), 125.78 (dd, *J* = 7.3, 3.6 Hz); ^{19}F NMR (376 MHz, $CDCl_3$) δ -62.3 (s).This compound is known.[2]

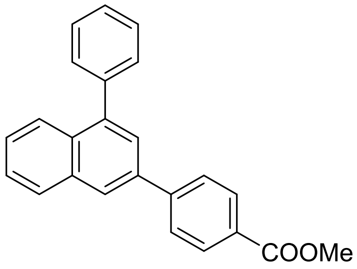

methyl 4-(4-phenylnaphthalen-2-yl)benzoate (3s): The representative general procedure mentioned above was followed. An oven dried 25 mL Schlenk tube was charged with carboxylic acid **1a** (0.2 mmol), DMAP (0.2 mmol, 1.0 eq.), LiBr (0.1 mmol, 0.5 eq.), $PdCl_2$ (5 mol%) and TFP (10 mol%). Subsequently, 4-ethynylbenzoate **2s** (0.4 mmol, 2.0 eq.), Piv_2O (1.4 eq.) and dioxane (2 mL) was added under N_2. The reaction mixture was allowed to react at 160 °C for 12 h. After completion of the reaction, the reaction mixture was then cooled to room temperature, and then the reaction mixture was transferred to a round-bottomed bottle and concentrated under reduced pressure. The crude residue was purified by flash chromatography on silica gel (PE/EA=20/1) to give the desired **3s** as yellow oil in 70% yield (47.3 mg). ^1H NMR (400 MHz, $CDCl_3$) δ 8.16 – 8.14 (s, 2H), 8.12 (s, 1H), 7.99 – 7.97 (m, 1H), 7.93 – 7.91 (m, 1H), 7.84 – 7.82 (m, 2H), 7.73 – 7.72 (m, 1H), 7.56 – 7.54 (m, 3H), 7.53 – 7.51 (s, 2H), 7.48 – 7.45 (m, 2H), 3.96 (s, 3H); ^1H NMR (400 MHz, $CDCl_3$) δ 8.14 (s, 2H), 8.12 (s, 1H), 7.97 (s, 1H), 7.91 (s, 1H), 7.84 (s, 2H), 7.73 (s, 1H), 7.55 (s, 3H), 7.53 (s, 2H), 7.49 (s, 2H), 3.96 (s, 3H). ^{13}C NMR (100 MHz, $CDCl_3$) δ 166.9 (s), 145.3 (s), 141.1 (s), 140.4 (s), 136.7 (s), 134.0 (s), 131.2 (s),

130.2 (s), 130.0 (s), 128.9 (s), 128.0 (s), 128.4 (s), 127.5 (s), 127.3 (s), 126.6 (s), 126.4 (s), 126.2 (s), 125.9 (s), 125. 9 (s), 52.2 (s). HRMS (APCI-POS) m/z: $[M+H]^+$ calcd. for $C_{24}H_{19}O_2^+$: 339.1830, found: 339.1830.

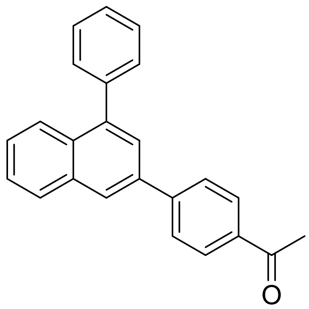

1-(4-(4-phenylnaphthalen-2-yl)phenyl)ethan-1-one (3t): The representative general procedure mentioned above was followed. An oven dried 25 mL Schlenk tube was charged with carboxylic acid **1a** (0.2 mmol), DMAP (0.2 mmol, 1.0 eq.), LiBr (0.1 mmol, 0.5 eq.), $PdCl_2$ (5 mol%) and TFP (10 mol%). Subsequently, 4-acetyl phenylene **2t** (0.4 mmol, 2.0 eq.), Piv_2O (1.4 eq.) and dioxane (2 mL) was added under N_2. The reaction mixture was allowed to react at 160 °C for 12 h. After completion of the reaction, the reaction mixture was then cooled to room temperature, and then the reaction mixture was transferred to a round-bottomed bottle and concentrated under reduced pressure. The crude residue was purified by flash chromatography on silica gel (PE/EA=20/1) to give the desired **3t** as a yellow solid in 70% yield (45.0 mg). mp: 118-119 °C ; ^{1}H NMR (400 MHz, $CDCl_3$) δ 8.09 (s, 1H), 8.07 – 8.01 (m, 2H), 7.96 – 7.94 (m, 1H), 7.91 – 7.98 (m, 1H), 7.83 – 7.81 (m, 2H), 7.71 – 7.70 (m, 1H), 7.55 – 7.52 (m, 3H), 7.52 – 7.47 (m, 2H), 7.47 – 7.42 (m, 2H), 2.63 (s, 3H); ^{13}C NMR (100 MHz, $CDCl_3$) δ 197.70 (s), 145.4 (s), 141.1 (s), 140.3 (s), 136.5 (s), 135.9 (s), 133.9 (s), 131.2 (s), 129.9 (s), 128.9 (s), 128.7 (s), 128.3 (s), 127.5 (s), 127.4 (s), 126.6 (s), 126.4 (s), 126.1 (s), 125.9 (s), 125. 9 (s), 26.6 (s). HRMS (APCI-POS) m/z: $[M+H]^+$ calcd. for $C_{24}H_{19}O^+$: 323.1431, found: 323.1435.

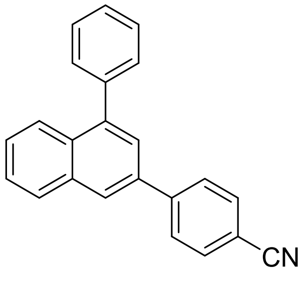

1-(4-phenylnaphthalen-2-yl)benzonitrile (3u): The representative general procedure mentioned above was followed. An oven dried 25 mL Schlenk tube was charged with carboxylic acid **1a** (0.2

mmol), DMAP (0.2 mmol, 1.0 eq.), LiBr (0.1 mmol, 0.5 eq.), $PdCl_2$ (5 mol%) and TFP (10 mol%). Subsequently, 4-cyanophenylacetylene **2u** (0.4 mmol, 2.0 eq.), Piv_2O (1.4 eq.) and dioxane (2 mL) was added under N_2. The reaction mixture was allowed to react at 160 °C for 12 h. After completion of the reaction, the reaction mixture was then cooled to room temperature, and then the reaction mixture was transferred to a round-bottomed bottle and concentrated under reduced pressure. The crude residue was purified by flash chromatography on silica gel (PE/EA=20/1) to give the desired **3u** as a yellow solid in 68% yield (41.4 mg). mp: 116-117 °C; ^1H NMR (400 MHz, $CDCl_3$) δ 8.08 (s, 1H), 7.99 – 7.97 (m, 1H), 7.94 – 7.92 (m, 1H), 7.85 – 7.84 (m, 2H), 7.77 – 7.75 (m, 2H), 7.67 – 7.66 (m, 1H), 7.59 – 7.52 (m, 5H), 7.51 – 7.47 (m, 2H); ^{13}C NMR (100 MHz, $CDCl_3$) δ 145.4 (s), 141.4 (s), 140.2 (s), 135.8 (s), 133.9 (s), 132.6 (s), 131.4 (s), 129.9 (s), 128.7 (s), 128.4 (s), 127.9 (s), 127.6 (s), 126.9 (s), 126.6 (s), 126.1 (s), 125.9 (s), 125.8 (s), 118.9 (s), 110.9 (s). HRMS (APCI-NEG) m/z: $[M]^-$ calcd. For $C_{23}H_{14}N^-$: 305.1210, found: 305.1203.

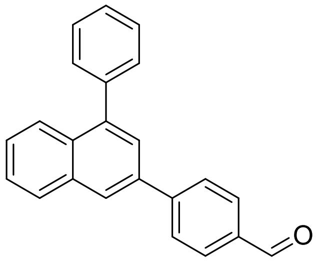

4-(4-phenylnaphthalen-2-yl)benzaldehyde (3v): The representative general procedure mentioned above was followed. An oven dried 25 mL Schlenk tube was charged with carboxylic acid **1a** (0.2 mmol), DMAP (0.2 mmol, 1.0 eq.), LiBr (0.1 mmol, 0.5 eq.), $PdCl_2$ (5 mol%) and TFP (10 mol%). Subsequently, 4-ethynylbezaldehyde **2v** (0.4 mmol, 2.0 eq.), Piv_2O (1.4 eq.) and dioxane (2 mL) was added under N_2. The reaction mixture was allowed to react at 160 °C for 12 h. After completion of the reaction, the reaction mixture was then cooled to room temperature, and then the reaction mixture was transferred to a round-bottomed bottle and concentrated under reduced pressure. The crude residue was purified by flash chromatography on silica gel (PE/EA=20/1) to give the desired **3v** as a yellow solid in 72% yield (44.3 mg). mp: 89 – 90 °C; ^1H NMR (400 MHz, $CDCl_3$) δ 10.05 (s, 1H), 8.11 (s, 1H), 7.99 – 7.95 (m, 3H), 7.92– 7.88 (m, 3H), 7.71– 7.70 (m, 1H), 7.57 – 7.53 (m, 2H), 7.53 – 7.51 (m, 2H), 7.48– 7.46 (m, 1H), 7.45– 7.44 (m, 1H); ^{13}C NMR (100 MHz, $CDCl_3$) δ 191. 9 (s), 146.8 (s), 141.3 (s), 140.3 (s), 136.4 (s), 135.2 (s), 133.9 (s), 131.4 (s), 130.3 (s), 129.9 (s), 128.8 (s), 128.4 (s), 127.9 (s), 127.5

(s), 126.8 (s), 126.5 (s), 126.2 (s), 126.1 (s), 125.9 (s). HRMS (APCI-POS) m/z: $[M+H]^+$ calcd. for $C_{23}H_{17}O^+$: 309.1274, found: 309.1274.

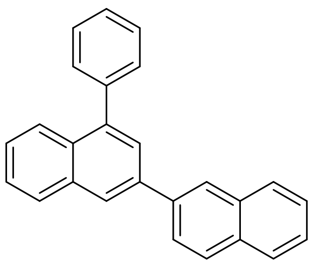

4-phenyl-2,2'-binaphthalene (3w): The representative general procedure mentioned above was followed. An oven dried 25 mL Schlenk tube was charged with carboxylic acid **1a** (0.2 mmol), DMAP (0.2 mmol, 1.0 eq.), LiBr (0.1 mmol, 0.5 eq.), $PdCl_2$ (5 mol%) and TFP (10 mol%). Subsequently, 2-naphthalene acetylene **2w** (0.4 mmol, 2.0 eq.), Piv_2O (1.4 eq.) and dioxane (2 mL) was added under N_2. The reaction mixture was allowed to react at 160 °C for 12 h. After completion of the reaction, the reaction mixture was then cooled to room temperature, and then the reaction mixture was transferred to a round-bottomed bottle and concentrated under reduced pressure. The crude residue was purified by flash chromatography on silica gel (PE) to give the desired **3w** as a yellow solid in 69% yield (45.5 mg). ^1H NMR (400 MHz, $CDCl_3$) δ 8.24 – 8.23 (m, 2H), 8.03 – 8.02 (m, 1H), 8.01 – 7.96 (m, 3H), 7.96 – 7.87 (m, 3H), 7.64 – 7.62 (m, 2H), 7.59 – 7.55 (m, 4H), 7.52 – 7.47 (m, 3H); ^{13}C NMR (100 MHz, $CDCl_3$) δ 140.9 (s), 140.7 (s), 138.1 (s), 137.8 (s), 134.2 (s), 133.7 (s), 132.7 (s), 130. 9 (s), 130.1 (s), 128.6 (s), 128.5 (s), 128.3 (s), 128.2 (s), 127.6 (s), 127.4 (s), 126.8 (s), 126.3 (s), 126.3 (s), 126.2 (s), 126.1 (s), 126.0 (s), 125.9 (s), 125.7 (s), 125.6 (s). This compound is known.[2]

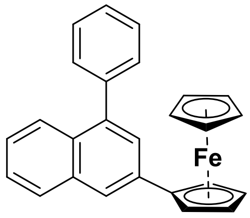

3-(ferrocenyl)-1-phenylnaphthalene (3x): The representative general procedure mentioned above was followed. An oven dried 25 mL Schlenk tube was charged with carboxylic acid **1a** (0.2 mmol), DMAP (0.2 mmol, 1.0 eq.), LiBr (0.1 mmol, 0.5 eq.), $PdCl_2$ (5 mol%) and TFP (10 mol%). Subsequently, ferrocenylethyne **2x** (0.4 mmol, 2.0 eq.), Piv_2O (1.4 eq.) and dioxane (2 mL) was added under N_2. The reaction mixture was allowed to react at 160 °C for 12 h. After completion of the reaction, the reaction

mixture was then cooled to room temperature, and then the reaction mixture was transferred to a round-bottomed bottle and concentrated under reduced pressure. The crude residue was purified by flash chromatography on silica gel (PE) to give the desired **3x** as a yellow solid in 61% yield (47.3 mg). mp: 134-135 °C; ^{1}H NMR (400 MHz, $CDCl_3$) δ 7.96 – 7.93 (m, 3H), 7.64 – 7. 63 (m, 3H), 7.56 – 7.46 (m, 6H), 4.88 – 4.86 (m, 2H), 4.47 – 7.45 (m, 2H), 4.17 – 4.15 (m, 5H); ^{13}C NMR (100 MHz, $CDCl_3$) δ 140.7 (s), 140.1 (s), 136.4 (s), 134.1 (s), 130.3 (s), 130.1 (s), 128.3 (s), 127.9 (s), 127.3 (s), 126.3 (s), 126.1 (s), 125.9 (s), 125.3 (s), 123.3 (s), 85.1 (s), 69.6 (s), 69.2 (s), 66.7 (s). HRMS (APCI-POS) m/z: $[M+H]^+$ calcd. for $C_{26}H_{21}Fe^+$: 389.0987, found: 389.0978.

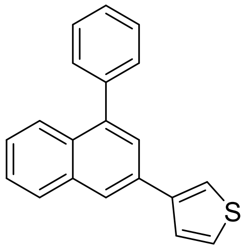

3-(4-phenylnaphthalen-2-yl)thiophene (3y): The representative general procedure mentioned above was followed. An oven dried 25 mL Schlenk tube was charged with carboxylic acid **1a** (0.2 mmol), DMAP (0.2 mmol, 1.0 eq.), LiBr (0.1 mmol, 0.5 eq.), $PdCl_2$ (5 mol%) and TFP (10 mol%). Subsequently, 3-thiophene acetylene **2y** (0.4 mmol, 2.0 eq.), Piv_2O (1.4 eq.) and dioxane (2 mL) was added under N_2. The reaction mixture was allowed to react at 160 °C for 12 h. After completion of the reaction, the reaction mixture was then cooled to room temperature, and then the reaction mixture was transferred to a round-bottomed bottle and concentrated under reduced pressure. The crude residue was purified by flash chromatography on silica gel (PE) to give the desired **3y** as yellow oil in 68% yield (38.9 mg). ^{1}H NMR (400 MHz, $CDCl_3$) δ 8.08 (s, 1H), 7.95 – 7.93 (m, 1H), 7. 90 – 7.88 (m, 1H), 7.73 – 7.72 (m, 1H), 7.61 (dd, J = 2.8, 1.2 Hz, 1H), 7.59 – 7.56 (m, 2H), 7.55 – 7.53 (m, 3H), 7.52 – 7.46 (m, 2H), 7.45 – 7.41 (m, 2H); ^{13}C NMR (100 MHz, $CDCl_3$) δ 142.0 (s), 140.83 (s), 140. 6 (s), 134.2 (s), 132.6 (s), 130.8 (s), 130.0 (s), 128.4 (s), 128.3 (s), 127.4 (s), 126.4 (s), 126.4 (s), 126.3 (s), 126.1 (s), 125.9 (s), 124.4 (s), 120.8 (s). HRMS (APCI-POS) m/z: $[M+H]^+$ calcd. for $C_{20}H_{15}S^+$: 287.0889, found: 287.0880.

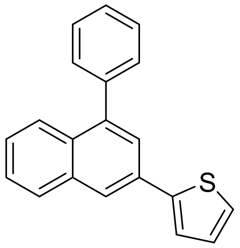

2-(4-phenylnaphthalen-2-yl)thiophene (3z): The representative general procedure mentioned above was followed. An oven dried 25 mL Schlenk tube was charged with carboxylic acid **1a** (0.2 mmol), DMAP (0.2 mmol, 1.0 eq.), LiBr (0.1 mmol, 0.5 eq.), $PdCl_2$ (5 mol%) and TFP (10 mol%). Subsequently, 2-thiophene acetylene **2z** (0.4 mmol, 2.0 eq.), Piv_2O (1.4 eq.) and dioxane (2 mL) was added under N_2. The reaction mixture was allowed to react at 160 °C for 12 h. After completion of the reaction, the reaction mixture was then cooled to room temperature, and then the reaction mixture was transferred to a round-bottomed bottle and concentrated under reduced pressure. The crude residue was purified by flash chromatography on silica gel (PE) to give the desired **3z** as yellow oil in 53% yield (30.3 mg). ^{1}H NMR (400 MHz, $CDCl_3$) δ 8.10 (s, 1H), 7.95 – 7.93 (m, 1H), 7.90 – 7.87 (m, 1H), 7.74 – 7.43 (m, 1H), 7.57 – 7.54 (m, 4H), 7.52 – 7.47 (m, 3H), 7.45 – 7.40 (m, 1H), 7.35 (dd, J = 5.2, 0.4 Hz, 1H), 7.15 (dd, J = 5.2, 3.6 Hz, 1H); ^{13}C NMR (100 MHz, $CDCl_3$) δ 144.2 (s), 140.9 (s), 140.3 (s), 134.1 (s), 131.3 (s), 131.0 (s), 130.0 (s), 128.4 (s), 128.3 (s), 128.1 (s), 127. 5 (s), 126.4 (s), 126.1 (s), 125.9 (s), 125.4 (s), 125.1 (s), 123.8 (s), 123.6 (s). HRMS (APCI-POS) m/z: $[M+H]^+$ calcd. for $C_{20}H_{15}S^+$: 287.0889, found: 287.0889.

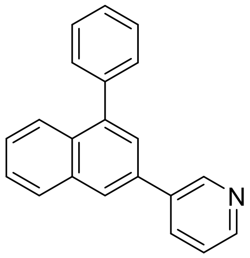

3-(4-phenylnaphthalen-2-yl)pyridine (3aa): The representative general procedure mentioned above was followed. An oven dried 25 mL Schlenk tube was charged with carboxylic acid **1a** (0.2 mmol), DMAP (0.2 mmol, 1.0 eq.), LiBr (0.1 mmol, 0.5 eq.), $PdCl_2$ (5 mol%) and TFP (10 mol%). Subsequently, 3-pyridine acetylene **2aa** (0.4 mmol, 2.0 eq.), Piv_2O (1.4 eq.) and dioxane (2 mL) was added under N_2. The reaction mixture was allowed to react at 160 °C for 12 h. After completion of the reaction, the reaction mixture was then cooled to room temperature, and then the reaction mixture was

transferred to a round-bottomed bottle and concentrated under reduced pressure. The crude residue was purified by flash chromatography on silica gel (PE/EA=20/1) to give the desired **3aa** as yellow oil in 66% yield (37.0 mg). ^{1}H NMR (400 MHz, $CDCl_3$) δ 9.05 (s, 1H), 8.65 (s, 1H), 8.07 (s, 1H), 8.04 – 8.02 (m, 1H), 7.99 – 7.97 (m, 1H), 7.94 – 7.93 (m, 1H), 7.68 – 7.67 (m, 1H), 7.57 – 7.54 (m, 3H), 7.53– 7.51 (m, 2H), 7.49 – 7.44 (m, 2H), 7.42 (dd, *J* = 7.6, 4.8 Hz, 1H); ^{13}C NMR (100 MHz, $CDCl_3$) δ 148.4 (s), 141.4 (s), 140.3 (s), 136.4 (s), 134.6 (s), 134.5 (s), 134.0 (s), 131.1 (s), 129.9 (s), 128.6 (s), 128.4 (s), 127.5 (s), 126.6 (s), 126.5 (s), 126.0 (s), 125.9 (s), 125.7 (s), 123.7 (s). HRMS (APCI-POS) m/z: $[M+H]^+$ calcd. for $C_{21}H_{16}N^+$: 282.1278, found: 282.1275.

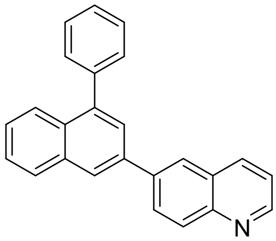

6-(4-phenylnaphthalen-2-yl)quinoline (3ab): The representative general procedure mentioned above was followed. An oven dried 25 mL Schlenk tube was charged with carboxylic acid **1a** (0.2 mmol), DMAP (0.2 mmol, 1.0 eq.), LiBr (0.1 mmol, 0.5 eq.), $PdCl_2$ (5 mol%) and TFP (10 mol%). Subsequently, 6-ethynylquinoline **2ab** (0.4 mmol, 2.0 eq.), Piv_2O (1.4 eq.) and dioxane (2 mL) was added under N_2. The reaction mixture was allowed to react at 160 °C for 12 h. After completion of the reaction, the reaction mixture was then cooled to room temperature, and then the reaction mixture was transferred to a round-bottomed bottle and concentrated under reduced pressure. The crude residue was purified by flash chromatography on silica gel (PE/EA=20/1) to give the desired **3ab** as yellow oil in 69% yield (45.7 mg). ^{1}H NMR (400 MHz, $CDCl_3$) δ 8.95 (s, 1H), 8.24 – 8.19 (m, 3H), 8.17 – 8.16 (m, 2H), 8.02 – 7.94 (m, 2H), 7.84 (d, J = 1.6 Hz, 1H), 7.63 – 7.52 (m, 5H), 7.51 – 7.41 (m, 3H); ^{13}C NMR (100 MHz, $CDCl_3$) δ 150.35 (s), 147.6 (s), 141.2 (s), 140.5 (s), 138.9 (s), 136.9 (s), 136.3 (s), 134.1 (s), 131.0 (s), 130.0 (s),129.9 (s), 129.4 (s), 128.6 (s),128,5 (s),128.3 (s), 127.5 (s), 126.5 (s), 126.4 (s), 126.0 (s), 125.9 (s),125.8 (s),121.54 (s). HRMS (APCI-POS) m/z: $[M+H]^+$ calcd. for $C_{25}H_{18}N^+$: 332.1434, found: 332.1434.

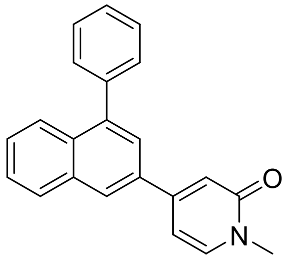

1-methyl-4-(4-phenylnaphthalen-2-yl)pyridin-2(1H)-one (3ac): The representative general procedure mentioned above was followed. An oven dried 25 mL Schlenk tube was charged with carboxylic acid **1a** (0.2 mmol), DMAP (0.2 mmol, 1.0 eq.), LiBr (0.1 mmol, 0.5 eq.), $PdCl_2$ (5 mol%) and TFP (10 mol%). Subsequently, 4-ethynyl-1-methylpyridin-2(*1H*)-one **2ac** (0.4 mmol, 2.0 eq.), Piv_2O (1.4 eq.) and dioxane (2 mL) was added under N_2. The reaction mixture was allowed to react at 160 °C for 12 h. After completion of the reaction, the reaction mixture was then cooled to room temperature, and then the reaction mixture was transferred to a round-bottomed bottle and concentrated under reduced pressure. The crude residue was purified by flash chromatography on silica gel (PE/EA=10/1) to give the desired **3ac** as a yellow solid in 56% yield (34.8 mg). mp: 88-89 °C ; ^1H NMR (400 MHz, $CDCl_3$) δ 8.07 (s, 1H), 7.97 – 7.90 (m, 2H), 7.63 (d, *J* = 1.6 Hz, 1H), 7.57 – 7.51 (m, 3H), 7.51 – 7.45 (m, 4H), 7.39 (d, *J* = 6.8 Hz, 1H), 7.02 (d, *J* = 1.6 Hz, 1H), 6.63 (dd, *J* = 7.2, 1.6 Hz, 1H), 3.61 (s, 3H); ^{13}C NMR (100 MHz, $CDCl_3$) δ 163.2 (s), 151.8 (s), 141.3 (s), 140.1 (s), 138.2 (s), 134.0 (s), 133.7 (s), 131. 9 (s), 129.9 (s), 128.9 (s), 128.4 (s), 127.6 (s), 127.1 (s), 126.6 (s), 125.9 (s), 125.8 (s), 125.1 (s), 117.1 (s), 105.8 (s), 37.4 (s). HRMS (APCI-POS) m/z: $[M+H]^+$ calcd. for $C_{22}H_{18}NO^+$: 312.1383, found: 312.1385.

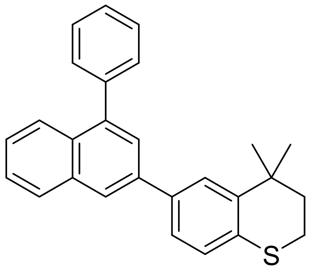

4,4-dimethyl-6-(4-phenylnaphthalen-2-yl)thiochromane (3ad): The representative general procedure mentioned above was followed. An oven dried 25 mL Schlenk tube was charged with carboxylic acid **1a** (0.2 mmol), DMAP (0.2 mmol, 1.0 eq.), LiBr (0.1 mmol, 0.5 eq.), $PdCl_2$ (5 mol%) and TFP (10 mol%). Subsequently, 6-ethynyl-4,4-dimethylthiochromane **2ad** (0.4 mmol, 2.0 eq.), Piv_2O (1.4 eq.) and dioxane (2 mL) was added under N_2. The reaction mixture was allowed to react at

160 °C for 12 h. After completion of the reaction, the reaction mixture was then cooled to room temperature, and then the reaction mixture was transferred to a round-bottomed bottle and concentrated under reduced pressure. The crude residue was purified by flash chromatography on silica gel (PE) to give the desired **3ad** as yellow oil in 77% yield (58.5 mg). ^{1}H NMR (400 MHz, CDCl$_3$) δ 8.03 (s, 1H), 7.98 – 7.96 (m, 1H), 7.91 – 7.89 (m, 1H), 7.76 (d, J = 1.6 Hz, 1H), 7.69 (d, J = 2.0 Hz, 1H), 7.60 – 7.56 (m, 2H), 7.56 – 7.53 (m, 2H), 7.53 – 7.47 (m, 2H), 7.47 – 7.41 (m, 2H), 7.22 (d, J = 8.4 Hz, 1H), 3.12 – 3.07 (m, 2H), 2.06 – 2.01 (m, 2H), 1.44 (s, 6H); ^{13}C NMR (100 MHz, CDCl$_3$) δ 142.4 (s), 140.8 (s), 140.7 (s), 137.9 (s), 136.7 (s), 134.2 (s), 131.4 (s), 130.7 (s), 130.1 (s), 128.4 (s), 128.3 (s), 127.3 (s), 127.1 (s), 126.4 (s), 126.2 (s), 125.9 (s), 125.4 (s), 125.1 (s), 124.8 (s), 37.7 (s), 33.2 (s), 30.3 (s), 23.1 (s). HRMS (APCI-POS) m/z: $[M+H]^+$ calcd. for $C_{27}H_{25}S^+$: 381.1671, found: 381.1677.

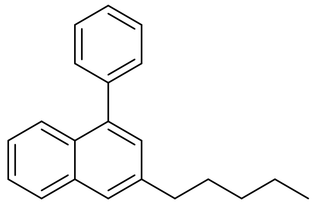

3-pentyl-1-phenylnaphthalene (3ae): The representative general procedure mentioned above was followed. An oven dried 25 mL Schlenk tube was charged with carboxylic acid **1a** (0.2 mmol), DMAP (0.2 mmol, 1.0 eq.), LiBr (0.1 mmol, 0.5 eq.), $PdCl_2$ (5 mol%) and TFP (10 mol%). Subsequently, 1-heptyne **2ae** (0.4 mmol, 2.0 eq.), Piv_2O (1.4 eq.) and dioxane (2 mL) was added under N_2. The reaction mixture was allowed to react at 160 °C for 12 h. After completion of the reaction, the reaction mixture was then cooled to room temperature, and then the reaction mixture was transferred to a round-bottomed bottle and concentrated under reduced pressure. The crude residue was purified by flash chromatography on silica gel (PE) to give the desired **3ae** as yellow oil in 53% yield (29.1 mg). ^{1}H NMR (400 MHz, CDCl$_3$) δ 7.87 (t, J = 9.2 Hz, 2H), 7.66 (s, 1H), 7.54 – 7.52 (m, 3H), 7.51 – 7.45 (m, 2H), 7.45 – 7.35 (m, 2H), 7.32 – 7.31 (m, 1H), 2.89 – 2.76 (m, 2H), 1.81 – 1.73 (m, 2H), 1.42– 1.38 (dm, 4H), 0.93 (t, J = 6.8 Hz, 3H); ^{13}C NMR (100 MHz, CDCl$_3$) δ 140.9 (s), 140.0 (s), 139.9 (s), 134.0 (s), 130.1 (s), 128.6 (s), 128.2 (s), 127.8 (s), 127.1 (s), 126.6 (s), 126.0 (s), 125.8 (s), 125.7 (s), 125.2 (s), 36.1 (s), 31.59 (s), 31.0 (s), 22.6 (s), 14.1 (s). HRMS (APCI-POS) m/z: $[M]^+$ calcd. for $C_{21}H_{21}^+$: 273.1638, found: 273.1635.

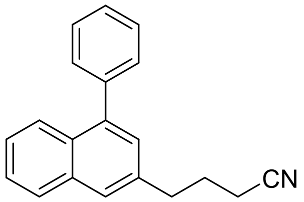

4-(4-phenylnaphthalen-2-yl)butanenitrile (3af): The representative general procedure mentioned above was followed. An oven dried 25 mL Schlenk tube was charged with carboxylic acid **1a** (0.2 mmol), DMAP (0.2 mmol, 1.0 eq.), LiBr (0.1 mmol, 0.5 eq.), $PdCl_2$ (5 mol%) and TFP (10 mol%). Subsequently, hex-5-ynenitrile **2af** (0.4 mmol, 2.0 eq.), Piv_2O (1.4 eq.) and dioxane (2 mL) was added under N_2. The reaction mixture was allowed to react at 160 °C for 12 h. After completion of the reaction, the reaction mixture was then cooled to room temperature, and then the reaction mixture was transferred to a round-bottomed bottle and concentrated under reduced pressure. The crude residue was purified by flash chromatography on silica gel (PE/EA=20/1) to give the desired **3af** as yellow oil in 69% yield (37.3 mg). ^{1}H NMR (400 MHz, $CDCl_3$) δ 7.90 – 7.86 (m, 2H), 7.68 (s, 1H), 7.52 – 7.51 (m, 2H), 7.50 – 7.48 (m, 3H), 7.29 – 7.28 (m, 1H), 2.99 (t, J = 7.4 Hz, 2H), 2.39 (t, J = 7.0 Hz, 2H), 2.15 – 2.06 (m, 2H); ^{13}C NMR (100 MHz, $CDCl_3$) δ 140.8 (s), 140.4 (s), 136.6 (s), 133.9 (s), 130.4 (s), 129.9 (s), 128.3 (s), 127.8 (s), 127.3 (s), 126.5 (s), 126.1 (s), 125.8 (s), 125.7 (s), 119. 5 (s), 34.4 (s), 26.7 (s), 16.4 (s). HRMS (APCI-POS) m/z: $[M+H]^+$ calcd. for $C_{20}H_{18}N^+$: 272.1434, found: 272.1430.

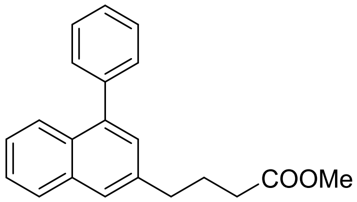

methyl 4-(4-phenylnaphthalen-2-yl)butanoate (3ag): The representative general procedure mentioned above was followed. An oven dried 25 mL Schlenk tube was charged with carboxylic acid **1a** (0.2 mmol), DMAP (0.2 mmol, 1.0 eq.), LiBr (0.1 mmol, 0.5 eq.), $PdCl_2$ (5 mol%) and TFP (10 mol%). Subsequently, methyl hex-5-ynoate **2ag** (0.4 mmol, 2.0 eq.), Piv_2O (1.4 eq.) and dioxane (2 mL) was added under N_2. The reaction mixture was allowed to react at 160 °C for 12 h. After completion of the reaction, the reaction mixture was then cooled to room temperature, and then the reaction mixture was transferred to a round-bottomed bottle and concentrated under reduced pressure. The crude residue was purified by flash chromatography on silica gel (PE/EA=20/1) to give the desired **3ag** as yellow oil in 57% yield (34.6 mg). ^{1}H NMR (400 MHz, $CDCl_3$) δ 7.89 – 7.84 (t, 2H), 7.65 (s, 1H), 7.52 – 7.48 (m, 4H), 7.48 – 7. 41 (m, 2H), 7.40 – 7. 30 (m, 1H), 7.30 (d, J = 2.0 Hz, 1H), 3.68 (s, 3H), 2.87 (d, J = 8.4

Hz, 2H), 2.41 (d, J = 7.4 Hz, 2H), 2.14 – 2.06 (m, 2H); ^{13}C NMR (100 MHz, $CDCl_3$) δ 173.9 (s), 140.7 (s), 140.3 (s), 138.3 (s), 133.9 (s), 130.2 (s), 130.0 (s), 128.3 (s), 128.2 (s), 127.8 (s), 127.2 (s), 126.3 (s), 125.8 (s), 125.8 (s), 125.4 (s), 51.5 (s), 35.2 (s), 33.2 (s), 26.3 (s). HRMS (APCI-POS) m/z: $[M+H]^+$ calcd. for $C_{21}H_{21}O_2^+$: 305.1527, found: 305.1527.

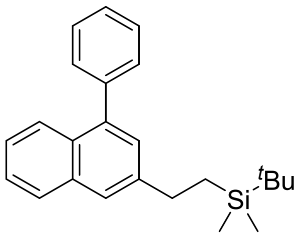

***tert*-butyldimethyl(2-(4-phenylnaphthalen-2-yl)ethyl)silane (3ah):** The representative general procedure mentioned above was followed. An oven dried 25 mL Schlenk tube was charged with carboxylic acid **1a** (0.2 mmol), DMAP (0.2 mmol, 1.0 eq.), LiI (0.1 mmol, 0.5 eq.), $PdCl_2$ (5 mol%) and TFP (10 mol%). Subsequently, methyl but-3-yn-1-yl(*tert*-butyl)dimethylsilane **2ah** (0.4 mmol, 2.0 eq.), Piv_2O (1.4 eq.) and dioxane (2 mL) was added under N_2. The reaction mixture was allowed to react at 160 °C for 12 h. After completion of the reaction, the reaction mixture was then cooled to room temperature, and then the reaction mixture was transferred to a round-bottomed bottle and concentrated under reduced pressure. The crude residue was purified by flash chromatography on silica gel (PE) to give the desired **3ah** as yellow oil in 76% yield (52.6 mg). ^{1}H NMR (400 MHz, $CDCl_3$) δ 7.88 – 7. 84 (m, 1H), , 7.69 (s, 1H), 7.51 – 7.49 (m, 4H), 7.47 – 7. 45 (m, 2H), 7.39 – 7. 36 (m, 1H), 7.32 (d, J = 1.6 Hz, 1H), 3.94(t, J = 7.0 Hz, 2H), 3.03 (t, J = 7.0 Hz, 2H), 0.88 (s, 9H), 0.00 (s, 6H); ^{13}C NMR (100 MHz, $CDCl_3$) δ 140.8 (s), 140.1 (s), 136.2 (s), 133.9 (s), 130.3 (s), 130.0 (s), 128.9 (s), 128.2 (s), 127.8 (s), 127.2 (s), 127.1 (s), 125.8 (s), 125.8 (s), 125.4 (s), 64.4 (s), 39.7 (s), 25.9 (s), 18.3 (s), -5.4 (s). HRMS (APCI-POS) m/z: $[M+H]^+$ calcd. for $C_{24}H_{31}OSi^+$: 363.2139, found: 363.2139.

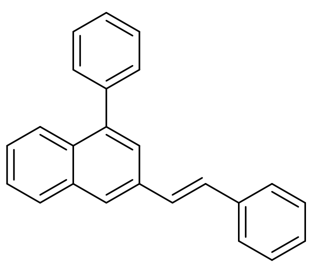

(E)-1-phenyl-3-styrylnaphthalene (3ai): The representative general procedure mentioned above was followed. An oven dried 25 mL Schlenk tube was charged with carboxylic acid **1a** (0.2 mmol), DMAP (0.2 mmol, 1.0 eq.), LiBr (0.1 mmol, 0.5 eq.), $PdCl_2$ (5 mol%) and TFP (10 mol%).

Subsequently, methyl but-(E)-but-1-en-3-yn-1-ylbenzene **2ai** (0.4 mmol, 2.0 eq.), Piv_2O (1.4 eq.) and dioxane (2 mL) was added under N_2. The reaction mixture was allowed to react at 160 °C for 12 h. After completion of the reaction, the reaction mixture was then cooled to room temperature, and then the reaction mixture was transferred to a round-bottomed bottle and concentrated under reduced pressure. The crude residue was purified by flash chromatography on silica gel (PE) to give the desired **3ai** as yellow oil in 80% yield (48.9 mg). ^1H NMR (400 MHz, $CDCl_3$) δ 8.09 (s, 1H), 8.00 – 7.97 (m, 1H), 7.95 – 7.93 (m, 1H), 7.79 – 7.77 (m, 2H), 7.74 (d, *J* = 1.2 Hz, 1H), 7.59 – 7.56 (m, 3H), 7.54 – 7.52 (m, 3H), 7.51 – 7.47 (m, 3H), 7.46 – 7.37 (m, 3H); ^{13}C NMR (100 MHz, $CDCl_3$) δ 140.9 (s), 140.8 (s), 140.7 (s), 137.9 (s), 134.1 (s), 130.82 (s), 130.1 (s), 128.9 (s), 128.6 (s), 128.3 (s), 127.4 (s), 127.4 (s), 126. 7 (s), 126.2 (s), 126.1 (s), 125. 9 (s), 125.4 (s). HRMS (APCI-POS) m/z: $[M+H]^+$ calcd. for $C_{24}H_{19}^+$: 307.1482, found: 307.1482.

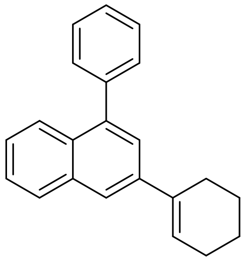

3-(cyclohex-1-en-1-yl)-1-phenylnaphthalene (3aj): The representative general procedure mentioned above was followed. An oven dried 25 mL Schlenk tube was charged with carboxylic acid **1a** (0.2 mmol), DMAP (0.2 mmol, 1.0 eq.), LiBr (0.1 mmol, 0.5 eq.), $PdCl_2$ (5 mol%) and TFP (10 mol%). Subsequently, 1-cyclohexene acetylene **2aj** (0.4 mmol, 2.0 eq.), Piv_2O (1.4 eq.) and dioxane (2 mL) was added under N_2. The reaction mixture was allowed to react at 160 °C for 12 h. After completion of the reaction, the reaction mixture was then cooled to room temperature, and then the reaction mixture was transferred to a round-bottomed bottle and concentrated under reduced pressure. The crude residue was purified by flash chromatography on silica gel (PE) to give the desired **3aj** as yellow oil in 57% yield (32.4 mg). ^1H NMR (400 MHz, $CDCl_3$) δ 7.89 – 7.87 (m, 1H), 7.85 – 7.83 (m, 1H), 7.81 (s, 1H), 7.57 (d, *J* = 2.0 Hz, 1H), 7.55 – 7.49 (m, 4H), 7.49 – 7.44 (m, 2H), 7.40 – 7.36 (m, 1H), 6.36 – 6.34 (m, 1H), 2.60 – 2.56 (m, 2H), 2.31 – 2.25 (m, 2H), 1.89 – 1.83 (m, 2H), 1.75 – 1.69 (m, 2H); ^{13}C NMR (100 MHz, $CDCl_3$) δ 141.0 (s), 139.90 (s), 139.20 (s), 136.2 (s), 133.9 (s), 130.6 (s), 130.0 (s), 128.4 (s), 128.2 (s), 127.2 (s), 125.9 (s), 125.7 (s), 125.5 (s), 124.9 (s), 122. 8 (s), 27.4 (s),

26.0 (s), 23.1 (s), 22.2 (s). HRMS (APCI-POS) m/z: $[M+H]^+$ calcd. for $C_{22}H_{21}^+$: 285.1638, found: 285.1637.

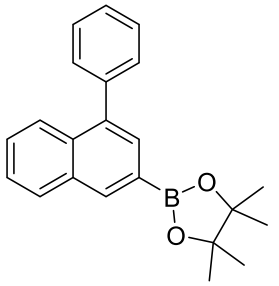

4,4,5,5-tetramethyl-2-(4-phenylnaphthalen-2-yl)-1,3,2-dioxaborolane (3ak): The representative general procedure mentioned above was followed. An oven dried 25 mL Schlenk tube was charged with carboxylic acid **1a** (0.2 mmol), DMAP (0.2 mmol, 1.0 eq.), LiBr (0.1 mmol, 0.5 eq.), $PdCl_2$ (5 mol%) and TFP (10 mol%). Subsequently, 2-ethynyl-4,4,5,5-tetramethyl-1,3,2-dioxaborolane **2ak** (0.8 mmol, 4.0 eq.), Piv_2O (1.4 eq.) and dioxane (2 mL) was added under N_2. The reaction mixture was allowed to react at 160 °C for 12 h. After completion of the reaction, the reaction mixture was then cooled to room temperature, and then the reaction mixture was transferred to a round-bottomed bottle and concentrated under reduced pressure. The crude residue was purified by flash chromatography on silica gel (PE) to give the desired **3ak** as a white oil in 38% yield (25.1 mg). ^1H NMR (400 MHz, $CDCl_3$) δ 8.40 (s, 1H), 8.00 – 7.89 (m, 2H), 7.82 (s, 1H), 7.54 – 7.50 (m, 3H), 7.50 – 7.48 (m, 2H), 7.48 – 7.40 (m, 2H), 1.40 (s, 12H); ^{13}C NMR (100 MHz, $CDCl_3$) δ 140.7 (s), 139.3 (s), 135.9 (s), 133.2 (s), 133.1 (s), 131.4 (s), 130.1 (s), 129.0 (s), 128.1 (s), 127.13 (s), 127.1 (s), 125.9 (s), 125.7 (s), 83.9 (s), 24.9 (s). This compound is known.[4]

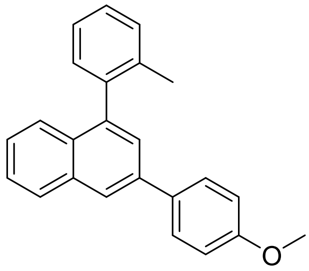

3-(4-methoxyphenyl)-1-(o-tolyl)naphthalene (3al): The representative general procedure mentioned above was followed. An oven dried 25 mL Schlenk tube was charged with 2-(1-(o-tolyl)vinyl)benzoic acid **1b** (0.2 mmol), DMAP (0.2 mmol, 1.0 eq.), LiBr (0.1 mmol, 0.5 eq.), $PdCl_2$ (5 mol%) and TFP (10 mol%). Subsequently, 4-methoxyphenylene **2e** (0.4 mmol, 2.0 eq.), Piv_2O (1.4 eq.) and dioxane (2 mL) was added under N_2. The reaction mixture was allowed to react at 160 °C for 12 h.

After completion of the reaction, the reaction mixture was then cooled to room temperature, and then the reaction mixture was transferred to a round-bottomed bottle and concentrated under reduced pressure. The crude residue was purified by flash chromatography on silica gel (PE) to give the desired **3al** as yellow oil in 76% yield (49.2 mg). ^{1}H NMR (400 MHz, $CDCl_3$) δ 7.92 (s, 1H), 7.83 (d, *J* = 8.4 Hz, 1H), 7.61 – 7.57 (m, 2H), 7.51 (d, *J* = 2.0 Hz, 1H), 7.39 – 7.34 (m, 2H), 7.29 – 7.24 (m, 3H), 7.22 – 7.19 (m, 2H), 6.93 – 6.89 (m, 2H), 3.75 (s, 3H), 1.97 (s, 3H); ^{13}C NMR (100 MHz, $CDCl_3$) δ 159.2 (s), 140.3 (s), 140.2 (s), 137.5 (s), 136.8 (s), 133.9 (s), 133.3 (s), 130.9 (s), 130.4 (s), 129.9 (s), 128.4 (s), 127.6 (s), 126.1 (s), 125.9 (s), 125.7 (s), 125.6 (s), 124.3 (s), 114.3 (s), 55.3 (s), 20.1 (s). HRMS (APCI-POS) m/z: $[M+H]^+$ calcd. for $C_{24}H_{21}O^+$: 325.1587, found: 325.1588.

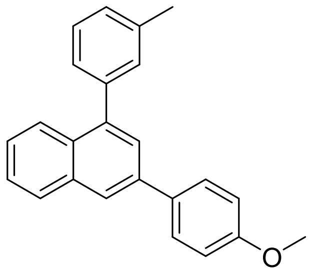

3-(4-methoxyphenyl)-1-(m-tolyl)naphthalene (3am): The representative general procedure mentioned above was followed. An oven dried 25 mL Schlenk tube was charged with 2-(1-(m-tolyl)vinyl)benzoic acid **1c** (0.2 mmol), DMAP (0.2 mmol, 1.0 eq.), LiBr (0.1 mmol, 0.5 eq.), $PdCl_2$ (5 mol%) and TFP (10 mol%). Subsequently, 4-methoxyphenylene **2e** (0.4 mmol, 2.0 eq.), Piv_2O (1.4 eq.) and dioxane (2 mL) was added under N_2. The reaction mixture was allowed to react at 160 °C for 12 h. After completion of the reaction, the reaction mixture was then cooled to room temperature, and then the reaction mixture was transferred to a round-bottomed bottle and concentrated under reduced pressure. The crude residue was purified by flash chromatography on silica gel (PE) to give the desired **3am** as yellow oil in 55% yield (35.6 mg). ^{1}H NMR (400 MHz, $CDCl_3$) δ 8.02 (d, *J* = 1.2 Hz, 1H), 7.99 – 7.89 (m, 2H), 7.74 – 7.68 (m, 3H), 7.54 – 7.49 (m, 1H), 7.45 – 7.40 (m, 2H), 7.39 – 7.36 (m, 2H), 7.29 (d, *J* = 7.6 Hz, 1H), 7.07 – 7.02 (m, 2H), 3.88 (s, 3H), 2.48 (s, 3H); ^{13}C NMR (100 MHz, $CDCl_3$) δ 159.3 (s), 140.8 (s), 140.7 (s), 137.9 (s), 137.5 (s), 134.2 (s), 133.4 (s), 130.8 (s), 130.6 (s), 128.4 (s), 128.1 (s), 128.0 (s), 127.2 (s), 126.4 (s), 126.1 (s), 125.9 (s), 125.7 (s), 124.5 (s), 114.3 (s), 55.3 (s), 21.5 (s). HRMS (APCI-POS) m/z: $[M+H]^+$ calcd. for $C_{24}H_{21}O^+$: 325.1587, found: 325.1587.

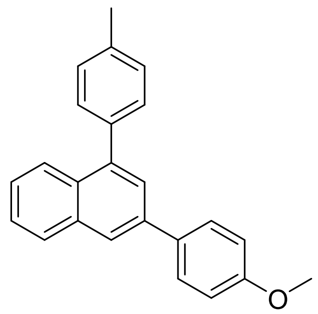

3-(4-methoxyphenyl)-1-(p-tolyl)naphthalene (3an): The representative general procedure mentioned above was followed. An oven dried 25 mL Schlenk tube was charged with 2-(1-(p-tolyl)vinyl)benzoic acid **1d** (0.2 mmol), DMAP (0.2 mmol, 1.0 eq.), LiBr (0.1 mmol, 0.5 eq.), $PdCl_2$ (5 mol%) and TFP (10 mol%). Subsequently, 4-methoxyphenylene **2e** (0.4 mmol, 2.0 eq.), Piv_2O (1.4 eq.) and dioxane (2 mL) was added under N_2. The reaction mixture was allowed to react at 160 °C for 12 h. After completion of the reaction, the reaction mixture was then cooled to room temperature, and then the reaction mixture was transferred to a round-bottomed bottle and concentrated under reduced pressure. The crude residue was purified by flash chromatography on silica gel (PE) to give the desired **3an** as yellow oil in 75% yield (48.6 mg). ^{1}H NMR (400 MHz, $CDCl_3$) δ 8.02 (d, J = 1.6 Hz, 1H), 7.98 – 7.91 (m, 2H), 7.73 – 7.69 (m, 3H), 7.54 – 7.48 (m, 2H), 7.47 – 7.40 (m, 2H), 7.35 (d, J = 8.0 Hz, 2H), 7.06 – 7.01 (m, 2H), 3.88 (s, 3H), 2.49 (s, 3H); ^{13}C NMR (100 MHz, $CDCl_3$) δ 159.2 (s), 140.7 (s), 137.8 (s), 137.6 (s), 137.0 (s), 134.2 (s), 133.4 (s), 130.6 (s), 129.9 (s), 128.9 (s), 128.4 (s), 126. 5 (s), 126.1 (s), 125.9 (s), 125.7 (s), 124.4 (s), 114.2 (s), 55.3 (s), 21.2 (s). This compound is known.[5]

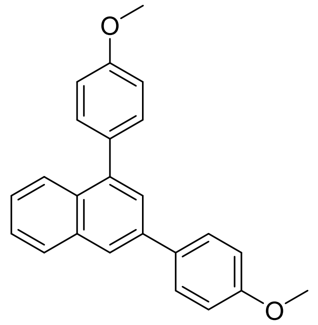

1,3-bis(4-methoxyphenyl)naphthalenee (3ao): The representative general procedure mentioned above was followed. An oven dried 25 mL Schlenk tube was charged with 2-(1-(4-methoxyphenyl)vinyl) benzoic acid **1e** (0.2 mmol), DMAP (0.2 mmol, 1.0 eq.), LiBr (0.1 mmol, 0.5 eq.), $PdCl_2$ (5 mol%) and TFP (10 mol%). Subsequently, 4-methoxyphenylene **2e** (0.4 mmol, 2.0 eq.), Piv_2O (1.4 eq.) and dioxane (2 mL) was added under N_2. The reaction mixture was allowed to react at 160 °C for 12 h. After completion of the reaction, the reaction mixture was then cooled to room

temperature, and then the reaction mixture was transferred to a round-bottomed bottle and concentrated under reduced pressure. The crude residue was purified by flash chromatography on silica gel (PE) to give the desired **3ao** as yellow oil in 57% yield (38.8 mg). ^{1}H NMR (400 MHz, $CDCl_3$) δ 7.97 (s, 1H), 7.92 – 7.89 (m, 2H), 7.68 – 7.69 (m, 2H), 7.65 (d, J = 1.6 Hz, 1H), 7.50 – 7.45 (m, 3H), 7.41 – 7.37 (m, 1H), 7.05– 7.03 (m, 2H), 7.05 – 7.03 (m, 2H), 3.88 (s, 3H), 3.85 (s, 3H); ^{13}C NMR (100 MHz, $CDCl_3$) δ 159.2 (s), 158.9 (s), 140.4 (s), 137.6 (s), 134.2 (s), 133.4 (s), 133.1 (s), 131.1 (s), 130.7 (s), 128.4 (s), 126.5 (s), 126.1 (s), 125. 9 (s), 125.7 (s), 124.3 (s), 114.3 (s), 113.7 (s), 55.3 (s).This compound is known.[2]

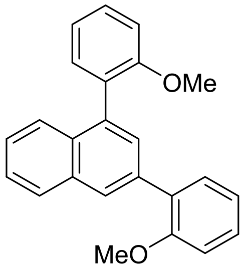

1,3-bis(2-methoxyphenyl)naphthalenee (3ap): The representative general procedure mentioned above was followed. An oven dried 25 mL Schlenk tube was charged with 2-(1-(2-methoxyphenyl)vinyl) benzoic acid **1f** (0.2 mmol), DMAP (0.2 mmol, 1.0 eq.), LiBr (0.1 mmol, 0.5 eq.), $PdCl_2$ (5 mol%) and TFP (10 mol%). Subsequently, 2-methoxyphenylene **2f** (0.4 mmol, 2.0 eq.), Piv_2O (1.4 eq.) and dioxane (2 mL) was added under N_2. The reaction mixture was allowed to react at 160 °C for 12 h. After completion of the reaction, the reaction mixture was then cooled to room temperature, and then the reaction mixture was transferred to a round-bottomed bottle and concentrated under reduced pressure. The crude residue was purified by flash chromatography on silica gel (PE) to give the desired **3ap** as yellow oil in 84% yield (57.1 mg). ^{1}H NMR (400 MHz, $CDCl_3$) δ 8.02 (s, 1H), 7.93 – 7.91 (m, 1H), 7.66 (d, J = 1.6 Hz, 1H), 7.62 – 7. 60 (m, 1H), 7.53 – 7.46 (m, 2H), 7.45– 7.42 (m, 1H), 7.41 – 7.33 (m, 3H), 7.13 – 7.05 (m, 3H), 7.04 – 7.02 (m, 1H), 3.84 (s, 3H), 3.73 (s, 3H); ^{13}C NMR (100 MHz, $CDCl_3$) δ 157.3 (s), 156.7 (s), 136.2 (s), 135.5 (s), 133. 5 (s), 132.1 (s), 131.2 (s), 131.1 (s), 130.6 (s), 129.6 (s), 129.5 (s), 128.9 (s), 128.6 (s), 128.4 (s), 127.9 (s), 126.2 (s), 125. 6 (s), 125.5 (s), 120.9 (s), 120.5 (s), 111.3 (s), 110.9 (s), 55.6 (s), 55.5 (s). HRMS (APCI-POS) m/z: $[M+H]^+$ calcd. for $C_{24}H_{21}O_2^+$: 341.1537, found: 341.1535.

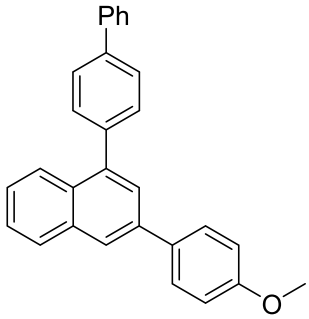

2-(1-([1,1'-biphenyl]-4-yl)vinyl)benzoic acid (3aq): The representative general procedure mentioned above was followed. An oven dried 25 mL Schlenk tube was charged with 2-(1-([1,1'-biphenyl]-4-yl)vinyl)benzoic acid **1g** (0.2 mmol), DMAP (0.2 mmol, 1.0 eq.), LiBr (0.1 mmol, 0.5 eq.), $PdCl_2$ (5 mol%) and TFP (10 mol%). Subsequently, 4-methoxyphenylene **2e** (0.4 mmol, 2.0 eq.), Piv_2O (1.4 eq.) and dioxane (2 mL) was added under N_2. The reaction mixture was allowed to react at 160 °C for 12 h. After completion of the reaction, the reaction mixture was then cooled to room temperature, and then the reaction mixture was transferred to a round-bottomed bottle and concentrated under reduced pressure. The crude residue was purified by flash chromatography on silica gel (PE) to give the desired **3aq** as yellow oil in 69% yield (53.2 mg). ^1H NMR (400 MHz, $CDCl_3$) δ 8.02 (d, J = 1.6 Hz, 1H), 7.97 (t, J = 7.6 Hz, 2H), 7.77 – 7.74 (m, 3H), 7.72 – 7.67 (m, 4H), 7.65 – 7.61 (m, 2H), 7.53 – 7.47 (m, 3H), 7.45 – 7.37 (m, 2H), 7.05 – 7.00 (m, 2H), 3.87 (s, 3H); ^{13}C NMR (100 MHz, $CDCl_3$) δ 159.3 (s), 140.8 (s), 140.3 (s), 140.2 (s), 139.7 (s), 137.6 (s), 134.3 (s), 133.4 (s), 130.5 (s), 128.9 (s), 128.5 (s), 128.4 (s), 127.4 (s), 127.1 (s), 127.0 (s), 126.5 (s), 126.2 (s), 125.7 (s), 124.7 (s), 114.3 (s), 55.4 (s). HRMS (APCI-POS) m/z: $[M+H]^+$ calcd. for $C_{29}H_{23}O^+$: 387.1744, found: 387.1744.

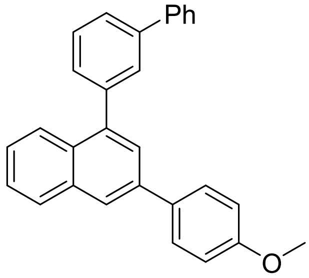

2-(1-([1,1'-biphenyl]-3-yl)vinyl)benzoic acid (3ar): The representative general procedure mentioned above was followed. An oven dried 25 mL Schlenk tube was charged with 2-(1-(2-methoxyphenyl)vinyl) benzoic acid **1h** (0.2 mmol), DMAP (0.2 mmol, 1.0 eq.), LiBr (0.1 mmol, 0.5 eq.), $PdCl_2$ (5 mol%) and TFP (10 mol%). Subsequently, 4-methoxyphenylene **2e** (0.4 mmol, 2.0 eq.), Piv_2O (1.4 eq.) and dioxane (2 mL) was added under N_2. The reaction mixture was allowed to react at 160 °C for 12 h. After completion of the reaction, the reaction mixture was then cooled to room

temperature, and then the reaction mixture was transferred to a round-bottomed bottle and concentrated under reduced pressure. The crude residue was purified by flash chromatography on silica gel (PE) to give the desired **3ar** as yellow oil in 73% yield (56.3 mg). ^{1}H NMR (400 MHz, $CDCl_3$) δ 8.08 (s, 1H), 8.03 – 7.99 (m, 2H), 7.86 (s, 1H), 7.81 (d, J = 1.6 Hz, 1H), 7.77 – 7.74 (m, 4H), 7.73 – 7.72 (m, 1H), 7.65 – 7.56 (m, 3H), 7.52 – 7.47 (m, 3H), 7.42 – 7.38 (m, 1H), 7.07 – 7.05 (m, 2H), 3.90 (s, 3H); ^{13}C NMR (100 MHz, $CDCl_3$) δ 159.3 (s), 141.2 (s), 140. 9 (s), 140.6 (s), 137.6 (s), 134.2 (s), 133.3 (s), 130.5 (s), 128.9 (s), 128.9 (s), 128.8 (s), 128.7 (s), 128.5 (s), 128.4 (s), 127.4 (s), 127.2 (s), 126.5 (s), 126.2 (s), 126.1 (s), 125.9 (s), 125.8 (s), 124.7 (s), 114.3 (s), 55.3 (s). HRMS (APCI-POS) m/z: $[M+H]^+$ calcd. for $C_{29}H_{23}O^+$: 387.1744, found: 387.1744.

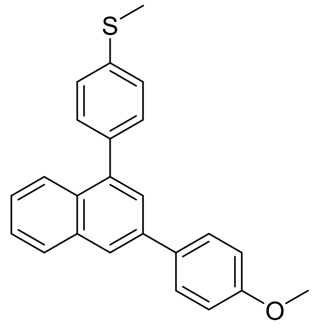

(4-(3-(4-methoxyphenyl)naphthalen-1-yl)phenyl)(methyl)sulfane (3as): The representative general procedure mentioned above was followed. An oven dried 25 mL Schlenk tube was charged with 2-(1-(4-(methylthio)phenyl)vinyl)benzoic acid **1i** (0.2 mmol), DMAP (0.2 mmol, 1.0 eq.), LiBr (0.1 mmol, 0.5 eq.), $PdCl_2$ (5 mol%) and TFP (10 mol%). Subsequently, 4-methoxyphenylene **2e** (0.4 mmol, 2.0 eq.), Piv_2O (1.4 eq.) and dioxane (2 mL) was added under N_2. The reaction mixture was allowed to react at 160 °C for 12 h. After completion of the reaction, the reaction mixture was then cooled to room temperature, and then the reaction mixture was transferred to a round-bottomed bottle and concentrated under reduced pressure. The crude residue was purified by flash chromatography on silica gel (PE/EA=20/1) to give the desired **3as** as yellow oil in 67% yield (47.7 mg). ^{1}H NMR (400 MHz, $CDCl_3$) δ 8.02 (s, 1H), 7.96 – 7.91 (m, 2H), 7.71 – 7.68 (m, 3H), 7.54 – 7.49 (m, 3H), 7.45 – 7.41 (m, 3H), 7.05 – 7.03 (m, 2H), 3.88 (s, 3H), 2.59 (s, 3H); ^{13}C NMR (100 MHz, $CDCl_3$) δ 159.3 (s), 140.1 (s), 137.6 (s), 137.5 (s), 137.4 (s), 134.2 (s), 133.3 (s), 130. 5 (s), 128.4 (s), 128.4 (s), 126.4 (s), 126.3 (s), 126.2 (s), 125.8 (s), 125.7 (s), 124.6 (s), 114.3 (s), 55.3 (s), 15.8 (s). HRMS (APCI-POS) m/z: $[M+H]^+$ calcd. for $C_{24}H_{21}OS^+$: 357.1308, found: 357.1297.

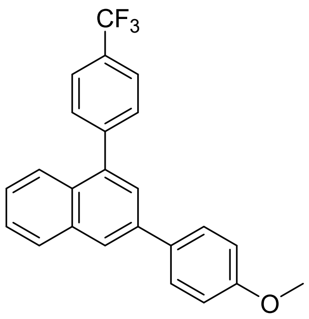

3-(4-methoxyphenyl)-1-(4-(trifluoromethyl)phenyl)naphthalene (3at): The representative general procedure mentioned above was followed. An oven dried 25 mL Schlenk tube was charged with 2-(1-(4-(trifluoromethyl)phenyl)vinyl)benzoic acid **1j** (0.2 mmol), DMAP (0.2 mmol, 1.0 eq.), LiBr (0.1 mmol, 0.5 eq.), $PdCl_2$ (5 mol%) and TFP (10 mol%). Subsequently, 4-methoxyphenylene **2e** (0.4 mmol, 2.0 eq.), Piv_2O (1.4 eq.) and dioxane (2 mL) was added under N_2. The reaction mixture was allowed to react at 160 °C for 12 h. After completion of the reaction, the reaction mixture was then cooled to room temperature, and then the reaction mixture was transferred to a round-bottomed bottle and concentrated under reduced pressure. The crude residue was purified by flash chromatography on silica gel (PE) to give the desired **3at** as yellow oil in 69% yield (52.1 mg). ^{1}H NMR (400 MHz, $CDCl_3$) δ 8.05 (d, *J* = 1.2 Hz, 1H), 7.96 (d, *J* = 8.0 Hz, 1H), 7.81 – 7.77 (m, 3H), 7.71 – 7.70 (m, 1H), 7.69 – 7.68 (m, 2H), 7.67 – 7.65 (m, 2H), 7.56 – 7.51 (m, 1H), 7.45 – 7.41 (m, 1H), 7.05 – 7.01 (m, 2H), 3.88 (s, 3H); ^{13}C NMR (100 MHz, $CDCl_3$) δ 159.41 (s), 144.45 (s), 139.22 (s), 137.63 (s), 134.21 (s), 133.03 (s), 130.40 (s), 130.13 (s), 128.58 (s), 128.41 (s), 126.59 (s), 126.41 (s), 126.18 (s), 125.28 (m), 114.38 (s), 55.38 (s); ^{19}F NMR (376 MHz, $CDCl_3$) δ -62.3 (s). HRMS (APCI-POS) m/z: $[M+H]^+$ calcd. for $C_{24}H_{18}F_3O^+$: 379.1305, found: 379.1305.

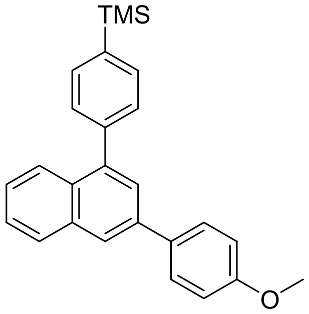

(4-(3-(4-methoxyphenyl)naphthalen-1-yl)phenyl)(methyl)sulfane (3au): The representative general procedure mentioned above was followed. An oven dried 25 mL Schlenk tube was charged with 2-(1-(4-(trimethylsilyl)phenyl)vinyl)benzoic acid **1k** (0.2 mmol), DMAP (0.2 mmol, 1.0 eq.), LiBr (0.1 mmol, 0.5 eq.), $PdCl_2$ (5 mol%) and TFP (10 mol%). Subsequently, 4-methoxyphenylene **2e** (0.4

mmol, 2.0 eq.), Piv_2O (1.4 eq.) and dioxane (2 mL) was added under N_2. The reaction mixture was allowed to react at 160 °C for 12 h. After completion of the reaction, the reaction mixture was then cooled to room temperature, and then the reaction mixture was transferred to a round-bottomed bottle and concentrated under reduced pressure. The crude residue was purified by flash chromatography on silica gel (PE) to give the desired **3au** as yellow oil in 67% yield (46.6 mg). ^1H NMR (400 MHz, $CDCl_3$) δ 8.04 (s, 1H), 7.97 – 7.95 (m, 2H), 7.74 – 7.68 (m, 5H), 7.59 – 7.57 (m, 2H), 7.53 (t, *J* = 6.8 Hz, 1H), 7.44 (t, *J* = 7.8 Hz, 1H), 7.10 – 6.95 (m, 2H), 3.89 (s, 3H), 0.39 (s, 9H); ^{13}C NMR (100 MHz, $CDCl_3$) δ 159.3 (s), 141.1 (s), 140.7 (s), 139.3 (s), 137.6 (s), 134.2 (s), 133.3 (s), 133.3 (s), 130. 5 (s), 129.4 (s), 128.4 (s), 126.5 (s), 126.1 (s), 125.9 (s), 125.8 (s), 124.6 (s), 114.3 (s), 55.3 (s), -1.0 (s). HRMS (APCI-POS) m/z: $[M+H]^+$ calcd. for $C_{26}H_{27}OSi^+$: 383.1826, found: 383.1836.

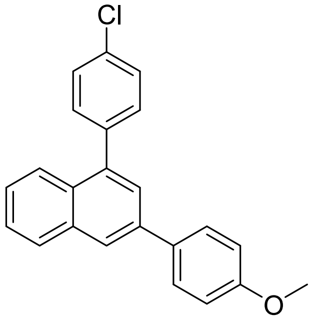

1-(4-chlorophenyl)-3-(4-methoxyphenyl)naphthalene (3av): The representative general procedure mentioned above was followed. An oven dried 25 mL Schlenk tube was charged with 2-(1-(4-chlorophenyl)vinyl)benzoic acid **1l** (0.2 mmol), DMAP (0.2 mmol, 1.0 eq.), LiBr (0.1 mmol, 0.5 eq.), $PdCl_2$ (5 mol%) and TFP (10 mol%). Subsequently, 4-methoxyphenylene **2e** (0.4 mmol, 2.0 eq.), Piv_2O (1.4 eq.) and dioxane (2 mL) was added under N_2. The reaction mixture was allowed to react at 160 °C for 12 h. After completion of the reaction, the reaction mixture was then cooled to room temperature, and then the reaction mixture was transferred to a round-bottomed bottle and concentrated under reduced pressure. The crude residue was purified by flash chromatography on silica gel (PE) to give the desired **3av** as yellow oil in 77% yield (52.9 mg). ^1H NMR (400 MHz, $CDCl_3$) δ 8.03 (s, 1H), 7.96 – 7.94 (m, 1H), 7.85 – 7.83 (m, 1H), 7.70 – 7.68 (m, 2H), 7.66 (d, *J* = 10.4 Hz, 1H), 7.54 – 7.51 (m, 1H), 7.50 – 7.47 (s, 4H), 7.45 – 7.42 (m, 1H), 7.06 – 7.01 (m, 2H), 3.88 (s, 3H)；^{13}C NMR (100 MHz, $CDCl_3$) δ 159.3 (s), 139.4 (s), 139.1 (s), 137.6 (s), 134.2 (s), 133.4 (s), 133.1 (s), 131.3 (s), 130.3 (s), 128.5 (s), 128.4 (s), 126.5 (s), 126.3 (s), 125.9 (s), 125.5 (s), 124.9 (s), 114.3 (s), 55.4 (s). This compound is known.[5]

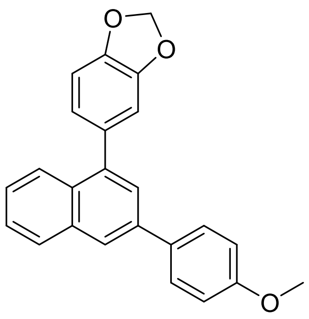

5-(3-(4-methoxyphenyl)naphthalen-1-yl)benzo[d][1,3]dioxole (3aw): The representative general procedure mentioned above was followed. An oven dried 25 mL Schlenk tube was charged with 2-(1-(benzo[d][1,3]dioxol-5-yl)vinyl)benzoic acid **1m** (0.2 mmol), DMAP (0.2 mmol, 1.0 eq.), LiBr (0.1 mmol, 0.5 eq.), $PdCl_2$ (5 mol%) and TFP (10 mol%). Subsequently, 4-methoxyphenylene **2e** (0.4 mmol, 2.0 eq.), Piv_2O (1.4 eq.) and dioxane (2 mL) was added under N_2. The reaction mixture was allowed to react at 160 °C for 12 h. After completion of the reaction, the reaction mixture was then cooled to room temperature, and then the reaction mixture was transferred to a round-bottomed bottle and concentrated under reduced pressure. The crude residue was purified by flash chromatography on silica gel (PE) to give the desired **3aw** as yellow oil in 65% yield (46.0 mg). ^1H NMR (400 MHz, $CDCl_3$) δ 8.01 (s, 1H), 7.95 (m, 2H), 7.99 – 7.93 (m, 3H), 7.72 – 7.68 (m, 3H), 7.54 – 7.50 (m, 1H), 7.46 – 7.42 (m, 1H), 7.06 – 7.05 (m, 1H), 7.05 – 7.02 (m, 3H), 6.98 – 6.96 (m, 1H), 6.07 (s, 2H), 3.88 (s, 3H); ^{13}C NMR (100 MHz, $CDCl_3$) δ 159.2 (s), 147.5 (s), 146.9 (s), 140.3 (s), 137.5 (s), 134.5 (s), 134.2 (s), 133.3 (s), 130.6 (s), 128.4 (s), 128.4 (s), 126.4 (s), 126.1 (s), 125. 8 (s), 125.7 (s), 124.5 (s), 123.4 (s), 114.3 (s), 110.6 (s), 108.2 (s), 101.1 (s), 55.3 (s). HRMS (APCI-POS) m/z: $[M+H]^+$ calcd. for $C_{24}H_{19}O_3^+$: 355.1329, found: 355.1320.

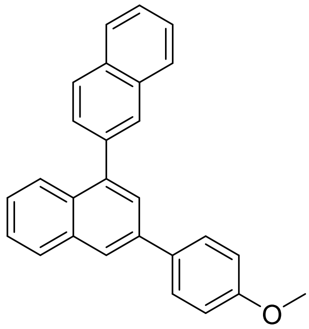

3-(4-methoxyphenyl)-1,2'-binaphthalene (3ax): The representative general procedure mentioned above was followed. An oven dried 25 mL Schlenk tube was charged with 2-(1-(naphthalen-2-yl)vinyl)benzoic acid **1n** (0.2 mmol), DMAP (0.2 mmol, 1.0 eq.), LiBr (0.1 mmol, 0.5 eq.), $PdCl_2$ (5 mol%) and TFP (10 mol%). Subsequently, 4-methoxyphenylene **2e** (0.4 mmol, 2.0 eq.), Piv_2O (1.4 eq.)

and dioxane (2 mL) was added under N_2. The reaction mixture was allowed to react at 160 °C for 12 h. After completion of the reaction, the reaction mixture was then cooled to room temperature, and then the reaction mixture was transferred to a round-bottomed bottle and concentrated under reduced pressure. The crude residue was purified by flash chromatography on silica gel (PE/EA=20/1) to give the desired **3ax** as a yellow solid in 66% yield (47.5 mg). mp: 122-123 °C; ^{1}H NMR (400 MHz, $CDCl_3$) δ 8.08 (s, 1H), 8.04 (s, 1H), 8.01 – 7.97 (m, 3H), 7.96 – 7.93 (m, 2H), 7.82 (d, *J* = 1.8 Hz, 1H), 7.76 – 7.71 (m, 3H), 7.58 – 7.56 (m, 2H), 7.54 – 7.52 (m, 1H), 7.46 – 7.41 (m, 1H), 7.05 (d, *J* = 8.4 Hz, 2H), 3.89 (s, 3H); ^{13}C NMR (100 MHz, $CDCl_3$) δ 159.3 (s), 140.6 (s), 138.3 (s), 137.6 (s), 134.2 (s), 133.4 (s), 133.3 (s), 132.6 (s), 130.6 (s), 128.7 (s), 128.5 (s), 128.4 (s), 128.1 (s), 127.7 (s), 127.6 (s), 126.8 (s), 126.3 (s), 126.2 (s), 126.1 (s), 125.9 (s), 125.8 (s), 124.7 (s), 114.3 (s), 55.3 (s). HRMS (APCI-POS) m/z: $[M+H]^+$ calcd. for $C_{27}H_{21}O^+$: 361.1587, found: 361.1579.

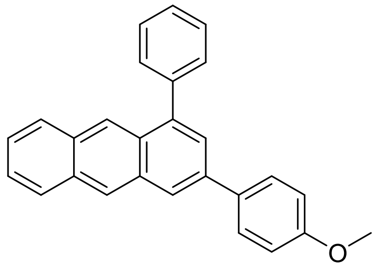

3-(4-methoxyphenyl)-1-phenylanthracene (3ay): The representative general procedure mentioned above was followed. An oven dried 25 mL Schlenk tube was charged with 2-(1-(naphthalen-2-yl)vinyl)benzoic acid **1o** (0.2 mmol), DMAP (0.2 mmol, 1.0 eq.), LiBr (0.1 mmol, 0.5 eq.), $PdCl_2$ (5 mol%) and TFP (10 mol%). Subsequently, 4-methoxyphenylene **2e** (0.4 mmol, 2.0 eq.), Piv_2O (1.4 eq.) and dioxane (2 mL) was added under N_2. The reaction mixture was allowed to react at 160 °C for 12 h. After completion of the reaction, the reaction mixture was then cooled to room temperature, and then the reaction mixture was transferred to a round-bottomed bottle and concentrated under reduced pressure. The crude residue was purified by flash chromatography on silica gel (PE) to give the desired **3ay** as a yellow solid in 75% yield (54.0 mg). mp: 136-137 °C; ^{1}H NMR (400 MHz, $CDCl_3$) δ 8.46 – 8.42 (m, 2H), 8.13 (s, 1H), 7.97 (d, *J* = 8.4 Hz, 1H), 7.85 (d, *J* = 8.4 Hz, 1H), 7.72 – 7.70 (m, 2H), 7.68 – 7.67 (m, 1H), 7.63 – 7.61 (m, 2H), 7.56 – 7.53 (m, 2H), 7.50 – 7.48 (m, 1H), 7.43 – 7.37 (m, 2H), 7.01 – 7.00 (m, 2H), 3.84 (s, 3H); ^{13}C NMR (100 MHz, $CDCl_3$) δ 159.3 (s), 140.6 (s), 138.3 (s), 137.6 (s), 134.2 (s), 133.4 (s), 133.3 (s), 132.6 (s), 130.6 (s), 128.7 (s), 128.5 (s), 128.4 (s), 128.1 (s), 127.7

(s), 127.7 (s), 126.8 (s), 126.3 (s), 126.2 (s), 126.1 (s), 125.9 (s), 125.8 (s), 124.7 (s), 114.3 (s), 55.3 (s). HRMS (APCI-POS) m/z: $[M+H]^+$ calcd. for $C_{27}H_{21}O^+$: 361.1587, found: 361.1589.

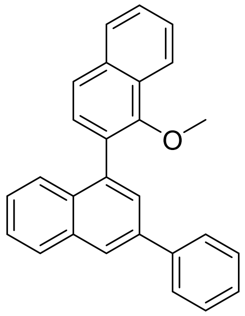

1'-methoxy-3-phenyl-1,2'-binaphthalene (3az): The representative general procedure mentioned above was followed. An oven dried 25 mL Schlenk tube was charged with 2-(1-(1-methoxynaphthalen-2-yl)vinyl)benzoic acid **1p** (0.2 mmol), DMAP (0.2 mmol, 1.0 eq.), LiBr (0.1 mmol, 0.5 eq.), $PdCl_2$ (5 mol%) and TFP (10 mol%). Subsequently, phenylacetylene **2a** (0.4 mmol, 2.0 eq.), Piv_2O (1.4 eq.) and dioxane (2 mL) was added under N_2. The reaction mixture was allowed to react at 160 °C for 12 h. After completion of the reaction, the reaction mixture was then cooled to room temperature, and then the reaction mixture was transferred to a round-bottomed bottle and concentrated under reduced pressure. The crude residue was purified by flash chromatography on silica gel (PE) to give the desired **3az** as a yellow solid in 65% yield (46.8 mg). ^1H NMR (400 MHz, $CDCl_3$) δ 8.33 (d, J = 7.8 Hz, 1H), 8.17 (s, 1H), 8.02 (d, J = 8.2 Hz, 1H), 7.99 – 7.95 (m, 1H), 7.94 – 7.88 (m, 1H), 7.85 – 7.80 (m, 2H), 7.77 – 7.74 (m, 2H), 7.64 – 7.58 (m, 2H), 7.57 – 7.49 (m, 4H), 7.46 – 7.37 (m, 2H), 3.52 (s, 3H); ^{13}C NMR (100 MHz, $CDCl_3$) δ 153.8 (s), 140.8 (s), 137.9 (s), 137.4 (s), 134.5 (s), 133.9 (s), 131.2 (s), 129.8 (s), 128.9 (s), 128.5 (s), 128.3 (s), 128.2 (s), 127.9 (s), 127.4 (s), 127.4 (s), 127.3 (s), 126.4 (s), 126.2 (s), 126.2 (s), 126.1 (s), 125.6 (s), 123.4 (s), 122.6 (s), 77.3 (s), 77.0 (s), 76.7 (s), 61.6 (s). HRMS (APCI-POS) m/z: $[M+H]^+$ calcd. for $C_{27}H_{21}O^+$: 361.1587, found: 361.1587.

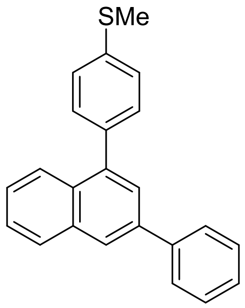

methyl(4-(3-phenylnaphthalen-1-yl)phenyl)sulfane (3ba): The representative general procedure mentioned above was followed. An oven dried 25 mL Schlenk tube was charged with 2-(1-(4-

(methylthio)phenyl)vinyl)benzoic acid **1i** (0.2 mmol), DMAP (0.2 mmol, 1.0 eq.), LiBr (0.1 mmol, 0.5 eq.), $PdCl_2$ (5 mol%) and TFP (10 mol%). Subsequently, phenylacetylene **2a** (0.4 mmol, 2.0 eq.), Piv_2O (1.4 eq.) and dioxane (2 mL) was added under N_2. The reaction mixture was allowed to react at 160 °C for 12 h. After completion of the reaction, the reaction mixture was then cooled to room temperature, and then the reaction mixture was transferred to a round-bottomed bottle and concentrated under reduced pressure. The crude residue was purified by flash chromatography on silica gel (PE) to give the desired **3ba** as yellow oil in 82% yield (53.4 mg). ^{1}H NMR (400 MHz, $CDCl_3$) δ 8.08 (d, J = 1.2 Hz, 1H), 7.99 – 7.93 (m, 2H), 7.79 – 7.76 (m, 2H), 7.72 (d, J = 1.6 Hz, 1H), 7.57 – 7.52 (m, 2H), 7.51 – 7.49 (m, 3H), 7.48 – 7.45 (m, 1H), 7.44 – 7.40 (m, 3H), 2.59 (s, 3H); ^{13}C NMR (100 MHz, $CDCl_3$) δ 140.8 (s), 140.2 (s), 138.0 (s), 137.7 (s), 137.4 (s), 134.2 (s), 130.8 (s), 130.5 (s), 128.9 (s), 128.6 (s), 127.4 (s), 127.4 (s), 126.6 (s), 126.4 (s), 126.2 (s), 126.1 (s), 125.8 (s), 125.4 (s), 15.8 (s). HRMS (APCI-POS) m/z: $[M+H]^+$ calcd. for $C_{23}H_{19}S^+$: 327.1202, found: 327.1202.

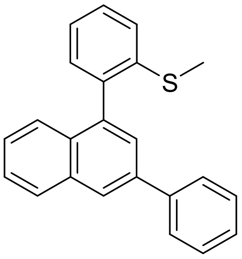

methyl(2-(3-phenylnaphthalen-1-yl)phenyl)sulfane (3bb): The representative general procedure mentioned above was followed. An oven dried 25 mL Schlenk tube was charged with 2-(1-(2-(methylthio)phenyl)vinyl)benzoic acid **1q** (0.2 mmol), DMAP (0.2 mmol, 1.0 eq.), LiBr (0.1 mmol, 0.5 eq.), $PdCl_2$ (5 mol%) and TFP (10 mol%). Subsequently, phenylacetylene **2a** (0.4 mmol, 2.0 eq.), Piv_2O (1.4 eq.) and dioxane (2 mL) was added under N_2. The reaction mixture was allowed to react at 160 °C for 12 h. After completion of the reaction, the reaction mixture was then cooled to room temperature, and then the reaction mixture was transferred to a round-bottomed bottle and concentrated under reduced pressure. The crude residue was purified by flash chromatography on silica gel (PE/EA=20/1) to give the desired **3bb** as yellow oil in 95% yield (61.9 mg). H NMR (400 MHz, $CDCl_3$) δ 8.15 (s, 1H), 8.01 –7.98 (m, 1H), 7.81 – 7.79 (m, 2H), 7.76 (d, J = 1.6 Hz, 1H), 7.57 –7.56 (m, 1H), 7.53 – 7.48 (m, 3H), 7.47 –7.45 (m, 1H), 7.41 –7.39 (m, 2H), 7.34 –7.33 (m, 3H), 2.36 (s, 3H); ^{13}C NMR (100 MHz, $CDCl_3$) δ 140.8 (s), 138.8 (s), 138.7 (s), 138.6 (s), 137.8 (s), 133.9 (s), 131.0 (s), 130.7 (s), 128.8 (s), 128.6 (s), 128.3 (s), 127.4 (s), 127.3 (s), 127.1 (s), 126.2 (s), 126.0 (s), 125.8 (s),

124.7 (s), 124.4 (s), 15.6 (s). HRMS (APCI-POS) m/z: $[M+H]^+$ calcd. for $C_{23}H_{19}S^+$: 327.1202, found: 327.1206.

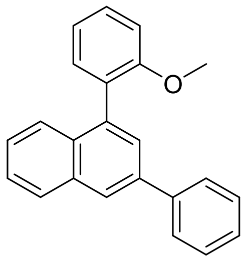

1-(2-methoxyphenyl)-3-phenylnaphthalene (3bc): The representative general procedure mentioned above was followed. An oven dried 25 mL Schlenk tube was charged with 2-(1-(2-methoxyphenyl)vinyl) benzoic acid **1f** (0.2 mmol), DMAP (0.2 mmol, 1.0 eq.), LiBr (0.1 mmol, 0.5 eq.), $PdCl_2$ (5 mol%) and TFP (10 mol%). Subsequently, phenylacetylene **2a** (0.4 mmol, 2.0 eq.), Piv_2O (1.4 eq.) and dioxane (2 mL) was added under N_2. The reaction mixture was allowed to react at 160 °C for 12 h. After completion of the reaction, the reaction mixture was then cooled to room temperature, and then the reaction mixture was transferred to a round-bottomed bottle and concentrated under reduced pressure. The crude residue was purified by flash chromatography on silica gel (PE) to give the desired **3bc** as yellow oil in 88% yield (54.5 mg). ^1H NMR (400 MHz, $CDCl_3$) δ 8.13 (d, J = 1.2 Hz, 1H), 8.01 – 7.99 (m, 1H), 7.84 – 7.81 (m, 2H), 7.78 (d, J = 1.6 Hz, 1H), 7.68 – 7.65 (m, 1H), 7.56 – 7.52 (m, 3H), 7.50 – 7.44 (m, 2H), 7.42 – 7.40 (m, 2H), 7.18 – 7.14 (m, 1H), 7.13 – 7.11 (m, 1H), 3.76 (s, 3H); ^{13}C NMR (100 MHz, $CDCl_3$) δ 157.2 (s), 141.0 (s), 137.9 (s), 137.5 (s), 133.7 (s), 132.4 (s), 131.9 (s), 131.3 (s), 129.8 (s), 129.4 (s), 129.1 (s), 128.8 (s), 128.4 (s), 127.4 (s), 127.2 (s), 127.1 (s), 126.3 (s), 126.0 (s), 125.7 (s), 125.4 (s), 120.6 (s), 110.9 (s). HRMS (APCI-POS) m/z: $[M+H]^+$ calcd. for $C_{23}H_{19}O^+$: 311.1431, found: 311.1437.

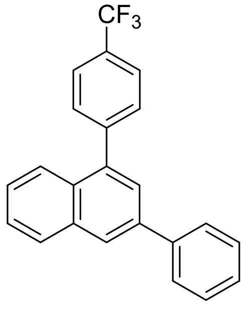

3-phenyl-1-(4-(trifluoromethyl)phenyl)naphthalene (3bd): The representative general procedure mentioned above was followed. An oven dried 25 mL Schlenk tube was charged with 2-(1-(4-(trifluoromethyl)phenyl)vinyl)benzoic acid **1j** (0.2 mmol), DMAP (0.2 mmol, 1.0 eq.), LiBr (0.1 mmol,

0.5 eq.), $PdCl_2$ (5 mol%) and TFP (10 mol%). Subsequently, phenylacetylene **2a** (0.4 mmol, 2.0 eq.), Piv_2O (1.4 eq.) and dioxane (2 mL) was added under N_2. The reaction mixture was allowed to react at 160 °C for 12 h. After completion of the reaction, the reaction mixture was then cooled to room temperature, and then the reaction mixture was transferred to a round-bottomed bottle and concentrated under reduced pressure. The crude residue was purified by flash chromatography on silica gel (PE) to give the desired **3bd** as yellow oil in 53% yield (36.9 mg). ^{1}H NMR (400 MHz, $CDCl_3$) δ 8.12 (s, 1H), 8.0 – 7.98 (m, 1H), 7.84 – 7.82 (m, 1H), 7.80 – 7.78 (m, 2H), 7.77 – 7.75 (m, 2H), 7.71 – 7.70 (s, 1H), 7.69 – 7.67 (m, 2H), 7.56 – 7.55 (s, 1H), 7.53 – 7.47 (m, 3H), 7.42 – 7.40 (m, 1H); ^{13}C NMR (100 MHz, $CDCl_3$) δ 144.4 (s), 140.6 (s), 139.3 (s), 138.0 (s), 134.1 (s), 130.4 (s), 130.4 (s), 129.8 (s), 129.4 (s), 128.9 (s), 128.7 (s), 127.6 (s), 127.4 (s), 126.8 (s), 126.5 (s), 126.1 (s), 125.7 (s), 125.4 (s), 125.3 (s), 125.2 (s). ^{19}F NMR (376 MHz, $CDCl_3$) δ -62.3 (s). HRMS (APCI-POS) m/z: $[M+H]^+$ calcd. for $C_{23}H_{16}F_3^+$: 349.1199, found: 349.1198.

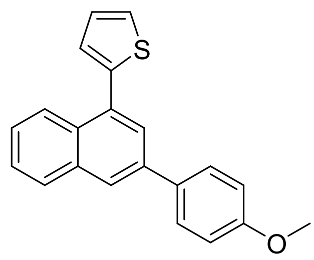

2-(3-(4-methoxyphenyl)naphthalen-1-yl)thiophene (3be): The representative general procedure mentioned above was followed. An oven dried 25 mL Schlenk tube was charged with 2-(1-(thiophen-2-yl)vinyl)benzoic acid **1r** (0.2 mmol), DMAP (0.2 mmol, 1.0 eq.), LiBr (0.1 mmol, 0.5 eq.), $PdCl_2$ (5 mol%) and TFP (10 mol%). Subsequently, 4-methoxyphenylene **2e** (0.4 mmol, 2.0 eq.), Piv_2O (1.4 eq.) and dioxane (2 mL) was added under N_2. The reaction mixture was allowed to react at 160 °C for 12 h. After completion of the reaction, the reaction mixture was then cooled to room temperature, and then the reaction mixture was transferred to a round-bottomed bottle and concentrated under reduced pressure. The crude residue was purified by flash chromatography on silica gel (PE) to give the desired **3be** as yellow oil in 91% yield (57.5 mg). ^{1}H NMR (400 MHz, $CDCl_3$) δ 8.10 (d, *J* = 8.4 Hz, 1H), 7.89 (s, 1H), 7.81 (d, *J* = 7.8 Hz, 1H), 7.72 (d, *J* = 2.0 Hz, 1H), 7.58 – 7.57 (m, 2H), 7.42 – 7.38 (m, 1H), 7.36 – 7.32 (m, 2H), 7.18 (dd, *J* = 4.8, 1.2 Hz, 1H), 7.09 (dd, *J* = 5.2, 3.6 Hz, 1H), 6.91 – 6.89 (m, 2H), 3.74 (s, 3H); ^{13}C NMR (100 MHz, $CDCl_3$) δ 159.3 (s), 141.7 (s), 137.5 (s), 134.3 (s), 133.0 (s), 132.9

(s), 130.7 (s), 128.5 (s), 128.4 (s), 127.7 (s), 127.4 (s), 127.3 (s), 126.4 (s), 126.2 (s), 125.7 (s), 125.6 (s), 125.3 (s), 114.3 (s), 55.3 (s). HRMS (APCI-POS) m/z: $[M + H]^+$ calcd. for $C_{21}H_{17}OS^+$: 317.0995, found: 317.0995.

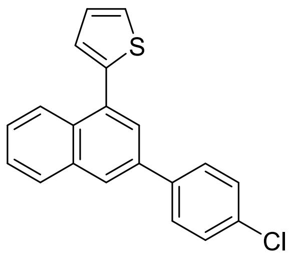

2-(3-(4-chlorophenyl)naphthalen-1-yl)thiophene (3bf): The representative general procedure mentioned above was followed. An oven dried 25 mL Schlenk tube was charged with 2-(1-(thiophen-2-yl)vinyl)benzoic acid **1r** (0.2 mmol), DMAP (0.2 mmol, 1.0 eq.), LiBr (0.1 mmol, 0.5 eq.), $PdCl_2$ (5 mol%) and TFP (10 mol%). Subsequently, 4-chlorophenylene **2m** (0.4 mmol, 2.0 eq.), Piv_2O (1.4 eq.) and dioxane (2 mL) was added under N_2. The reaction mixture was allowed to react at 160 °C for 12 h. After completion of the reaction, the reaction mixture was then cooled to room temperature, and then the reaction mixture was transferred to a round-bottomed bottle and concentrated under reduced pressure. The crude residue was purified by flash chromatography on silica gel (PE) to give the desired **3bf** as a yellow solid in 69% yield (44.1 mg). mp: 81-82 °C; ^1H NMR (400 MHz, $CDCl_3$) δ 8.24 (d, J = 8.0 Hz, 1H), 8.02 (s, 1H), 7.95 – 7.94 (m, 1H), 7.81 (d, J = 2.0 Hz, 1H), 7.69 – 7.66 (m, 2H), 7.56 – 7.52 (m, 2H), 7.48 – 7.45 (m, 3H), 7.31 (d, J = 3.6, 1.2 Hz, 1H), 7.24 – 7.21 (d, J = 5.2, 3.6 Hz, 1H); ^{13}C NMR (100 MHz, $CDCl_3$) δ 141.4 (s), 138.9 (s), 136.6 (s), 134.1 (s), 133.6 (s), 133.2 (s), 131.1 (s), 129.0 (s), 128.6 (s), 127.6 (s), 127.4 (s), 127.3 (s), 126. 7 (s), 126.6 (s), 126.0 (s), 125.8 (s), 125.7 (s). HRMS (APCI-POS) m/z: $[M + H]^+$ calcd. for $C_{20}H_{14}ClS^+$: 321.0500, found: 321.0500.

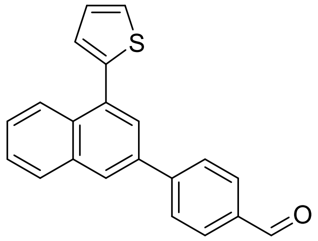

4-(4-(thiophen-2-yl)naphthalen-2-yl)benzaldehyde (3bg): The representative general procedure mentioned above was followed. An oven dried 25 mL Schlenk tube was charged with 2-(1-(thiophen-2-yl)vinyl)benzoic acid **1r** (0.2 mmol), DMAP (0.2 mmol, 1.0 eq.), LiBr (0.1 mmol, 0.5 eq.),

$PdCl_2$ (5 mol%) and TFP (10 mol%). Subsequently, 4-ethynylbezaldehyde **2m** (0.4 mmol, 2.0 eq.), Piv_2O (1.4 eq.) and dioxane (2 mL) was added under N_2. The reaction mixture was allowed to react at 160 °C for 12 h. After completion of the reaction, the reaction mixture was then cooled to room temperature, and then the reaction mixture was transferred to a round-bottomed bottle and concentrated under reduced pressure. The crude residue was purified by flash chromatography on silica gel (PE/EA=20/1) to give the desired **3bg** as yellow oil in 61% yield (38.3 mg); ^{1}H NMR (400 MHz, $CDCl_3$) δ 10.08 (s, 1H), 8.28 – 8.21 (m, 1H), 8.11 (d, *J* = 0.8 Hz, 1H), 8.00 –7.96 (m, 3H), 7.91 –7.88 (m, 2H), 7.86 (d, *J* = 1.6 Hz, 1H), 7.59 – 7.52 (m, 2H), 7.48 (dd, *J* = 5.2, 1.2 Hz, 1H), 7.31 (dd, *J* = 3.6, 1.2 Hz, 1H), 7.23 (dd, *J* = 5.2, 3.6 Hz, 1H); ^{13}C NMR (100 MHz, $CDCl_3$) δ 191.9 (s), 146.5 (s), 141.2 (s), 136.3 (s), 135.3 (s), 134.0 (s), 133.5 (s), 131.5 (s), 130.3 (s), 128.8 (s), 127.9 (s), 127.7 (s), 127.4 (s), 127.3 (s), 127.1 (s), 126.8 (s), 126.7 (s), 125.9 (s), 125.7 (s). HRMS (APCI-POS) m/z: $[M + H]^+$ calcd. for $C_{21}H_{15}OS^+$: 315.0839, found: 315.0839.

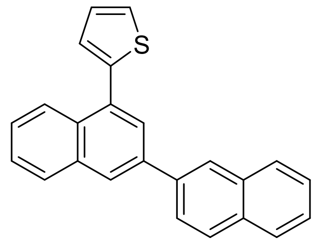

2-([2,2'-binaphthalen]-4-yl)thiophene (3bh): The representative general procedure mentioned above was followed. An oven dried 25 mL Schlenk tube was charged with 2-(1-(thiophen-2-yl)vinyl)benzoic acid **1r** (0.2 mmol), DMAP (0.2 mmol, 1.0 eq.), LiBr (0.1 mmol, 0.5 eq.), $PdCl_2$ (5 mol%) and TFP (10 mol%). Subsequently, 2-naphthalene acetylene **2w** (0.4 mmol, 2.0 eq.), Piv_2O (1.4 eq.) and dioxane (2 mL) was added under N_2. The reaction mixture was allowed to react at 160 °C for 12 h. After completion of the reaction, the reaction mixture was then cooled to room temperature, and then the reaction mixture was transferred to a round-bottomed bottle and concentrated under reduced pressure. The crude residue was purified by flash chromatography on silica gel (PE) to give the desired **3bh** as a yellow solid in 63% yield (42.3 mg). mp: 138-139 °C; ^{1}H NMR (400 MHz, $CDCl_3$) δ 8.28 – 8.25 (m, 1H), 8.22 – 8.18 (m, 2H), 8.00 – 7.98 (m, 2H), 7.95 – 7.92 (m, 2H), 7.91 – 7.86 (m, 2H), 7.58 – 7.53 (m, 2H), 7.51 – 7.49 (m, 2H), 7.48 (dd, *J* = 5.2, 1.2 Hz, 1H), 7.35 (dd, *J* = 3.6, 1.2 Hz, 1H), 7.25 (dd, *J* = 5.2, 3.6 Hz, 1H); ^{13}C NMR (100 MHz, $CDCl_3$) δ 141.7 (s), 137.9 (s), 137.8 (s), 134.3 (s), 133.7 (s), 133.2 (s), 132.7 (s), 131.1 (s), 128.7 (s), 128.6 (s), 128.2 (s), 128.0 (s), 127.7 (s), 127.6 (s), 127.3

(s), 126.5 (s), 126.5 (s), 126.4 (s), 126.4 (s), 126.2 (s), 126.1 (s), 125.8 (s), 125.7 (s), 125.6 (s); HRMS (APCI-POS) m/z: $[M + H]^+$ calcd. for $C_{24}H_{17}S^+$: 337.1046, found: 337.1047.

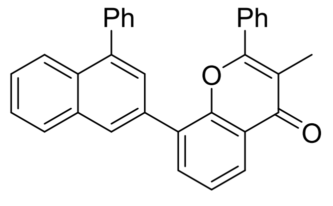

3-methyl-2-phenyl-8-(4-phenylnaphthalen-2-yl)-4H-chromen-4-one (3bi): The representative general procedure mentioned above was followed. An oven dried 25 mL Schlenk tube was charged with carboxylic acid **1a** (0.2 mmol), DMAP (0.2 mmol, 1.0 eq.), LiBr (0.1 mmol, 0.5 eq.), $PdCl_2$ (5 mol%) and TFP (10 mol%). Subsequently, 8-ethynyl-3-methyl-2-phenyl-4H-chromen-4-one **2al** (0.4 mmol, 2.0 eq.), Piv_2O (1.4 eq.) and dioxane (2 mL) was added under N_2. The reaction mixture was allowed to react at 160 °C for 12 h. After completion of the reaction, the reaction mixture was then cooled to room temperature, and then the reaction mixture was transferred to a round-bottomed bottle and concentrated under reduced pressure. The crude residue was purified by flash chromatography on silica gel (PE/EA=10/1) to give the desired **3bh** as a yellow solid in 72% yield (63.0 mg). mp: 141-142 °C; ^1H NMR (400 MHz, $CDCl_3$) δ 8.34 (dd, J = 8.0, 1.2 Hz, 1H), 8.07 (s, 1H), 7.92 (d, J = 8.7 Hz, 2H), 7.88 (dd, J = 7.2, 1.2 Hz, 1H), 7.77 (d, J = 1.6 Hz, 1H), 7.57 – 7.55 (m, 2H), 7.54 – 7.48 (m, 3H), 7.47 – 7.46 (m, 3H), 7.43 – 7.42 (m, 2H), 7.41 – 7.36 (m, 3H), 2.24 (s, 3H); ^{13}C NMR (100 MHz, $CDCl_3$) δ 178.9 (s), 160.9 (s), 153.2 (s), 140.3 (s), 139.9 (s), 134.3 (s), 133.8 (s), 133.1 (s), 133.1 (s), 130.9 (s), 130.0 (s), 129.9 (s), 129.0 (s), 128.7 (s), 128.5 (s), 128.3 (s), 128.2 (s), 128.0 (s), 127.3 (s), 126.5 (s), 126.2 (s), 125.8 (s), 125.4 (s), 124.8 (s), 122.9 (s), 117.3 (s), 11.8 (s). HRMS (APCI-POS) m/z: $[M + H]^+$ calcd. for $C_{32}H_{23}O_2^+$: 439.1693, found: 439.1693.

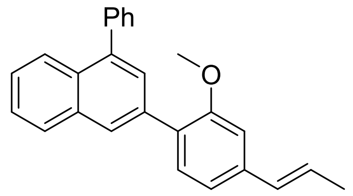

(E)-3-(2-methoxy-4-(prop-1-en-1-yl)phenyl)-1-phenylnaphthalene (3bj): The representative general procedure mentioned above was followed. An oven dried 25 mL Schlenk tube was charged with carboxylic acid **1a** (0.2 mmol), DMAP (0.2 mmol, 1.0 eq.), LiBr (0.1 mmol, 0.5 eq.), $PdCl_2$ (5 mol%) and TFP (10 mol%). Subsequently, 1-ethynyl-2-methoxy-4-propylbenzene **2am** (0.4 mmol, 2.0

eq.), Piv_2O (1.4 eq.) and dioxane (2 mL) was added under N_2. The reaction mixture was allowed to react at 160 °C for 12 h. After completion of the reaction, the reaction mixture was then cooled to room temperature, and then the reaction mixture was transferred to a round-bottomed bottle and concentrated under reduced pressure. The crude residue was purified by flash chromatography on silica gel (PE) to give the desired **3bj** as yellow oil in 74% yield (51.8 mg). ^{1}H NMR (400 MHz, $CDCl_3$) δ 8.02 (s, 1H), 7.93 (t, J = 7.6 Hz, 2H), 7.67 (d, J = 2.0 Hz, 1H), 7.59 – 7.56 (m, 2H), 7.53 – 7.49 (m, 3H), 7.46 – 7.40 (m, 3H), 7.06 (dd, J = 8.0, 1.6 Hz, 1H), 7.00 (d, J = 1.2 Hz, 1H), 6.47 (dd, J = 15.6, 1.2 Hz, 1H), 6.40 – 6.27 (m, 1H), 3.86 (s, 3H), 1.94 (d, J = 6.4, 1.6 Hz, 3H); ^{13}C NMR (100 MHz, $CDCl_3$) δ 156.7 (s), 140.8 (s), 139.5 (s), 138.8 (s), 135.4 (s), 133.8 (s), 131.2 (s), 130.8 (s), 130.6 (s), 130.2 (s), 129.0 (s), 128.9 (s), 128.5 (s), 128.2 (s), 127.7 (s), 127.1 (s), 126.2 (s), 125.9 (s), 125.8 (s), 125.7 (s), 118.7 (s), 108.6 (s), 55.6 (s), 18.5 (s). HRMS (APCI-POS) m/z: $[M + H]^+$ calcd. for $C_{26}H_{23}O^+$: 351.1744, found: 351.1746.

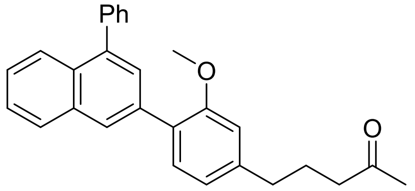

5-(3-methoxy-4-(4-phenylnaphthalen-2-yl)phenyl)pentan-2-one (3bk): The representative general procedure mentioned above was followed. An oven dried 25 mL Schlenk tube was charged with carboxylic acid **1a** (0.2 mmol), DMAP (0.2 mmol, 1.0 eq.), LiBr (0.1 mmol, 0.5 eq.), $PdCl_2$ (5 mol%) and TFP (10 mol%). Subsequently, 5-(4-ethynyl-3-methoxyphenyl)pentan-2-one **2an** (0.4 mmol, 2.0 eq.), Piv_2O (1.4 eq.) and dioxane (2 mL) was added under N_2. The reaction mixture was allowed to react at 160 °C for 12 h. After completion of the reaction, the reaction mixture was then cooled to room temperature, and then the reaction mixture was transferred to a round-bottomed bottle and concentrated under reduced pressure. The crude residue was purified by flash chromatography on silica gel (PE/EA=20/1) to give the desired **3bk** as yellow oil in 68% yield (53.3.0 mg). ^{1}H NMR (400 MHz, $CDCl_3$) δ 8.00 (s, 1H), 7.95 – 7.92 (m, 2H), 7.66 (d, J = 1.2 Hz, 1H), 7.58 – 7.56 (m, 2H), 7.53 – 7.50 (m, 3H), 7.46 – 7.43 (m, 2H), 7.41 – 7.38 (m, 1H), 6.92 – 6.88 (m, 2H), 3.85 (s, 3H), 2.97 (t, J = 7.2 Hz, 2H), 2.86 (t, J = 7.6 Hz, 2H), 2.20 (s, 3H); ^{13}C NMR (100 MHz, $CDCl_3$) δ 207.9 (s), 156.6 (s), 142.1 (s), 140.8 (s), 139.5 (s), 135.4 (s), 133.8 (s), 131.1 (s), 130.5 (s), 130.1 (s), 129.0 (s), 128.5 (s), 128.2 (s), 128.2 (s), 127.7 (s), 127.1 (s), 125.9 (s), 125.8 (s), 125.7 (s), 120.6 (s), 111.5 (s), 55.6 (s),

45.1 (s), 30.1 (s), 29.8 (s). HRMS (APCI-POS) m/z: $[M + H]^+$ calcd. for $C_{28}H_{27}O_2^+$: 395.2006, found: 395.2006.

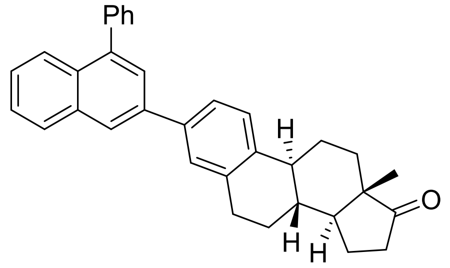

(8R,9S,13S,14S)-13-methyl-3-(4-phenylnaphthalen-2-yl)-6,7,8,9,11,12,13,14,15,16-decahydro-17H-cyclopenta[*a*]phenanthren-17-one (3bl): The representative general procedure mentioned above was followed. An oven dried 25 mL Schlenk tube was charged with carboxylic acid **1a** (0.2 mmol), DMAP (0.2 mmol, 1.0 eq.), LiBr (0.1 mmol, 0.5 eq.), $PdCl_2$ (5 mol%) and TFP (10 mol%). Subsequently,(8R,9S,13S,14S)-3-ethynyl-13-methyl-6,7,8,9,11,12,13,14,15,16-decahydro-17H-cyclopenta[a]phenanthren-17-one **2ao** (0.4 mmol, 2.0 eq.), Piv_2O (1.4 eq.) and dioxane (2 mL) was added under N_2. The reaction mixture was allowed to react at 160 °C for 12 h. After completion of the reaction, the reaction mixture was then cooled to room temperature, and then the reaction mixture was transferred to a round-bottomed bottle and concentrated under reduced pressure. The crude residue was purified by flash chromatography on silica gel (PE/EA=20/1) to give the desired **3bl** as a yellow solid in 69% yield (62.9 mg). mp: 117-118 °C; ^1H NMR (400 MHz, $CDCl_3$) δ 8.07 (s, 1H), 7.98 – 7.91 (m, 2H), 7.73 (d, J = 2.0 Hz, 1H), 7.59 – 7.54 (m, 4H), 7.53 – 7.50 (m, 3H), 7.49 – 7.41 (m, 3H), 3.05 – 3.01 (m, 2H), 2.58 – 2.47 (m, 2H), 2.41 – 2.35 (m, 1H), 2.22 – 2.12 (m, 2H), 2.09 – 2.03 (m, 2H), 1.71 – 1.61 (m, 2H), 1.56 – 1.46 (m, 4H), 0.95 (s, 3H); ^{13}C NMR (100 MHz, $CDCl_3$) δ 220.8 (s), 140.7 (s), 139.1 (s), 138.3 (s), 137.7 (s), 136.9 (s), 134.1 (s), 130.7 (s), 130.0 (s), 128.5 (s), 128.3 (s), 127.9 (s), 127.3 (s), 126.5 (s), 126.1 (s), 125.9 (s), 125.8 (s), 125.0 (s), 124.7 (s), 50.4 (s), 47.9 (s), 44.3 (s), 38.1 (s), 35.8 (s), 31. 6 (s), 29.5 (s), 26.5 (s), 25.7 (s), 21.6 (s), 13.8 (s). HRMS (APCI-POS) m/z: $[M + H]^+$ calcd. for $C_{34}H_{33}O^+$: 457.2526, found: 457.2526.

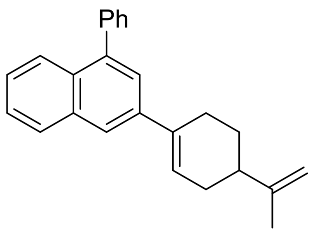

1-phenyl-3-(4-(prop-1-en-2-yl)cyclohex-1-en-1-yl)naphthalene (3bm): The representative general procedure mentioned above was followed. An oven dried 25 mL Schlenk tube was charged with carboxylic acid **1a** (0.2 mmol), DMAP (0.2 mmol, 1.0 eq.), LiBr (0.1 mmol, 0.5 eq.), $PdCl_2$ (5 mol%) and TFP (10 mol%). Subsequently, 1-ethynyl-4-(prop-1-en-2-yl)cyclohex-1-ene **2ap** (0.4 mmol, 2.0 eq.), Piv_2O (1.4 eq.) and dioxane (2 mL) was added under N_2. The reaction mixture was allowed to react at 160 °C for 12 h. After completion of the reaction, the reaction mixture was then cooled to room temperature, and then the reaction mixture was transferred to a round-bottomed bottle and concentrated under reduced pressure. The crude residue was purified by flash chromatography on silica gel (PE) to give the desired **3bm** as a yellow solid in 72% yield (46.6 mg). ^1H NMR (400 MHz, $CDCl_3$) δ 7.00 – 6.91 (m, 3H), 6.67 – 6.62 (m, 4H), 6.57 – 6.55 (m, 4H), 6.50 – 6.48 (m, 1H), 6.38 – 6.37 (m, 1H), 5.47 – 5.46 (m, 1H), 3.90 (s, 2H), 1.80 – 1.78 (m, 2H), 1.49 – 1.43 (m, 2H), 1.33 – 1.17 (m, 2H), 0.92 (s, 3H), 0.43 – 0.37 (m, 1H); ^{13}C NMR (100 MHz, $CDCl_3$) δ 149.7 (s), 140.9 (s), 139.9 (s), 138.7 (s), 135.8 (s), 133.9 (s), 130.7 (s), 130.0 (s), 128.4 (s), 128.2 (s), 127.2 (s), 125.9 (s), 125.8 (s), 125.7 (s), 125.1 (s), 124.9 (s), 122.9 (s), 108.8 (s), 40.8 (s), 31.5 (s), 27.9 (s), 27.8 (s), 20.8 (s). HRMS (APCI-POS) m/z: $[M]^+$ calcd. for $C_{25}H_{23}^+$: 323.1795, found: 323.1795.

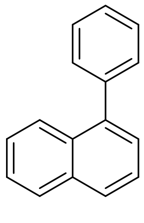

1-phenylnaphthalene (6)： ^1H NMR (400 MHz, $CDCl_3$) δ 8.02 – 7.98 (m, 2H), 7.95 – 7.93 (m, 1H), 7.64 – 7.58 (m, 4H), 7.57 – 7.55 (m, 2H), 7.52 – 7.49 (td, J = 6.9, 1.1 Hz, 3H); ^{13}C NMR (100 MHz, $CDCl_3$) δ 140.7 (s), 140.2 (s), 133.8 (s), 131.6 (s), 130.0 (s), 128.2 (s), 127.6 (s), 127.2 (s), 126.9 (s), 125.9 (s), 125.7 (s), 125.3 (s).

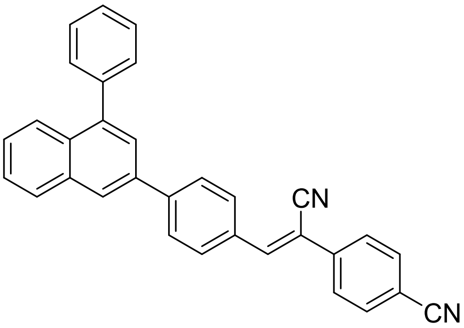

(Z)-4-(1-cyano-2-(4-(4-phenylnaphthalen-2-yl)phenyl)vinyl)benzonitrile (7): ^{1}H NMR (400 MHz, $CDCl_3$) δ 8.07 (s, 1H), 7.98 (d, *J* = 7.8 Hz, 2H), 7.92 (d, *J* = 8.0 Hz, 1H), 7.84 (t, *J* = 7.6 Hz, 3H), 7.76 – 7.74 (m, 2H), 7.70 – 7.69 (m, 1H), 7.67 (d, *J* = 2.0 Hz, 1H), 7.61 (s, 1H), 7.50 – 7.46 (m, 3H), 7.46 – 7.44 (m, 2H), 7.42 – 7.37 (m, 2H), 7.18 (s, 1H); ^{13}C NMR (100 MHz, $CDCl_3$) δ 144.3 (s), 143.9 (s), 141.3 (s), 140.4 (s), 138.8 (s), 136.3 (s), 134.1 (s), 132.8 (s), 131.9 (s), 131.3 (s), 130.3 (s), 130.0 (s), 128.8 (s), 128.4 (s), 127.9 (s), 127.6 (s), 126.7 (s), 126.5 (s), 126.4 (s), 125.9 (s), 125.8 (s), 118.2 (s), 117.3 (s), 112.6 (s), 109.4 (s).

***N*-(4-(4-phenylnaphthalen-2-yl)phenyl)dibenzo[b,d]thiophen-3-amine (8):** ^{1}H NMR (400 MHz, $CDCl_3$) 1H NMR (400 MHz, CDCl3) δ 7.96 – 7.94 (m, 3H), 7.87 (d, *J* = 8.2 Hz, 1H), 7.82 (d, J = 8.4 Hz, 1H), 7.72 (d, J = 8.2 Hz, 1H), 7.65 – 7.63 (m, 3H), 7.50 – 7.48 (m, 3H), 7.45 – 7.41 (m, 3H), 7.40 – 7.24 (m, 5H), 7.20 – 7.09 (m, 3H); ^{13}C NMR (100 MHz, $CDCl_3$) δ 142.2 (s), 142.1 (s), 141.0 (s), 140.8 (s), 140.7 (s), 138.6 (s), 137.5 (s), 135.6 (s), 134.2 (s), 133.8 (s), 130.5 (s), 130.1 (s), 129.5 (s), 128.4 (s), 128.3 (s), 128.2 (s), 127.32 (s), 126.3 (s), 126.2 (s), 125.9 (s), 125.8 (s), 125.5 (s), 124.4 (s), 124.3 (s),122.6 (s), 122.3 (s), 120.6 (s), 118.4 (s), 116.0 (s), 110.1 (s).

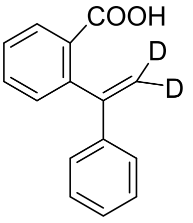

2-(1-phenylvinyl-2,2-*d*$_2$)benzoic acid: ^{1}H NMR (400 MHz, $CDCl_3$) δ 7.96 (dd, *J* = 8.0, 1.2 Hz, 1H), 7.59 (td, *J* = 7.6, 1.6 Hz, 1H), 7.46 (td, *J* = 7.6, 1.2 Hz, 1H), 7.40 (dd, *J* = 7.6, 0.8 Hz, 1H), 7.29 – 7.26 (m, 2H), 7.26 – 7.22 (m, 3H), 5.69 (s, 0.07H), 5.24 (s, 0.07H); ^{13}C NMR (100 MHz, $CDCl_3$) δ 171.6 (s), 149.4 (s), 143.5 (s), 140.8 (s), 132.4 (s), 131.6 (s), 130.6 (s), 128.1 (s), 127.7 (s), 127.5 (s), 126.8 (s).

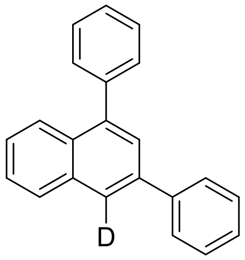

3-(4-methoxyphenyl)-1-phenylnaphthalene-2-*d* (3a-*d*)：^{1}H NMR (400 MHz, $CDCl_3$) δ 8.10 (d, *J* = 1.4 Hz, 0.16H), 8.01 – 7.93 (m, 2H), 7.81– 7.78 (m, 2H), 7.76 (s, 1H), 7.62 – 7.56 (m, 3H), 7.55 – 7.50 (m, 4H), 7.50 – 7.44 (m, 2H), 7.44 – 7.39 (m, 1H).

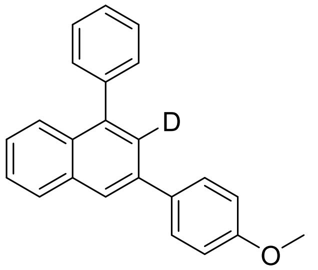

3-(4-methoxyphenyl)-1-phenylnaphthalene-2-*d* (3e-*d*)：^{1}H NMR (400 MHz, $CDCl_3$) δ 8.04 (s, 1H), 7.97 – 7.91 (m, 2H), 7.73 – 7.71 (m, 2H), 7.60 – 7.56 (m, 2H), 7.56 – 7.51 (m, 3H), 7.50 – 7.47 (m, 1H), 7.45 – 7.41 (m, 1H), 7.07 – 7.02 (m, 2H), 3.89 (s, 3H); ^{13}C NMR (100 MHz, $CDCl_3$) δ 159.3 (s), 140.83 – 140.55 (m), 137.6 (s), 137.5 (s), 134.2 (s), 133.4 (s), 133.3 (s), 130.5 (s), 130.1 (s), 128.4 (s), 128.3 (s), 127.3 (s), 126.5 (s), 126.1 (s), 125.7 (s), 125.8 (s), 124.6 (s), 114.3 (s), 55.4 (s).

5. References

[1] Li, H.; Shan, L.; Min, L.; Weng, Y.; Wang, X.; Hu, Y. Tandem Synthesis of 1,3-Disubstituted Naphthalenes via TfOH Promoted Directed-Aldol and Friedel−Crafts Reactions. *J. Org. Chem.* **2021**, *86,* 15011−15019.

[2] Rohand, T.; Sbi, S.; Mkpenie, V.; Tanemura, K. Ligand-Free and Solvent-Free Synthesis of 1,3-Disubstituted Naphthalenes through Stille Coupling. *Synlett* **2020**; *31*, 903−906.

[3] Zhang, C.; Chen, L.; Chen, K.; Jiang, H.; Zhu. S. Iron/Zinc-catalyzed Benzannulation Reactions of 2-(2-Oxo-Alkyl)Benzketones Leading to Naphthalene and Isoquinoline Derivatives. *Org. Chem. Front.*, 2**018**, *5*, 1028−1033.

[4] Cao, J.; Zhang, H.; Tang, W.; Xu, X.; Li, L.; Wang, Z.; Zhang, J.; He, L. Preparation of Anthracene Derivatives for Organic Electroluminescent Materials, Light Emitting Devices and Consumer Products. China, CN116444468.

[5] Gupta, K. C.; Pathak, P. K.; Saxena, B. K. Reaction of Benzyltriphenylarsonium Ylides with *α,β*-Unsaturated Ketones: Synthesis of 1,3-Diarylnaphthalenes. *Indian J. Chem. B.* **1985**, *24B*, 783−784.

[6] Carl, P. R.; Benson, J. J.; Alvar, D. G.; Antonio, T. Exposing the Origins of Irreproducibility in Fluorine NMR Spectroscopy. Angew. Chem. Int. Ed. **2018**, 57, 9528−9533.

6. Copies of ^{1}H ,^{13}C and ^{19}F Spectrum of the Products

^{1}H NMR (400 MHz, $CDCl_3$) and ^{13}C NMR (100 MHz, $CDCl_3$) Spectrum of **3a**.

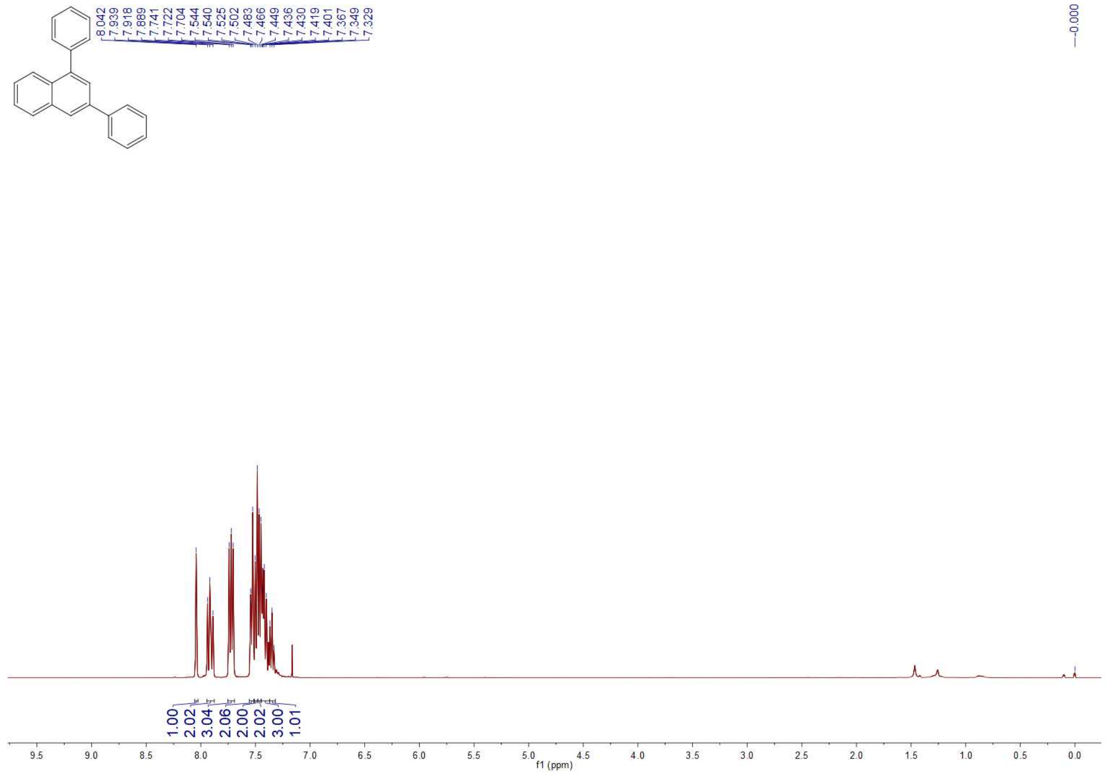

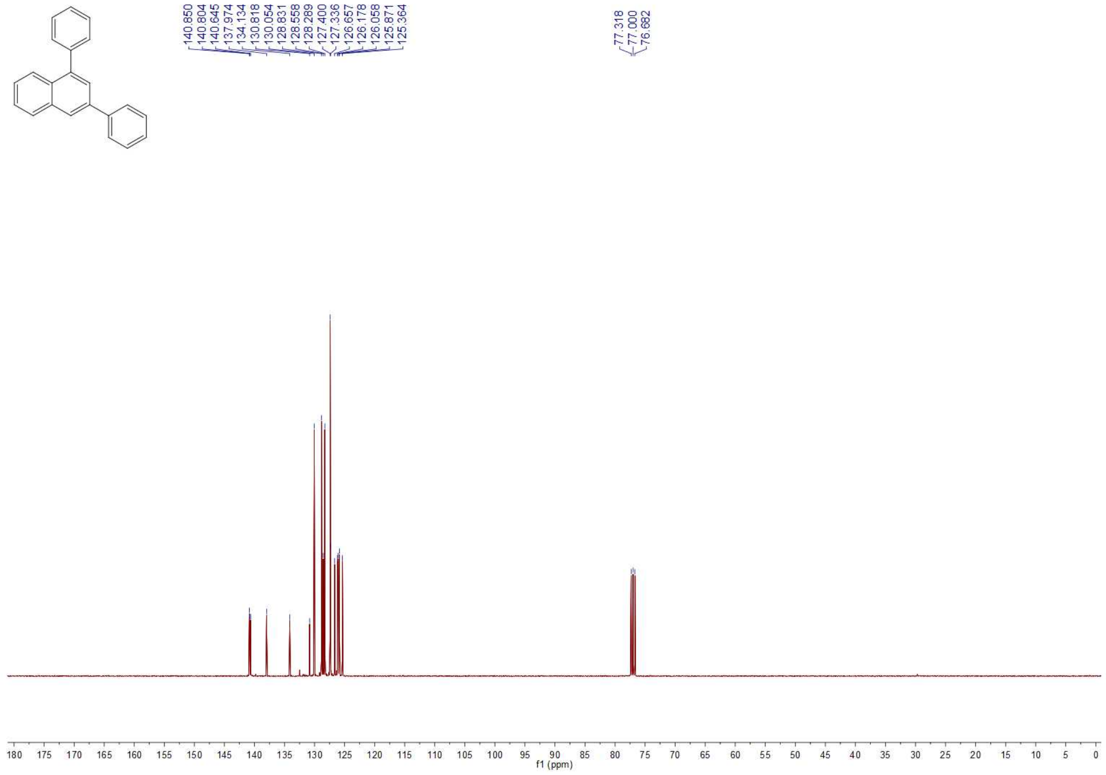

^{1}H NMR (400 MHz, $CDCl_3$) and ^{13}C NMR (100 MHz, $CDCl_3$) Spectrum of **3b**.

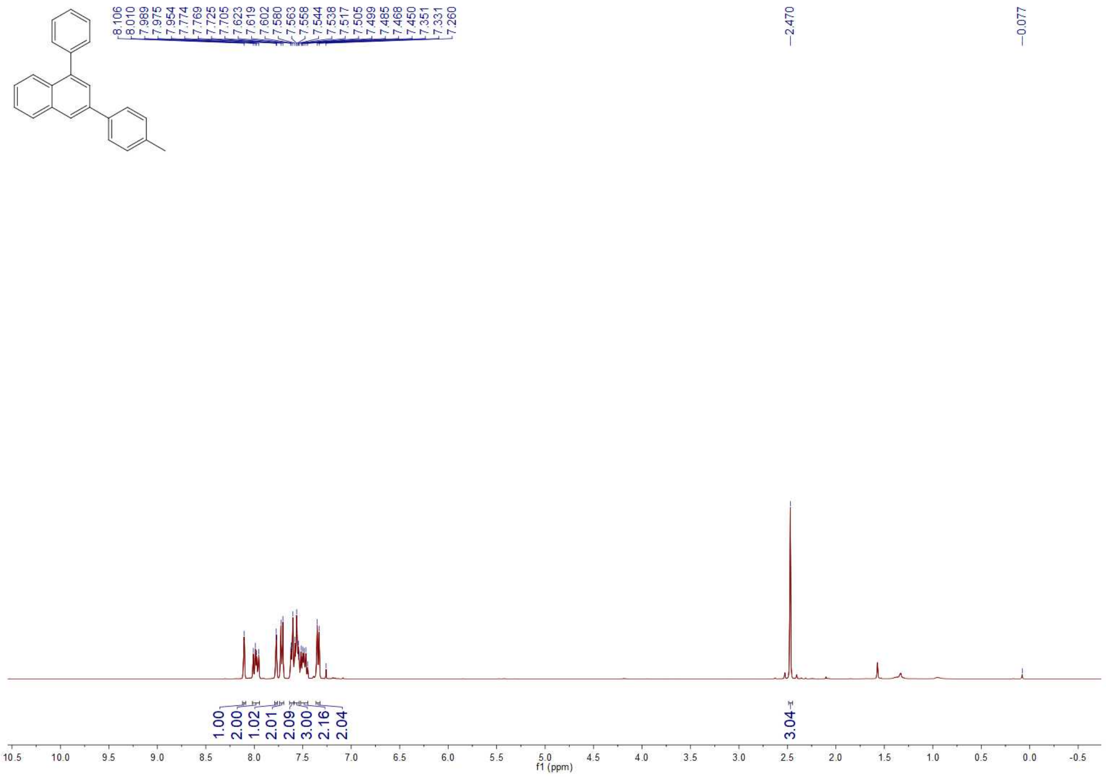

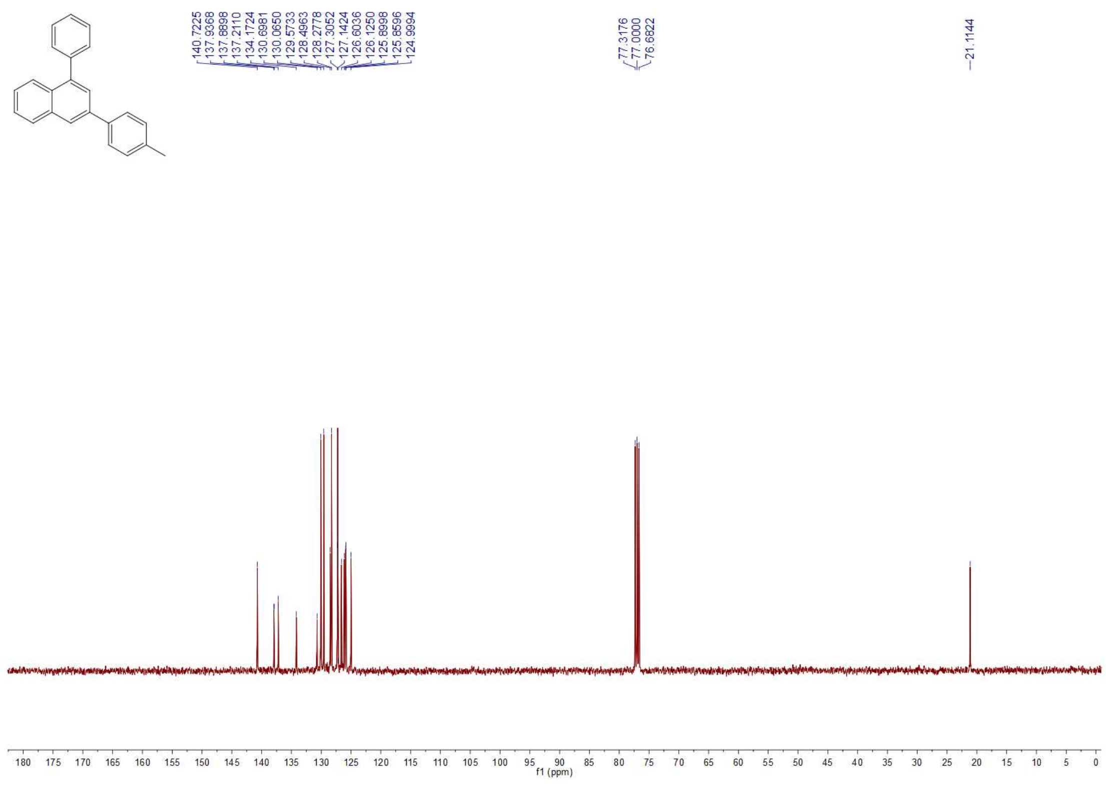

^{1}H NMR (400 MHz, $CDCl_3$) and ^{13}C NMR (100 MHz, $CDCl_3$) Spectrum of **3c**.

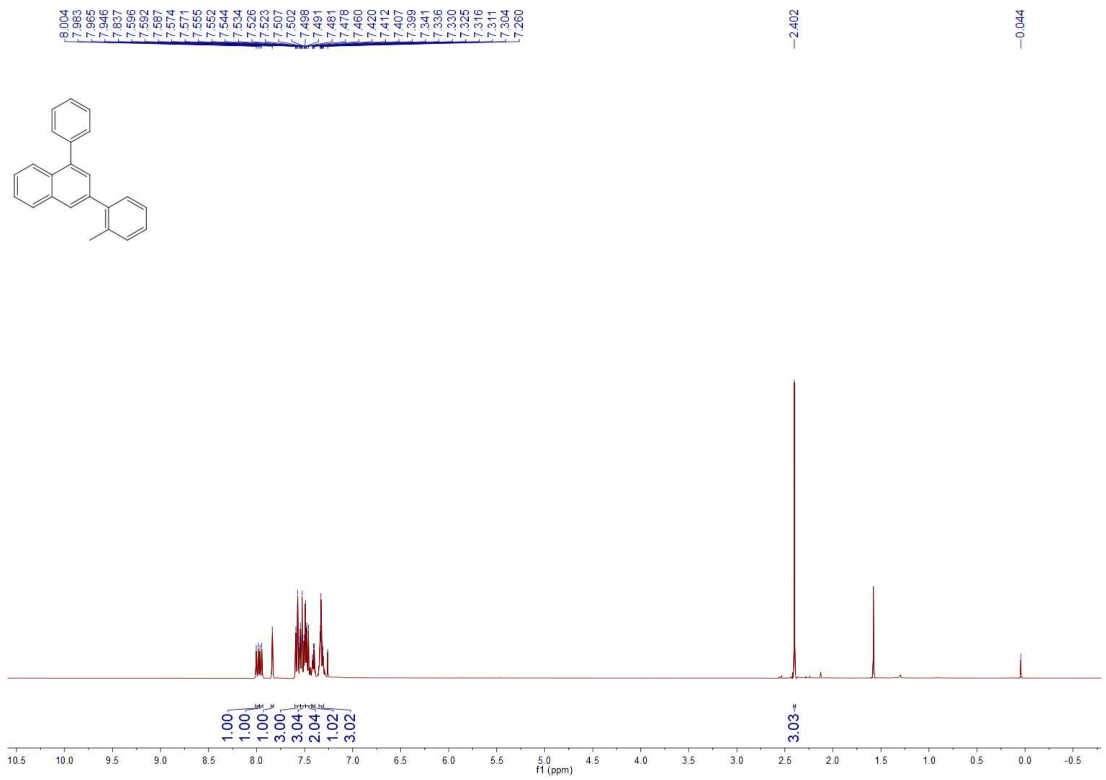

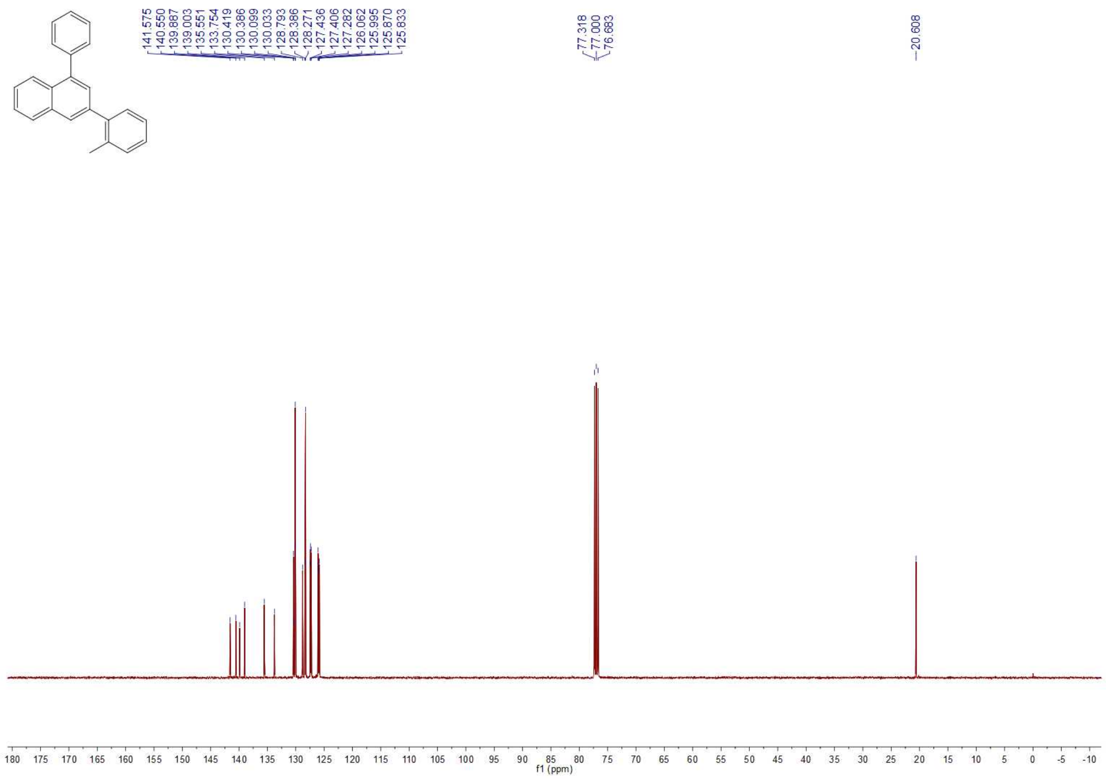

^{1}H NMR (400 MHz, $CDCl_3$) and ^{13}C NMR (100 MHz, $CDCl_3$) Spectrum of **3d**.

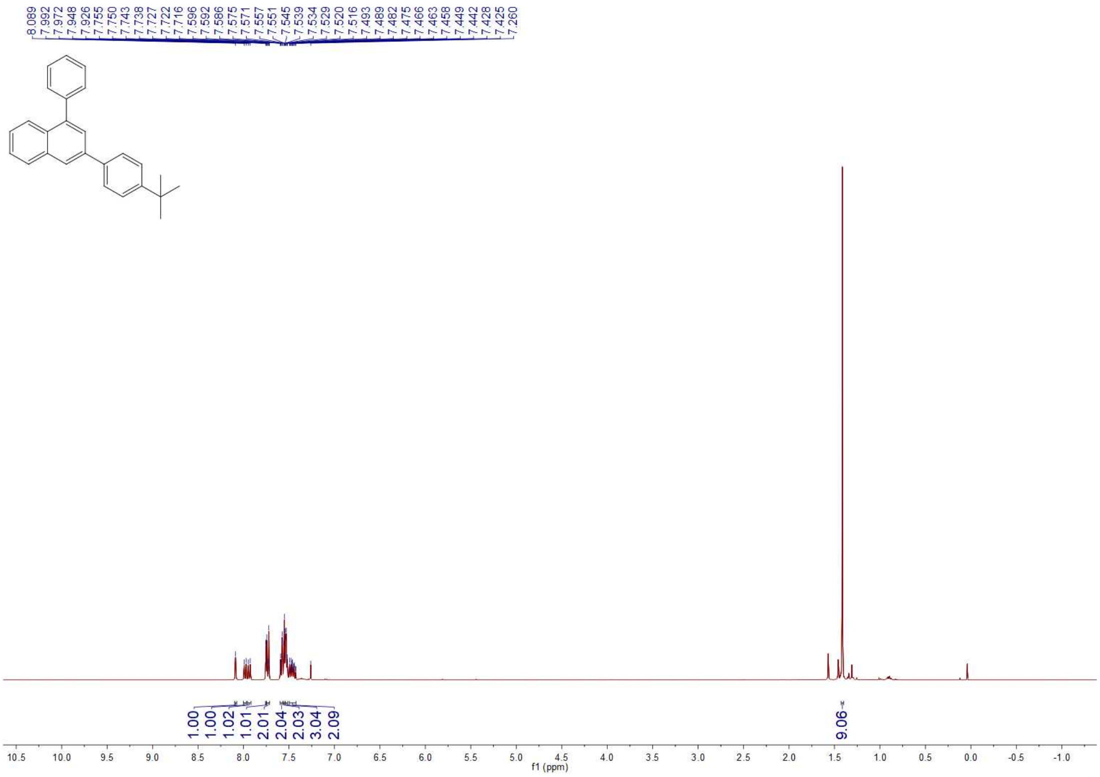

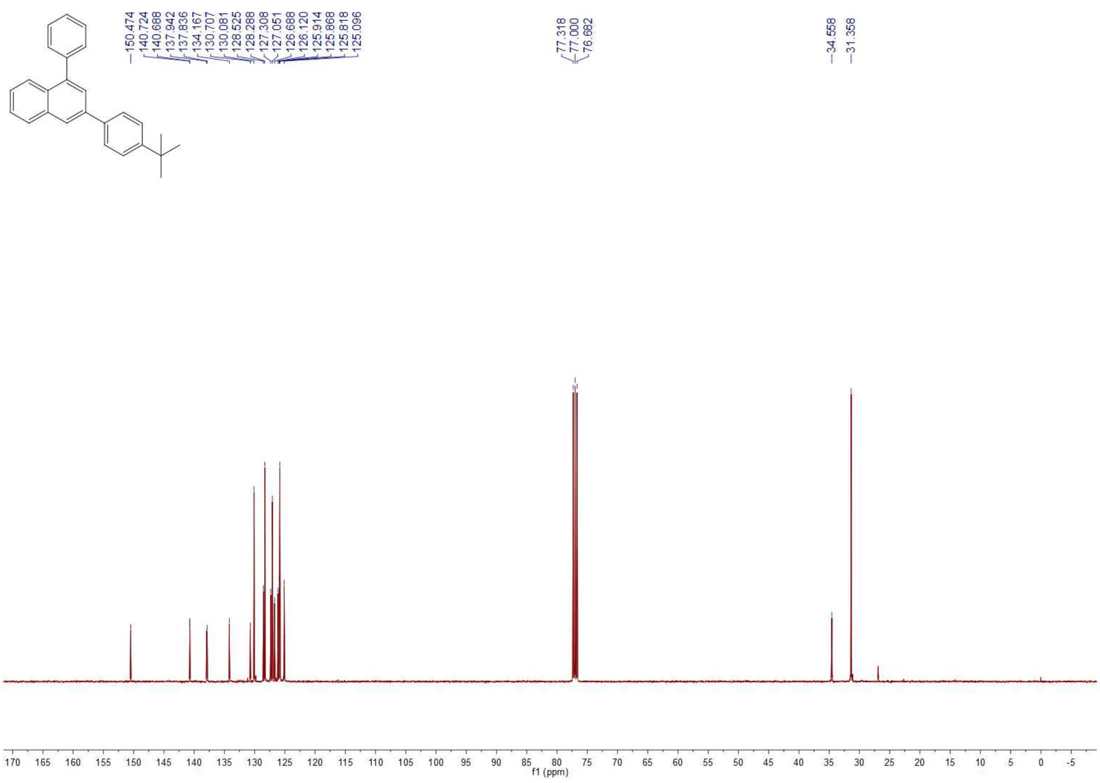

^{1}H NMR (400 MHz, $CDCl_3$) and ^{13}C NMR (100 MHz, $CDCl_3$) Spectrum of **3e**.

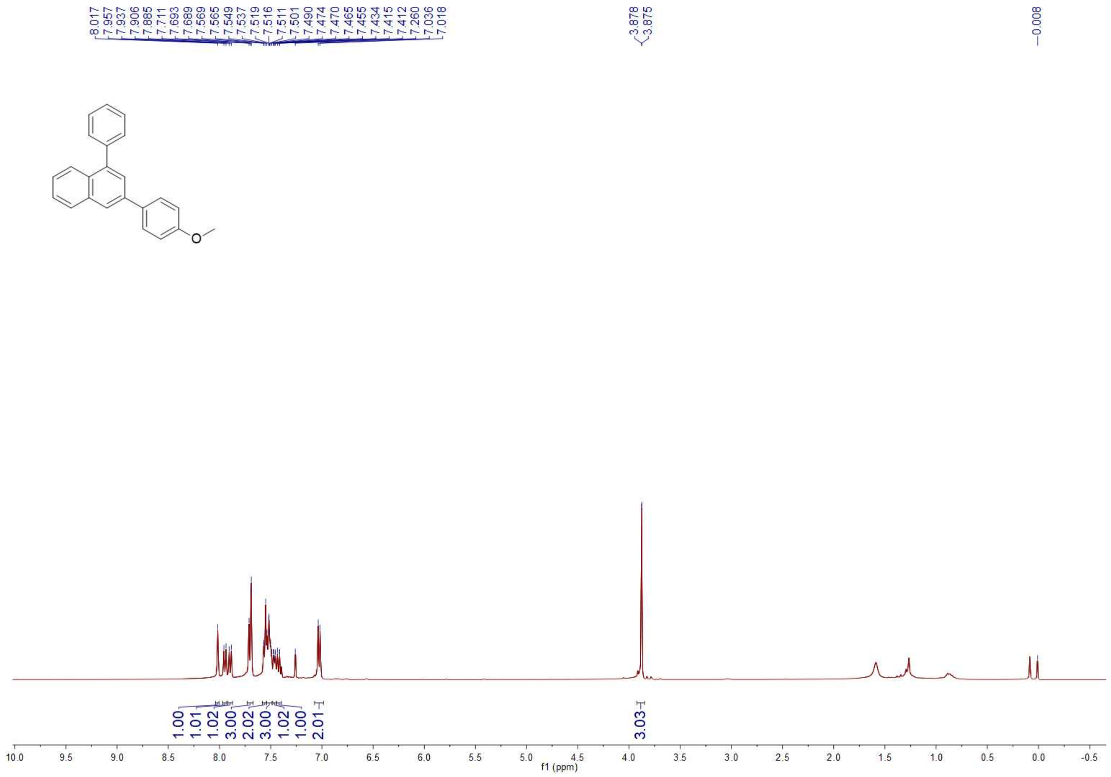

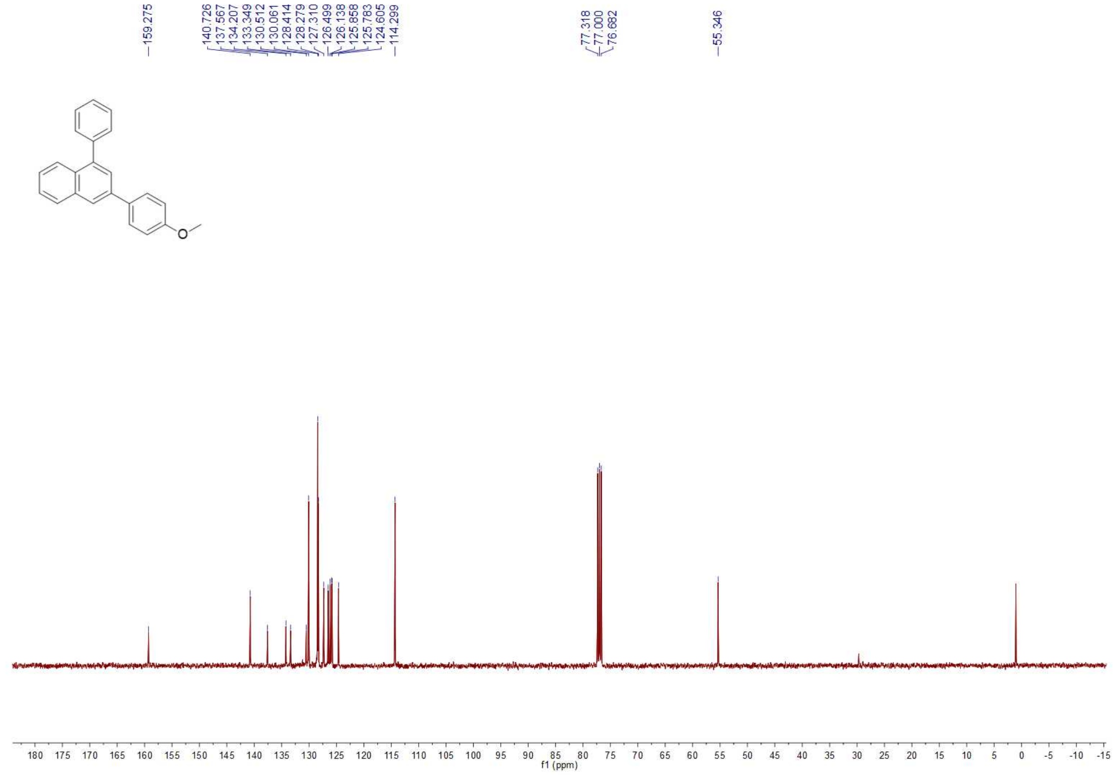

^{1}H NMR (400 MHz, $CDCl_3$) and ^{13}C NMR (100 MHz, $CDCl_3$) Spectrum of **3f**.

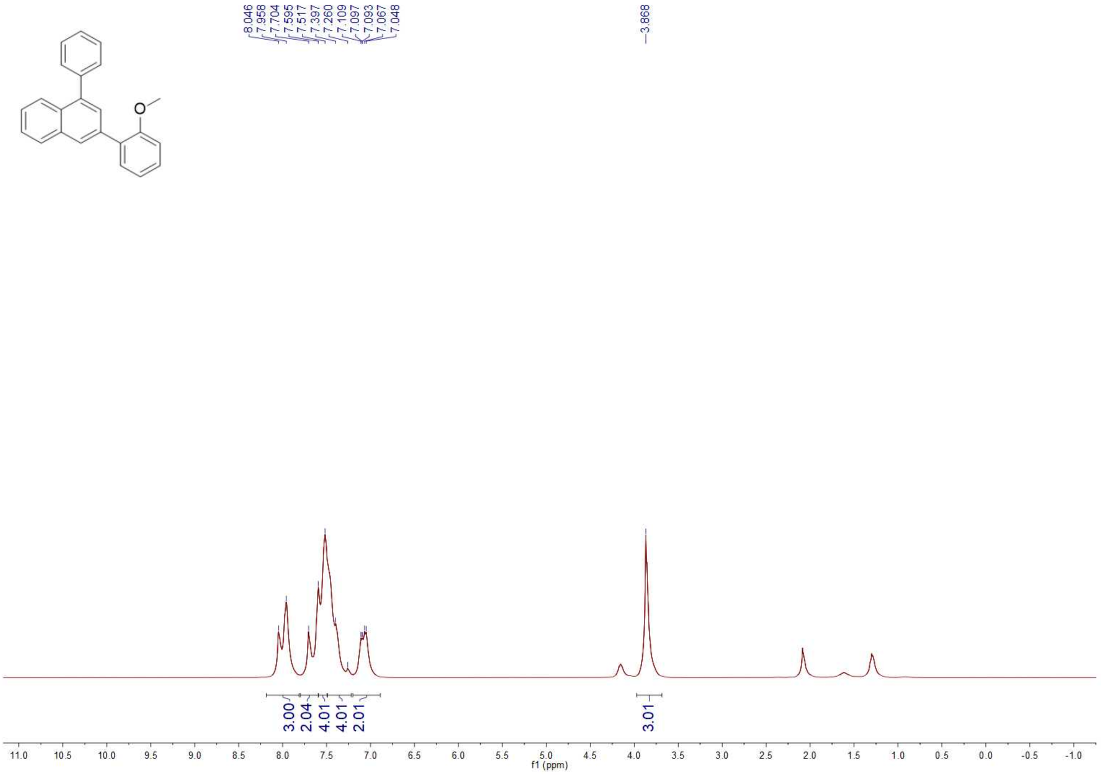

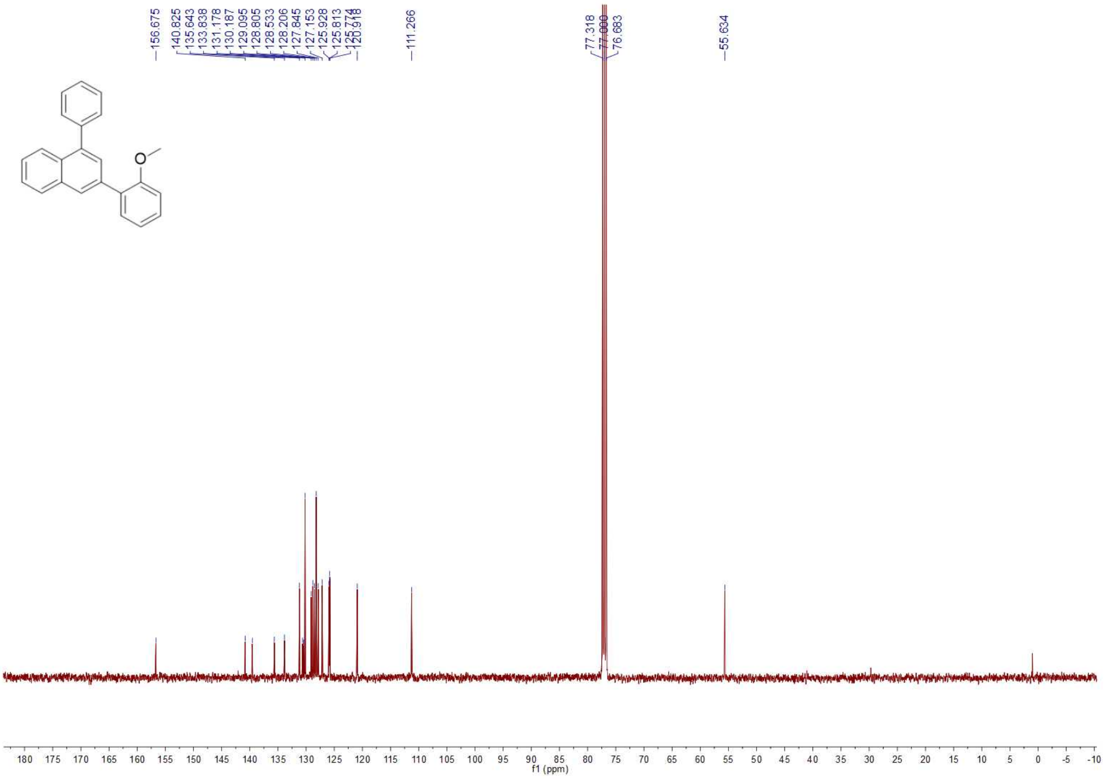

^{1}H NMR (400 MHz, $CDCl_3$) and ^{13}C NMR (100 MHz, $CDCl_3$) Spectrum of **3g**.

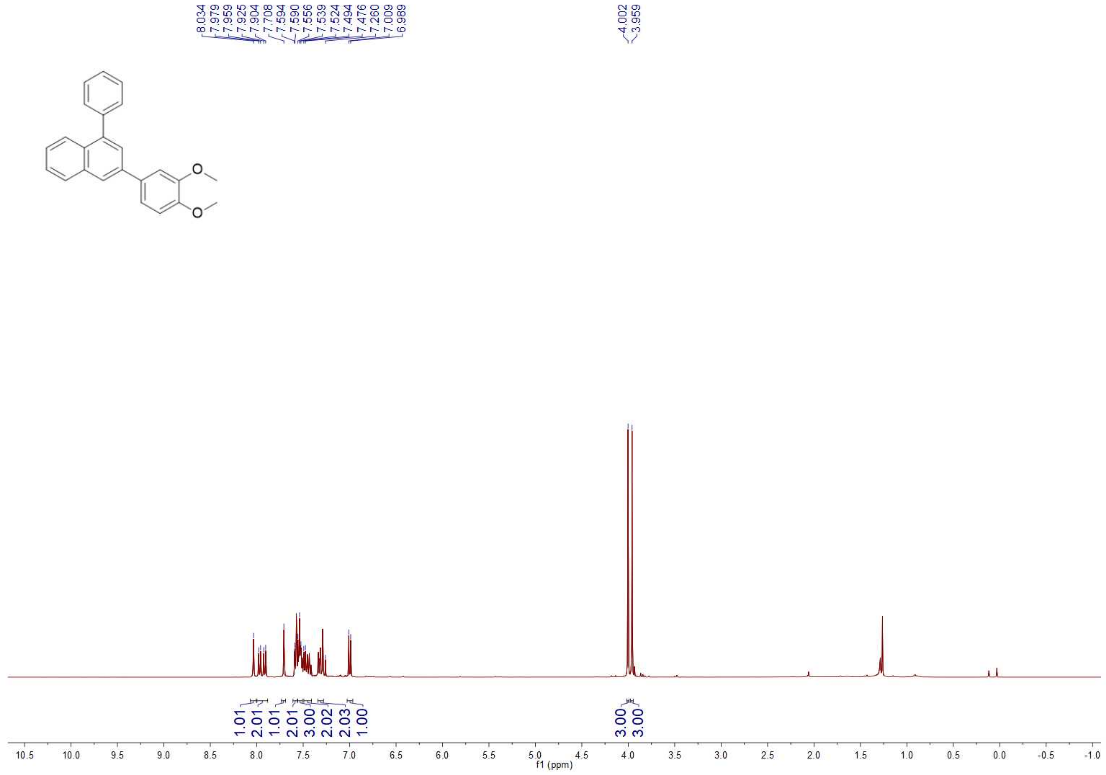

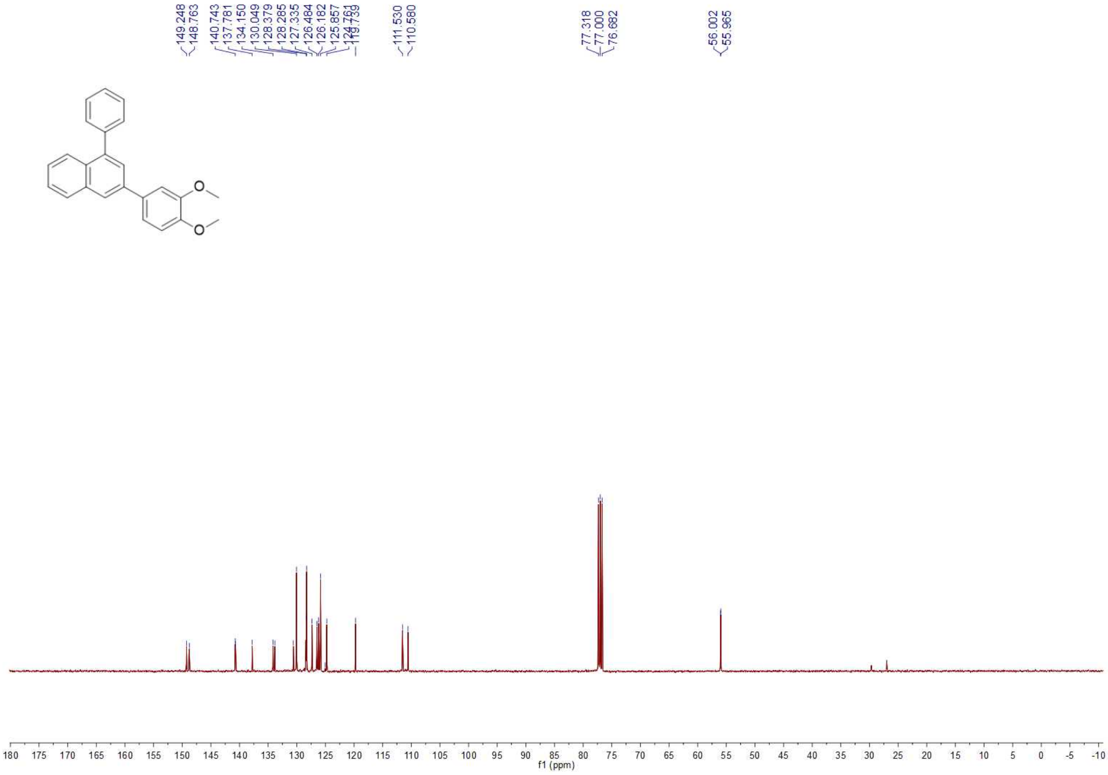

^{1}H NMR (400 MHz, $CDCl_3$) and ^{13}C NMR (100 MHz, $CDCl_3$) Spectrum of **3h**.

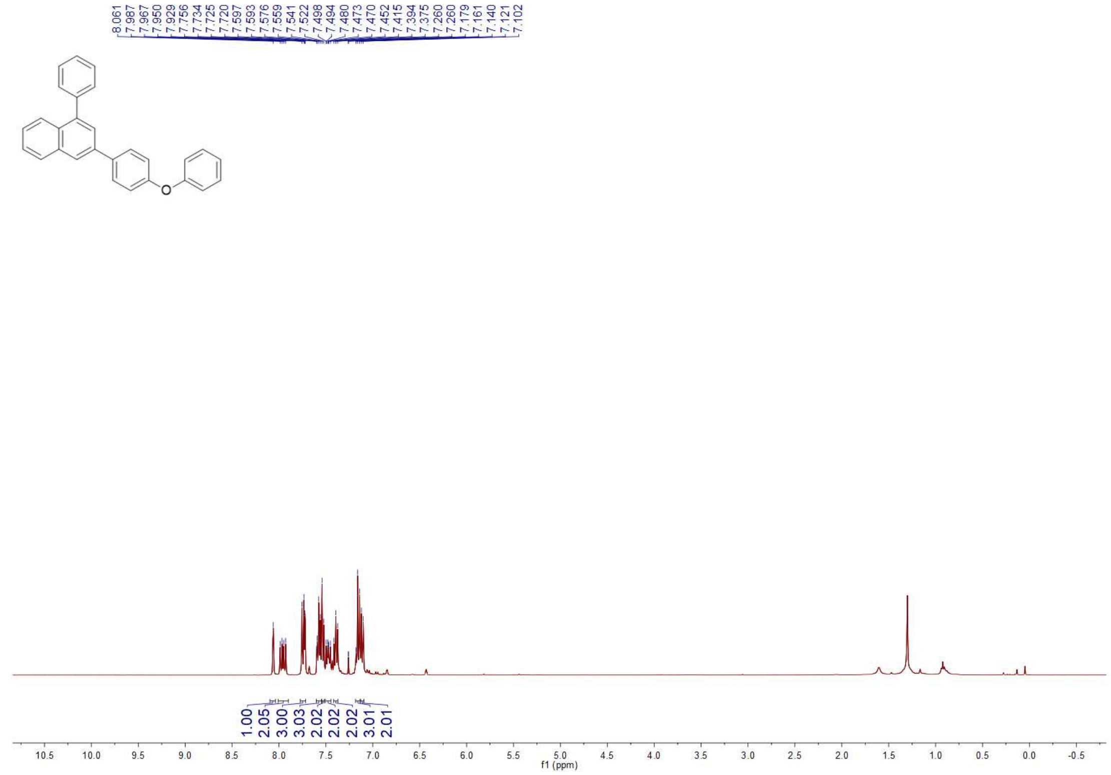

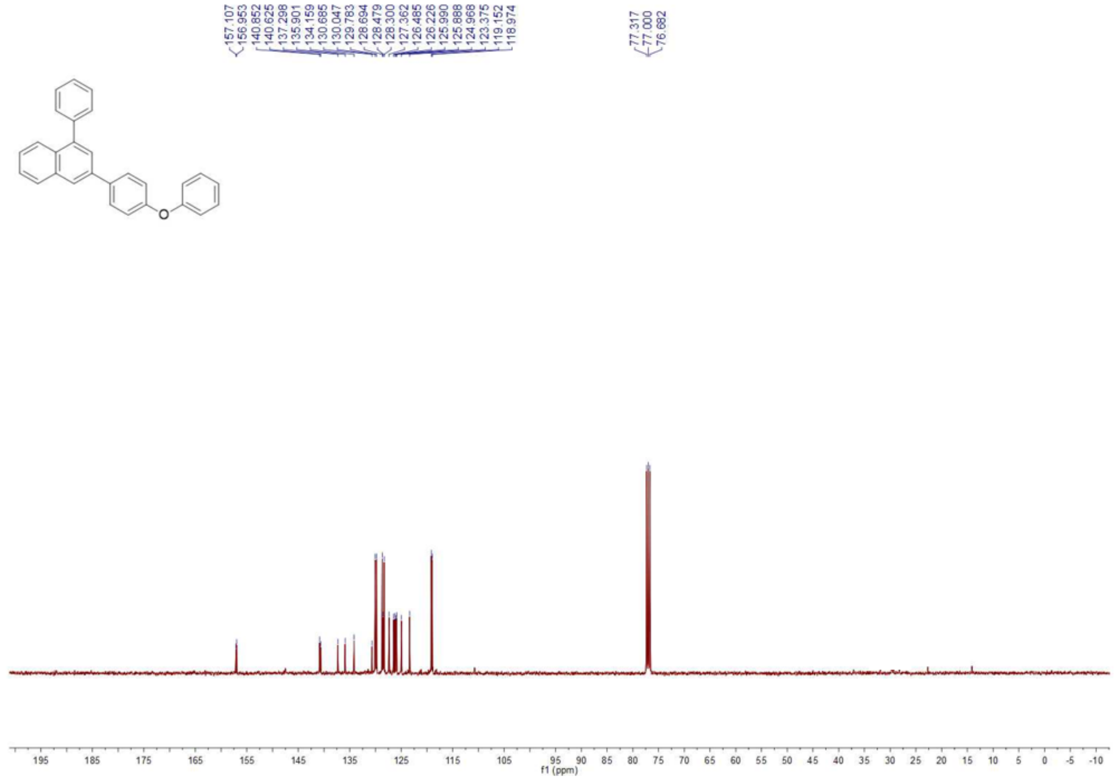

^{1}H NMR (400 MHz, $CDCl_3$) and ^{13}C NMR (100 MHz, $CDCl_3$) Spectrum of **3i**.

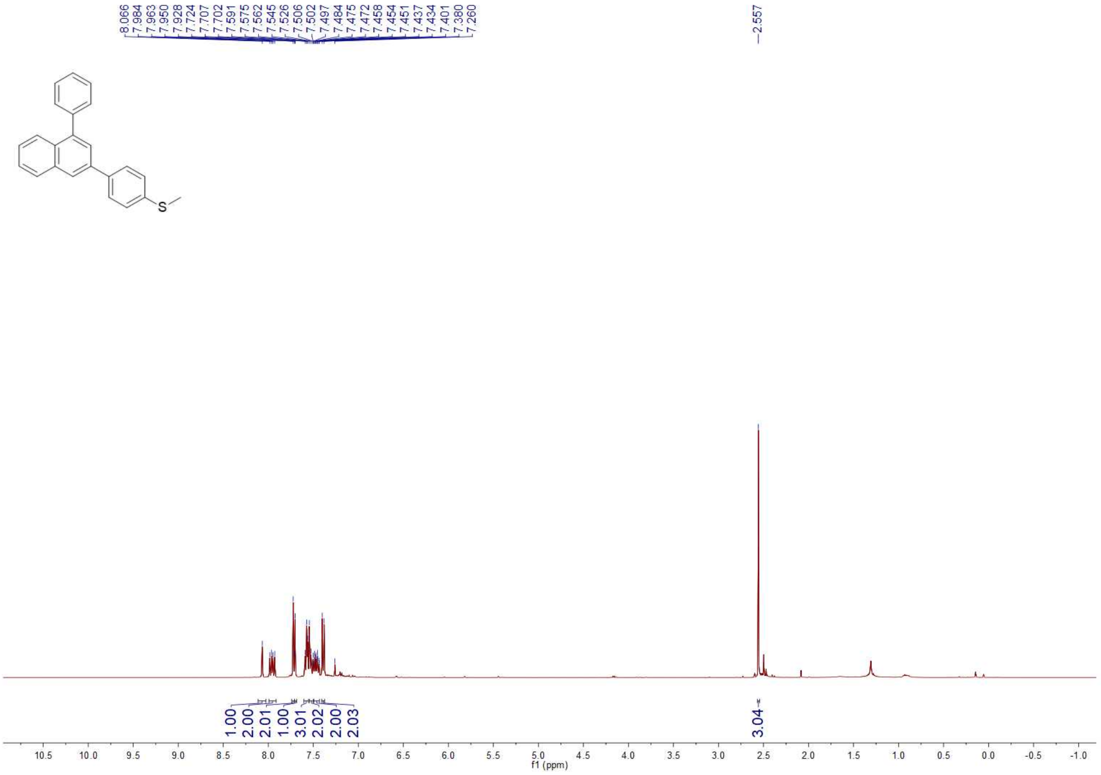

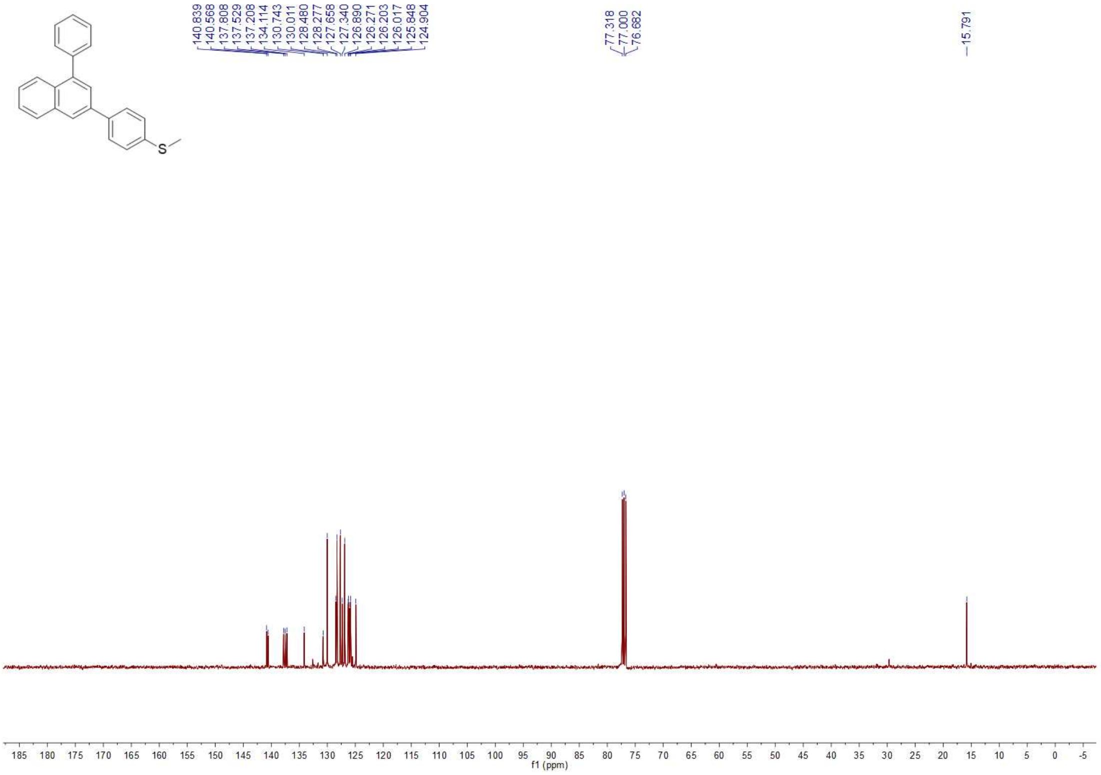

^{1}H NMR (400 MHz, $CDCl_3$) and ^{13}C NMR (100 MHz, $CDCl_3$) Spectrum of **3j**.

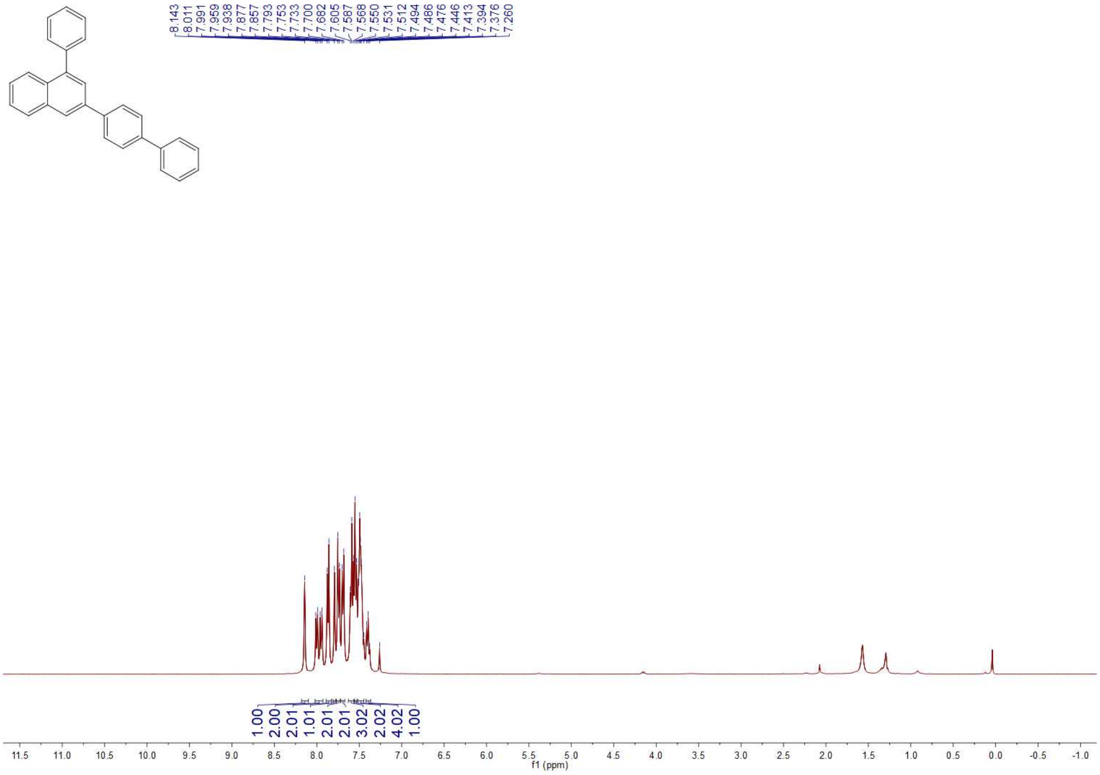

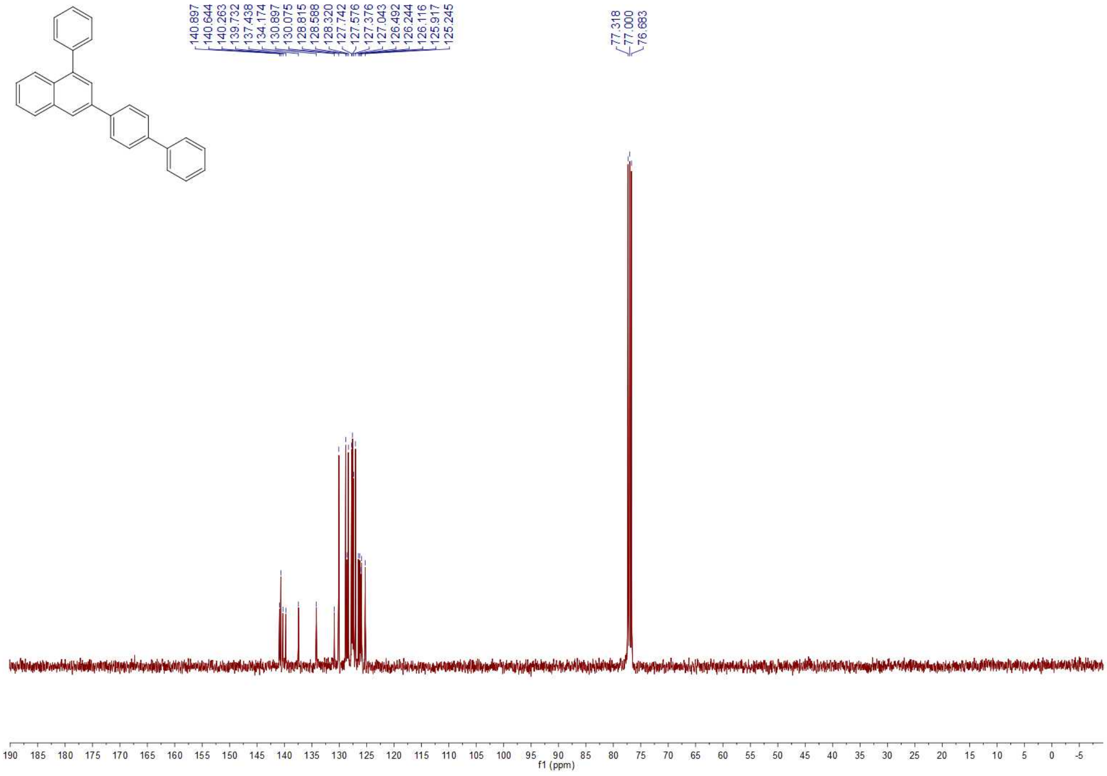

^{1}H NMR (400 MHz, $CDCl_3$), ^{13}C NMR (100 MHz, $CDCl_3$) and ^{19}F NMR (376 MHz, $CDCl_3$) Spectrum of **3k**.

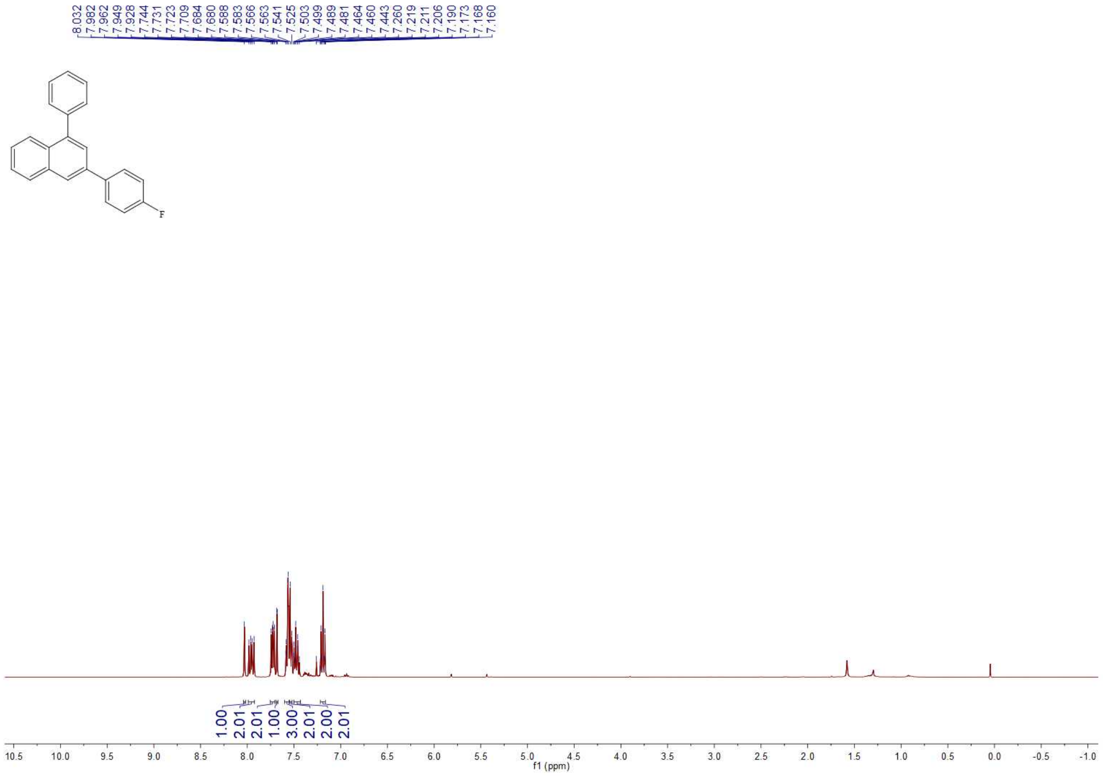

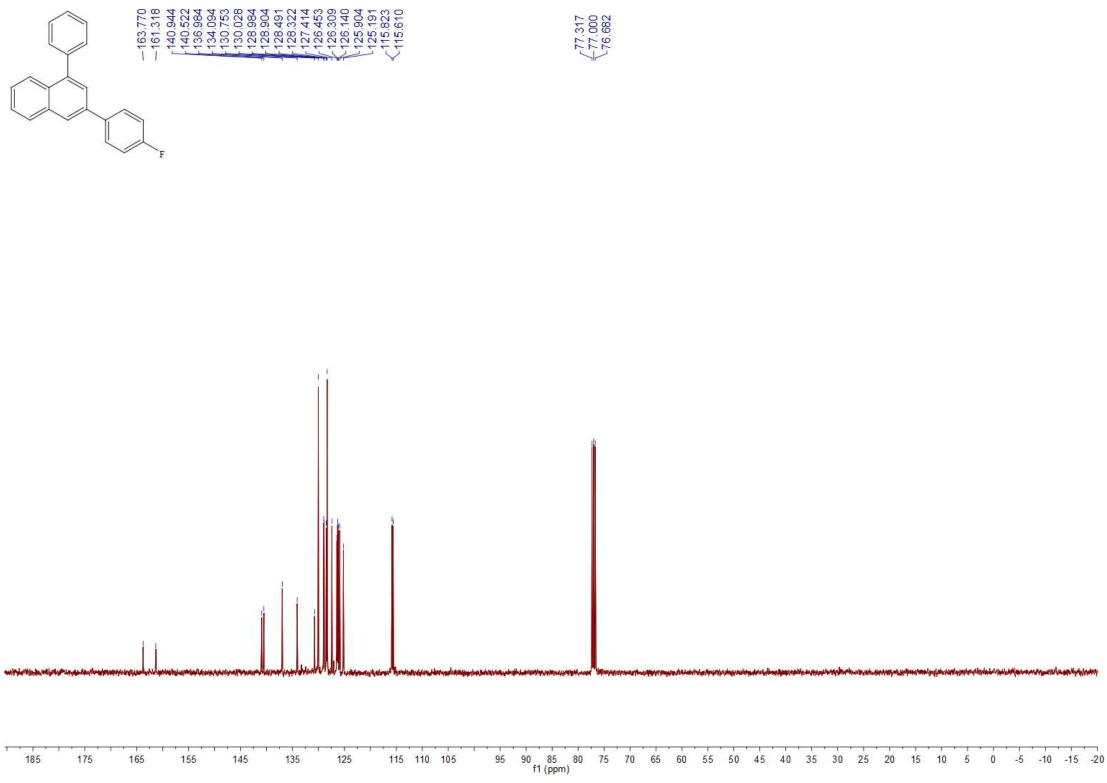

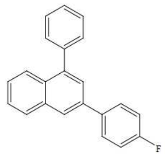

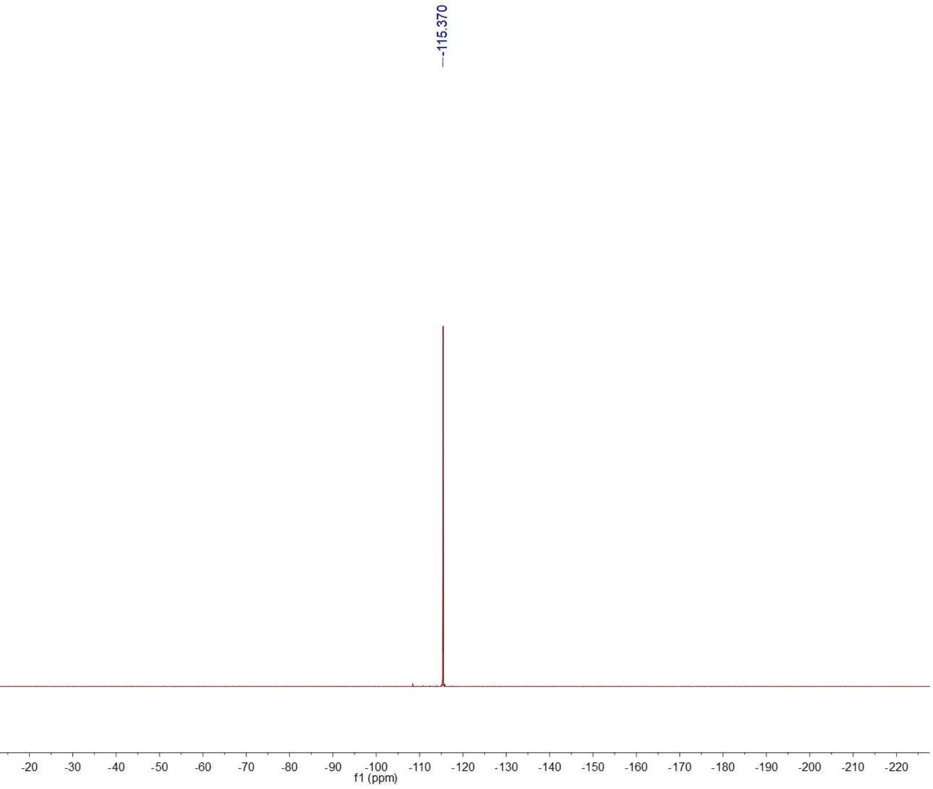
-115.370
20
10
0
-10
-20
-30
-40
-50
-60
-70
-80
-90
-100
-110
-120
-130
-140
-150
-160
-170
-180
-190
-200
-210
-220
f1 (ppm)

^{1}H NMR (400 MHz, $CDCl_3$), ^{13}C NMR (100 MHz, $CDCl_3$) and ^{19}F NMR (376 MHz, $CDCl_3$) Spectrum of **3l**.

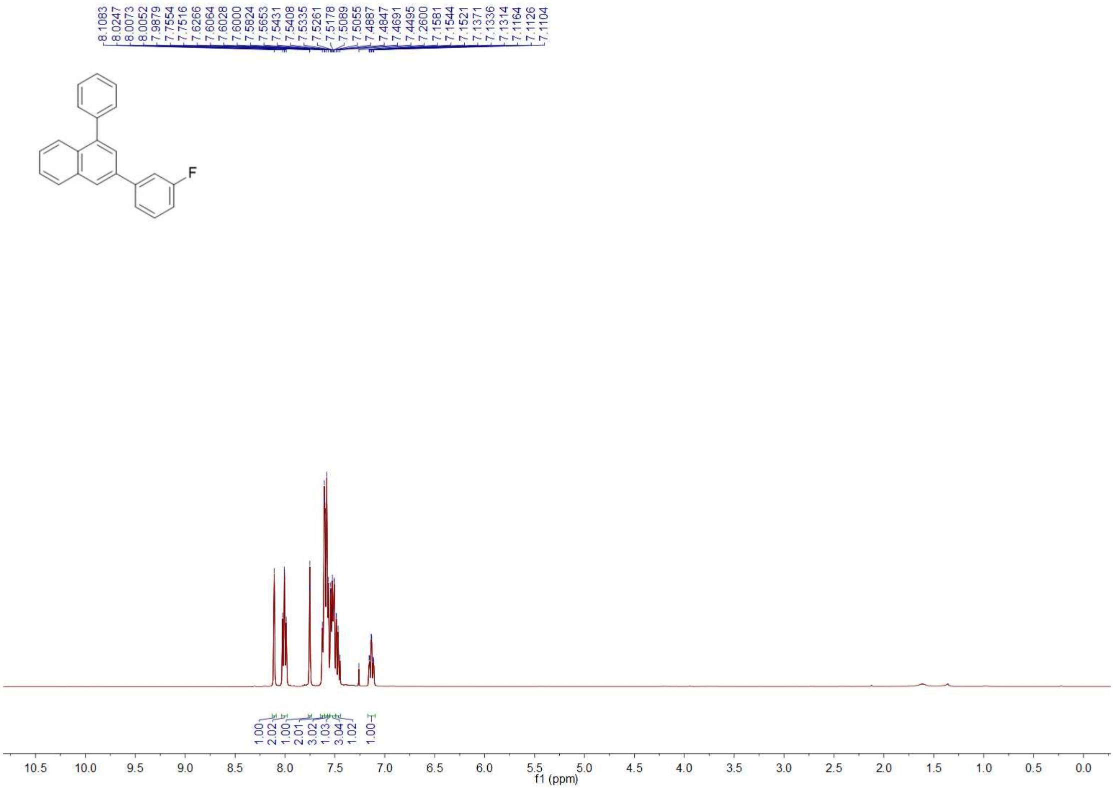

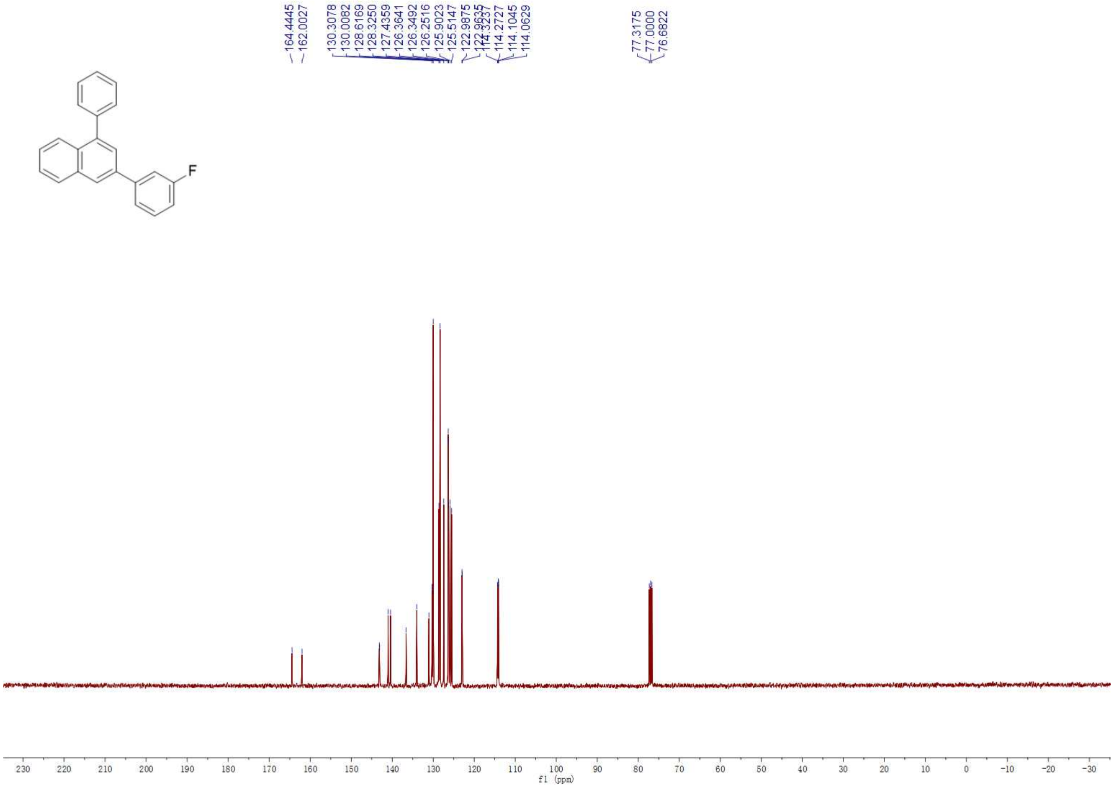

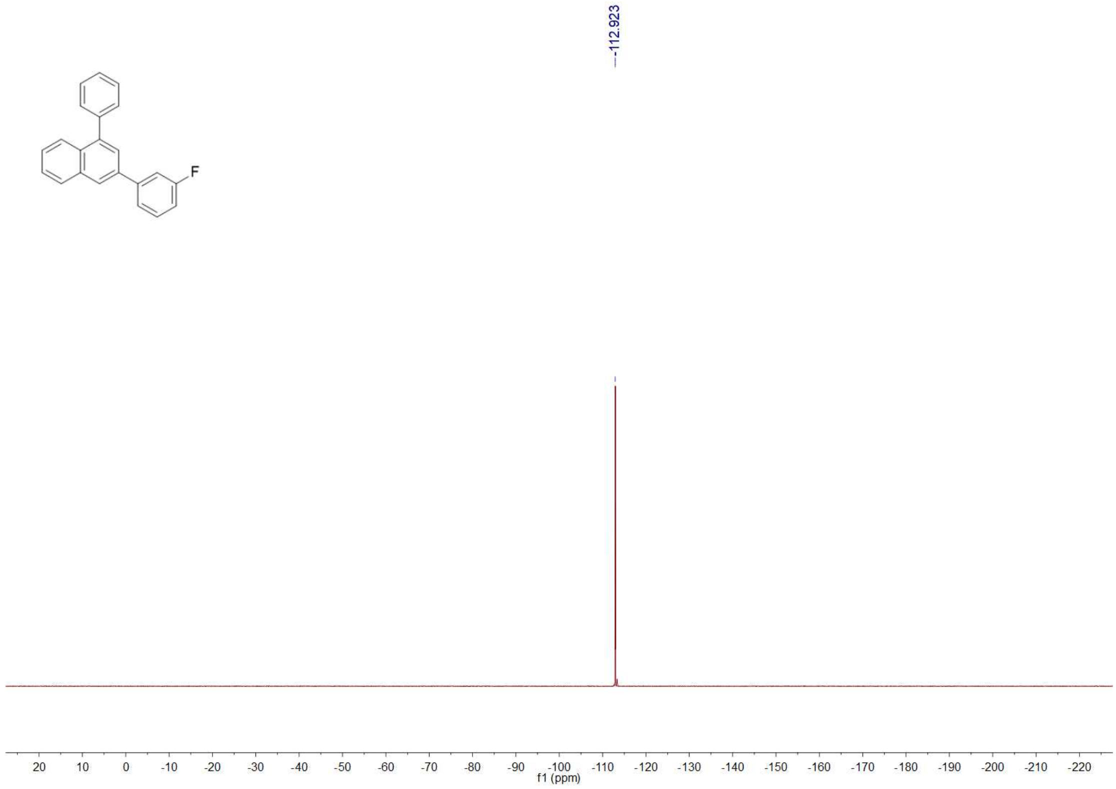

-112.923
F
20 10 0 -10 -20 -30 -40 -50 -60 -70 -80 -90 -100 -110 -120 -130 -140 -150 -160 -170 -180 -190 -200 -210 -220
f1 (ppm)

^{1}H NMR (400 MHz, $CDCl_3$) and ^{13}C NMR (100 MHz, $CDCl_3$) Spectrum of **3m**.

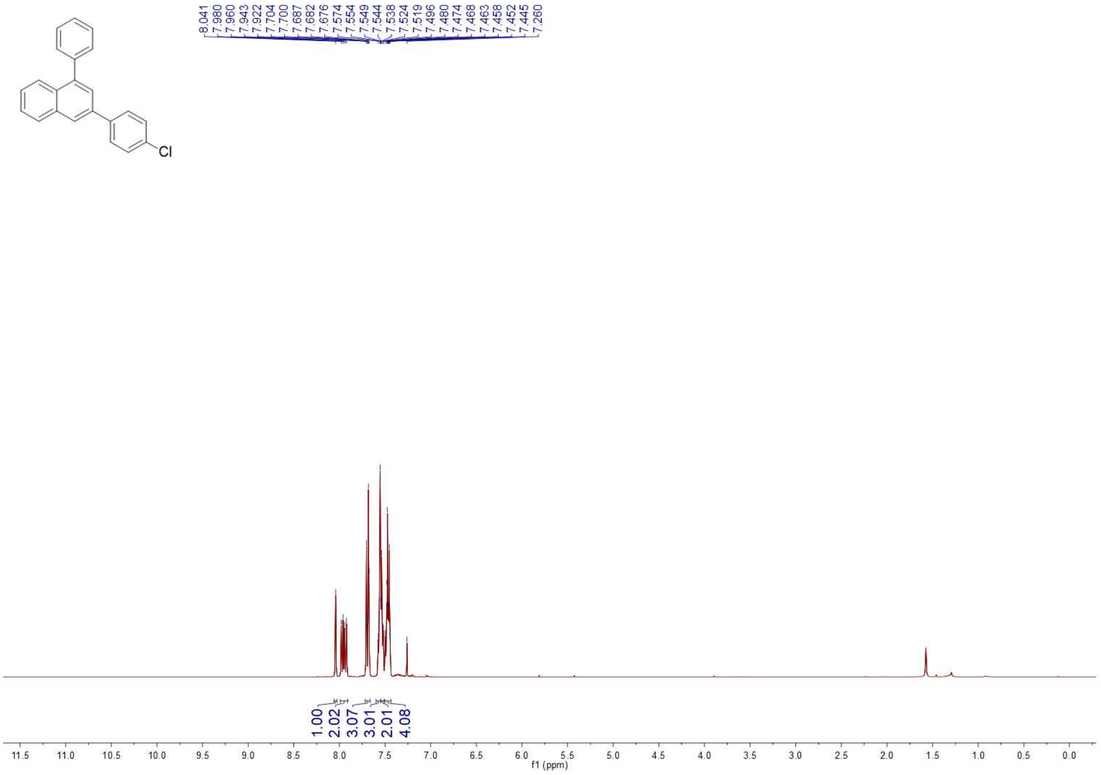

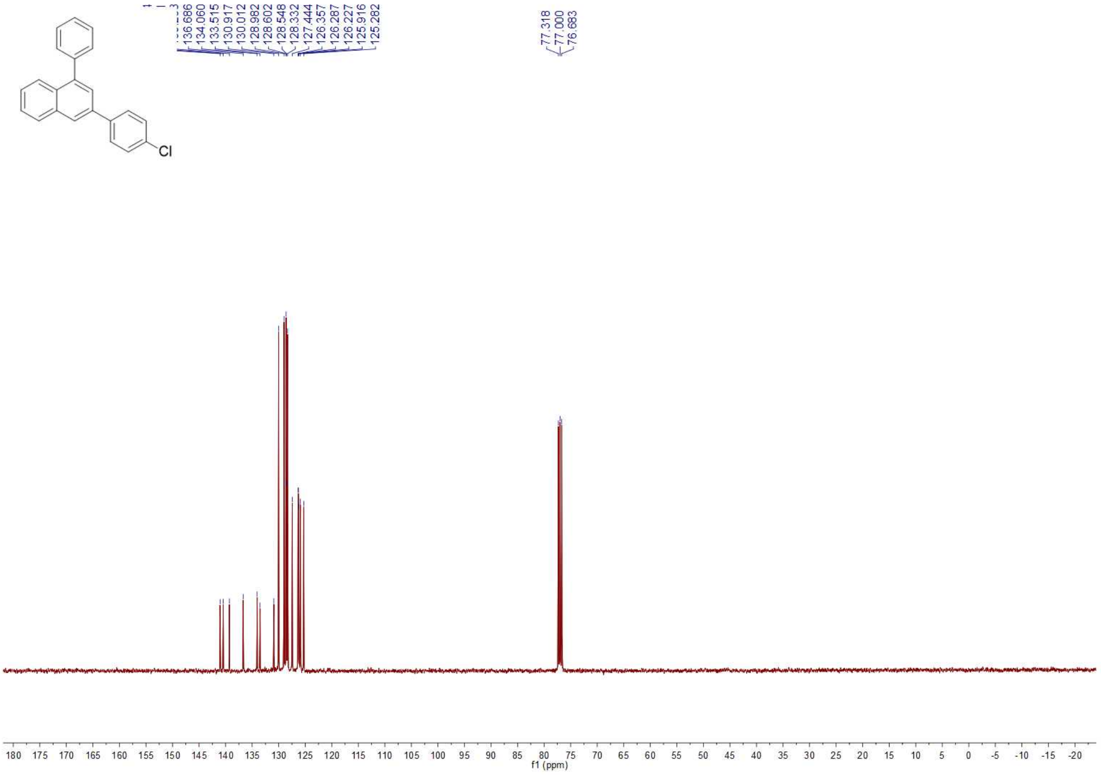

^{1}H NMR (400 MHz, $CDCl_3$) and ^{13}C NMR (100 MHz, $CDCl_3$) Spectrum of **3n**.

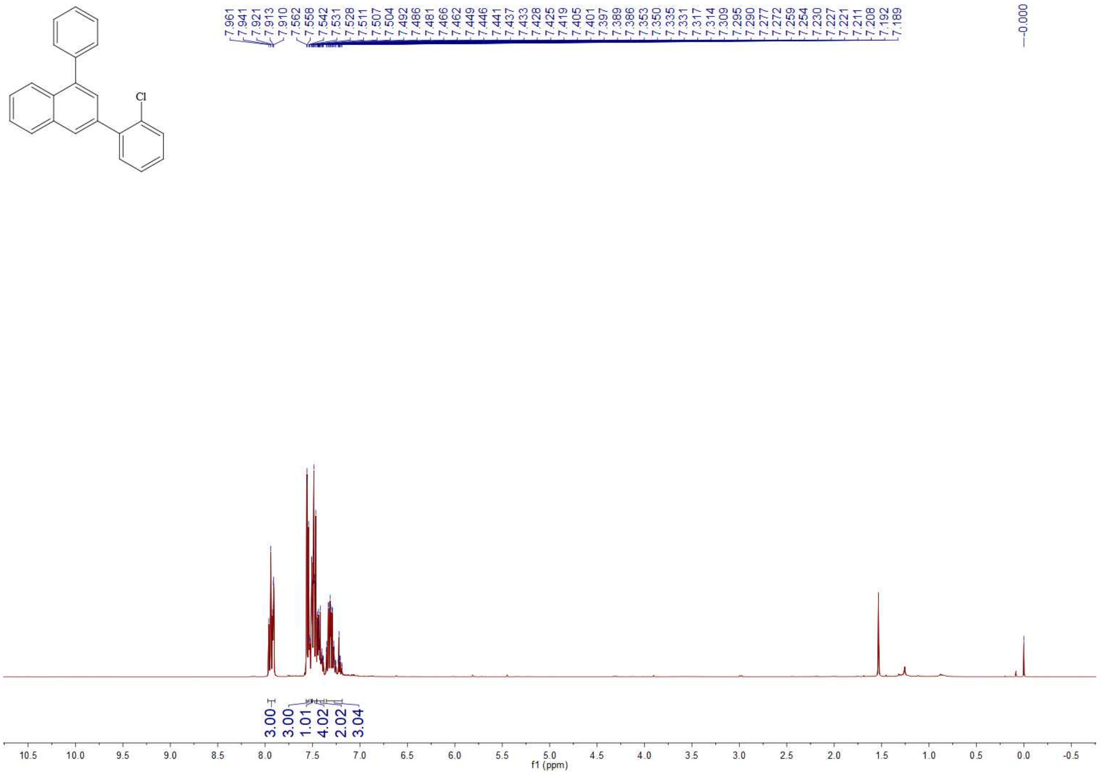

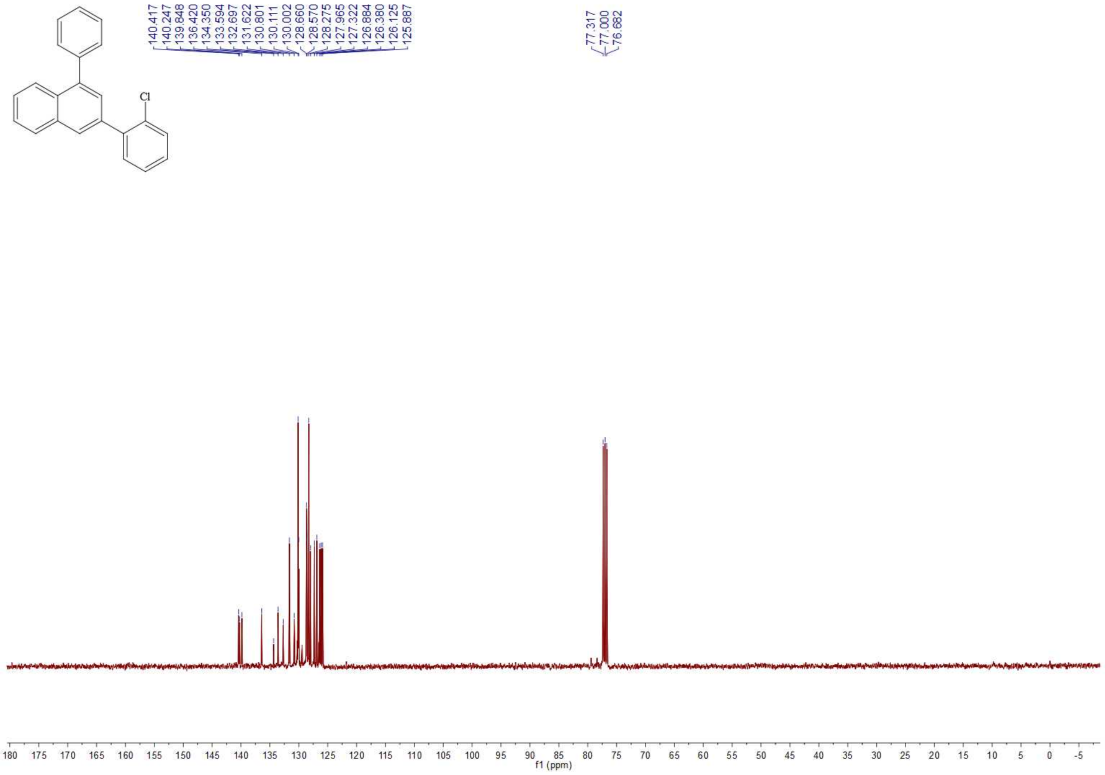

^{1}H NMR (400 MHz, $CDCl_3$) and ^{13}C NMR (100 MHz, $CDCl_3$) Spectrum of **3o**.

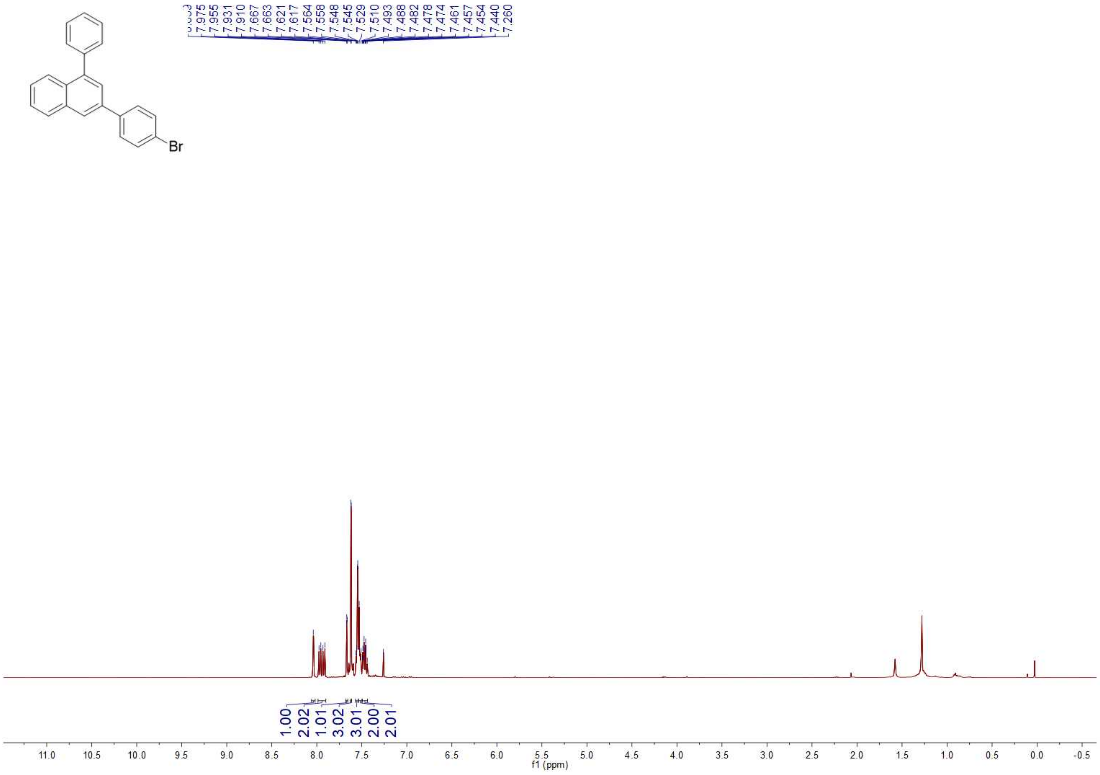

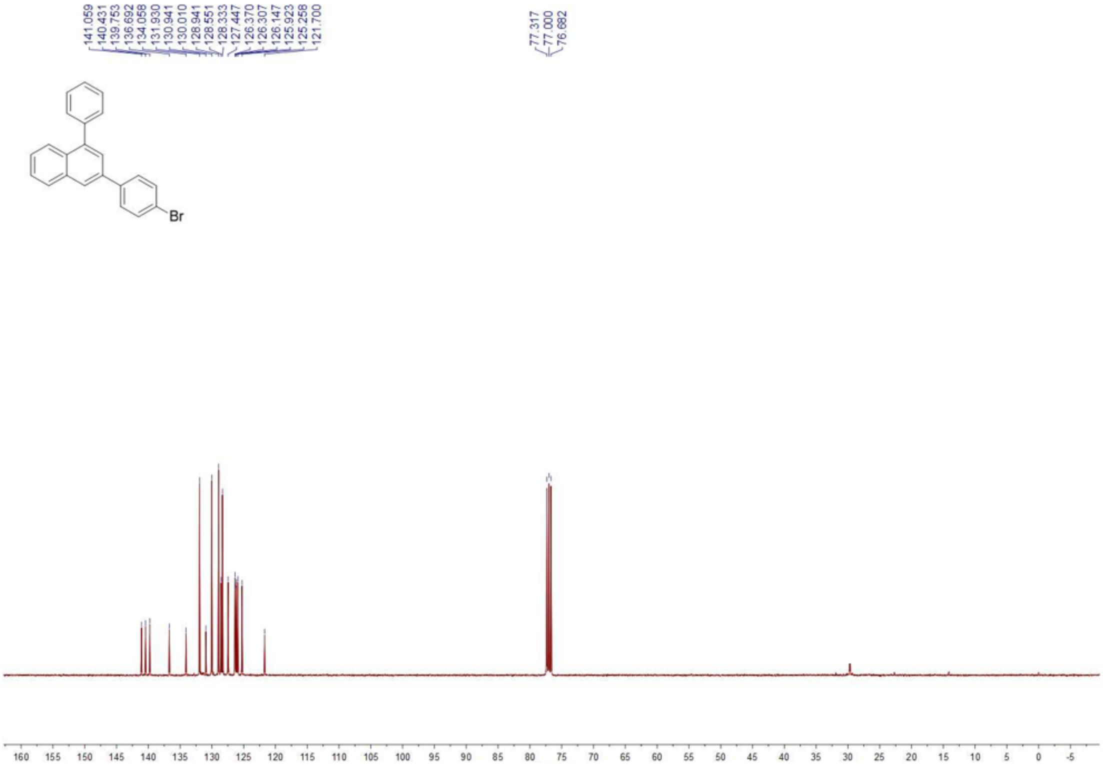

^{1}H NMR (400 MHz, $CDCl_3$), ^{13}C NMR (100 MHz, $CDCl_3$) and ^{19}F NMR (376 MHz, $CDCl_3$) Spectrum of **3p**.

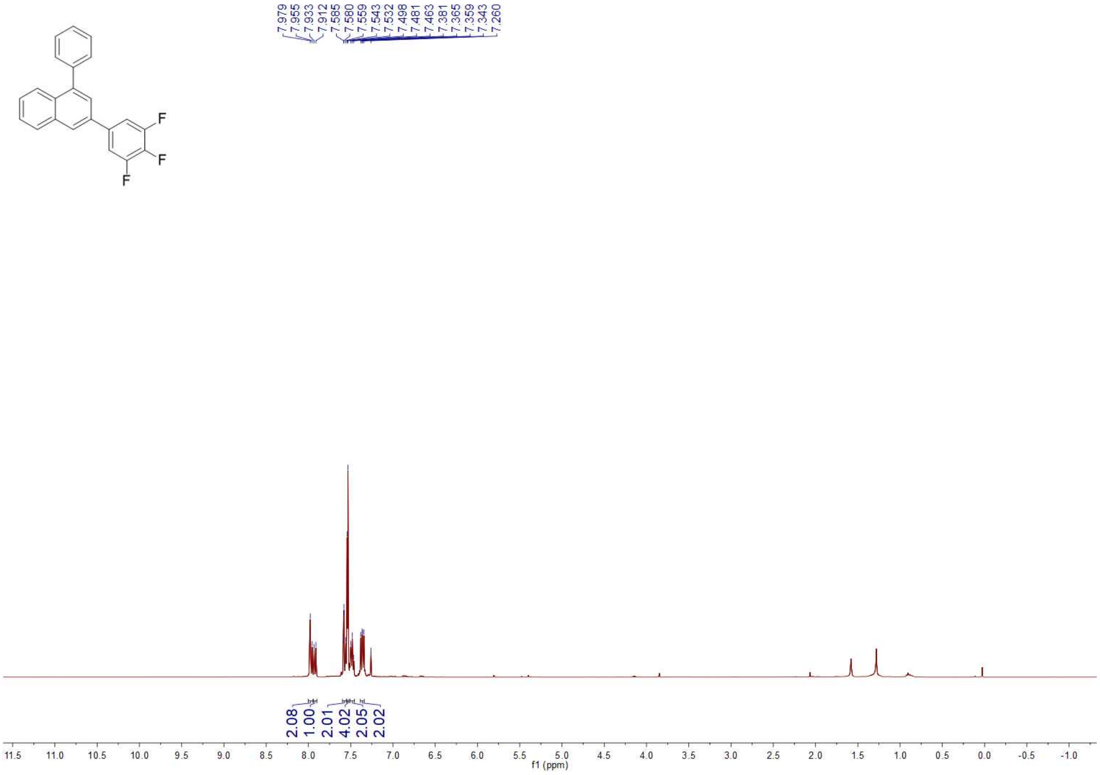

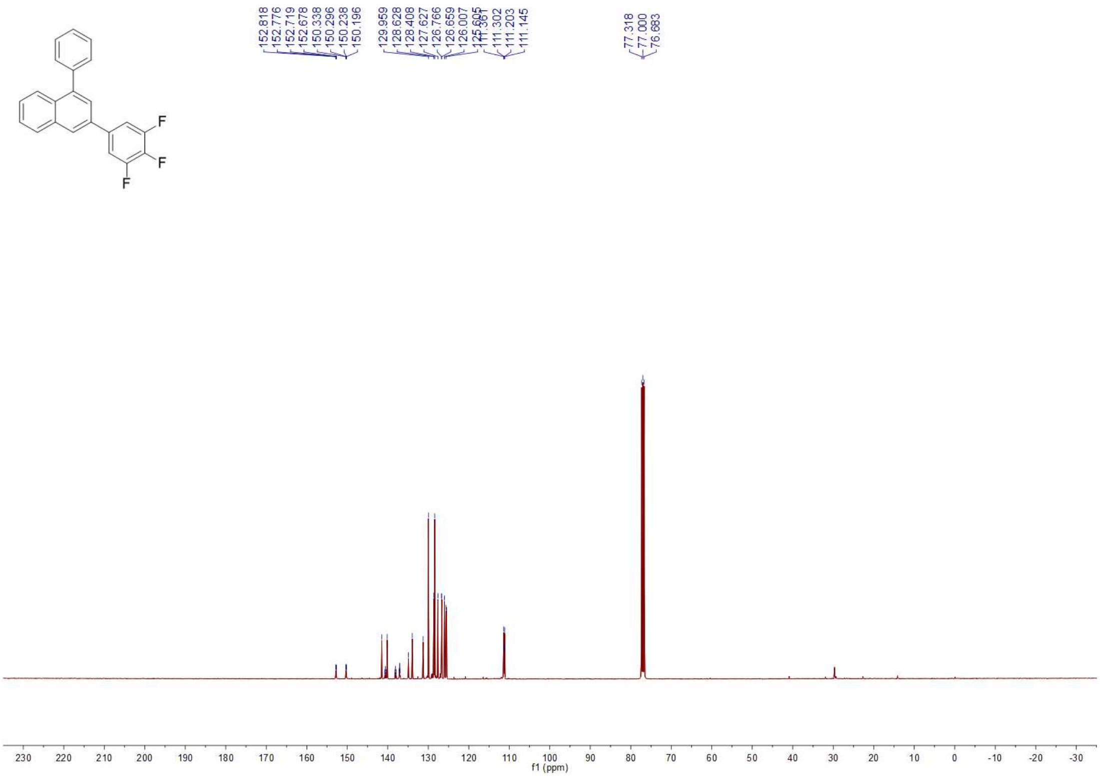

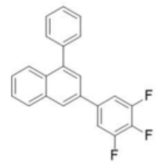

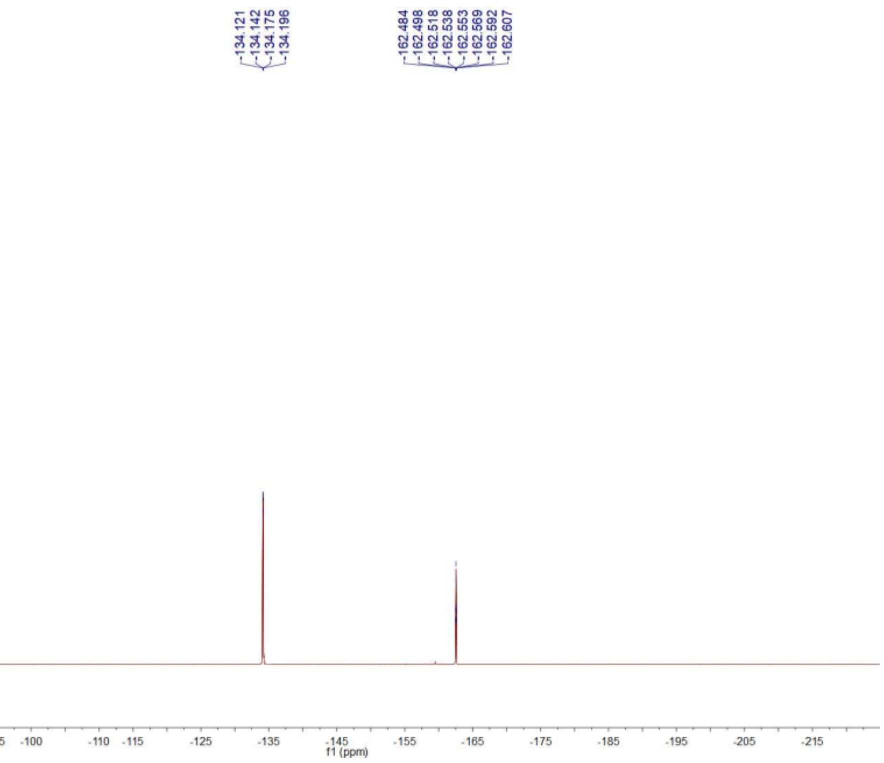
-134.121
-134.142
-134.175
-134.196
-162.484
-162.498
-162.518
-162.538
-162.553
-162.569
-162.592
-162.607
-70 -75 -80 -85 -90 -95 -100 -110 -115 -125 -135 -145 -155 -165 -175 -185 -195 -205 -215
f1 (ppm)

^{1}H NMR (400 MHz, $CDCl_3$), ^{13}C NMR (100 MHz, $CDCl_3$) and ^{19}F NMR (376 MHz, $CDCl_3$) Spectrum of **3q**.

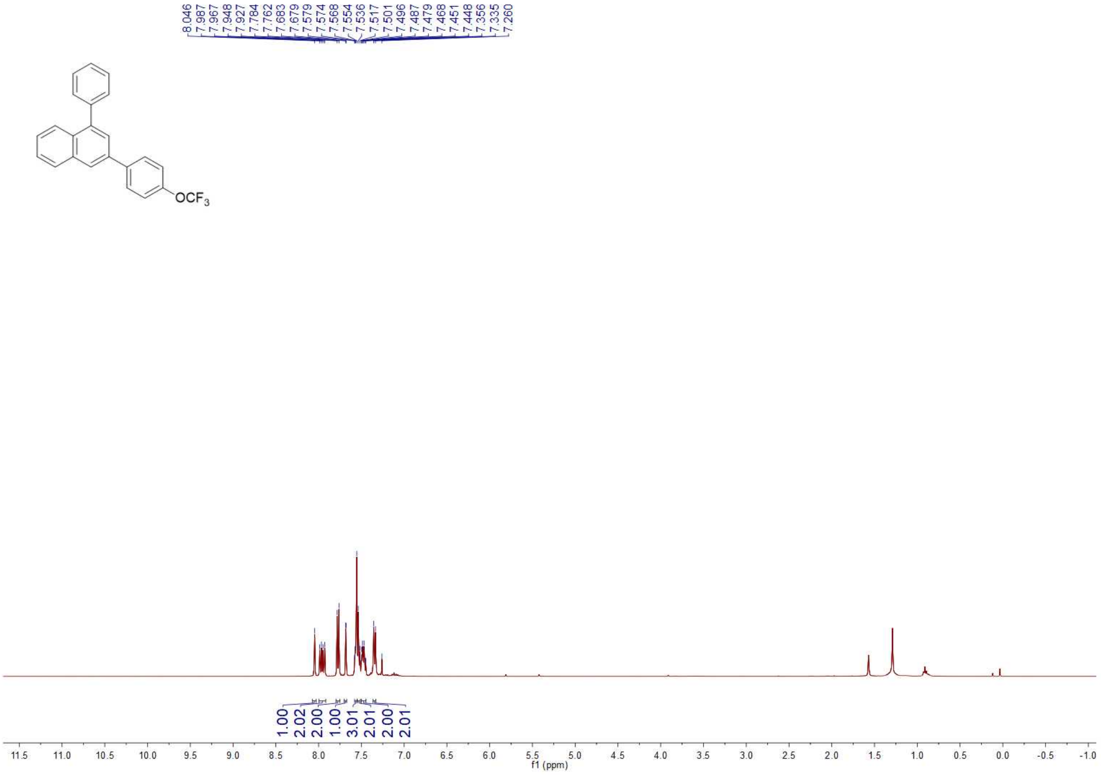

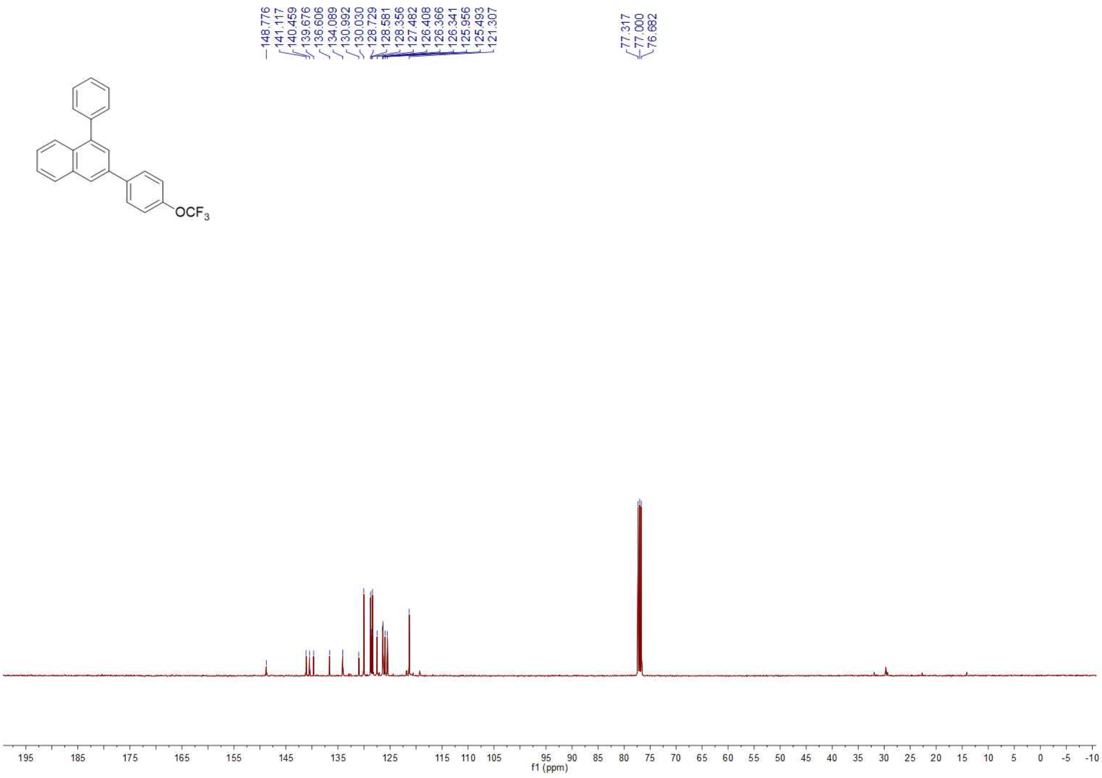

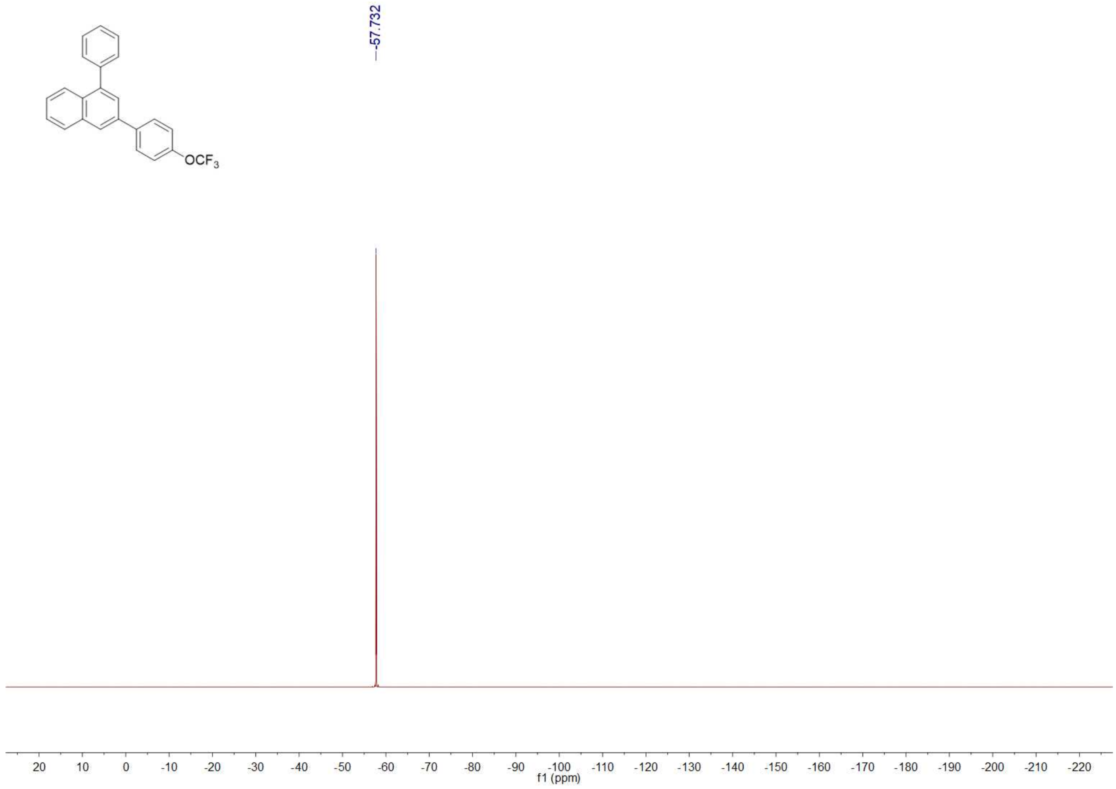

^{1}H NMR (400 MHz, $CDCl_3$), ^{13}C NMR (100 MHz, $CDCl_3$) and ^{19}F NMR (376 MHz, $CDCl_3$) Spectrum of **3r**.

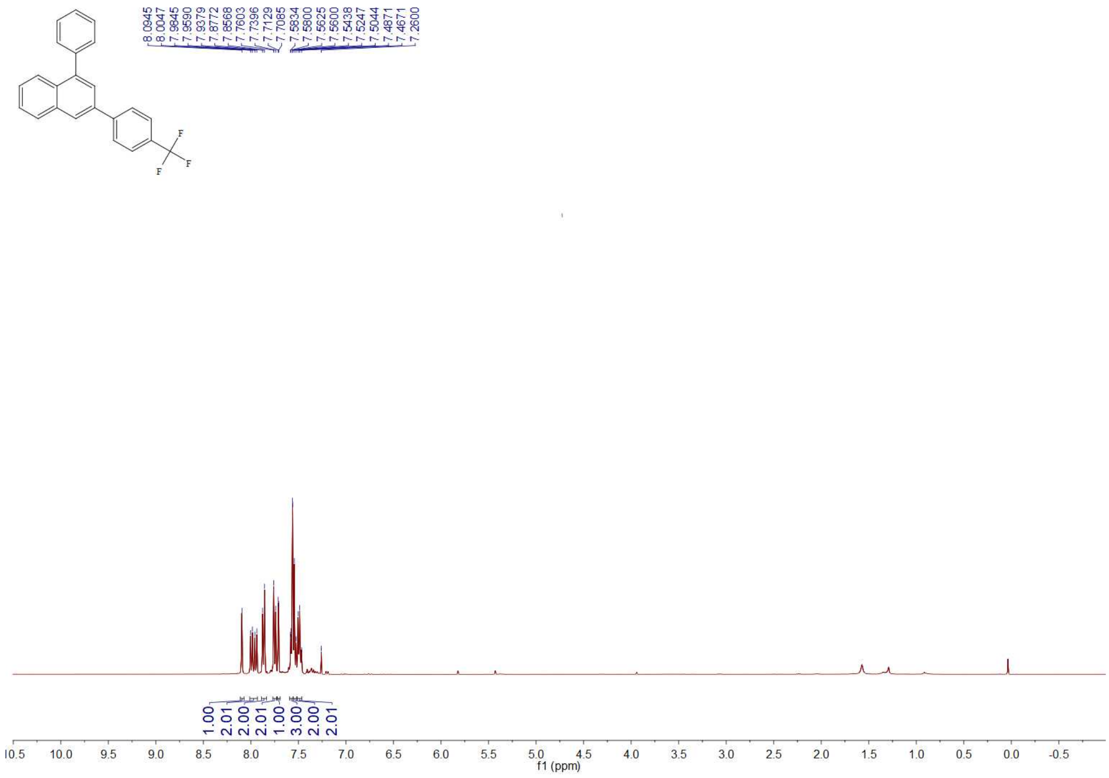

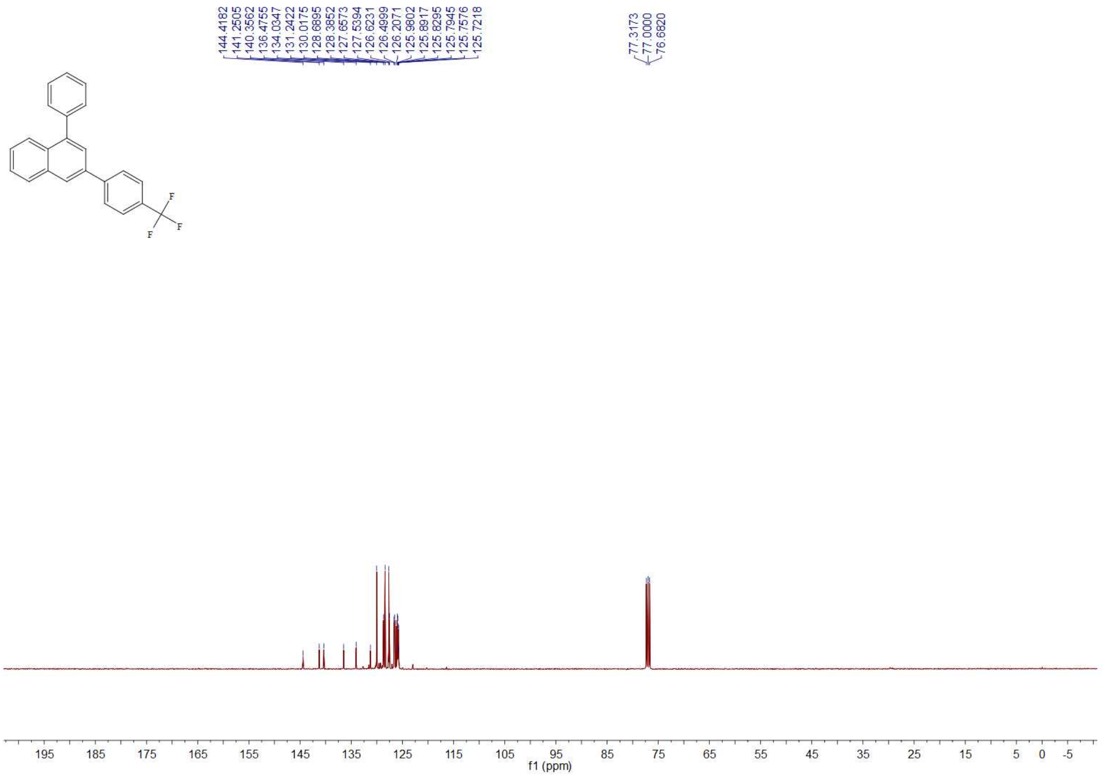
144.4182
141.2505
140.3562
136.4755
134.0347
131.2422
130.0175
128.6895
128.3852
127.5394
126.6231
126.4999
126.2071
125.9802
125.8917
125.8295
125.7945
125.7576
125.7218
77.3173
77.0000
76.6820
F
F
F
f1 (ppm)

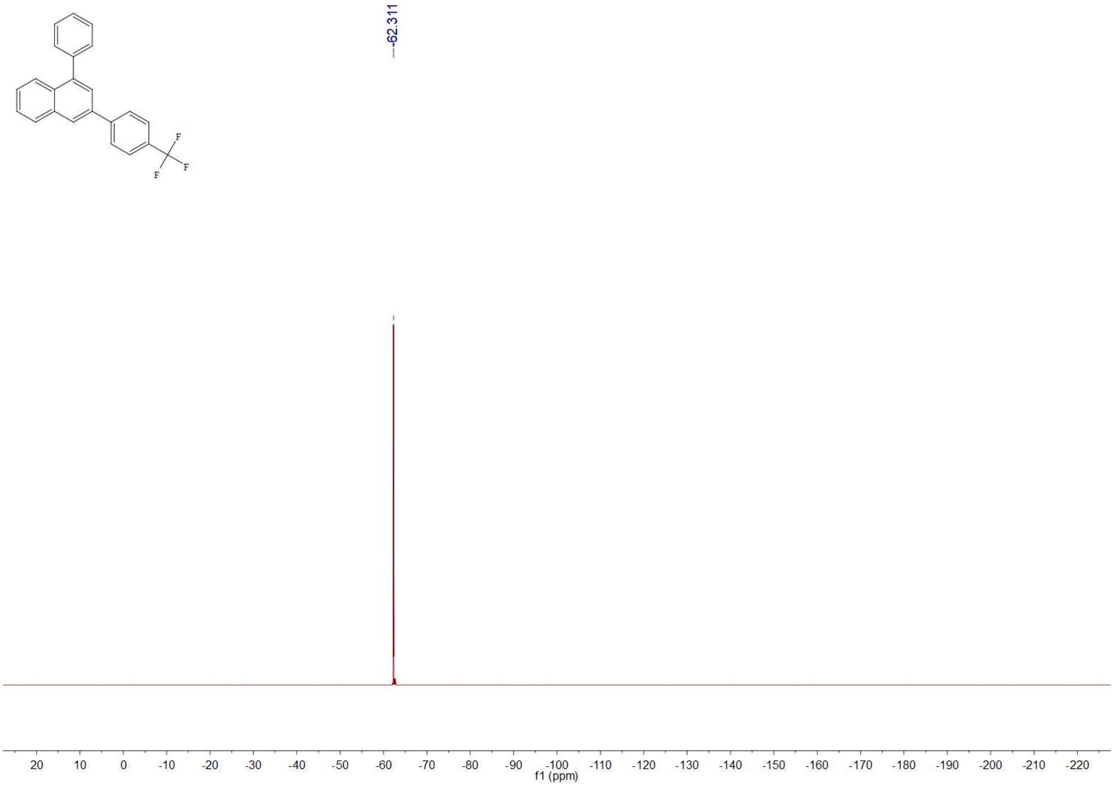
-62.311
F
F
F
f1 (ppm)

^{1}H NMR (400 MHz, $CDCl_3$) and ^{13}C NMR (100 MHz, $CDCl_3$) Spectrum of **3s**.

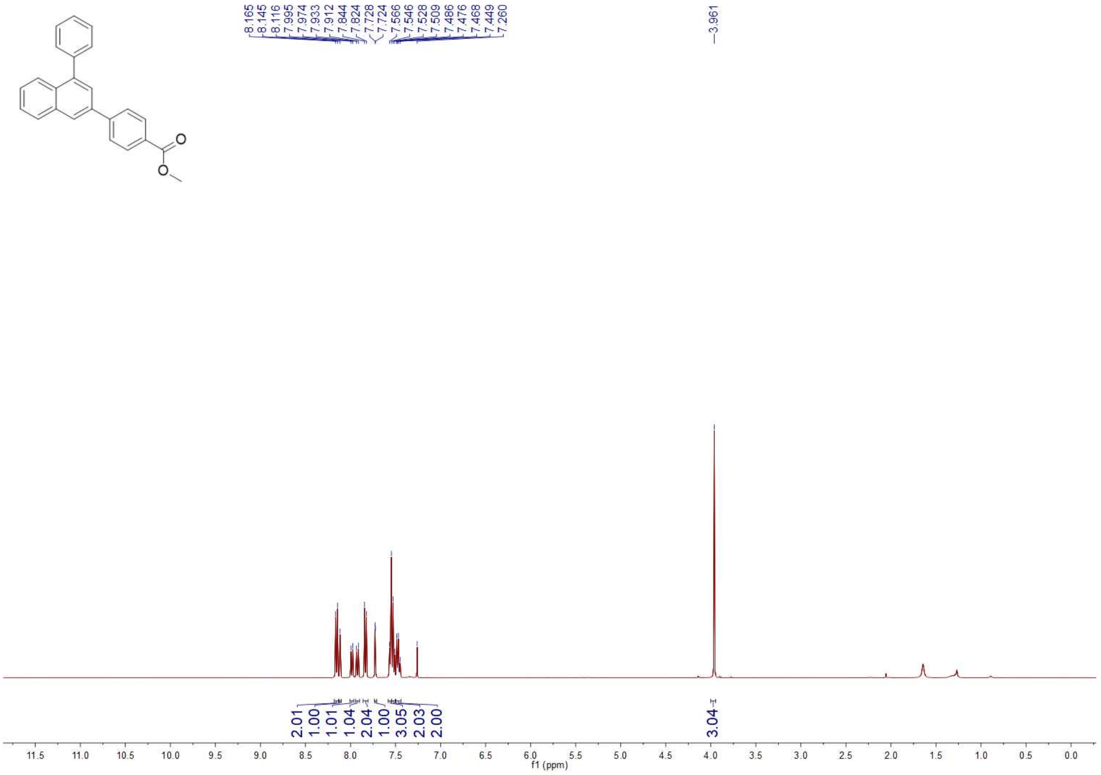

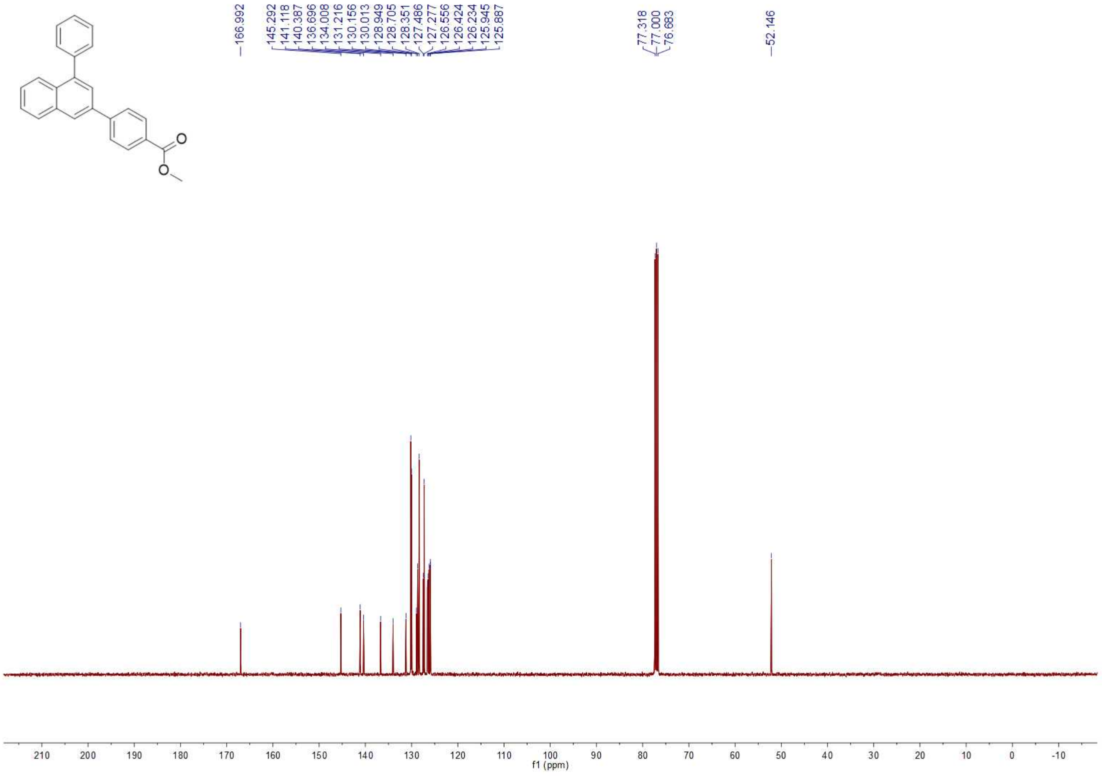

^{1}H NMR (400 MHz, $CDCl_3$) and ^{13}C NMR (100 MHz, $CDCl_3$) Spectrum of **3t**.

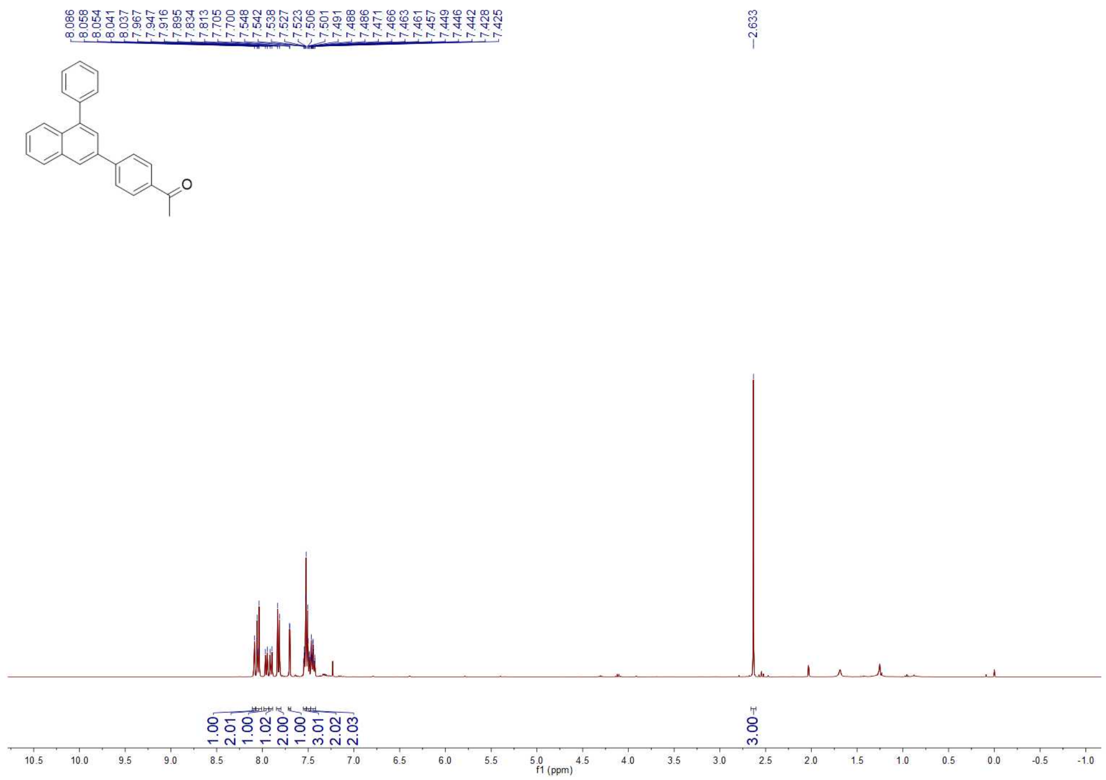

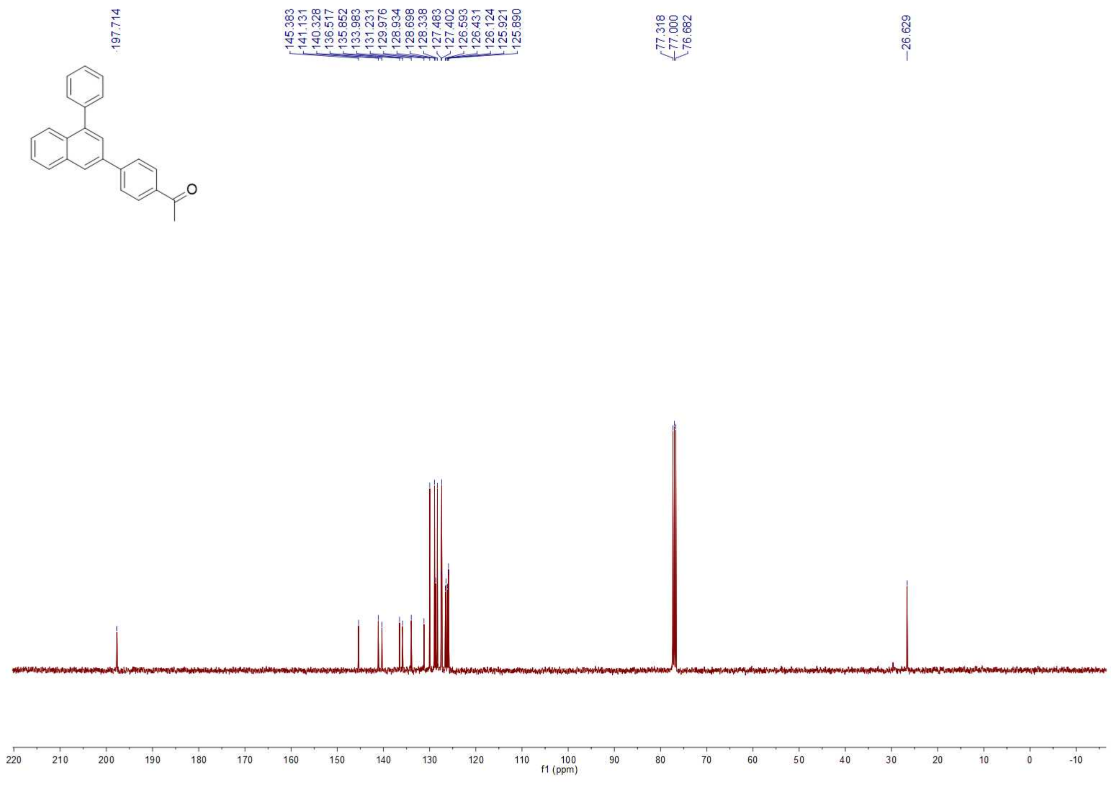

^{1}H NMR (400 MHz, $CDCl_3$) and ^{13}C NMR (100 MHz, $CDCl_3$) Spectrum of **3u**.

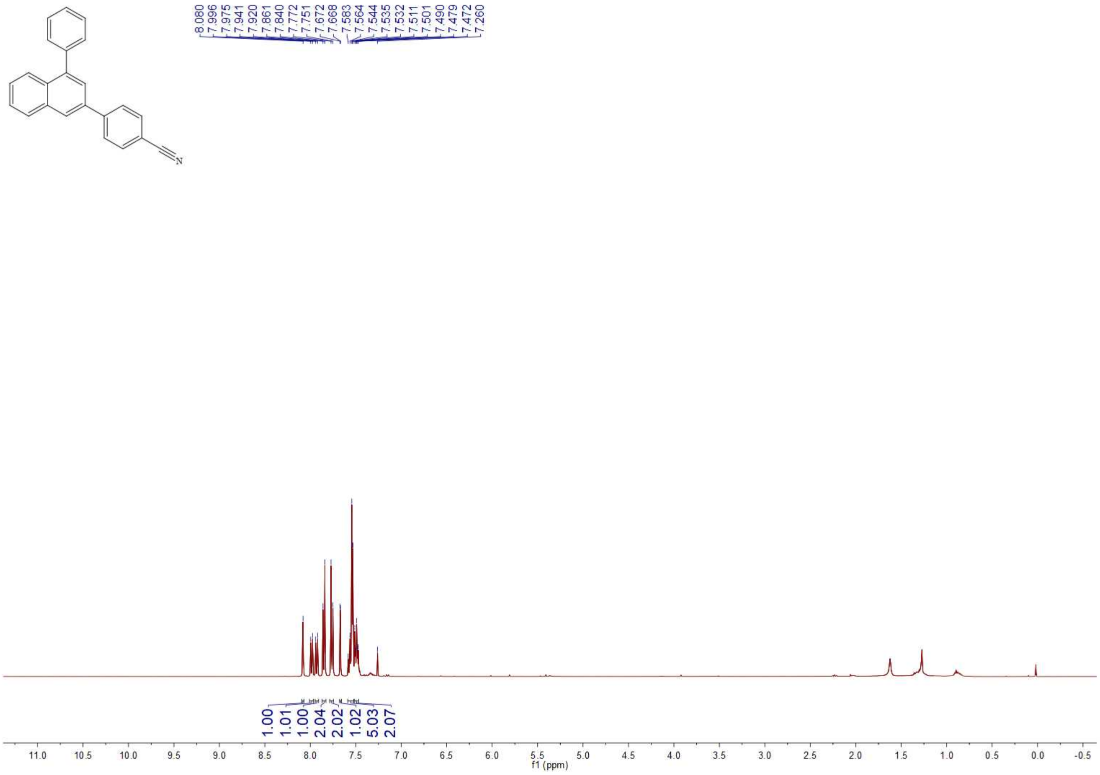

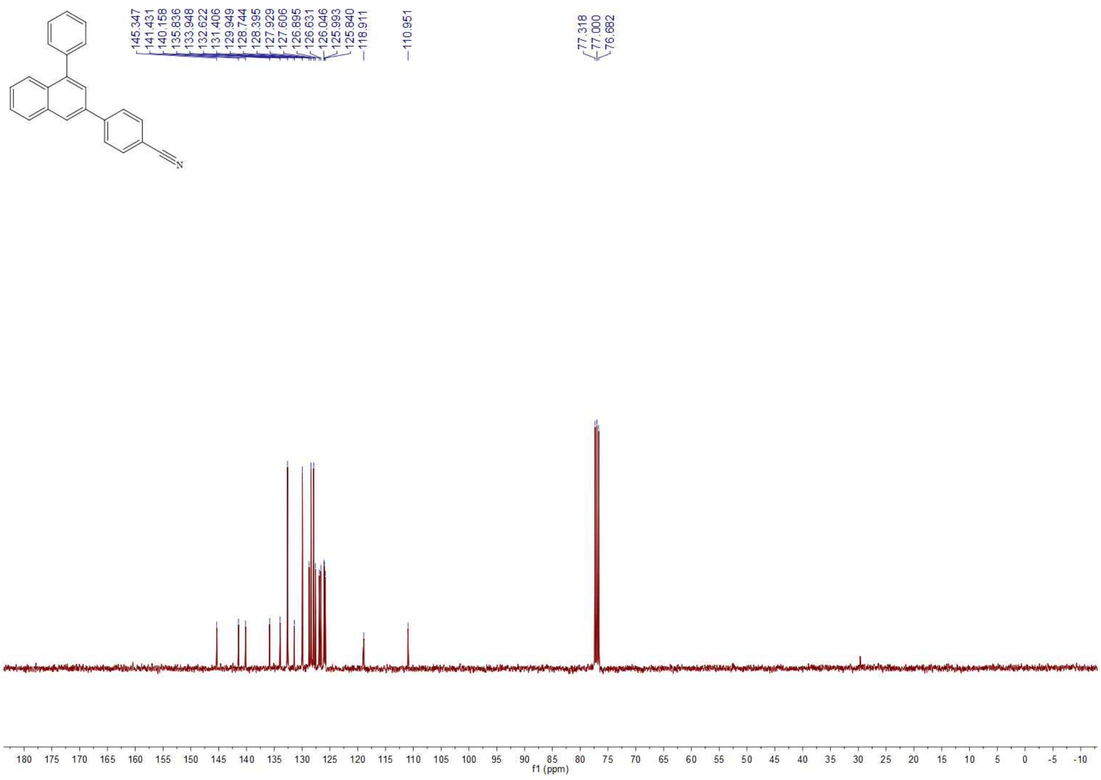

^{1}H NMR (400 MHz, $CDCl_3$) and ^{13}C NMR (100 MHz, $CDCl_3$) Spectrum of **3v**.

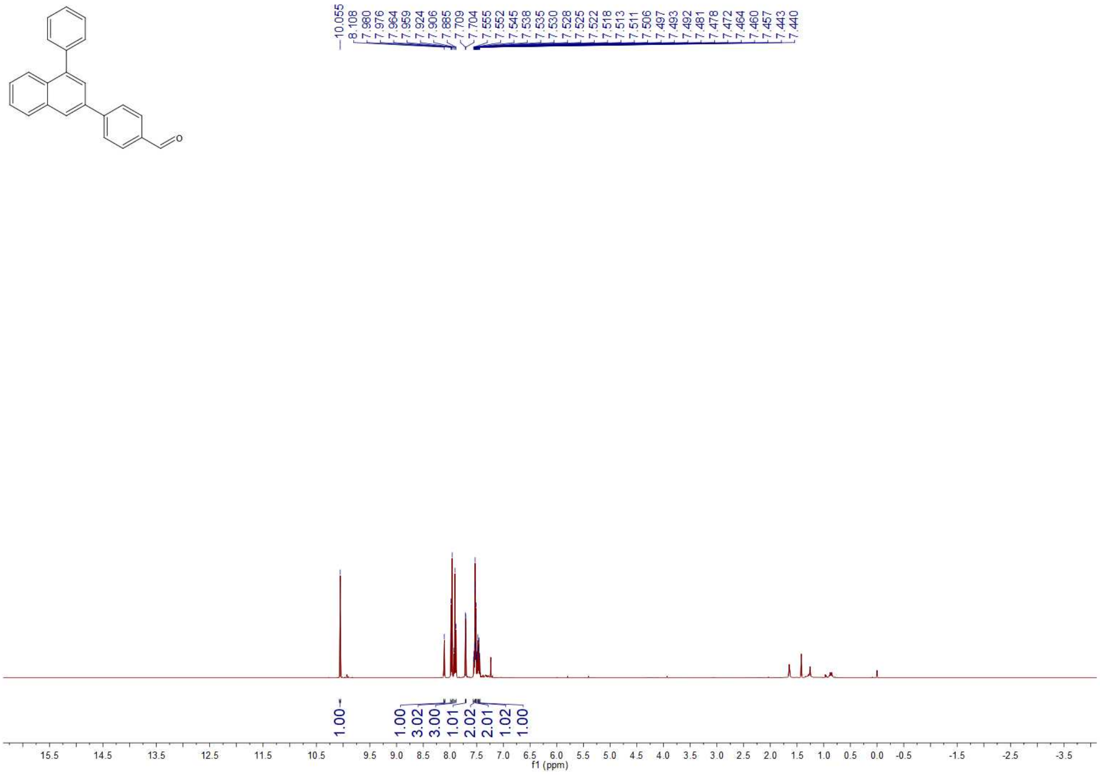

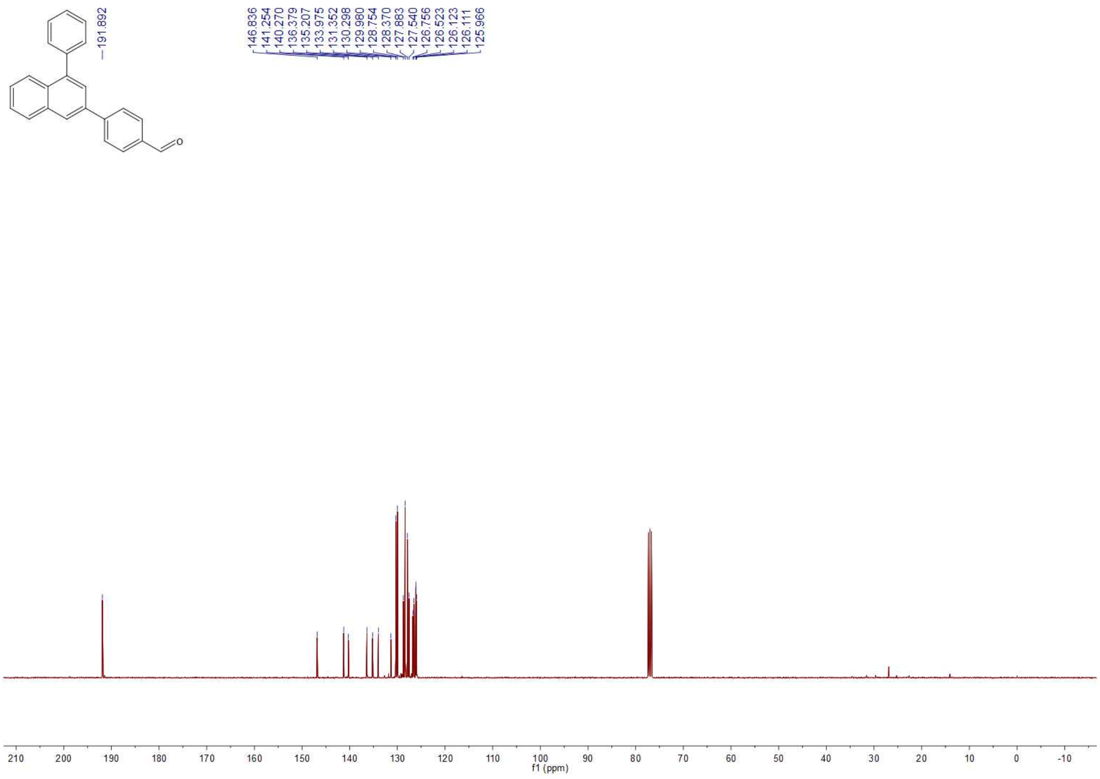

^{1}H NMR (400 MHz, $CDCl_3$) and ^{13}C NMR (100 MHz, $CDCl_3$) Spectrum of **3w**.

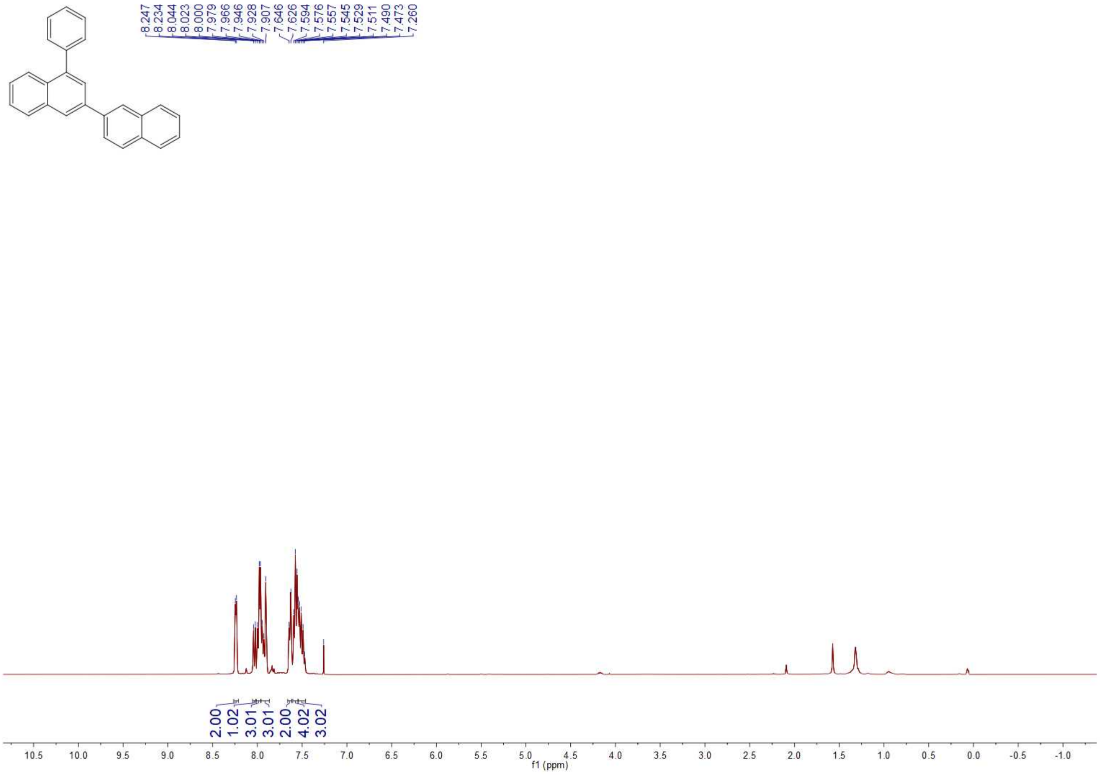

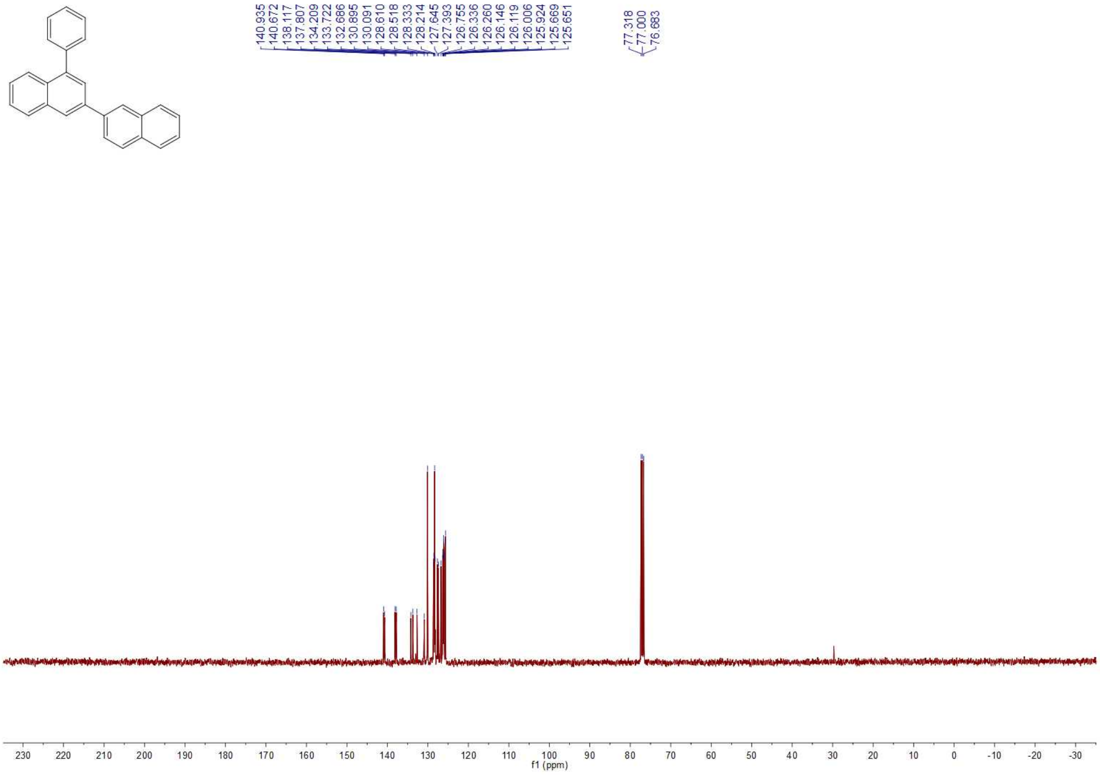

^{1}H NMR (400 MHz, $CDCl_3$) and ^{13}C NMR (100 MHz, $CDCl_3$) Spectrum of **3x**.

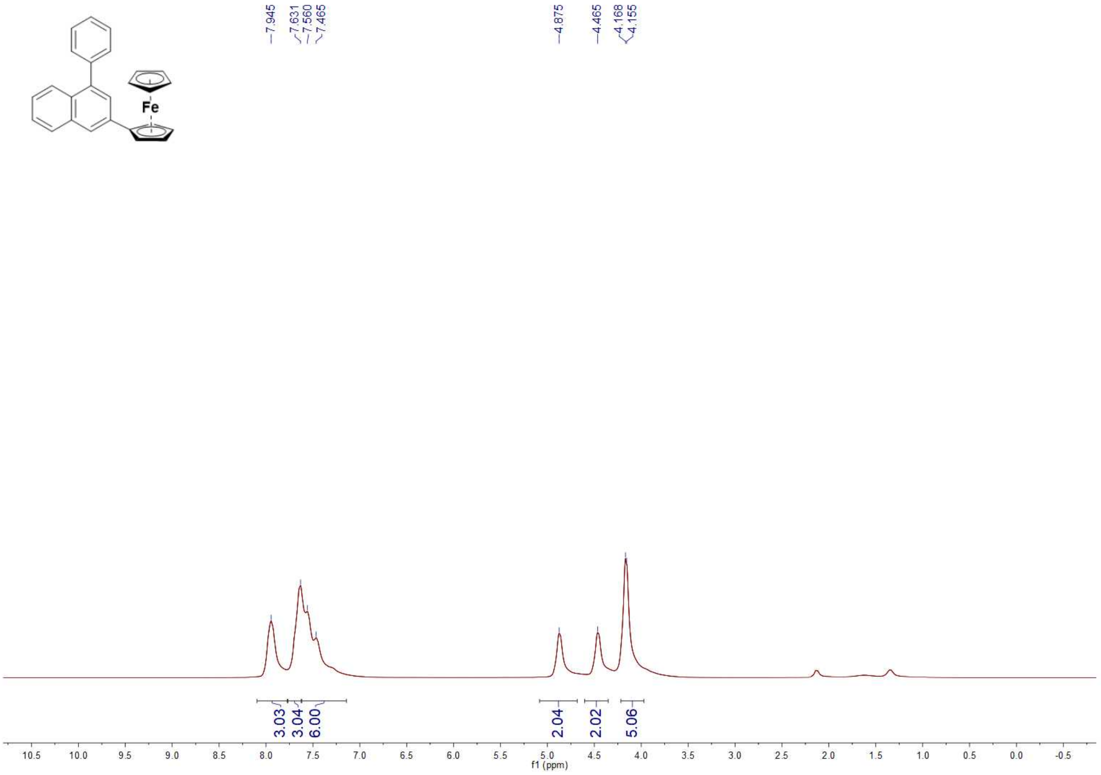

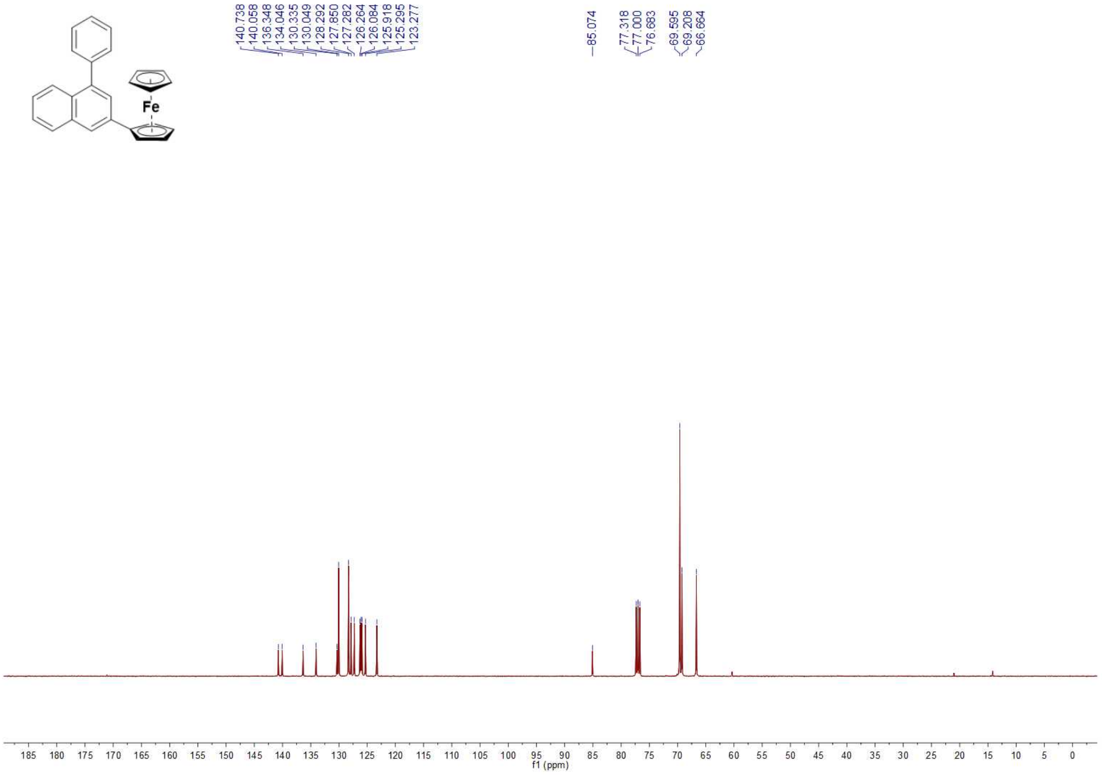

^{1}H NMR (400 MHz, $CDCl_3$) and ^{13}C NMR (100 MHz, $CDCl_3$) Spectrum of **3y**.

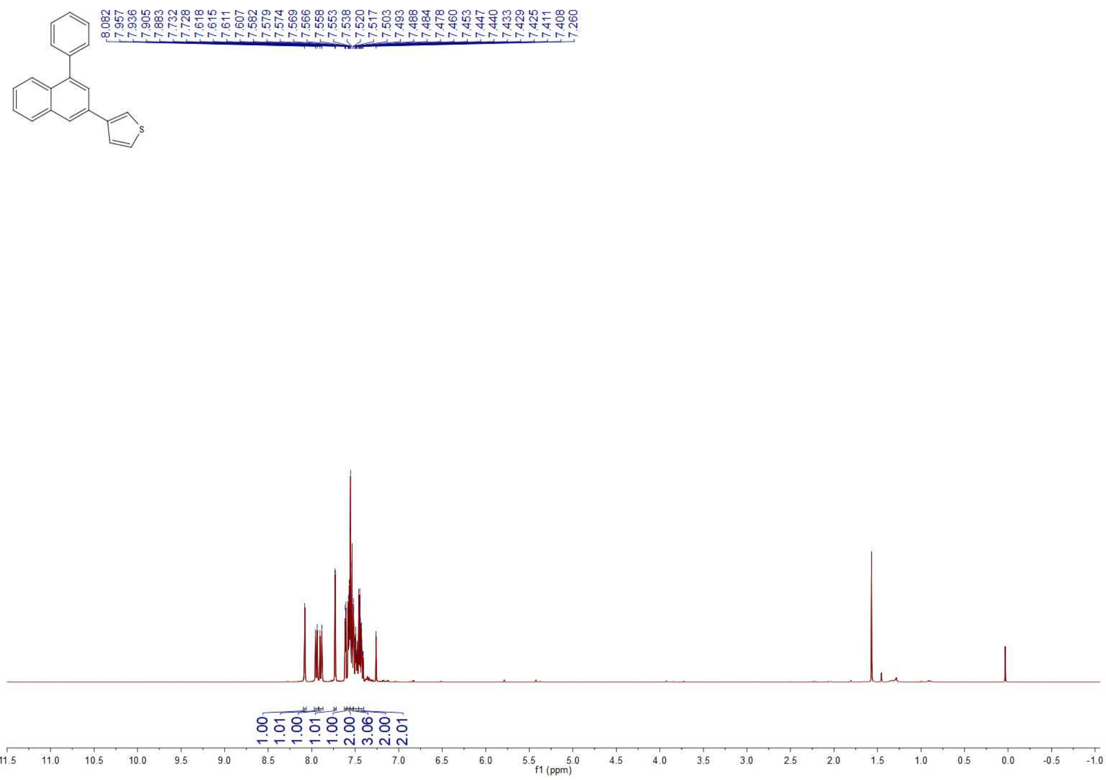

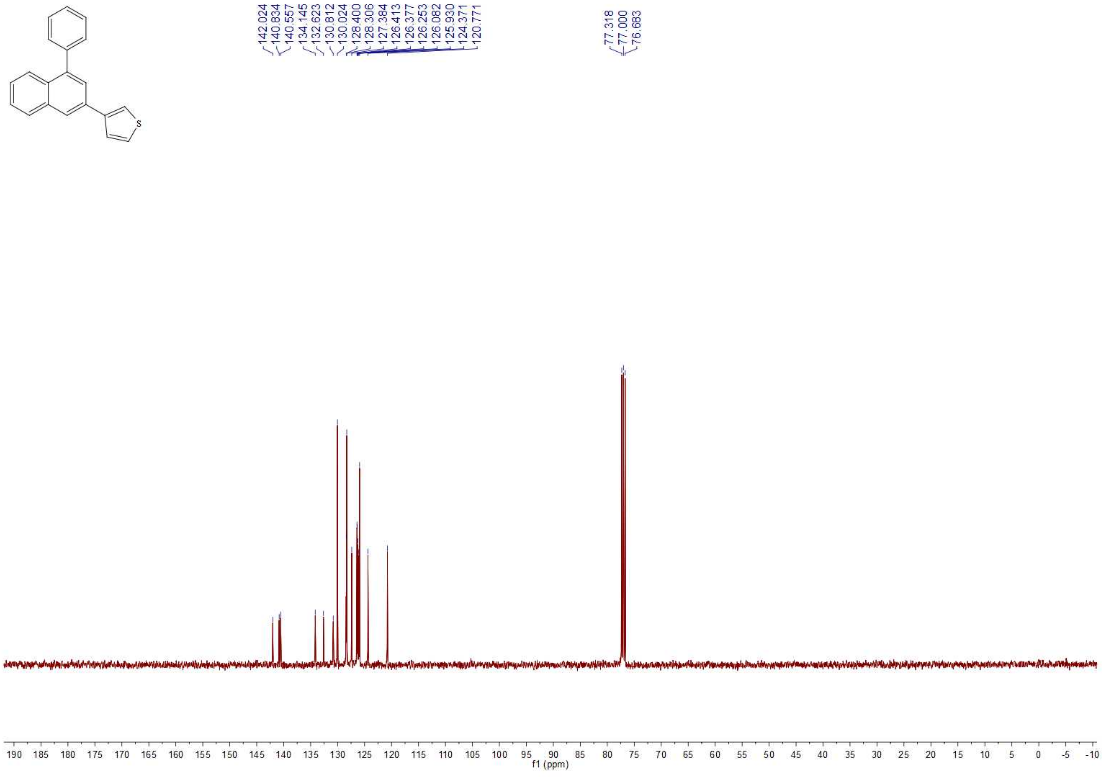

^1H NMR (400 MHz, $CDCl_3$) and ^{13}C NMR (100 MHz, $CDCl_3$) Spectrum of **3z**.

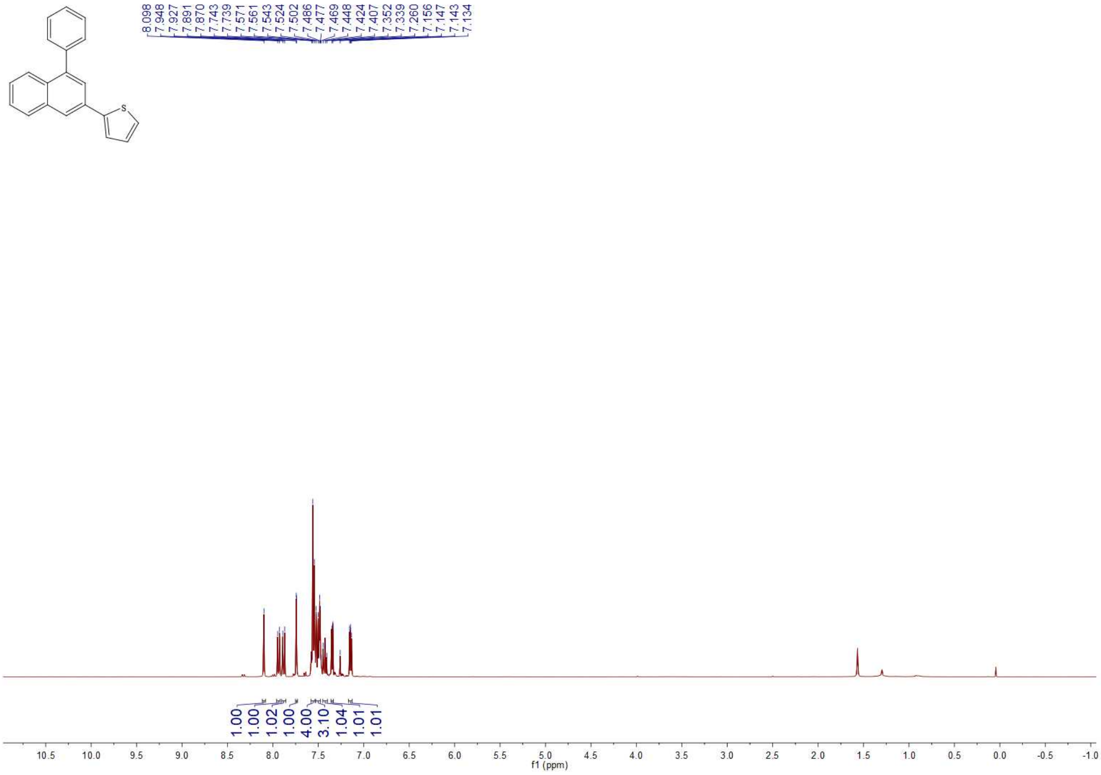

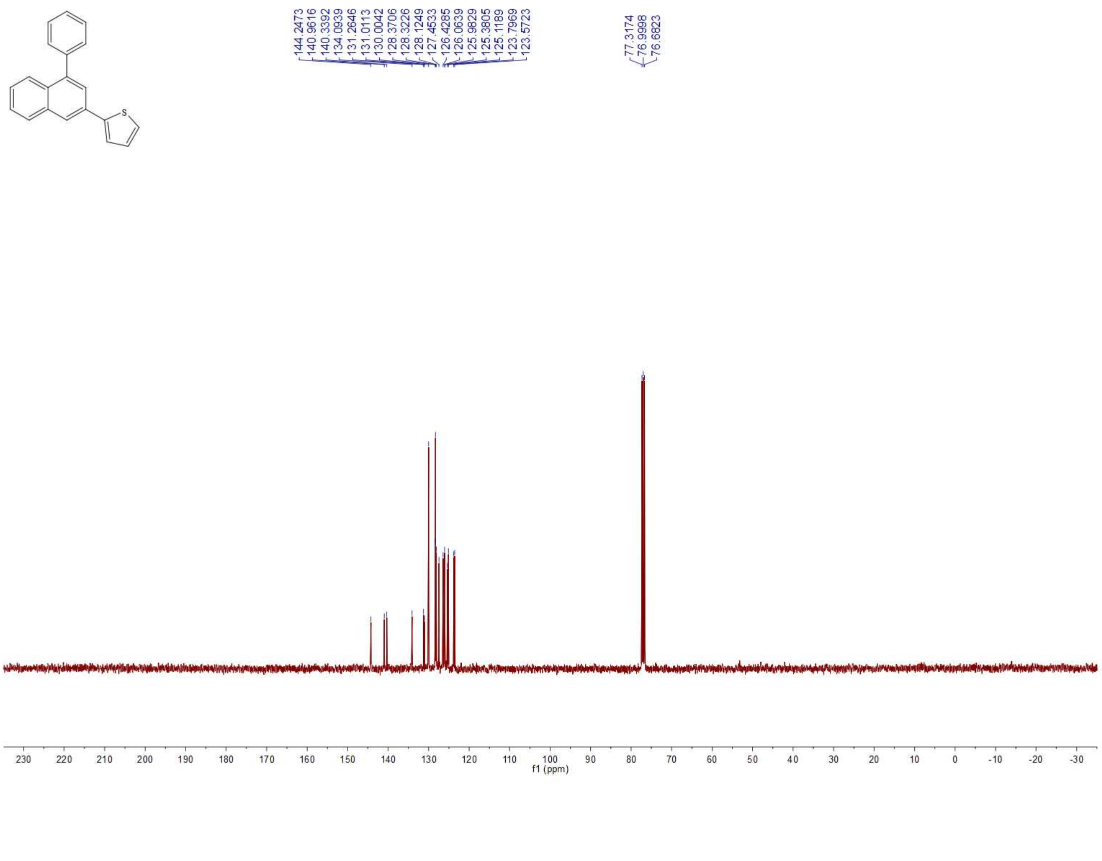

^{1}H NMR (400 MHz, $CDCl_3$) and ^{13}C NMR (100 MHz, $CDCl_3$) Spectrum of **3aa**.

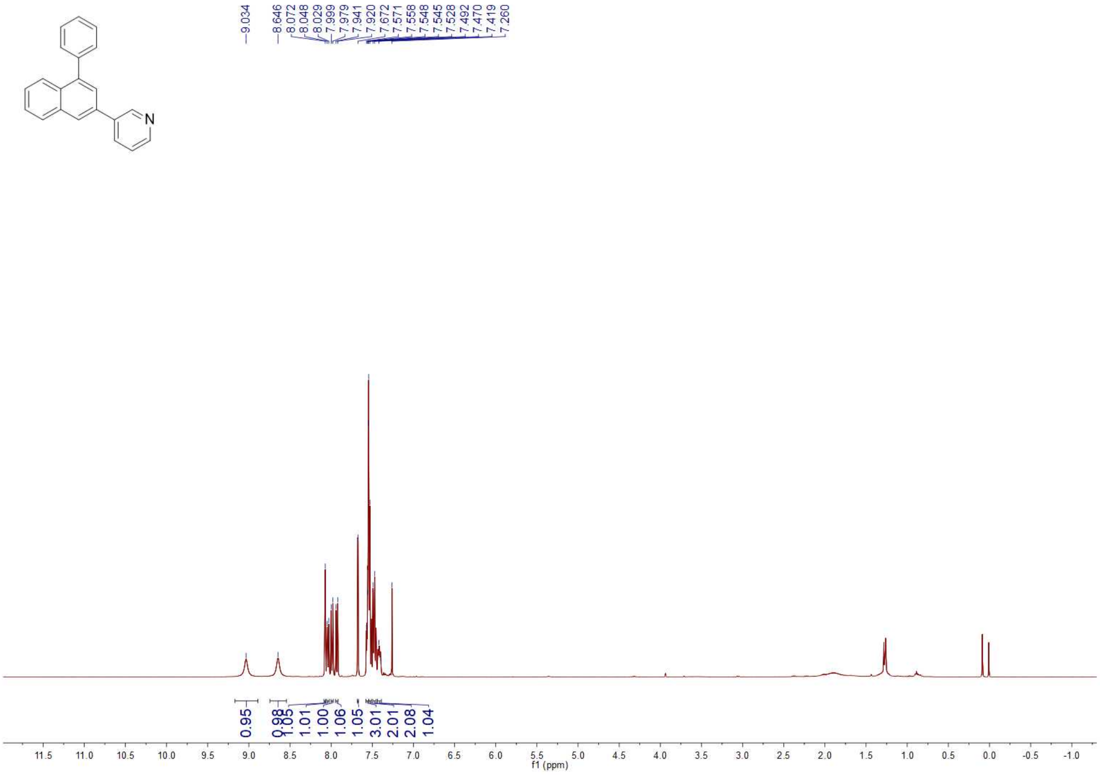

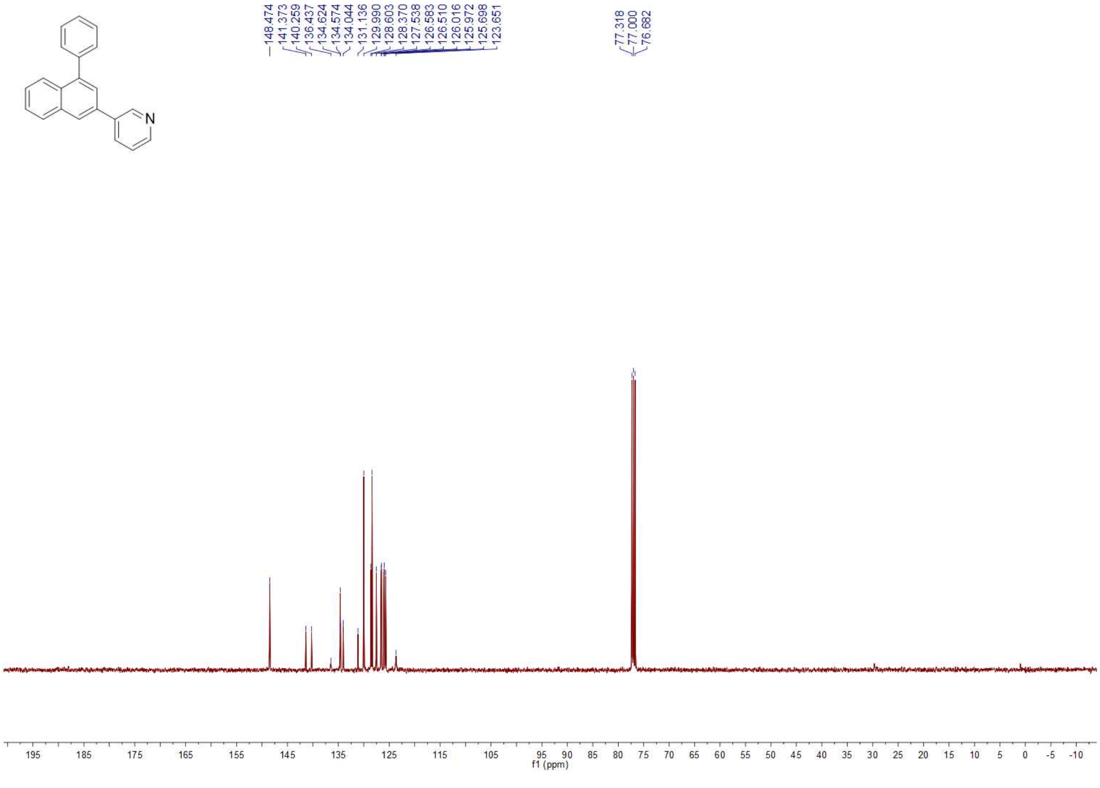

^{1}H NMR (400 MHz, $CDCl_3$) and ^{13}C NMR (100 MHz, $CDCl_3$) Spectrum of **3ab**.

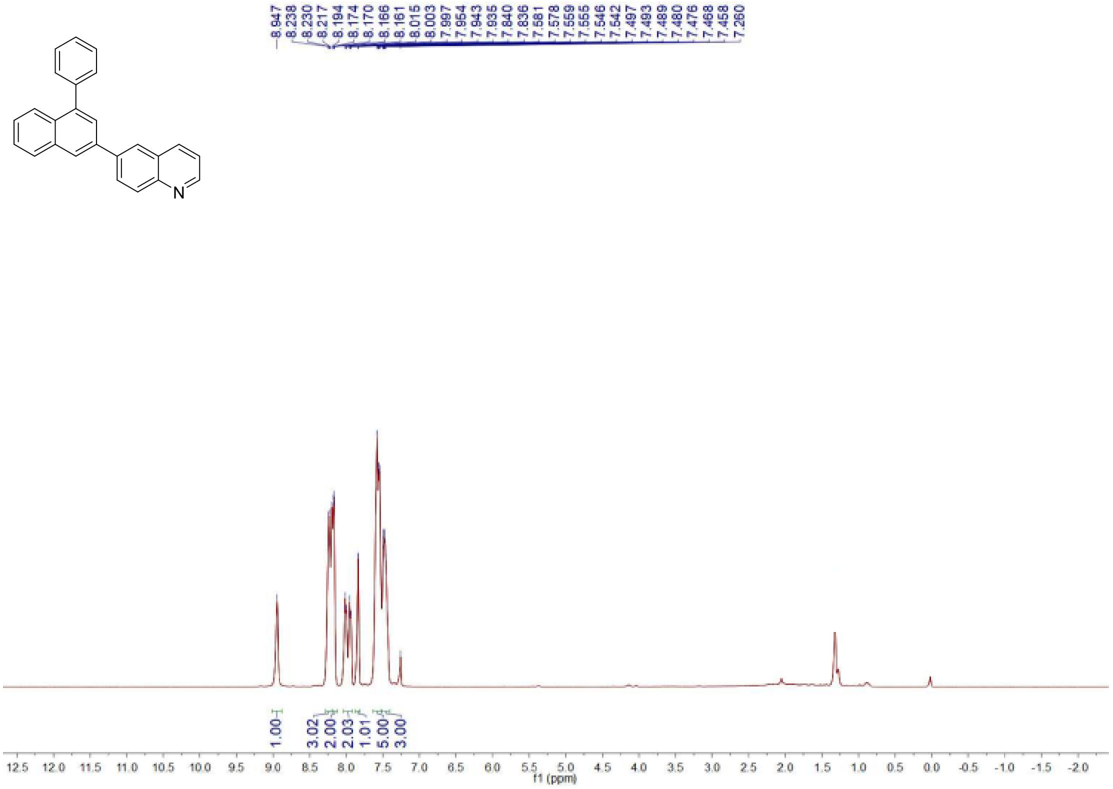

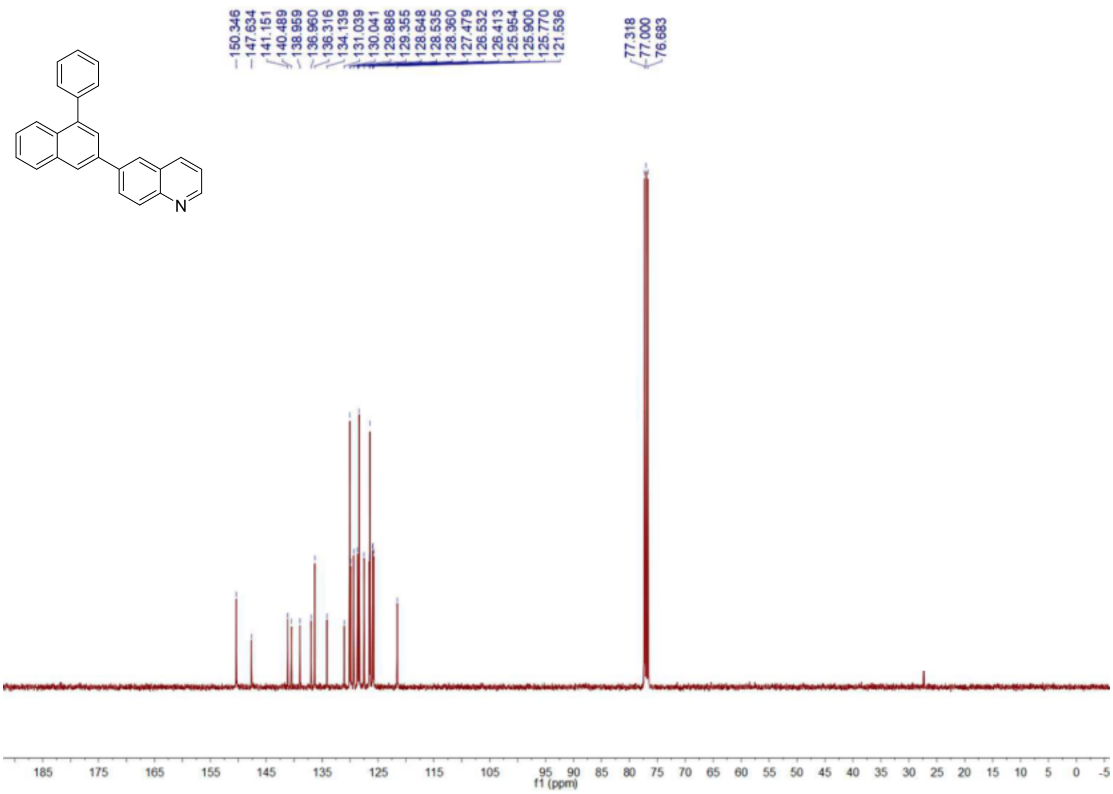

^{1}H NMR (400 MHz, $CDCl_3$) and ^{13}C NMR (100 MHz, $CDCl_3$) Spectrum of **3ac**.

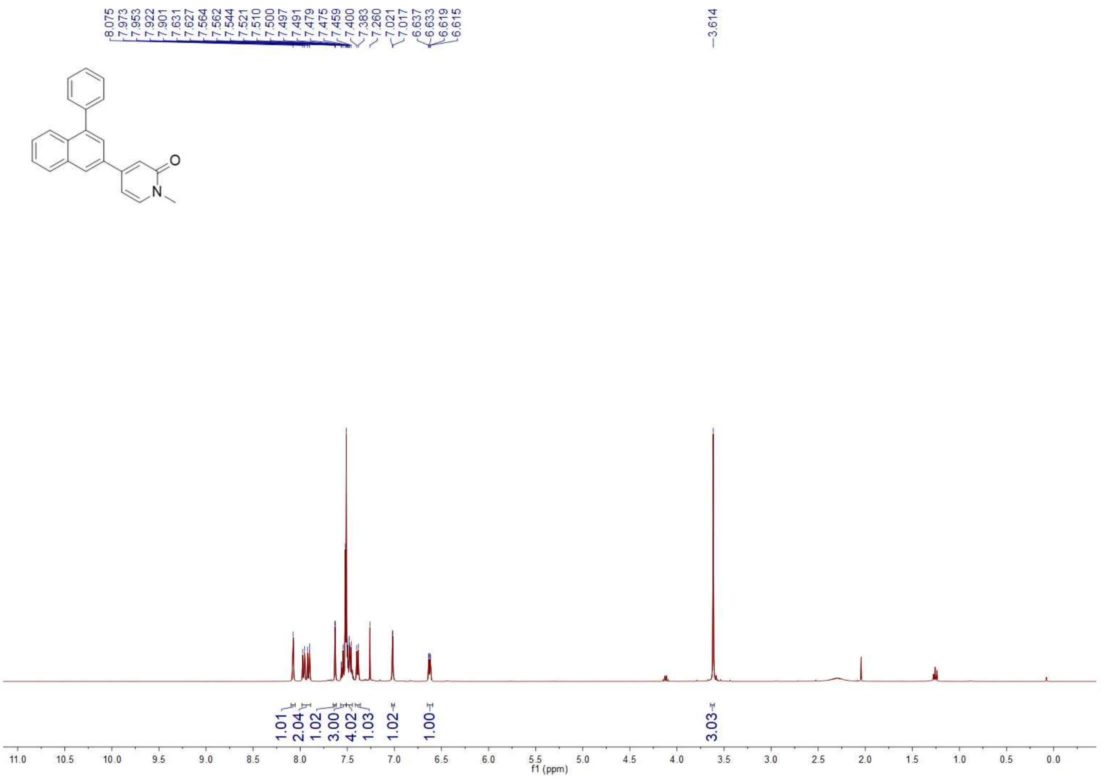

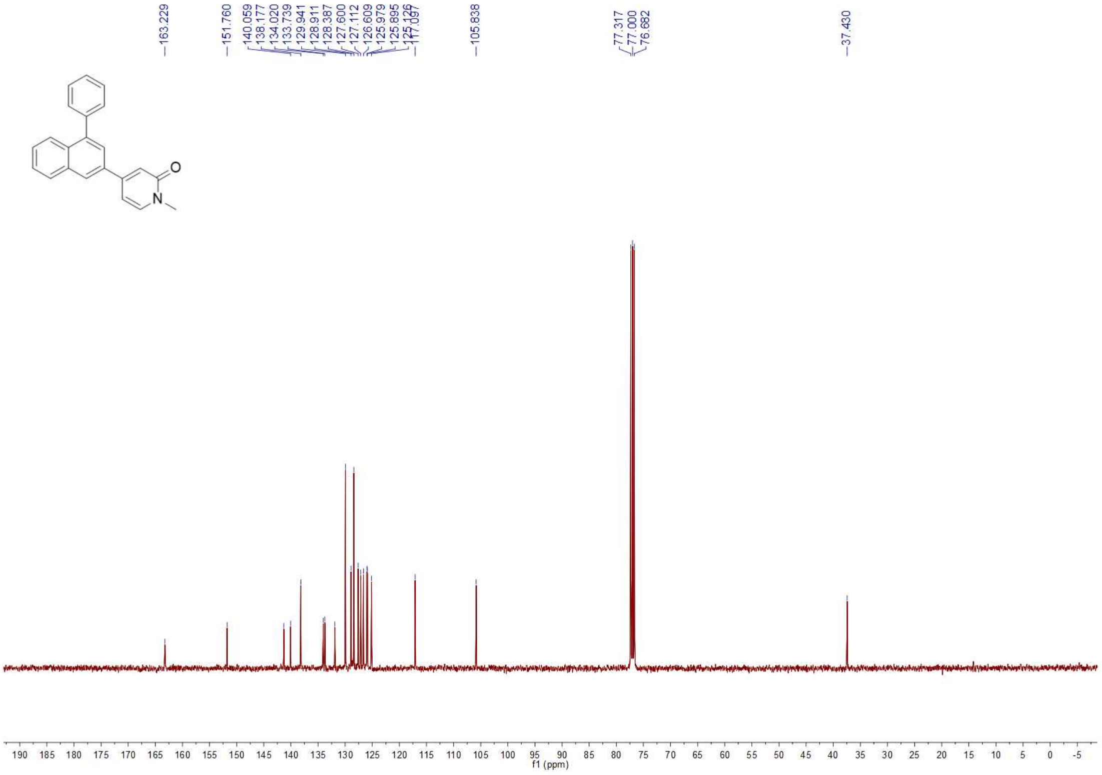

^{1}H NMR (400 MHz, $CDCl_3$) and ^{13}C NMR (100 MHz, $CDCl_3$) Spectrum of **3ad**.

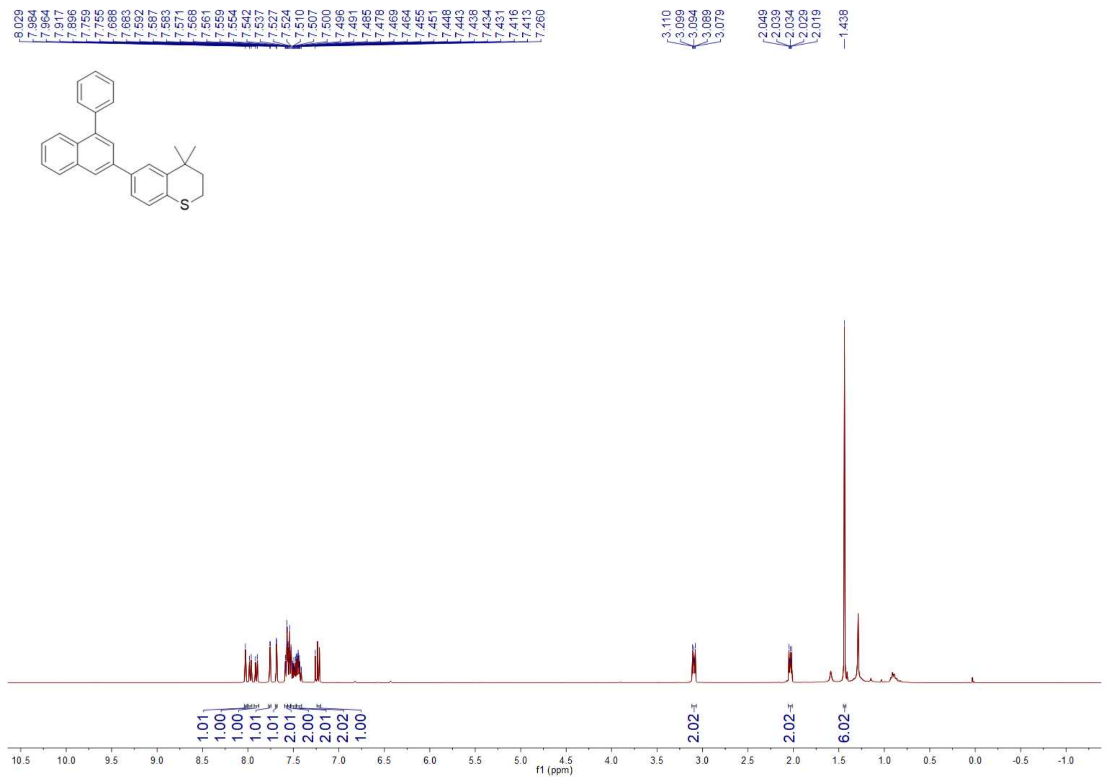

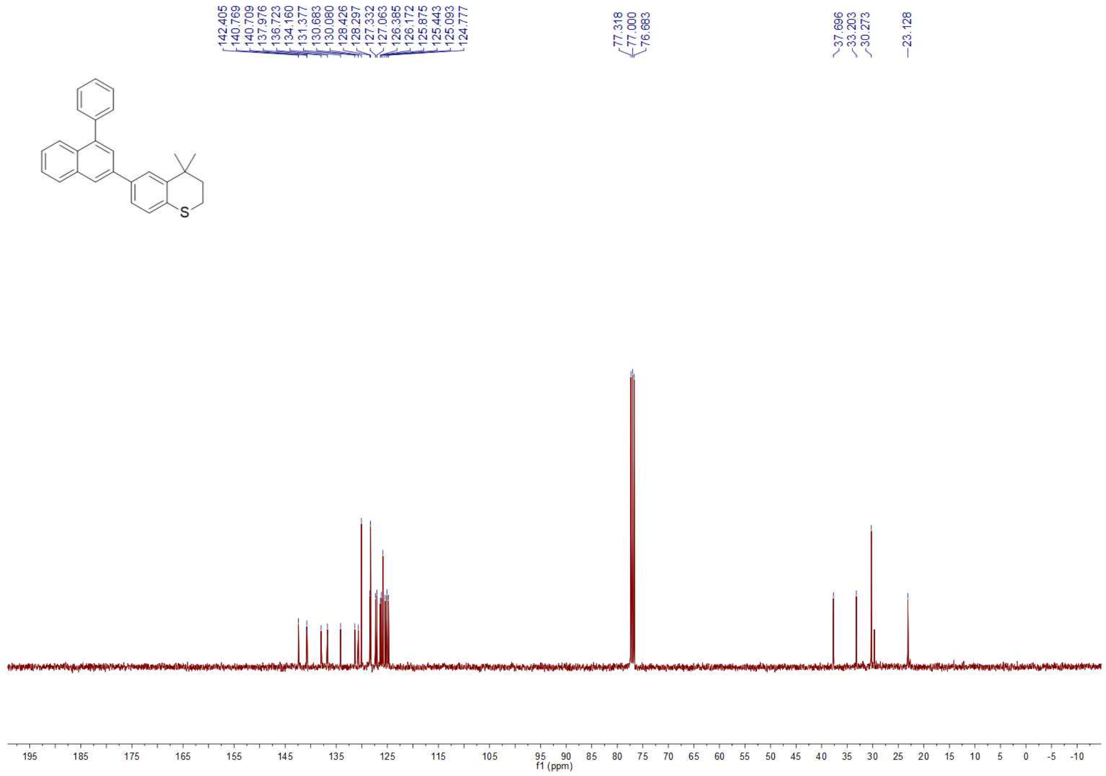

^{1}H NMR (400 MHz, $CDCl_3$) and ^{13}C NMR (100 MHz, $CDCl_3$) Spectrum of **3ae**.

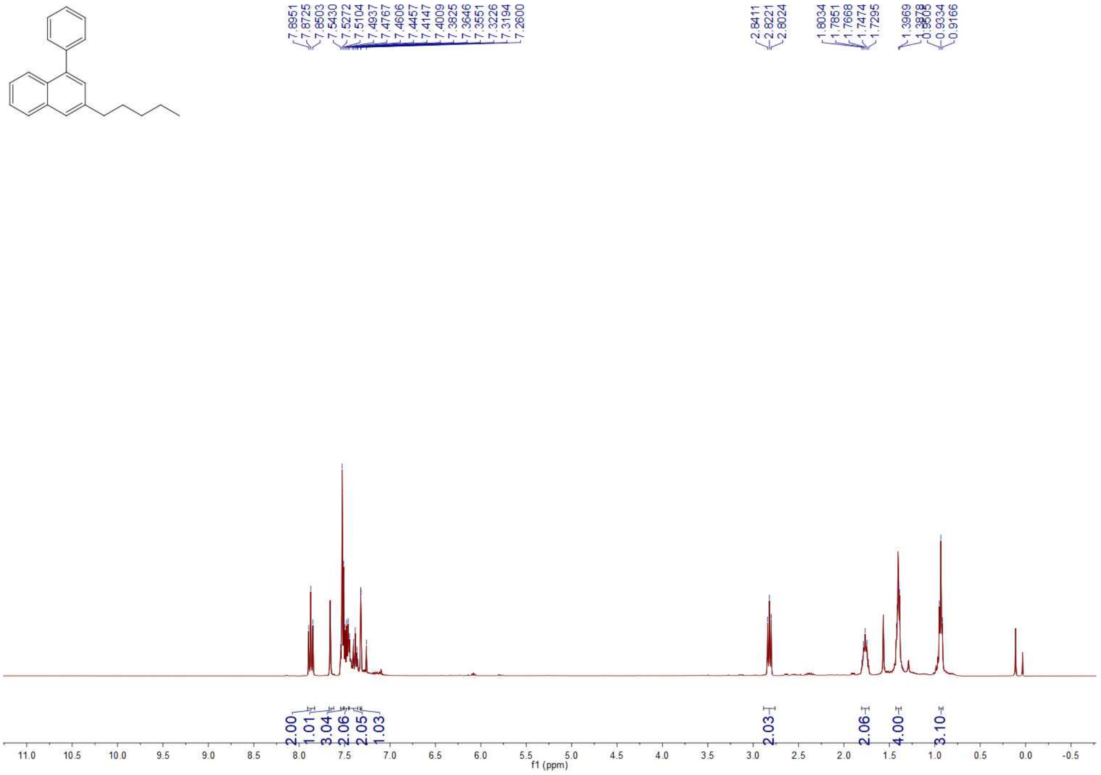

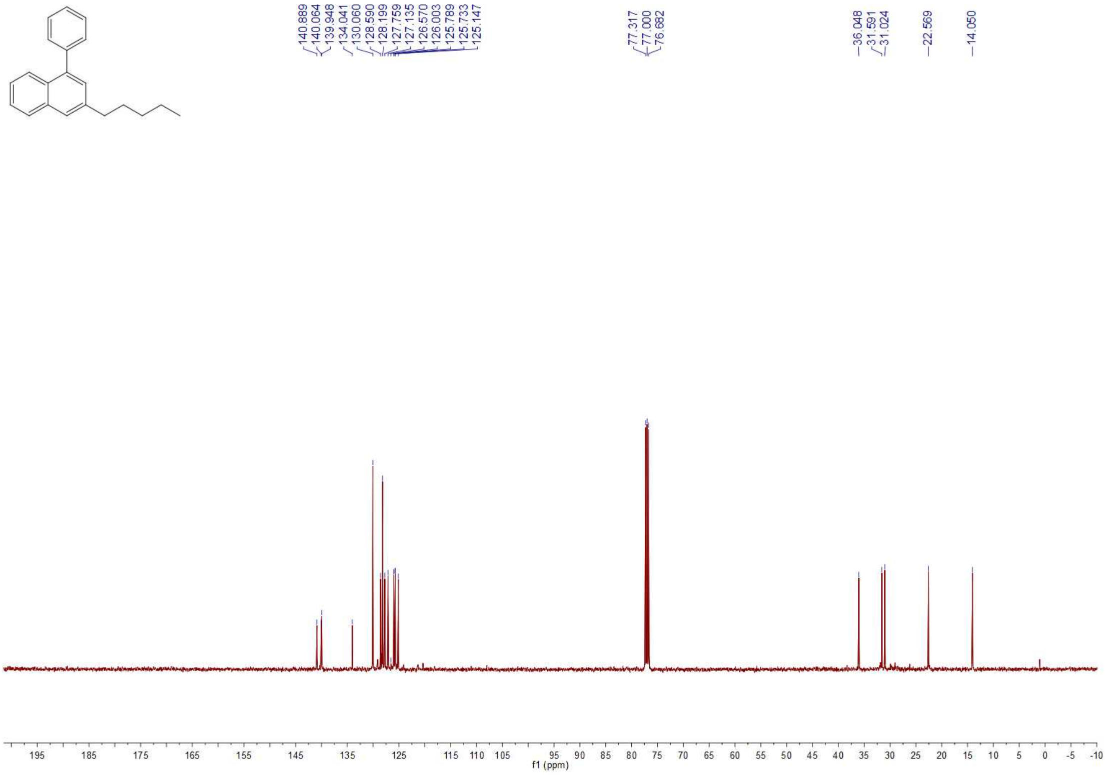

^{1}H NMR (400 MHz, $CDCl_3$) and ^{13}C NMR (100 MHz, $CDCl_3$) Spectrum of **3af**.

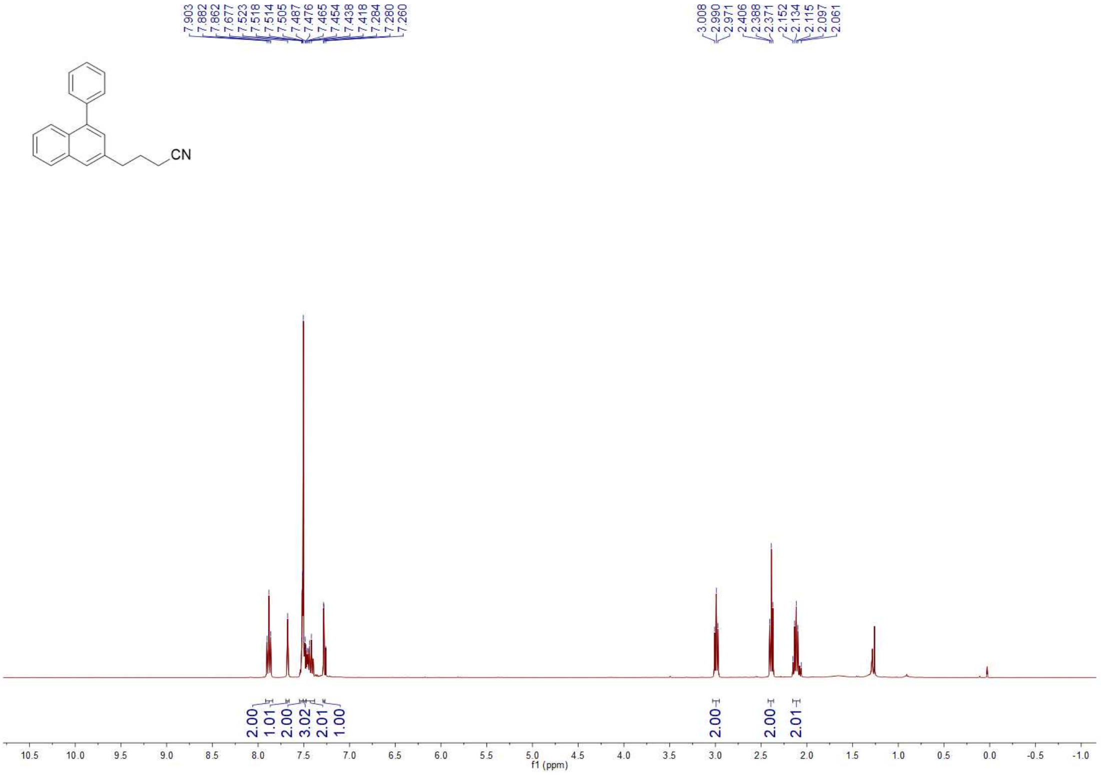

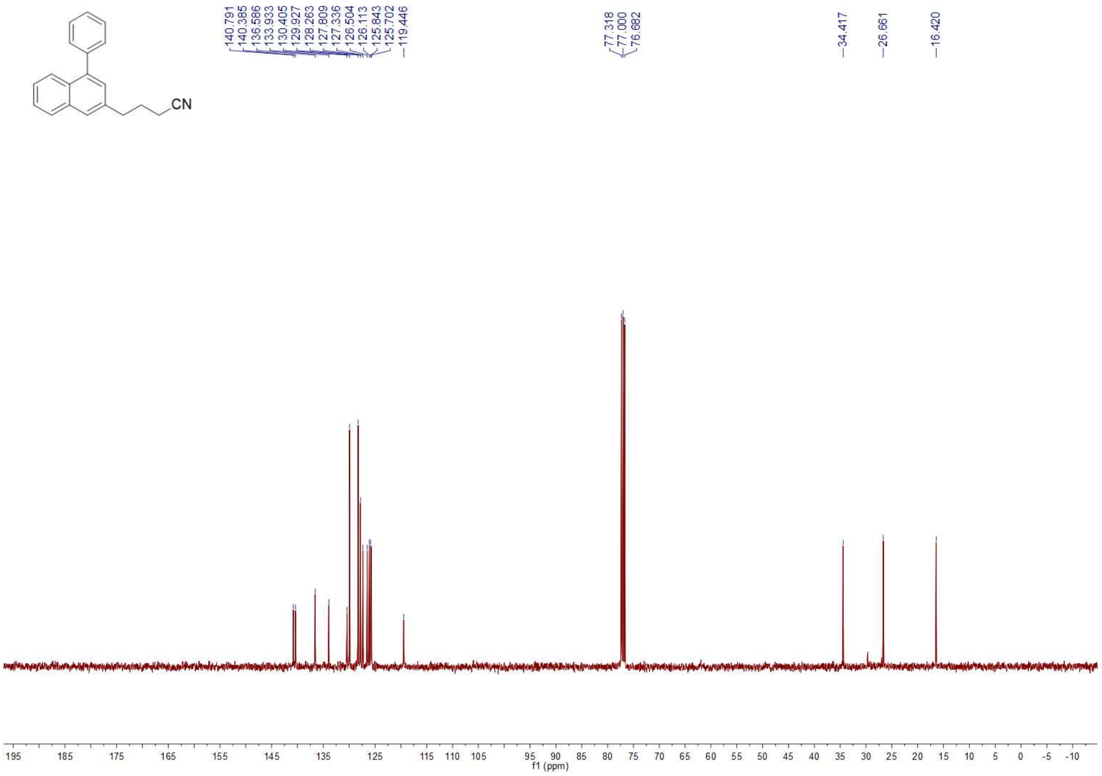

^{1}H NMR (400 MHz, $CDCl_3$) and ^{13}C NMR (100 MHz, $CDCl_3$) Spectrum of **3ag**.

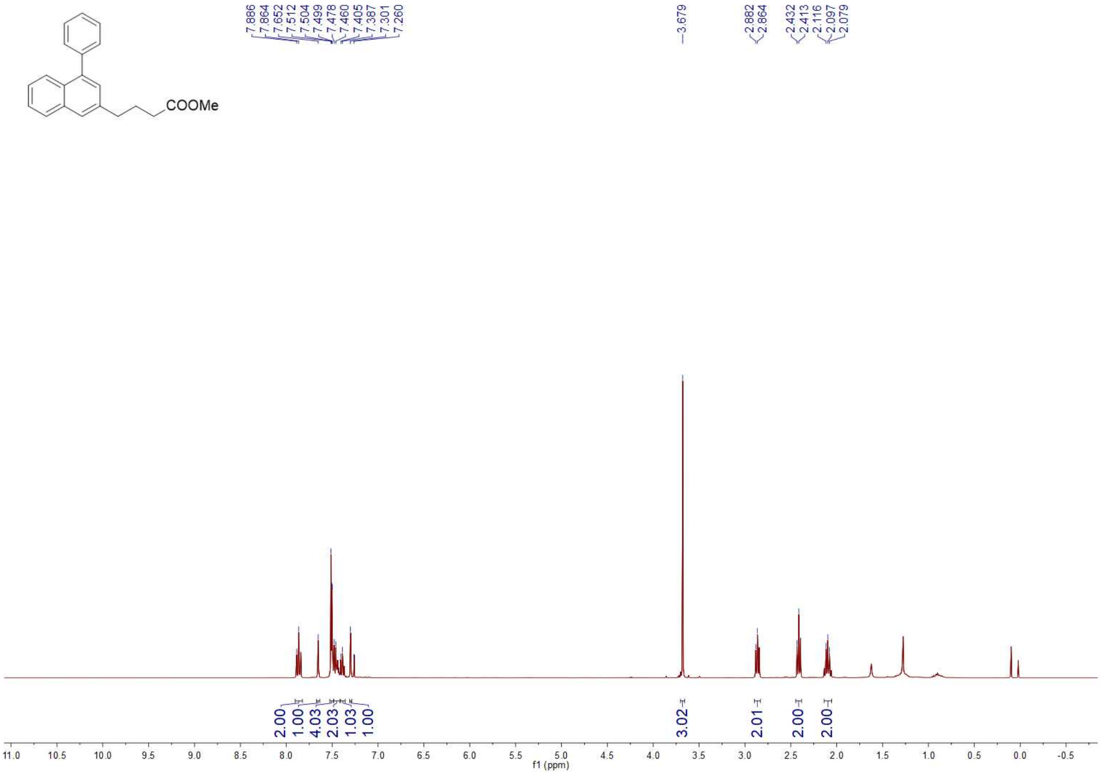

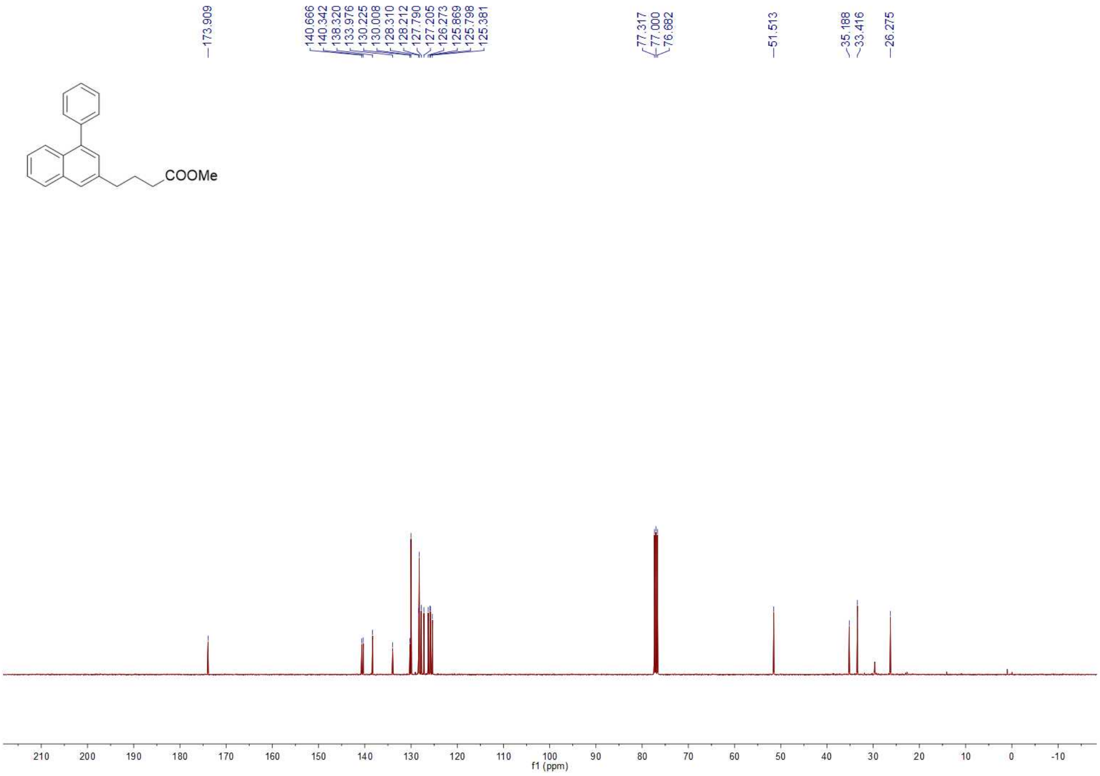

^1H NMR (400 MHz, $CDCl_3$) and ^{13}C NMR (100 MHz, $CDCl_3$) Spectrum of **3ah**.

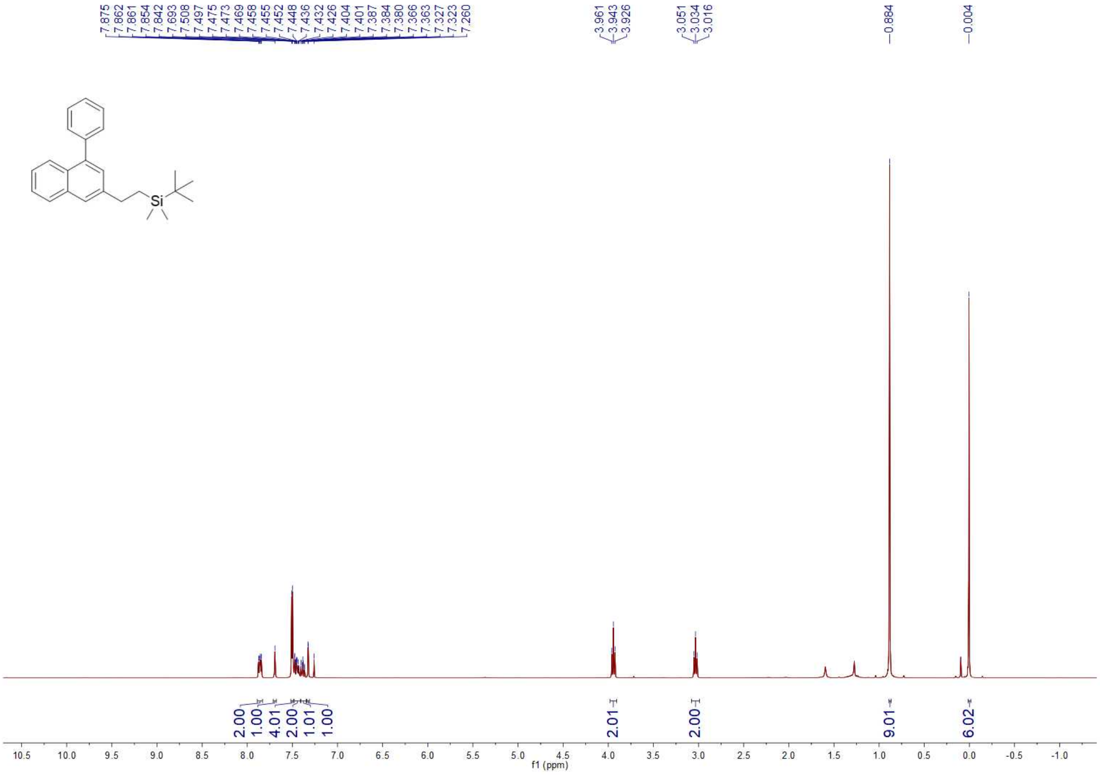

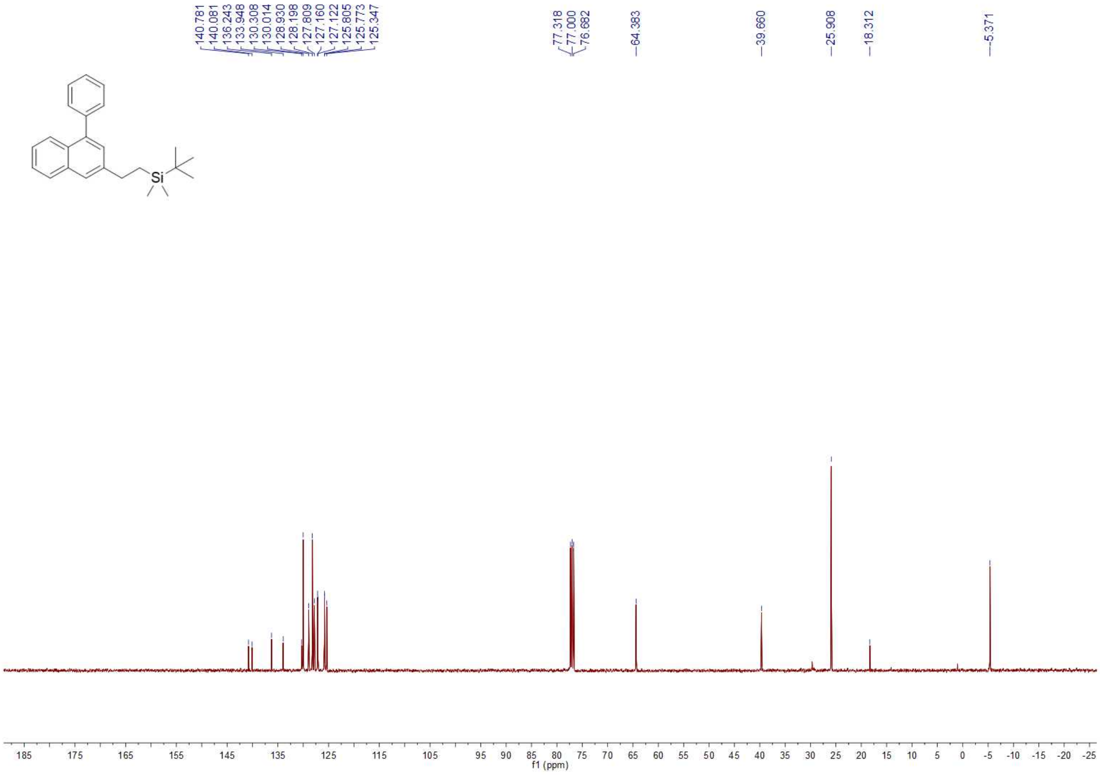

^{1}H NMR (400 MHz, $CDCl_3$) and ^{13}C NMR (100 MHz, $CDCl_3$) Spectrum of **3ai**.

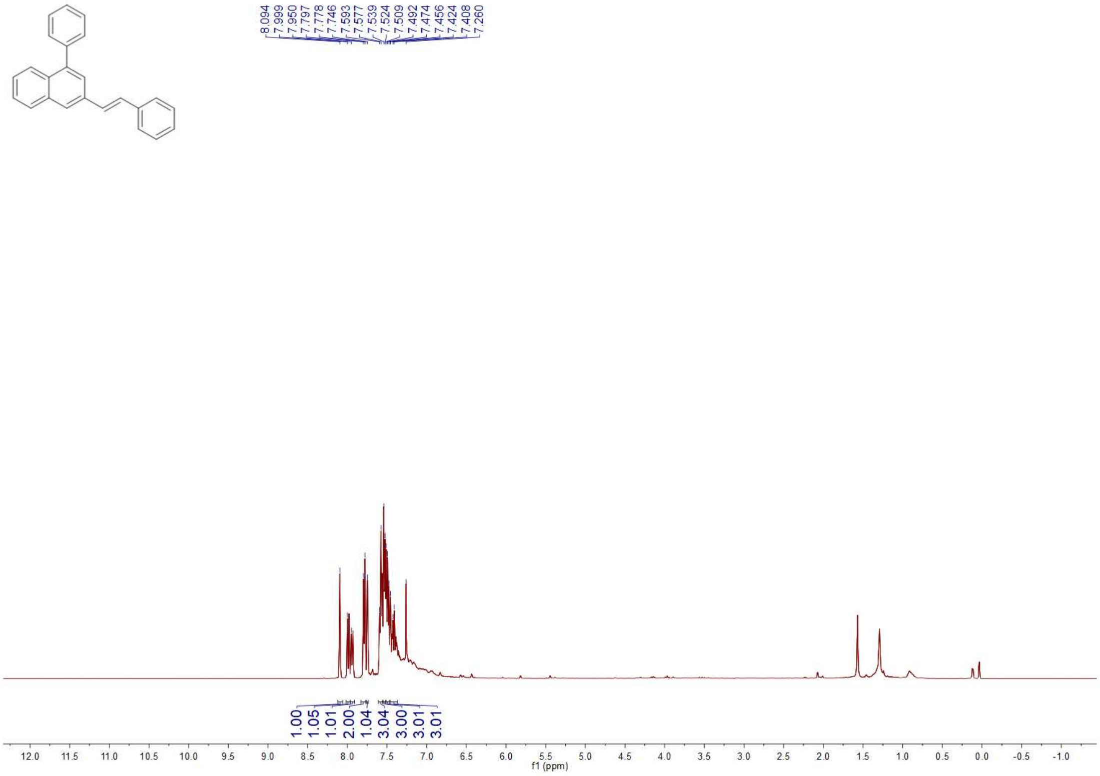

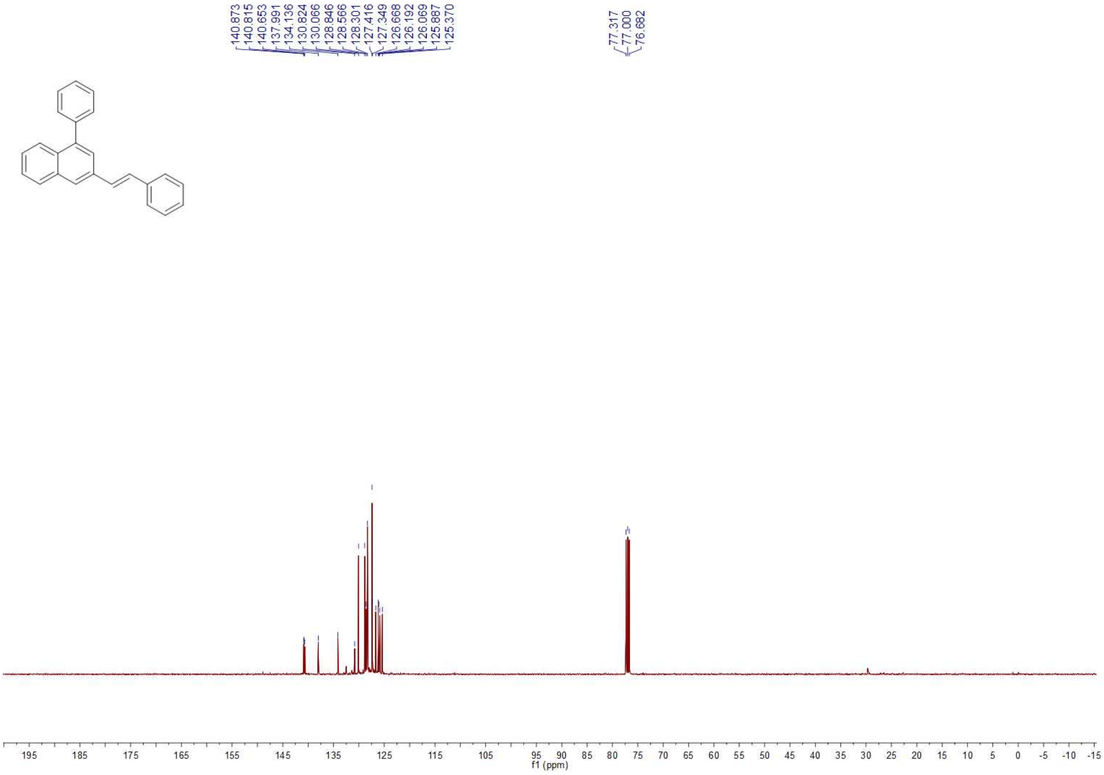

^{1}H NMR (400 MHz, $CDCl_3$) and ^{13}C NMR (100 MHz, $CDCl_3$) Spectrum of **3aj**.

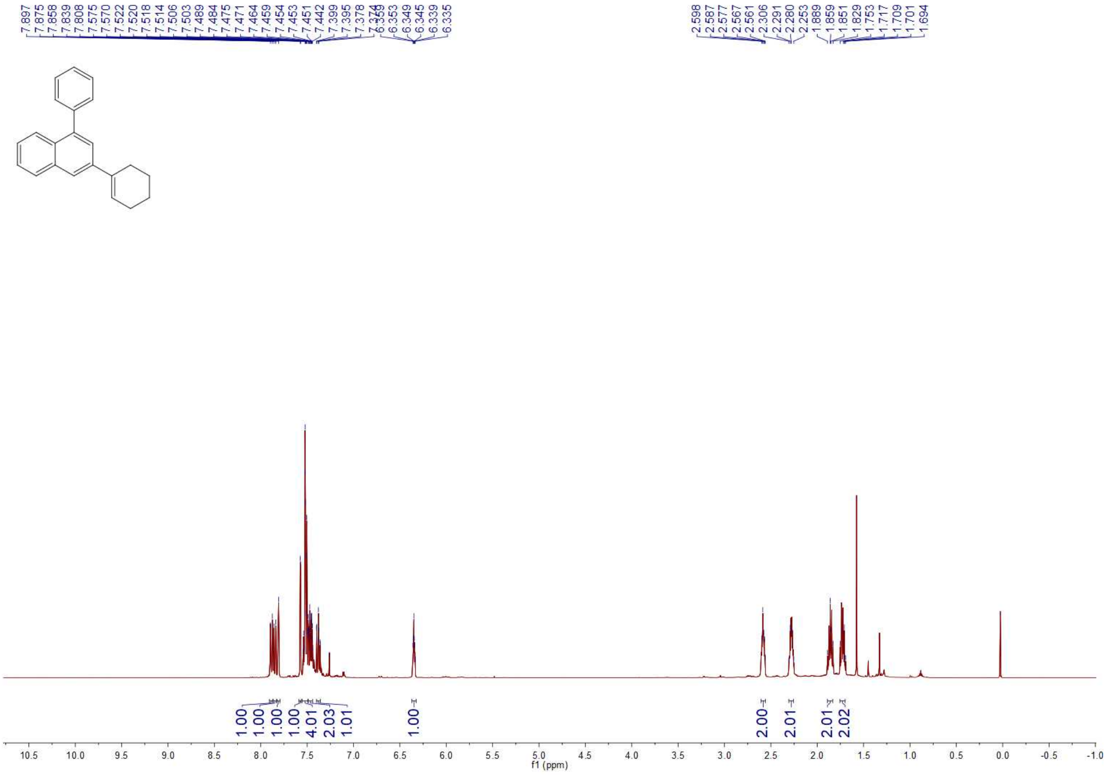

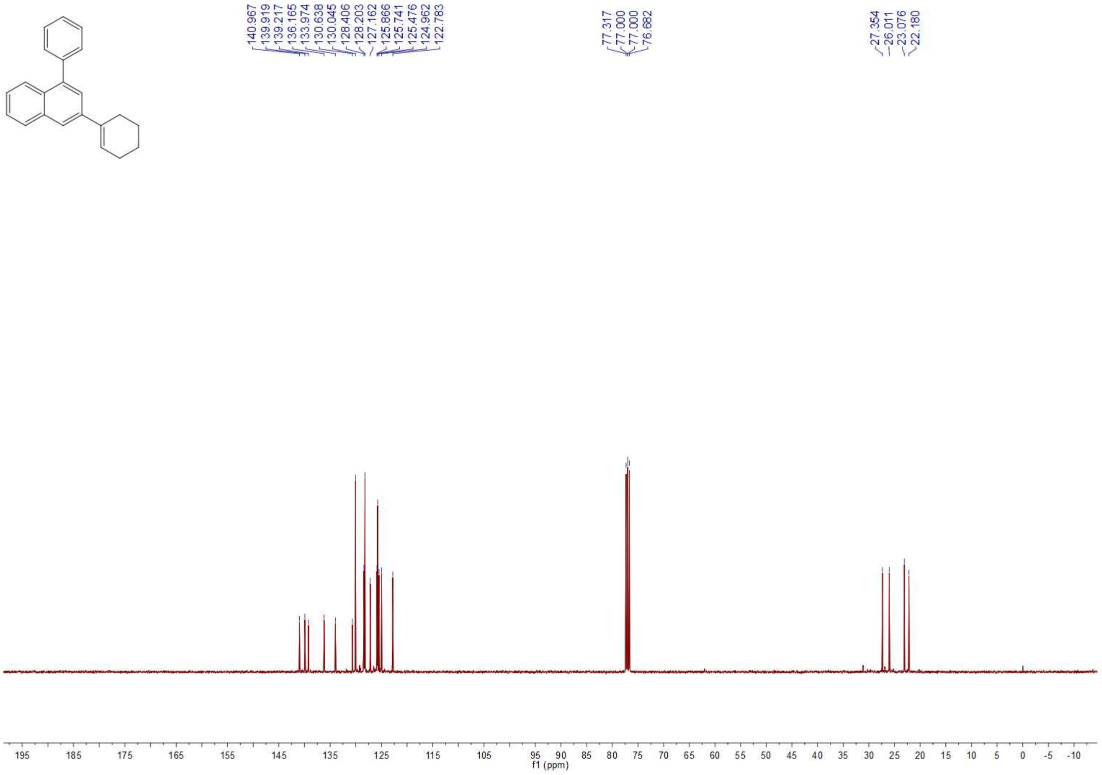

^{1}H NMR (400 MHz, $CDCl_3$) and ^{13}C NMR (100 MHz, $CDCl_3$) Spectrum of **3ak**.

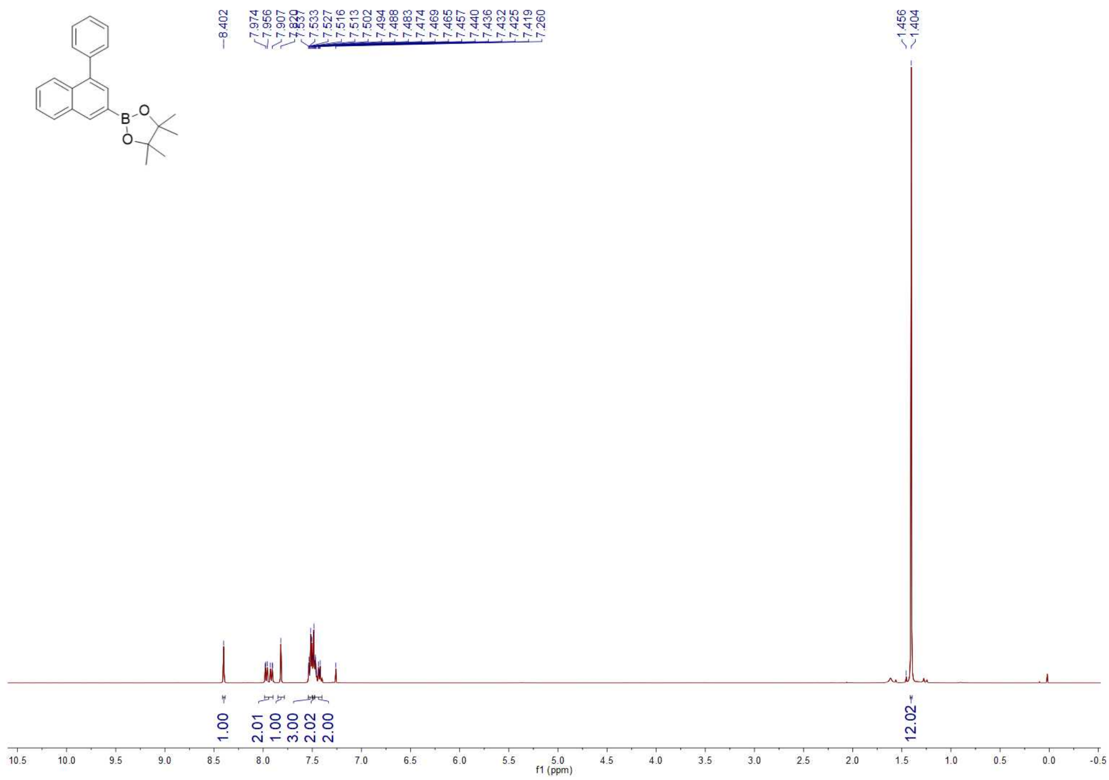

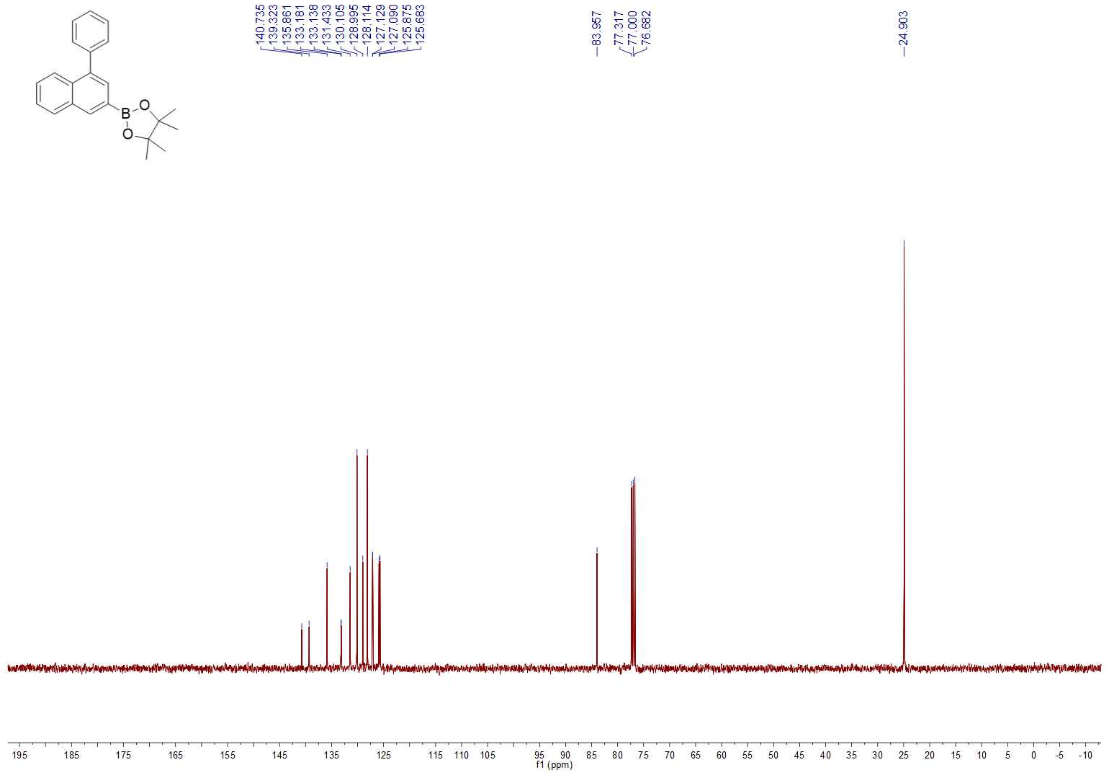

^{1}H NMR (400 MHz, $CDCl_3$) and ^{13}C NMR (100 MHz, $CDCl_3$) Spectrum of **3al**.

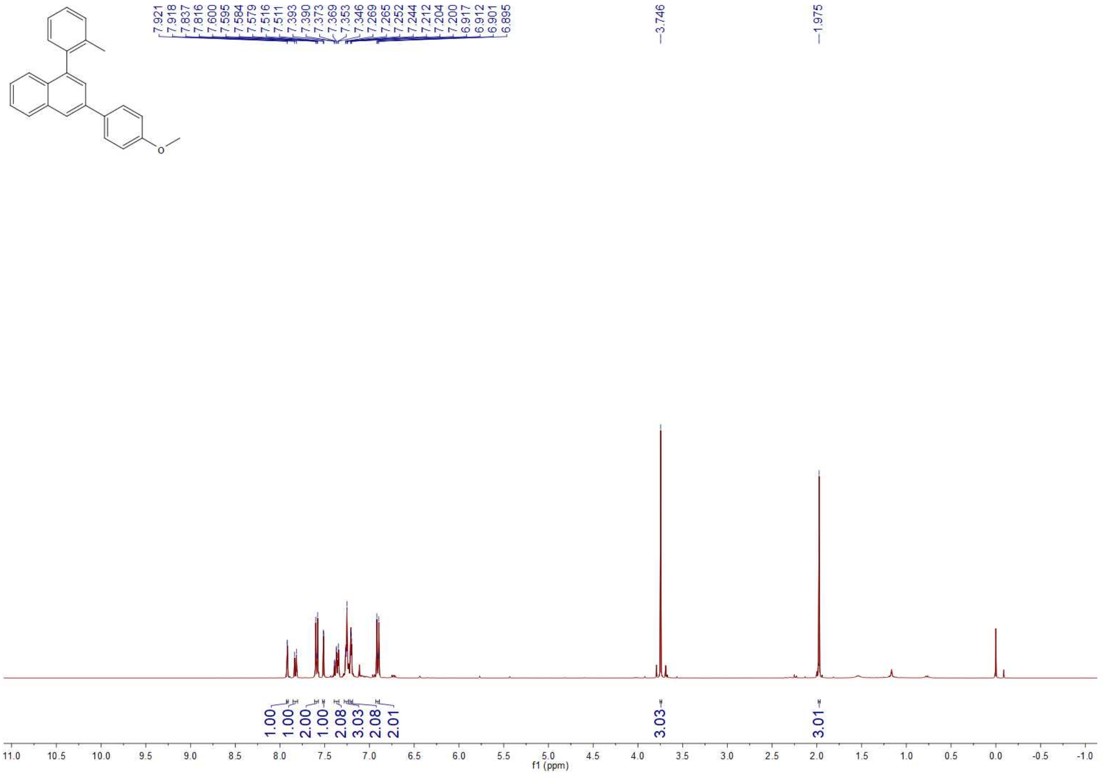

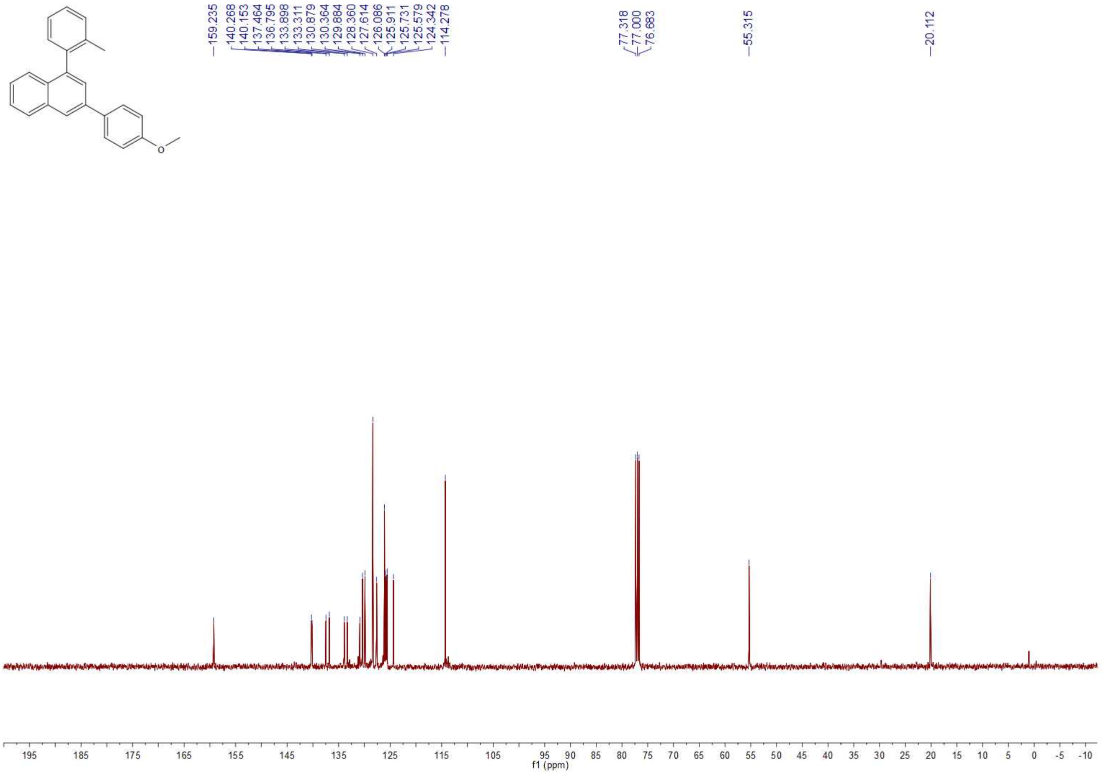

^{1}H NMR (400 MHz, $CDCl_3$) and ^{13}C NMR (100 MHz, $CDCl_3$) Spectrum of **3am**.

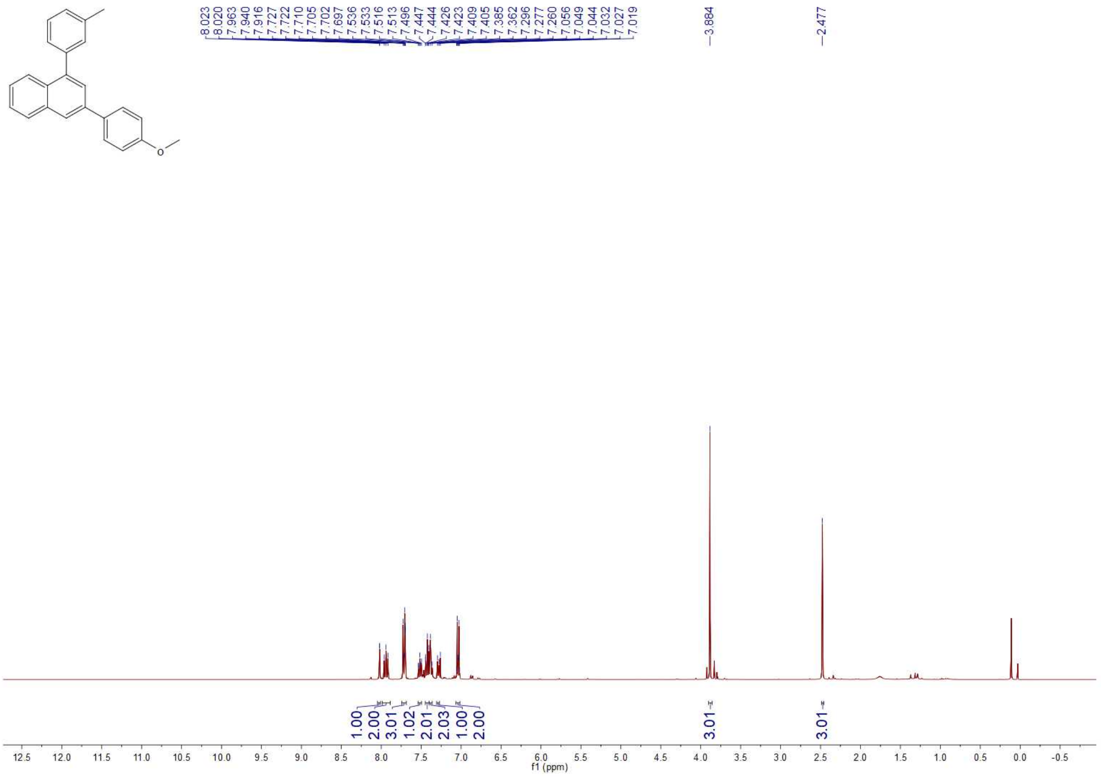

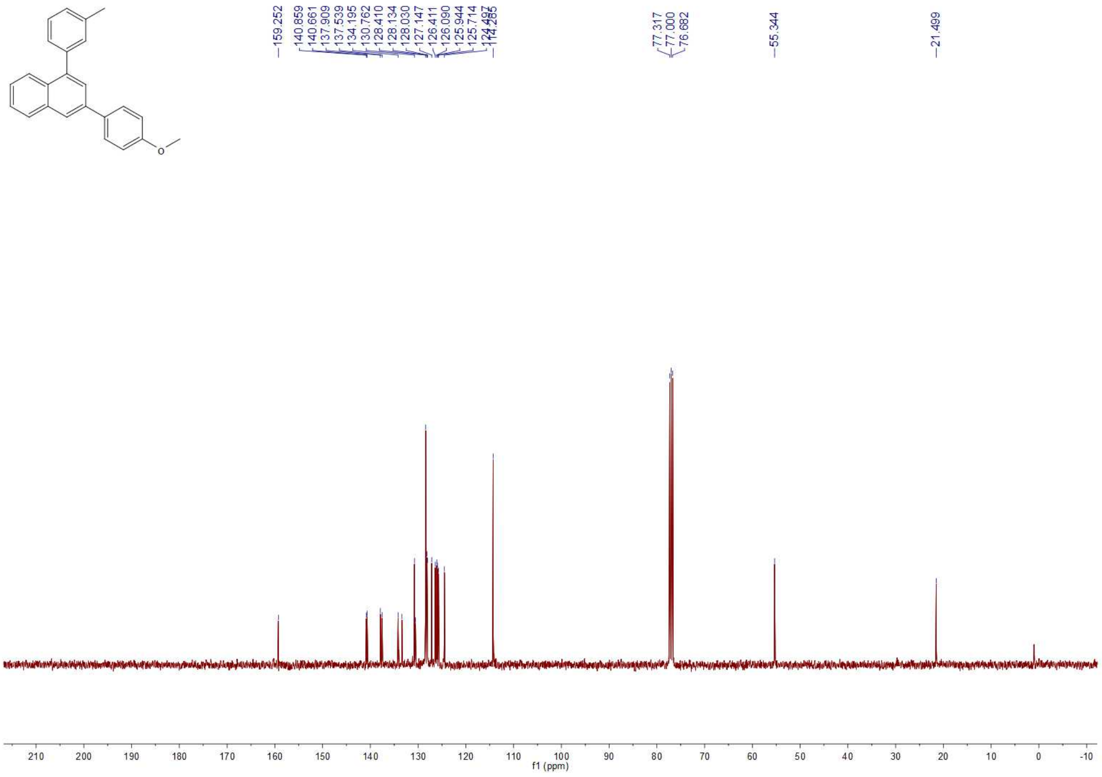

^{1}H NMR (400 MHz, $CDCl_3$) and ^{13}C NMR (100 MHz, $CDCl_3$) Spectrum of **3an**.

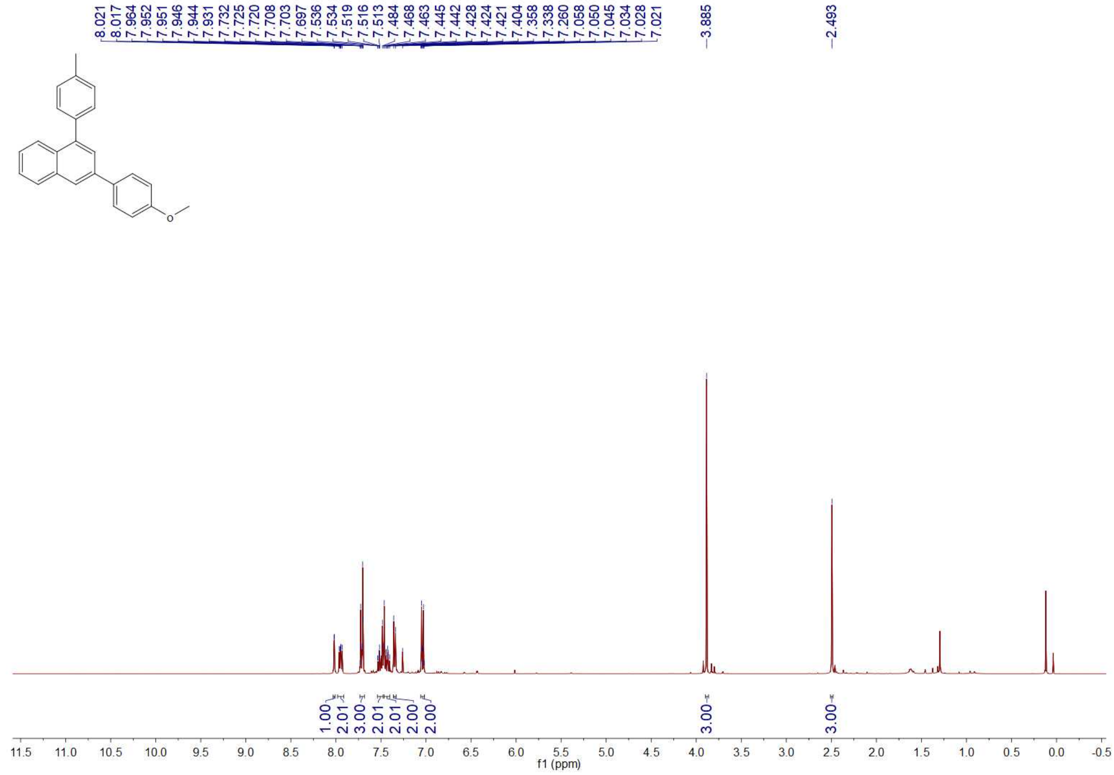

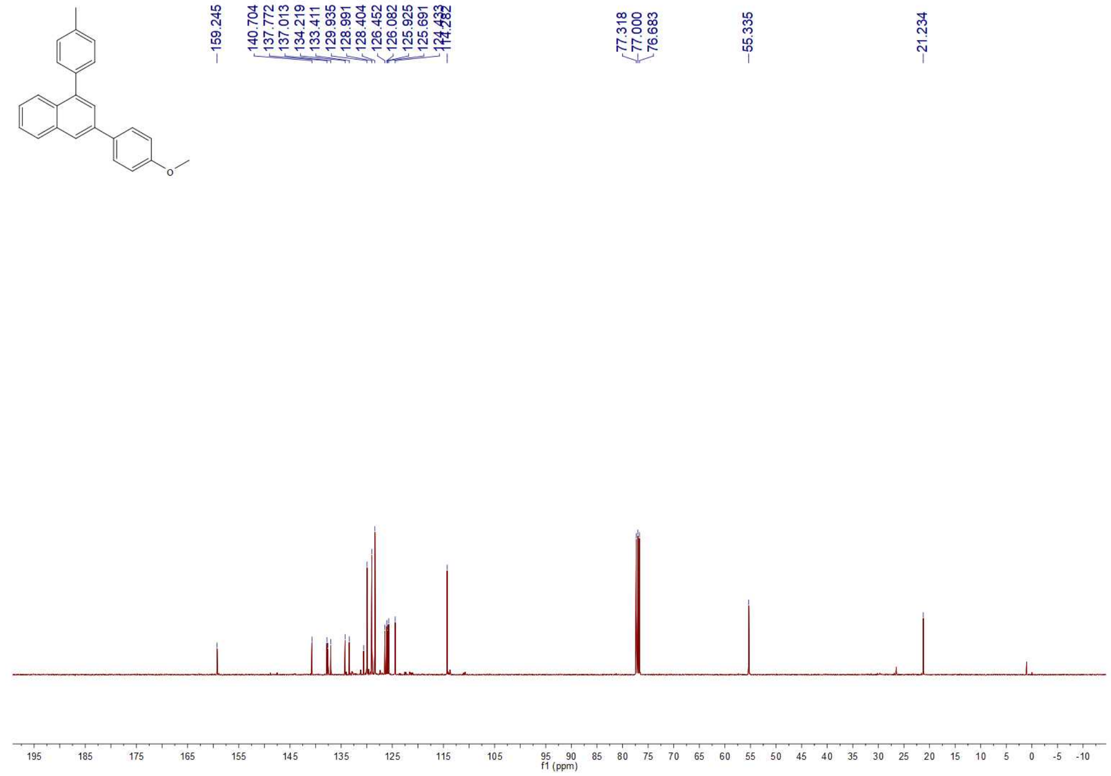

^{1}H NMR (400 MHz, $CDCl_3$) and ^{13}C NMR (100 MHz, $CDCl_3$) Spectrum of **3ao**.

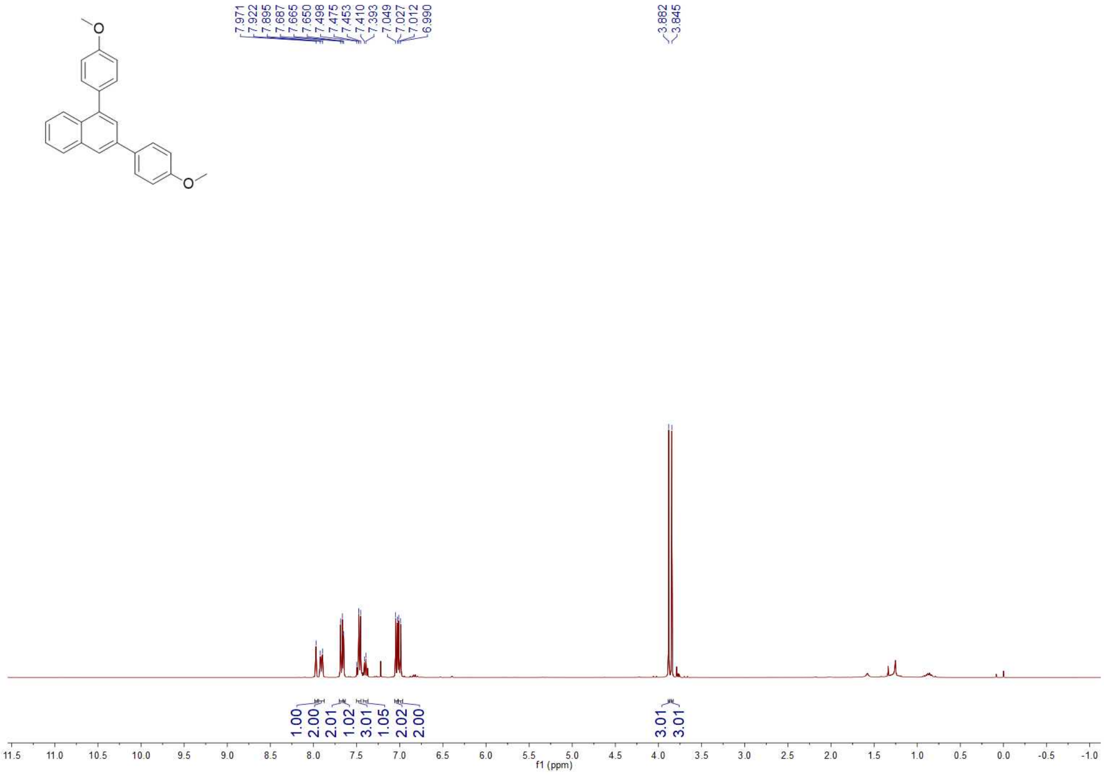

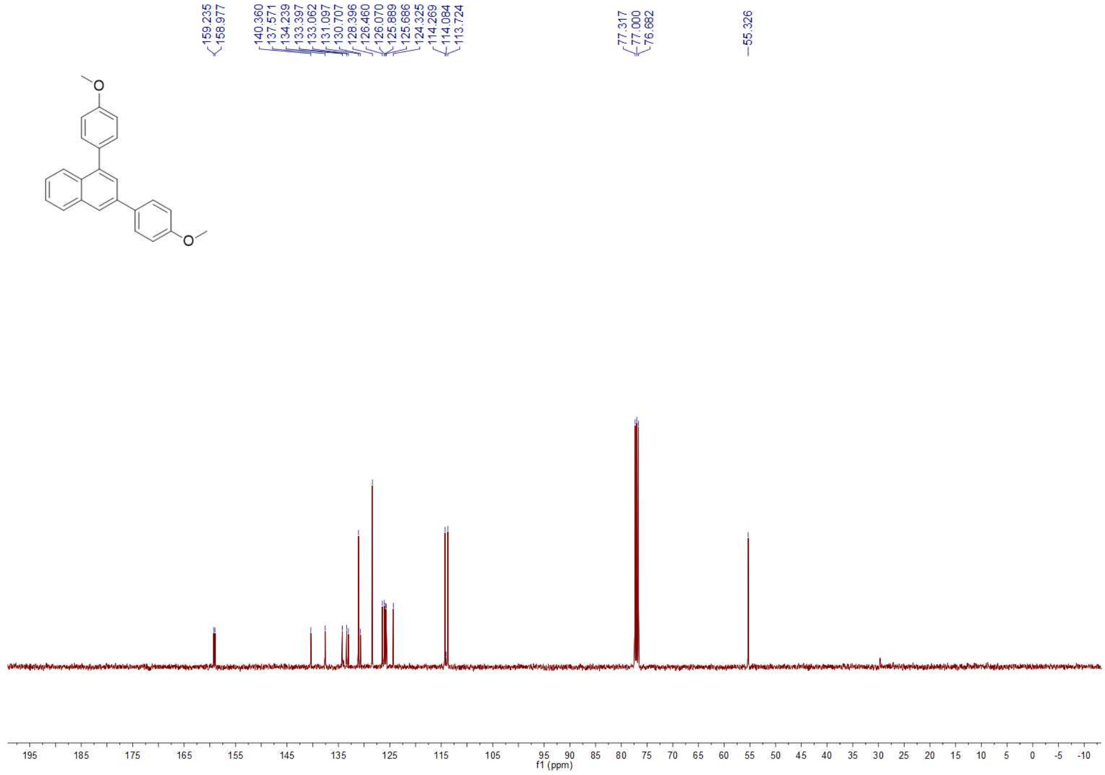

^{1}H NMR (400 MHz, $CDCl_3$) and ^{13}C NMR (100 MHz, $CDCl_3$) Spectrum of **3ap**.

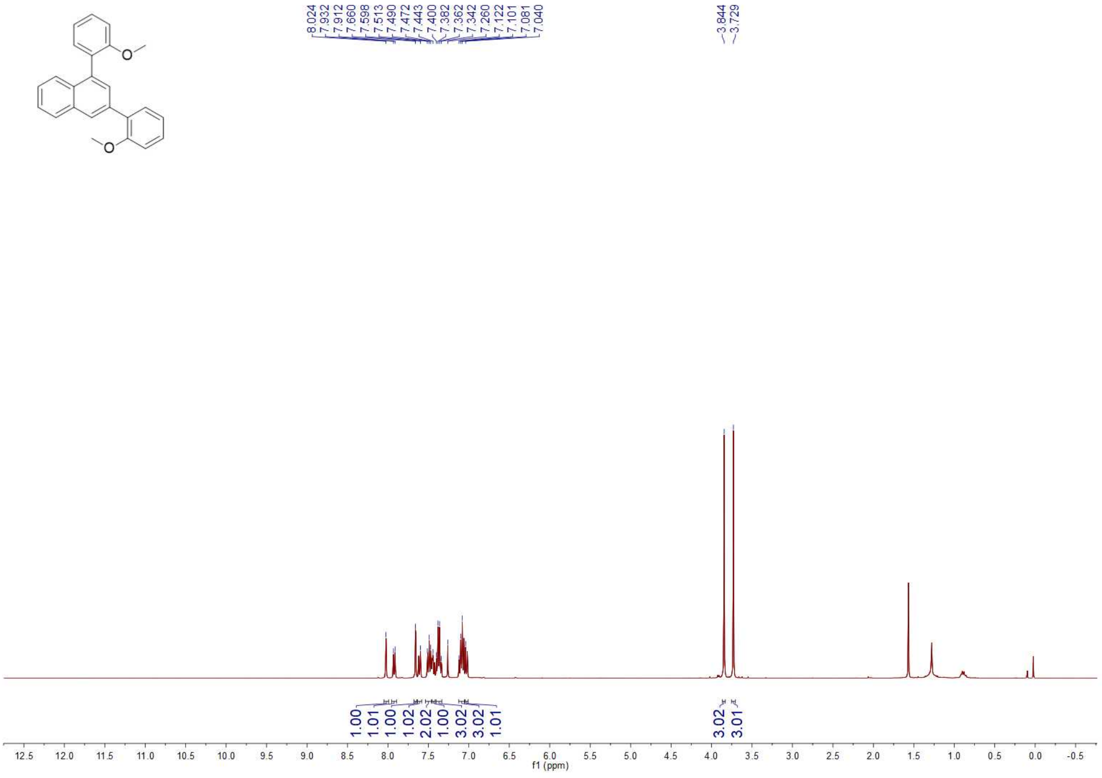

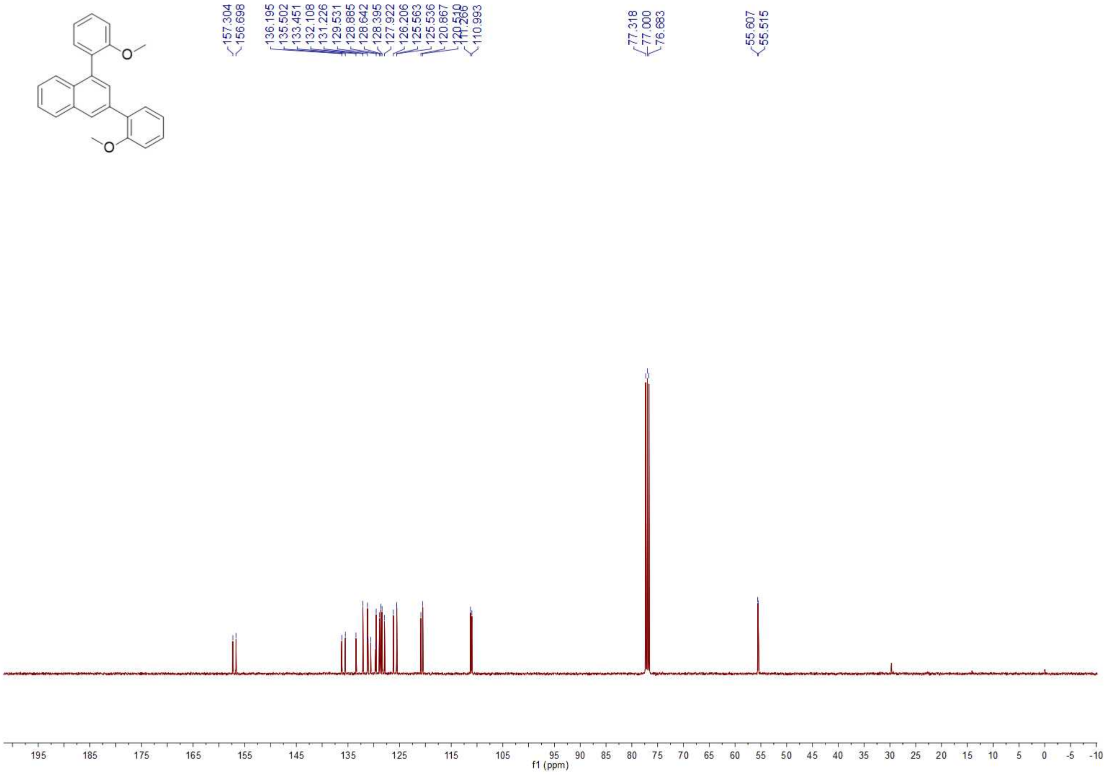

^{1}H NMR (400 MHz, $CDCl_3$) and ^{13}C NMR (100 MHz, $CDCl_3$) Spectrum of **3aq**.

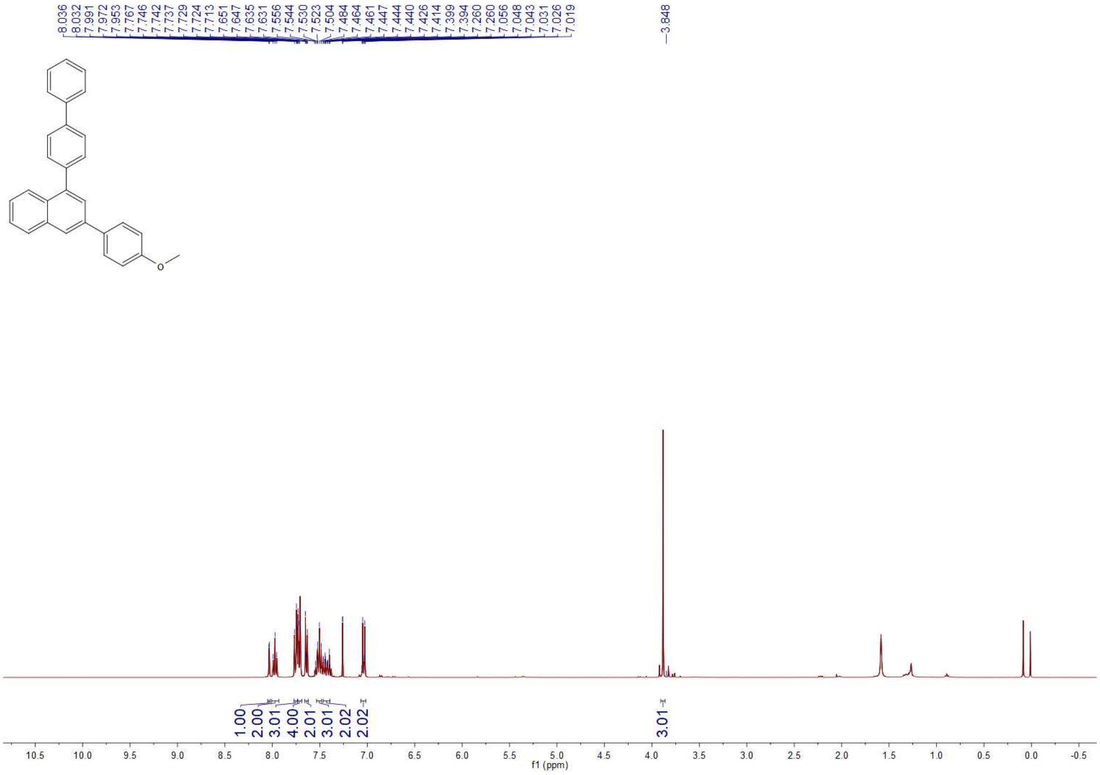

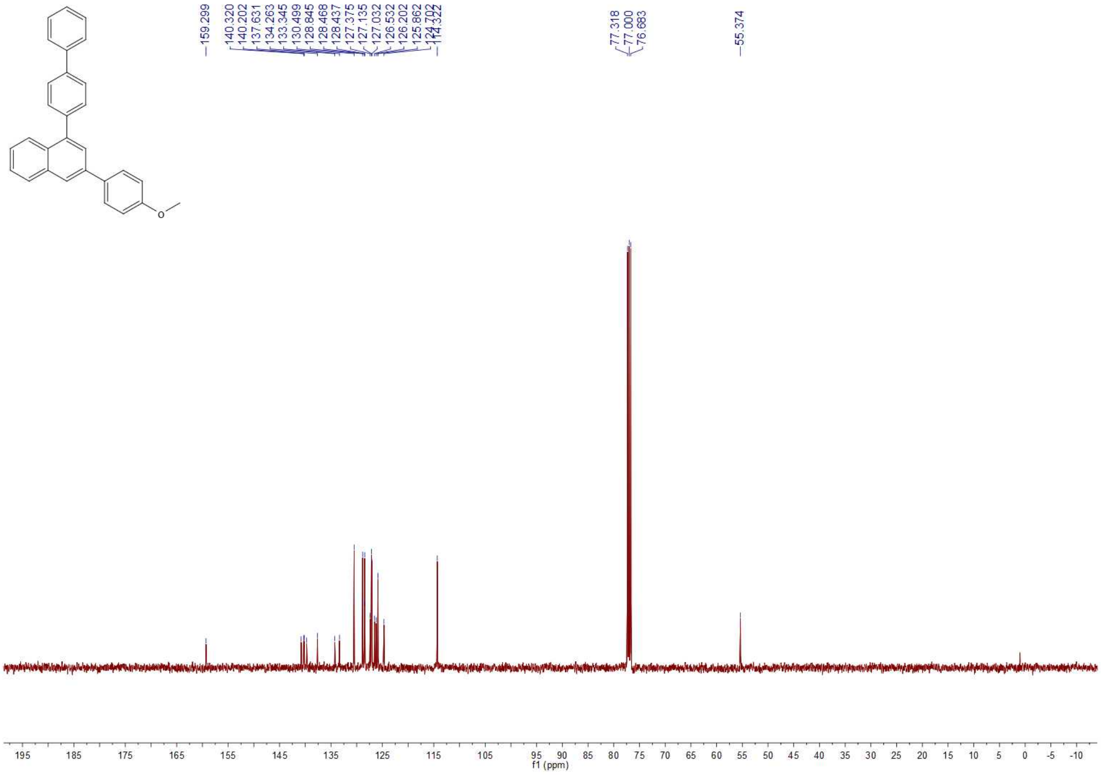

^1H NMR (400 MHz, $CDCl_3$) and ^{13}C NMR (100 MHz, $CDCl_3$) Spectrum of **3ar**.

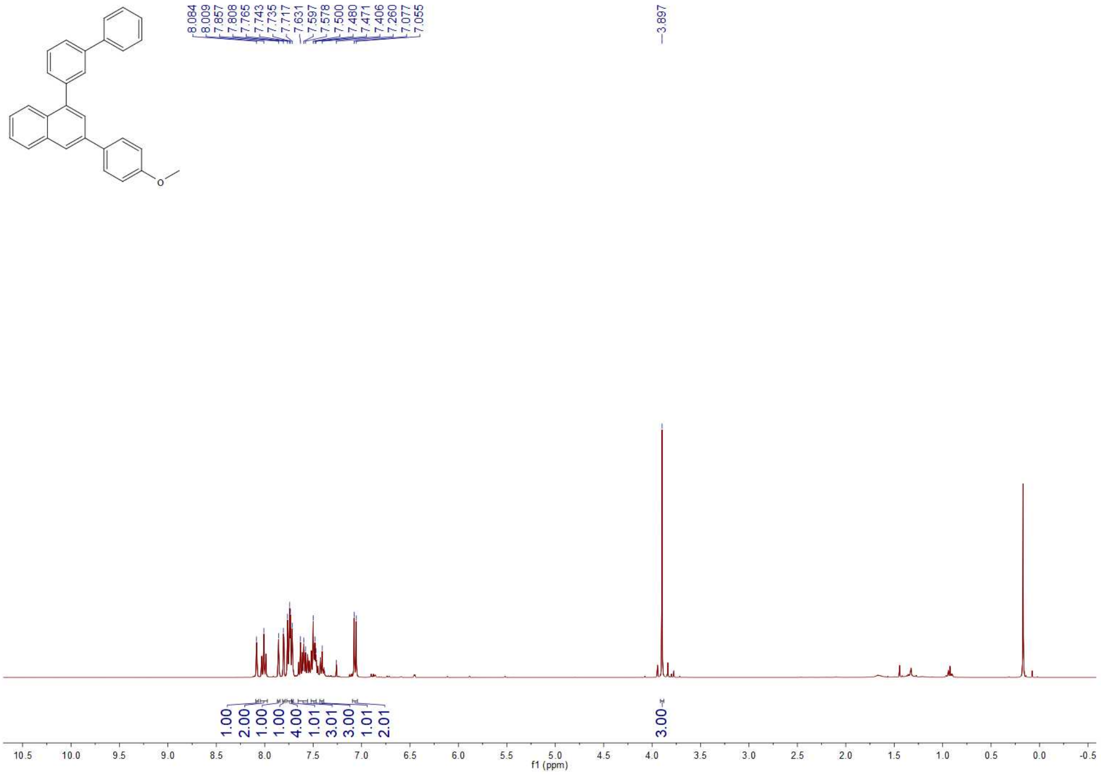

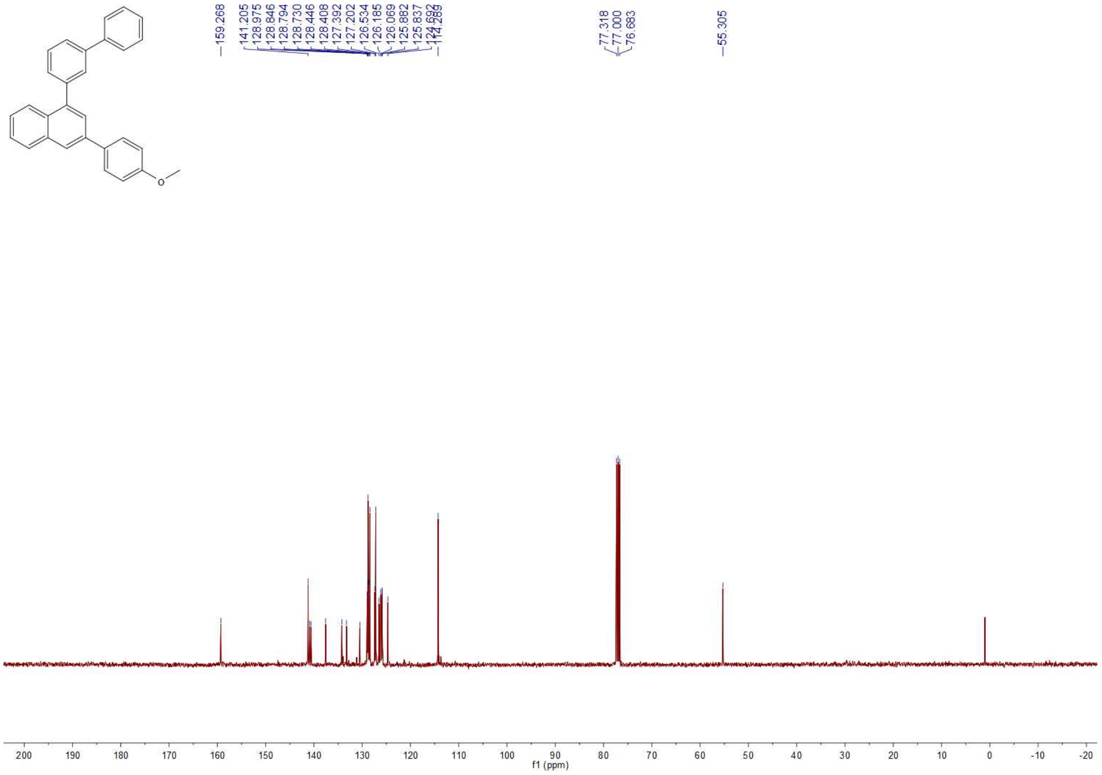

^{1}H NMR (400 MHz, $CDCl_3$) and ^{13}C NMR (100 MHz, $CDCl_3$) Spectrum of **3as**.

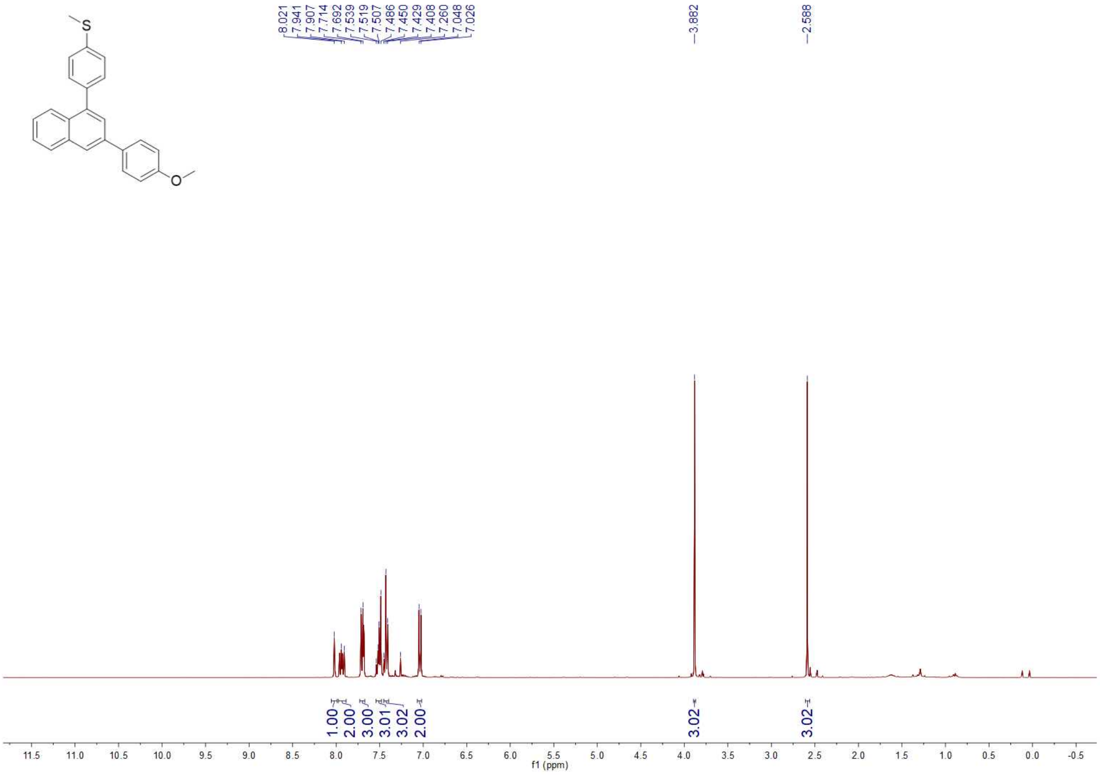

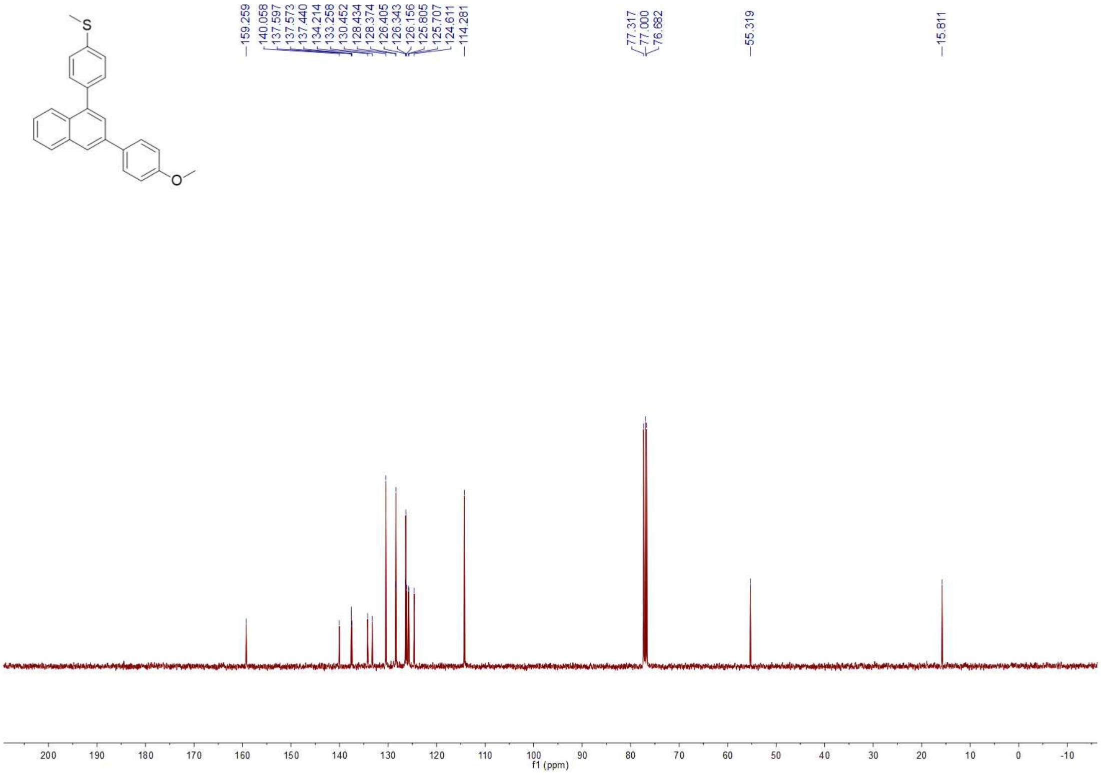

^{1}H NMR (400 MHz, $CDCl_3$), ^{13}C NMR (100 MHz, $CDCl_3$) and ^{19}F NMR (376 MHz, $CDCl_3$) Spectrum of **3at**.

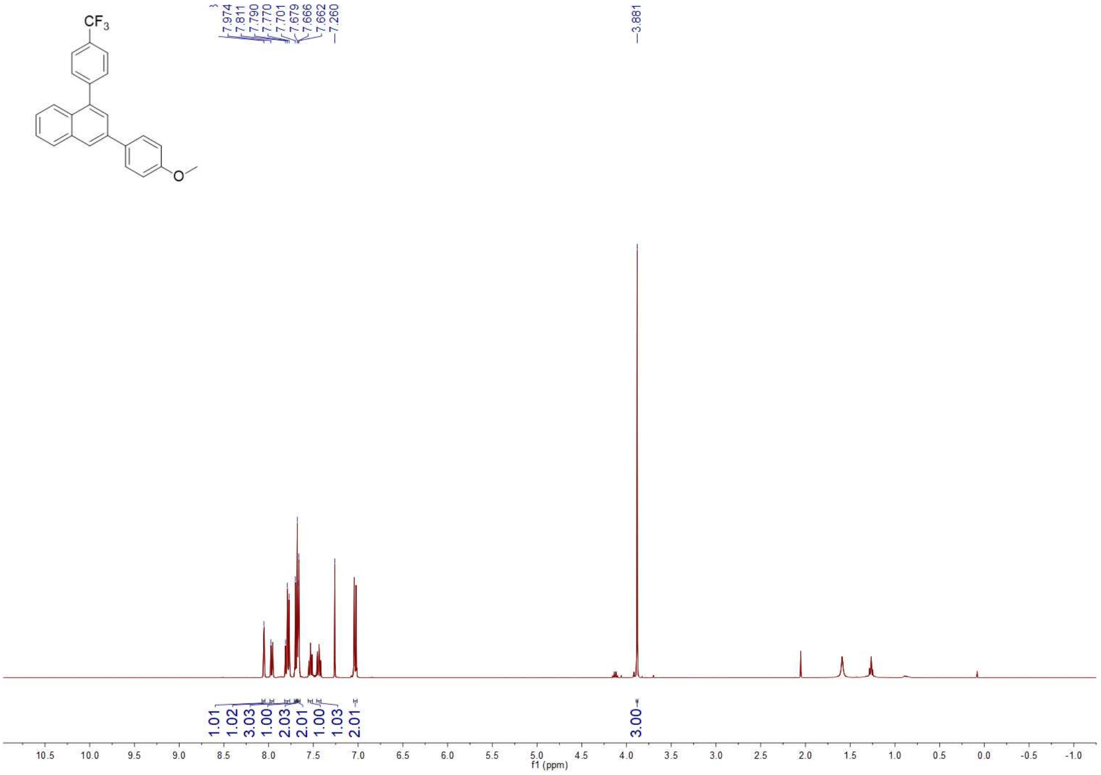

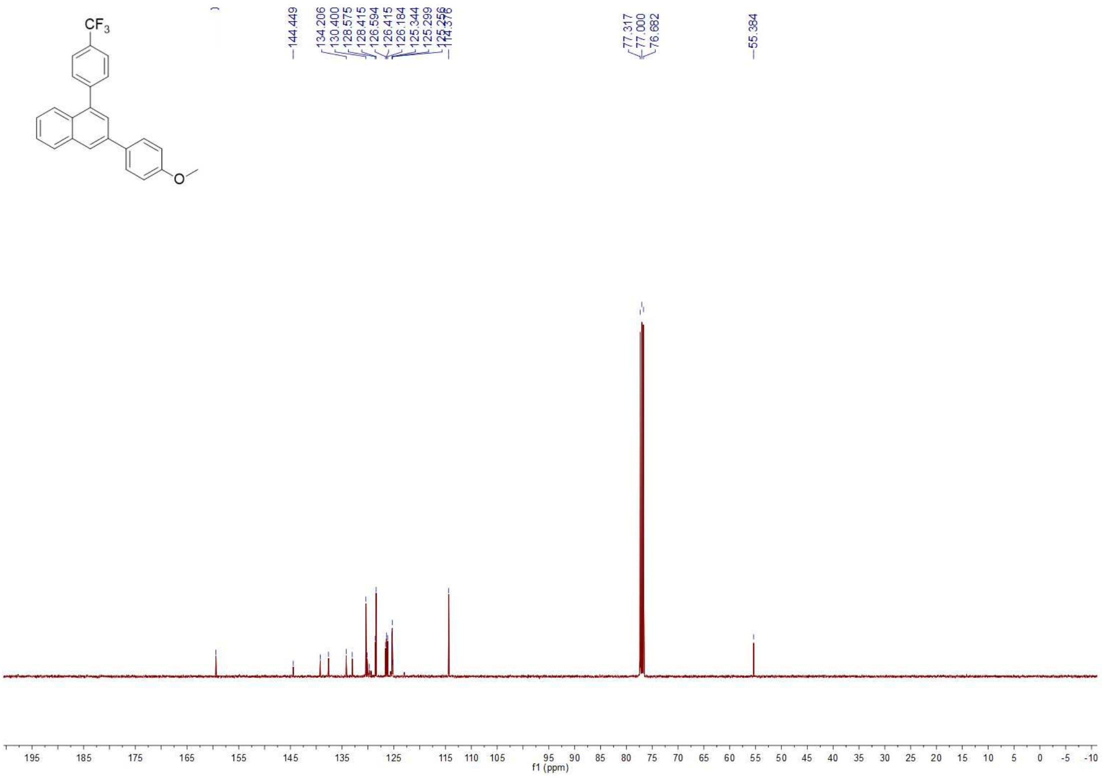

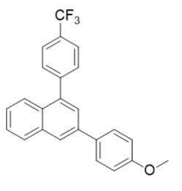

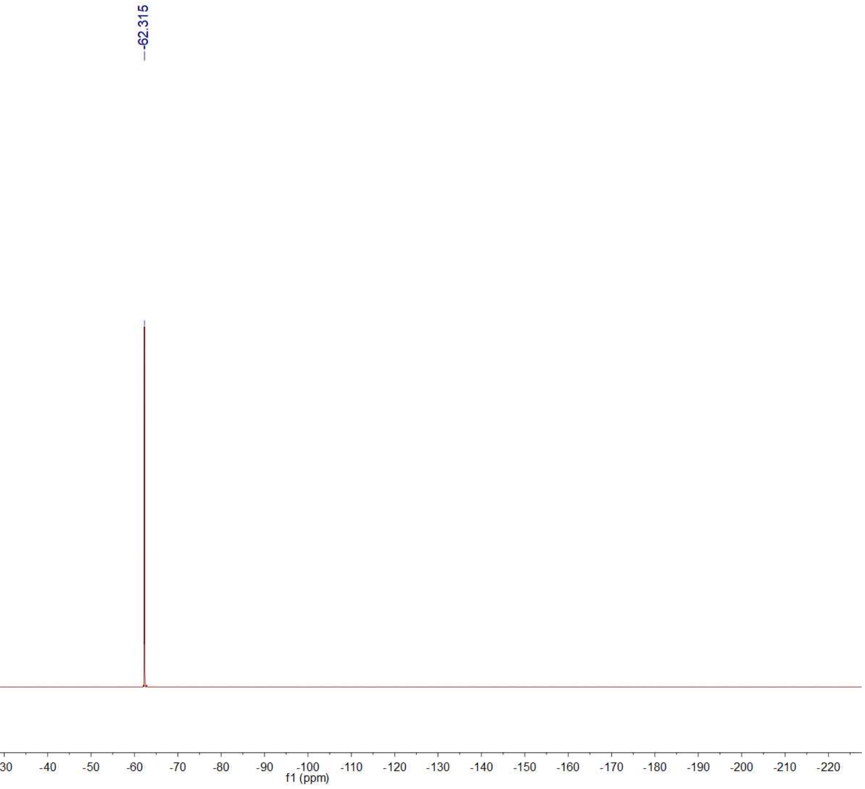

-62.315
20
10
0
-10
-20
-30
-40
-50
-60
-70
-80
-90
-100
-110
-120
-130
-140
-150
-160
-170
-180
-190
-200
-210
-220
f1 (ppm)

^{1}H NMR (400 MHz, $CDCl_3$) and ^{13}C NMR (100 MHz, $CDCl_3$) Spectrum of **3au**.

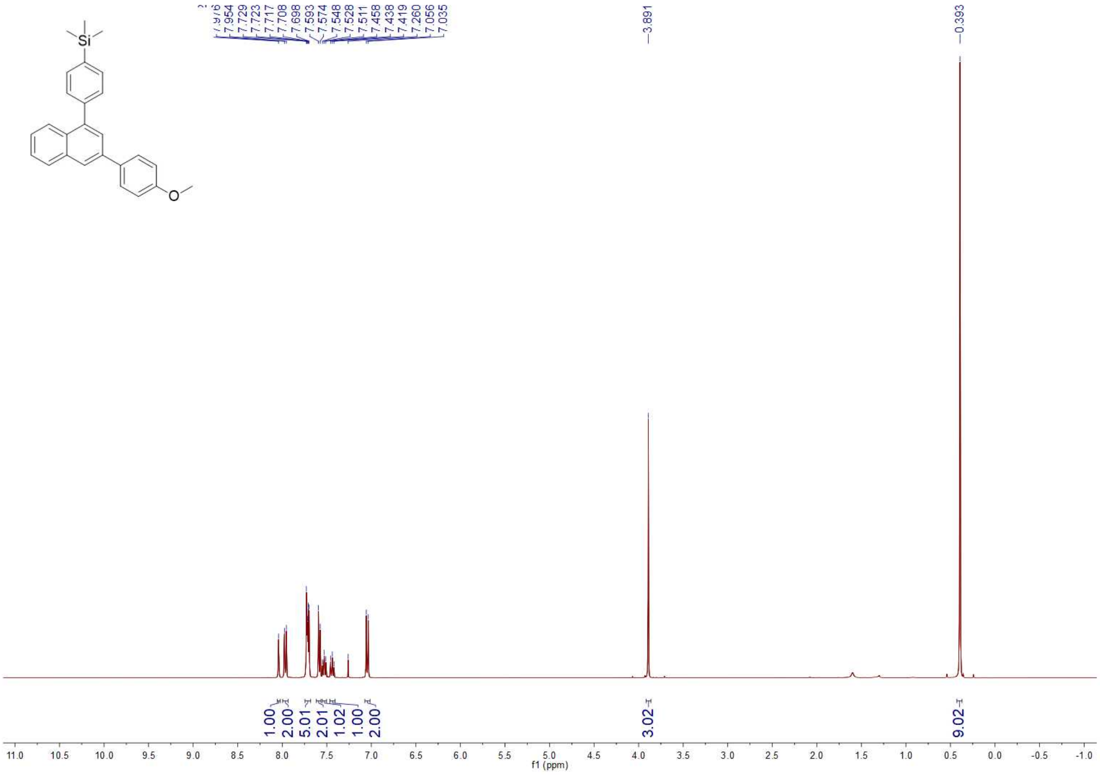

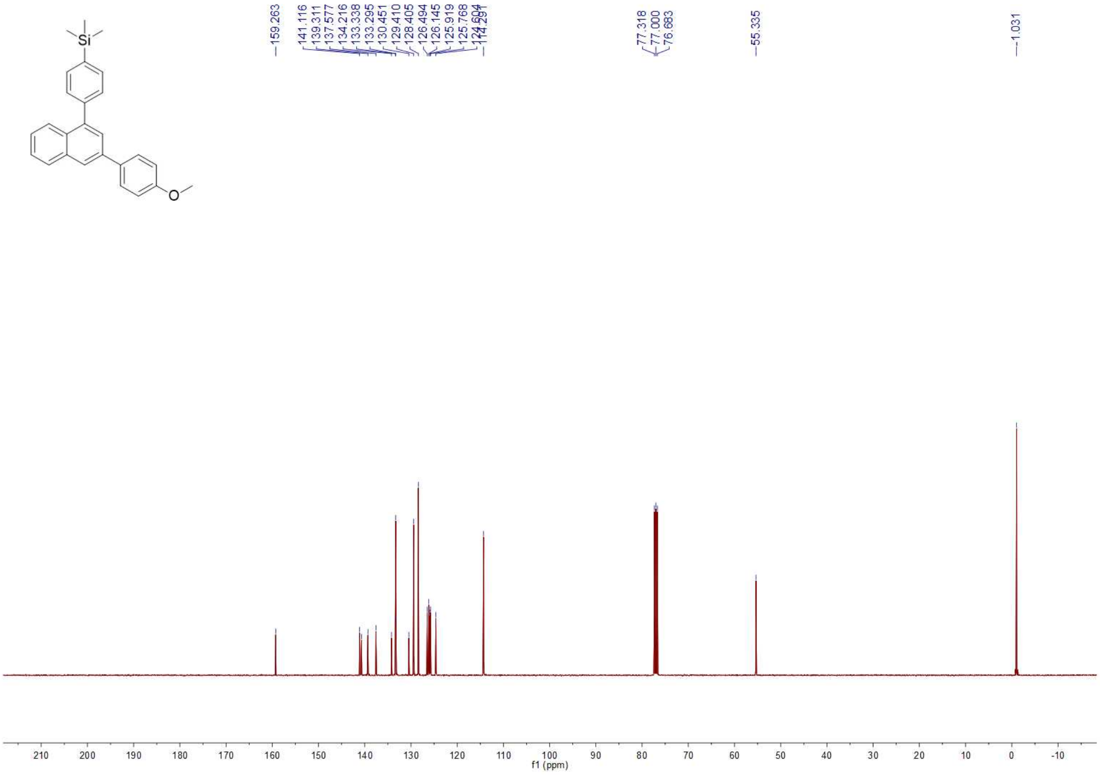

^{1}H NMR (400 MHz, $CDCl_3$) and ^{13}C NMR (100 MHz, $CDCl_3$) Spectrum of **3av**.

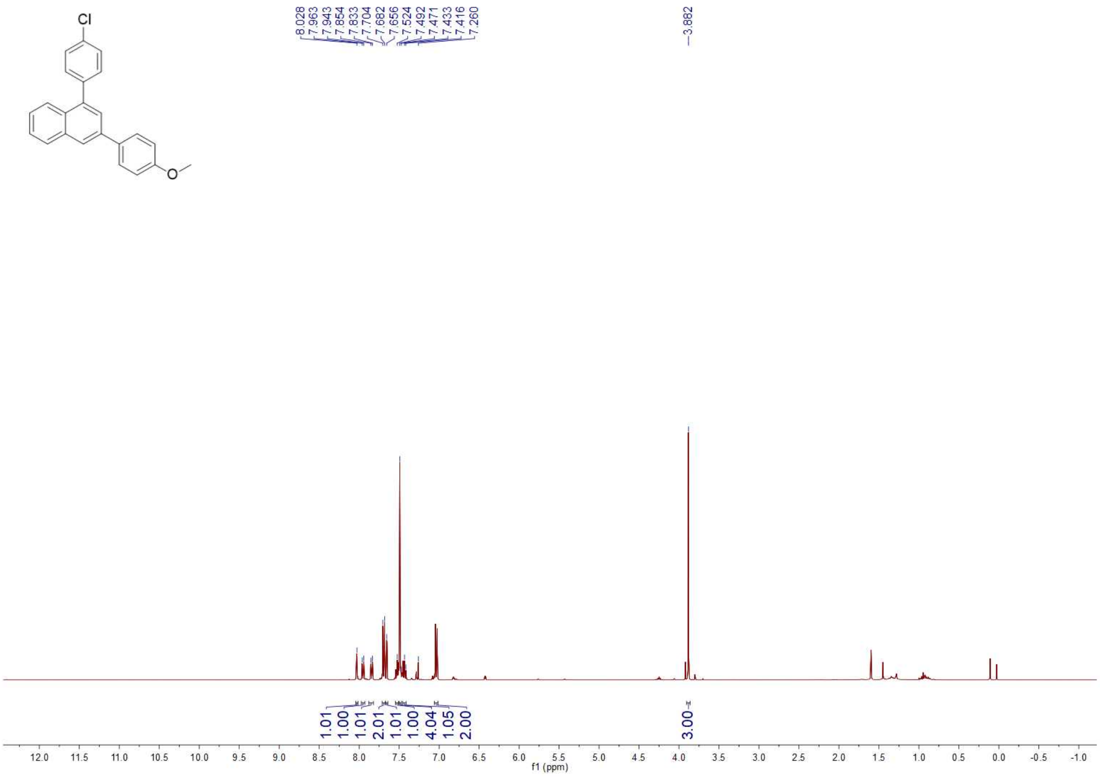

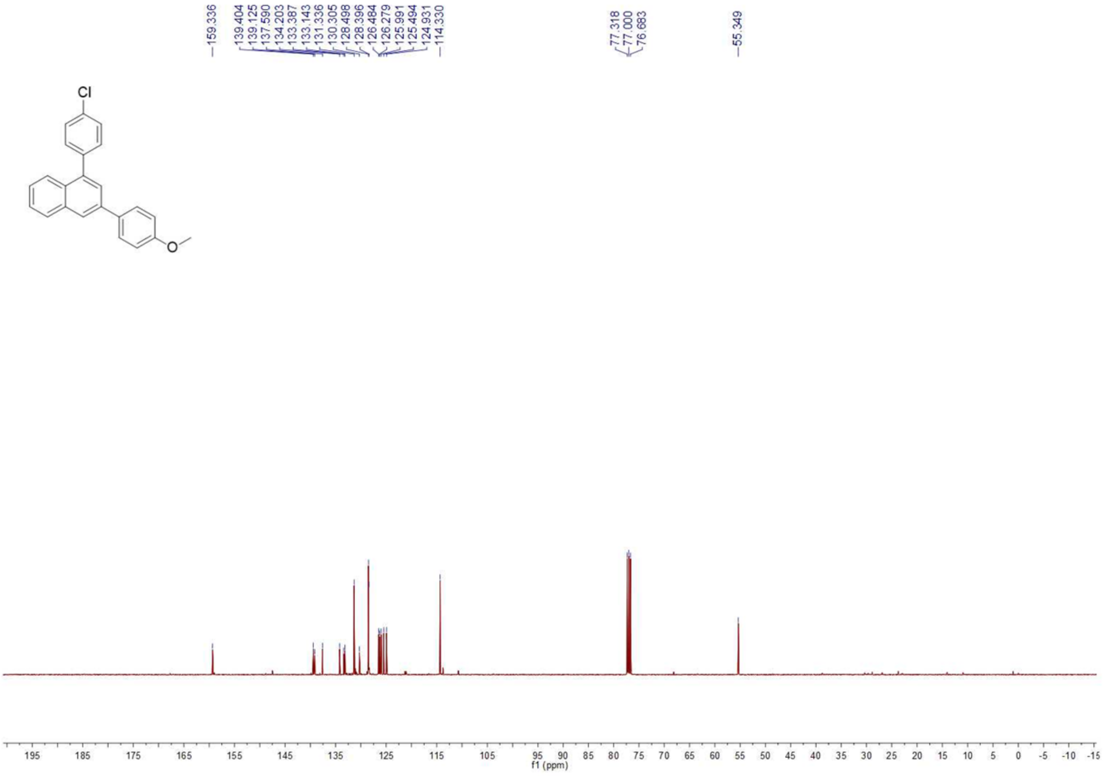

^{1}H NMR (400 MHz, $CDCl_3$) and ^{13}C NMR (100 MHz, $CDCl_3$) Spectrum of **3aw**.

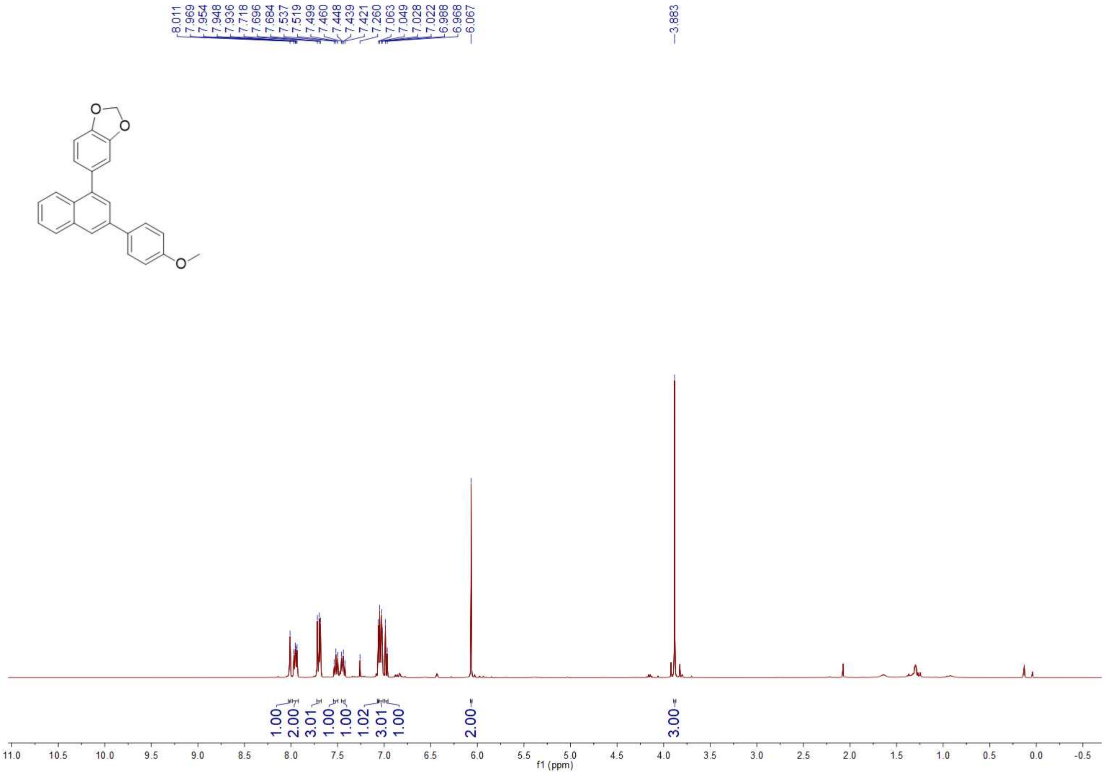

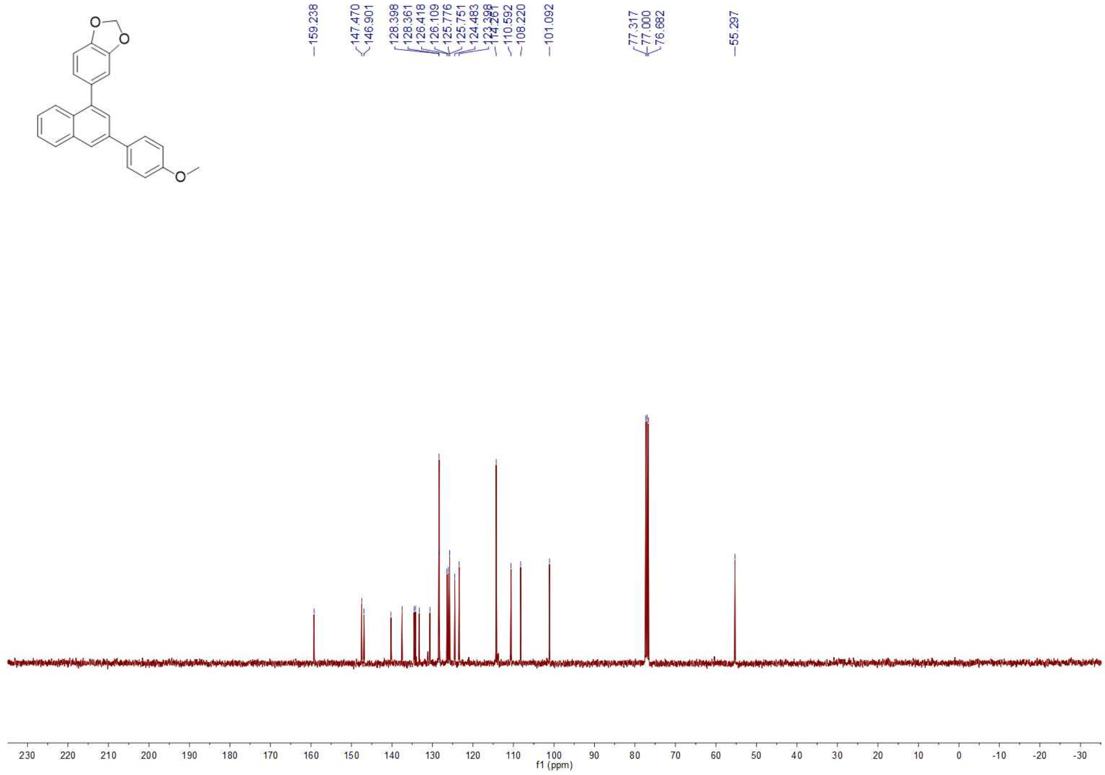

^{1}H NMR (400 MHz, $CDCl_3$) and ^{13}C NMR (100 MHz, $CDCl_3$) Spectrum of **3ax**.

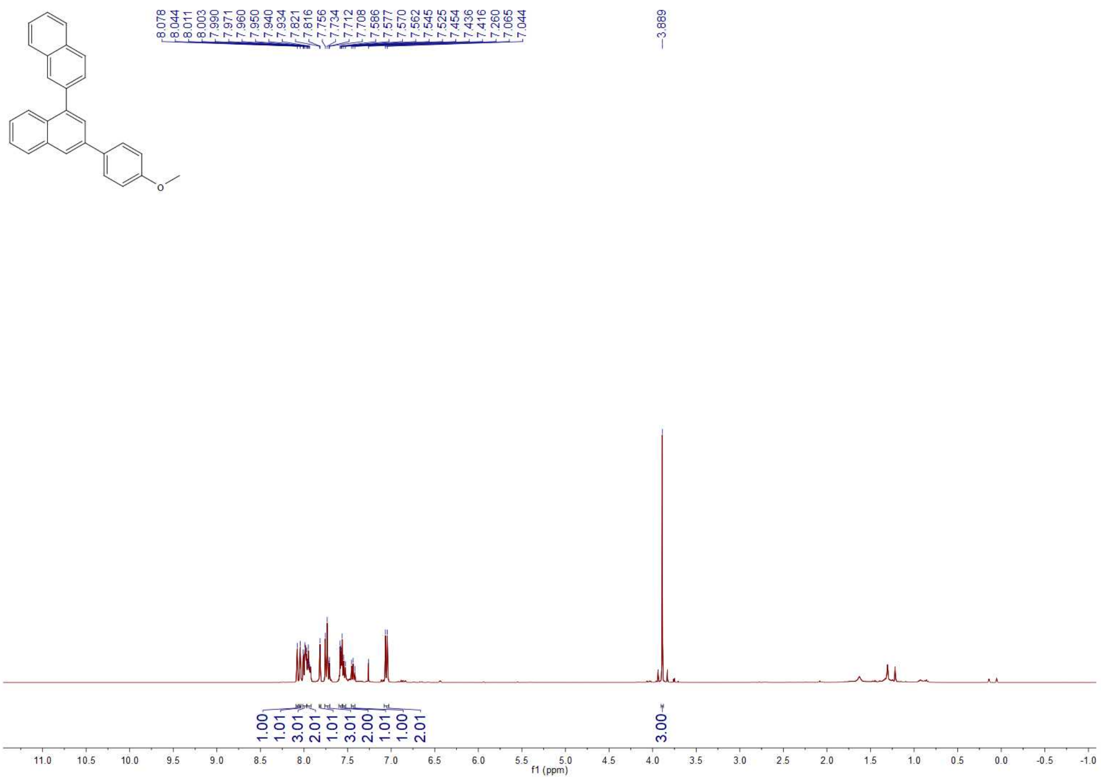

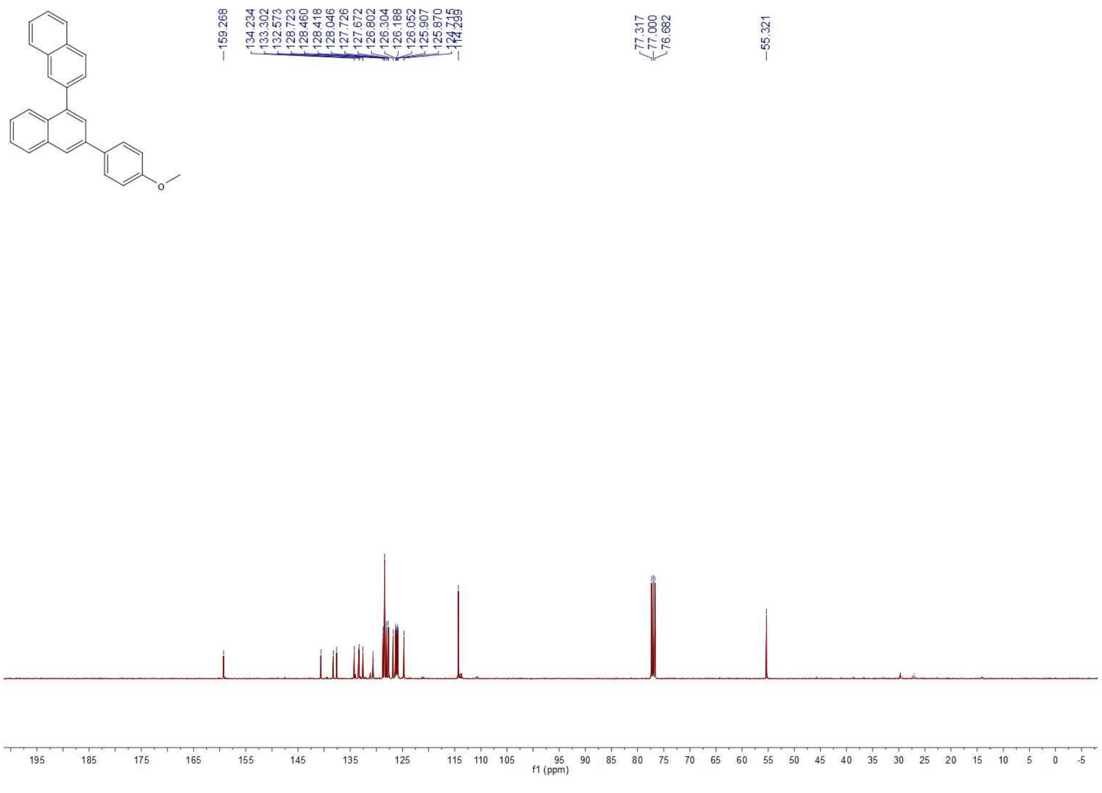

^{1}H NMR (400 MHz, $CDCl_3$) and ^{13}C NMR (100 MHz, $CDCl_3$) Spectrum of **3ay**.

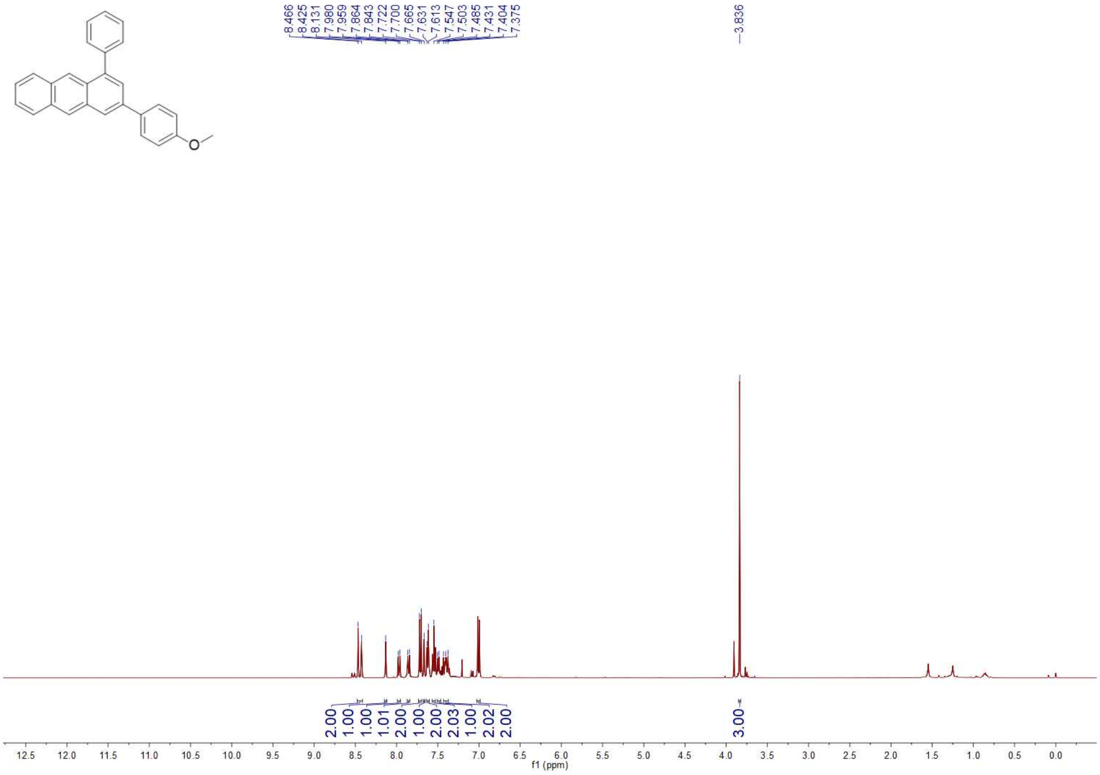

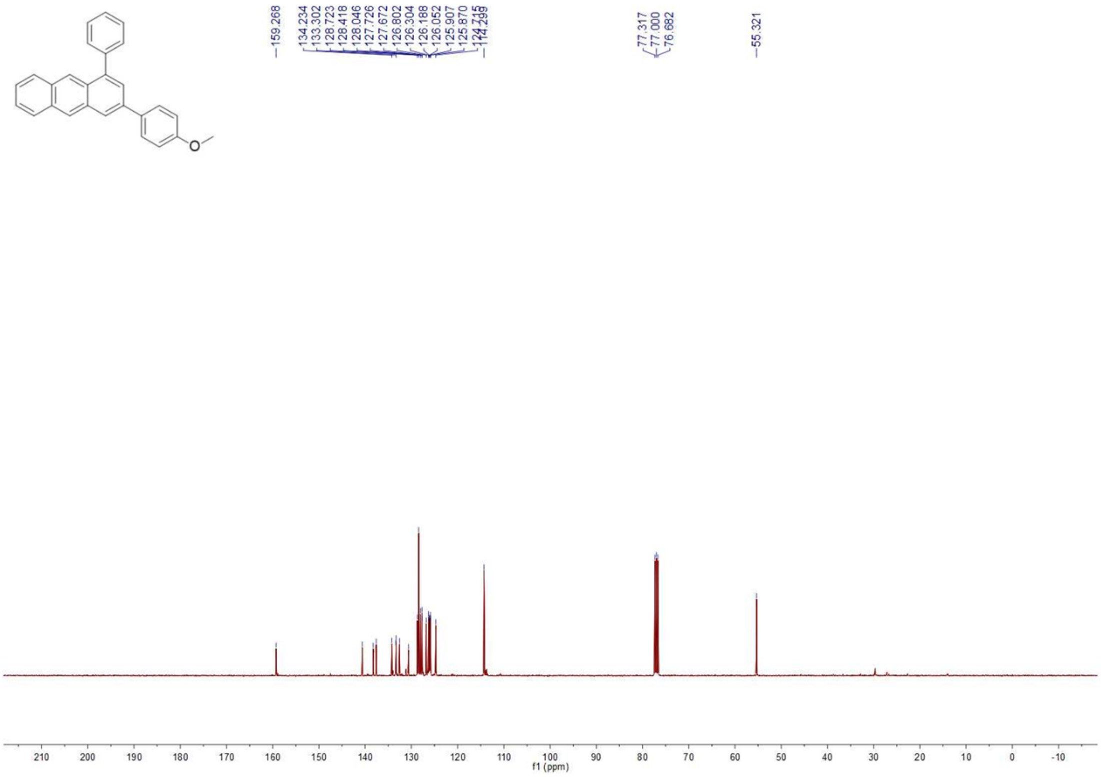

^{1}H NMR (400 MHz, $CDCl_3$) and ^{13}C NMR (100 MHz, $CDCl_3$) Spectrum of **3az**.

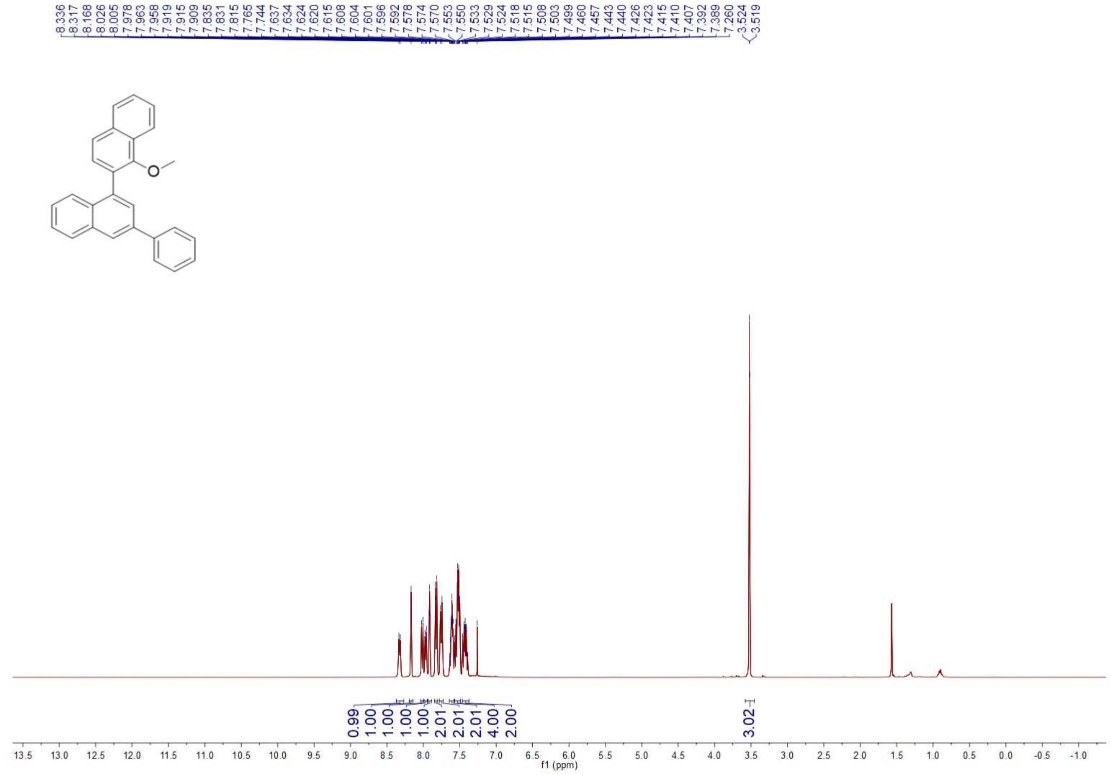

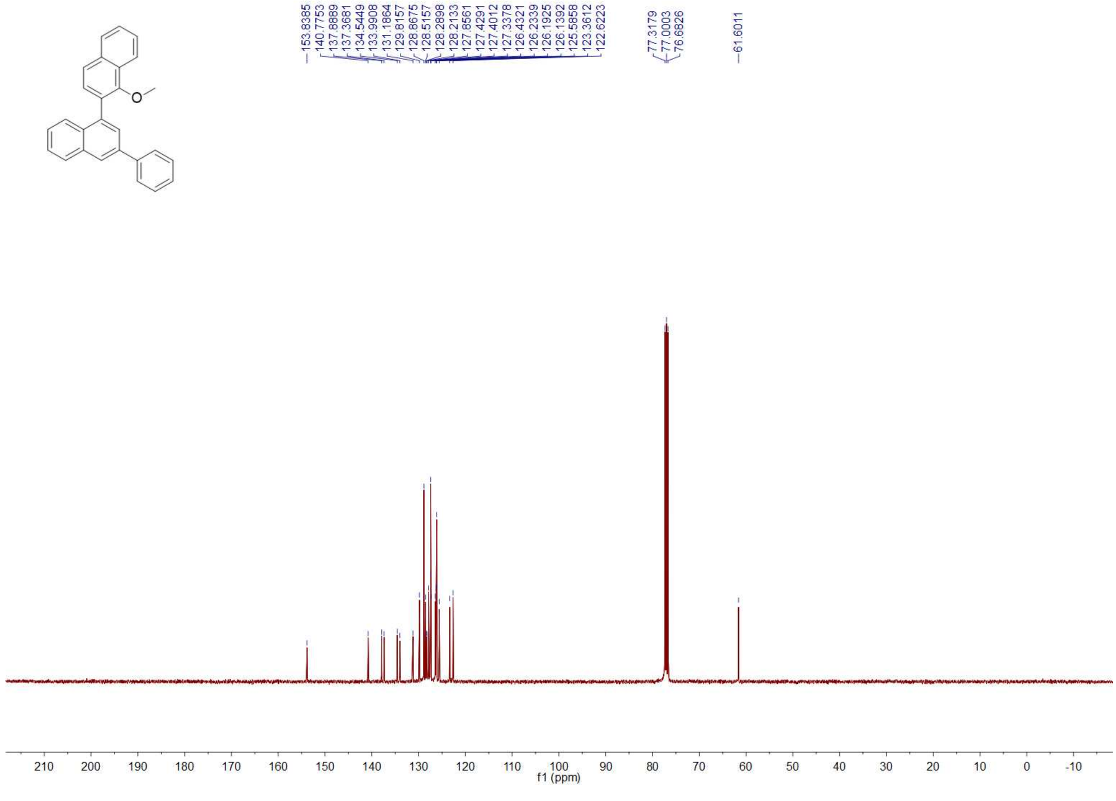

^{1}H NMR (400 MHz, $CDCl_3$) and ^{13}C NMR (100 MHz, $CDCl_3$) Spectrum of **3ba**.

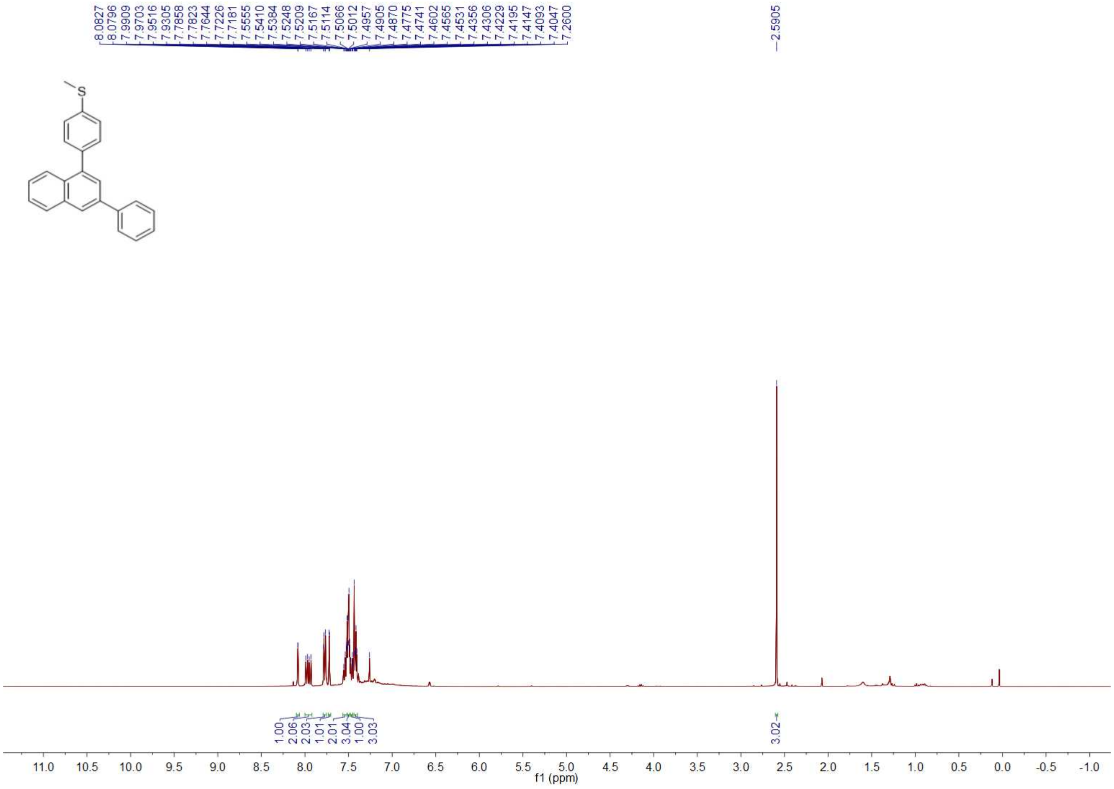

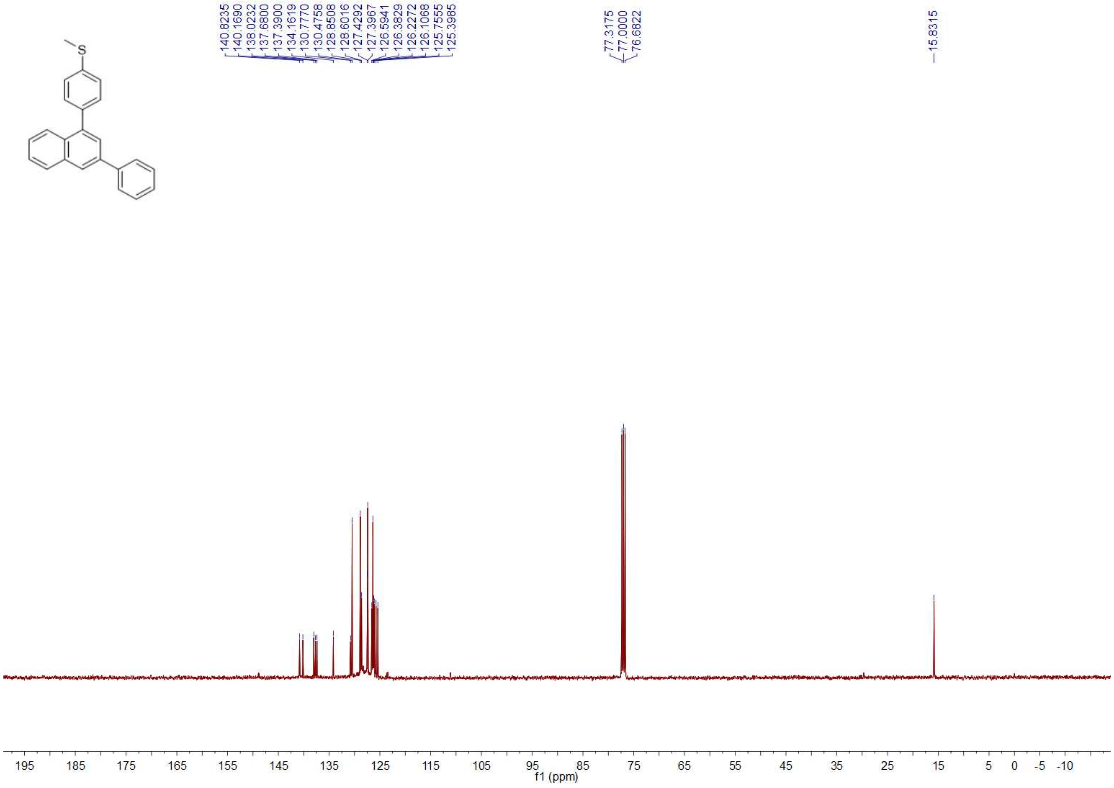

^{1}H NMR (400 MHz, $CDCl_3$) and ^{13}C NMR (100 MHz, $CDCl_3$) Spectrum of **3bb**.

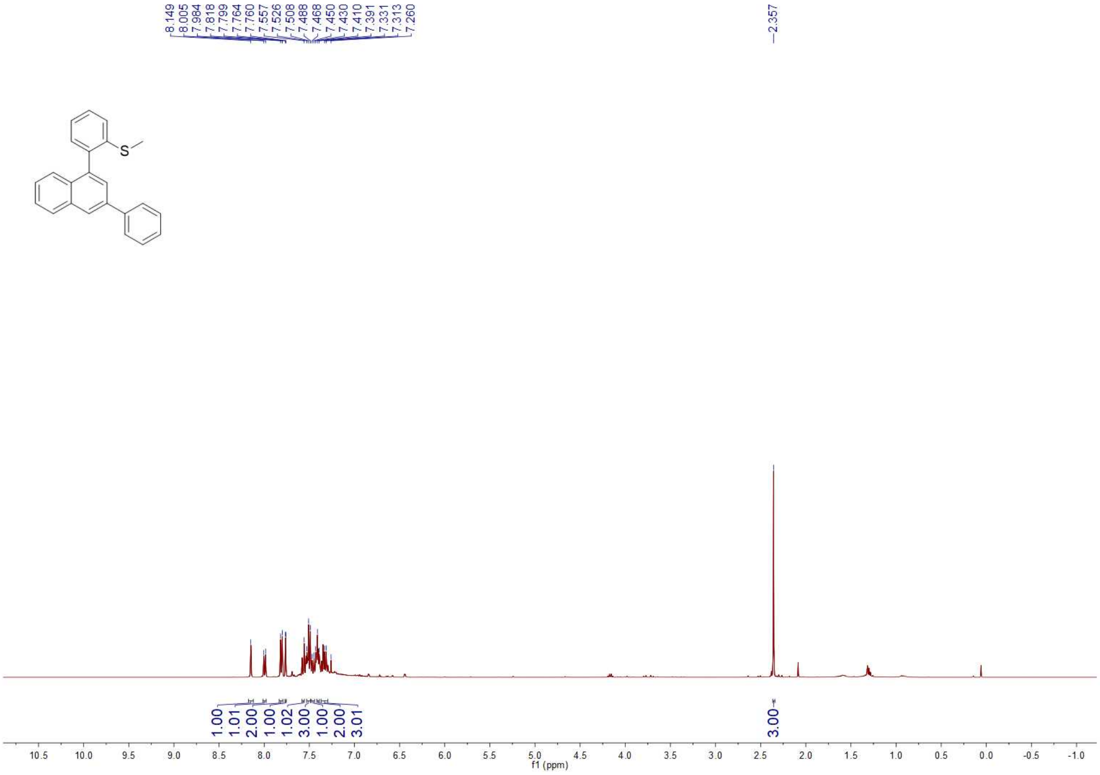

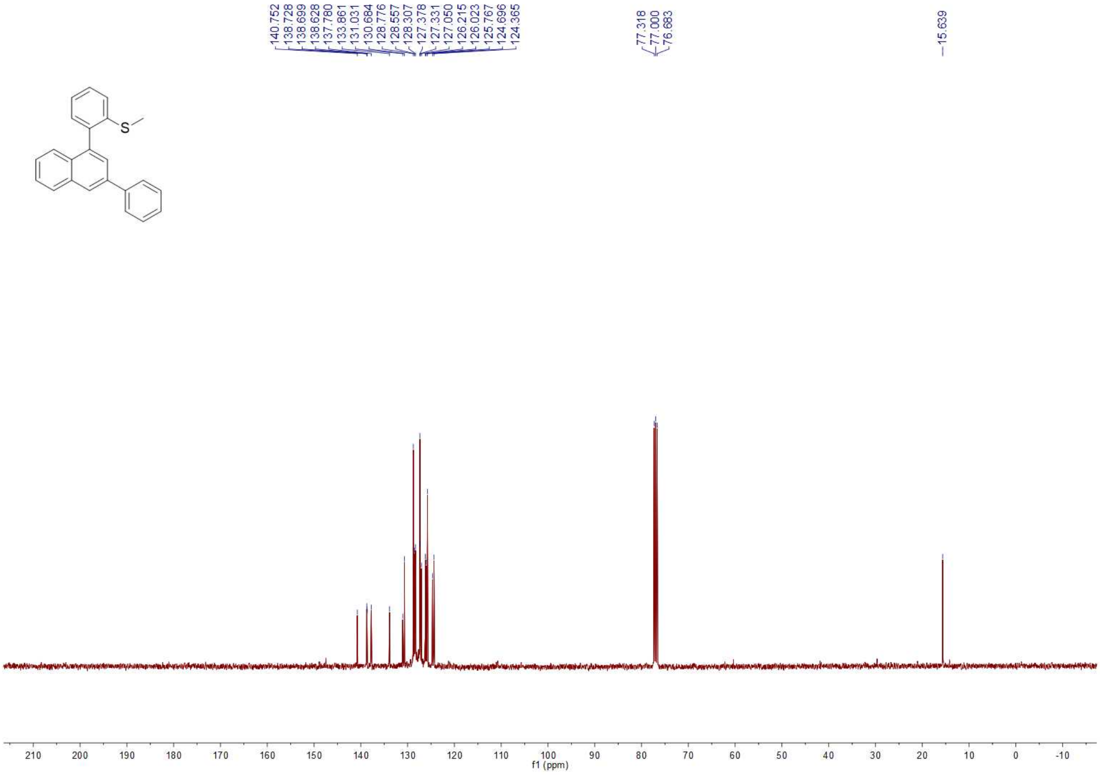

^{1}H NMR (400 MHz, $CDCl_3$) and ^{13}C NMR (100 MHz, $CDCl_3$) Spectrum of **3bc**.

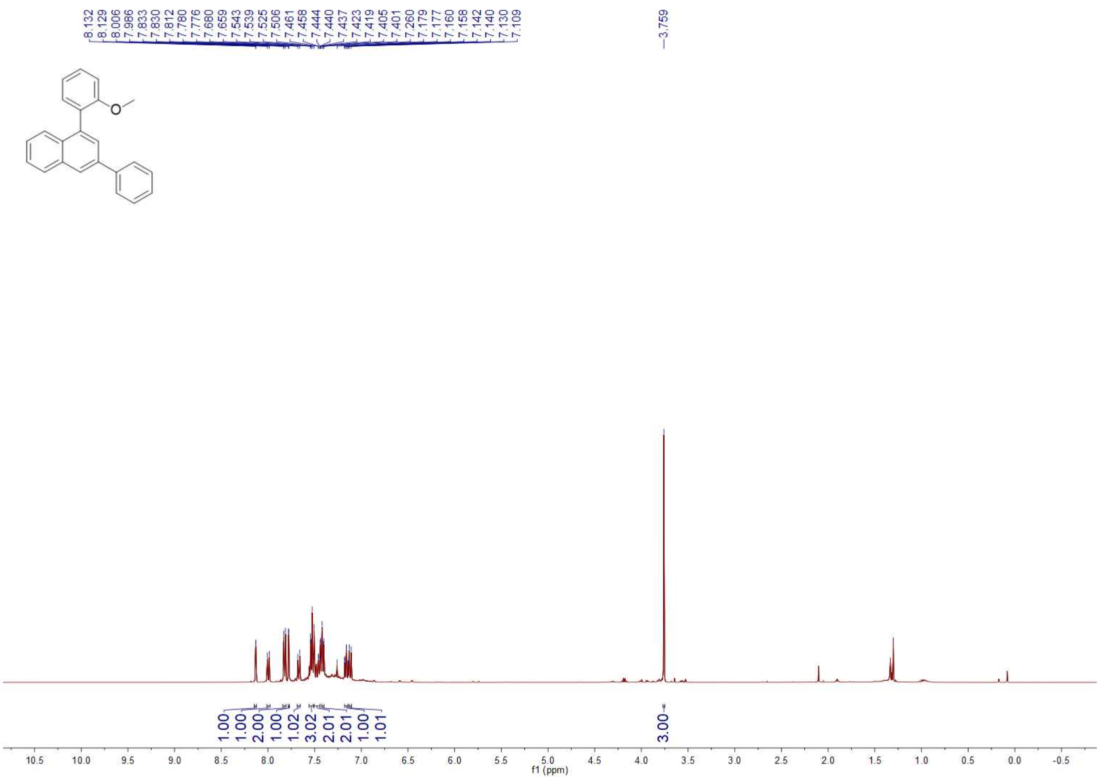

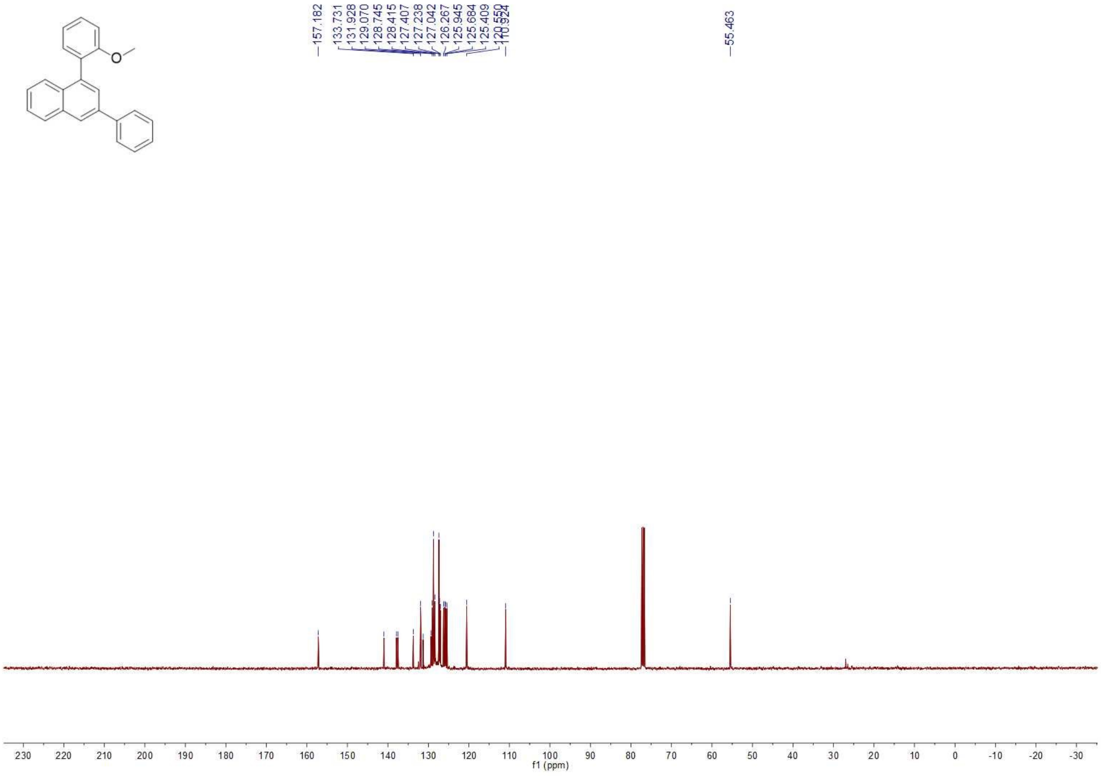

^1H NMR (400 MHz, $CDCl_3$), ^{13}C NMR (100 MHz, $CDCl_3$) and ^{19}F NMR (376 MHz, $CDCl_3$) Spectrum of **3bd**.

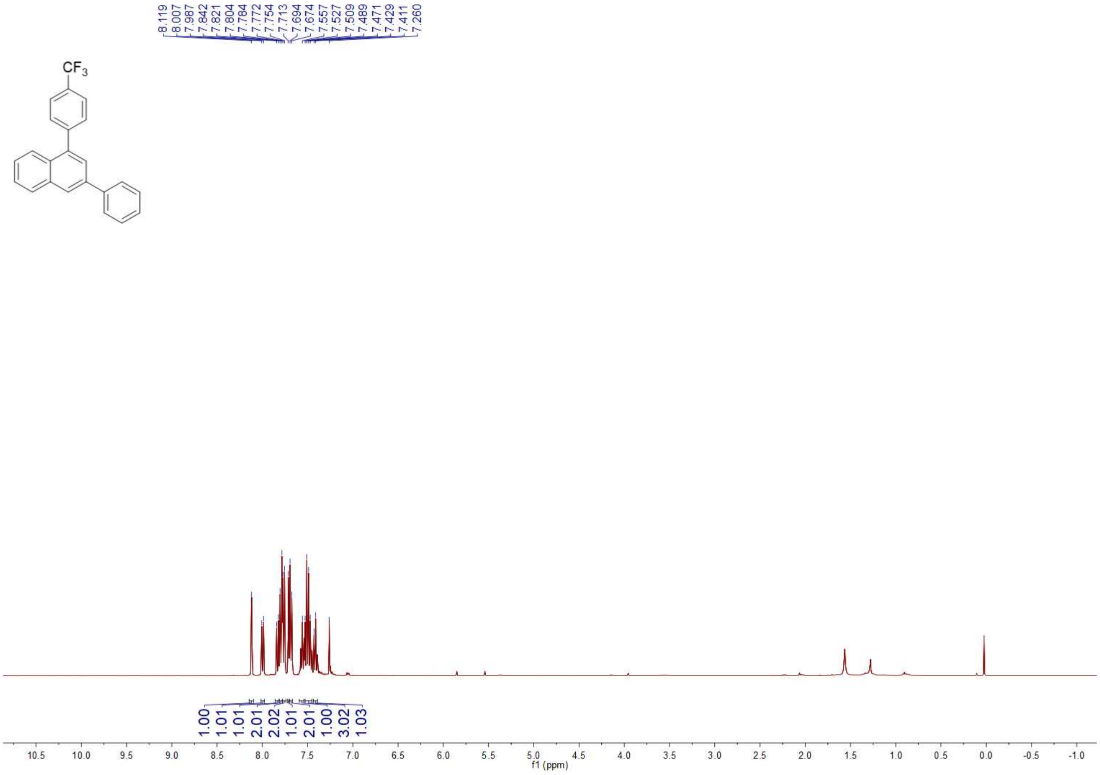

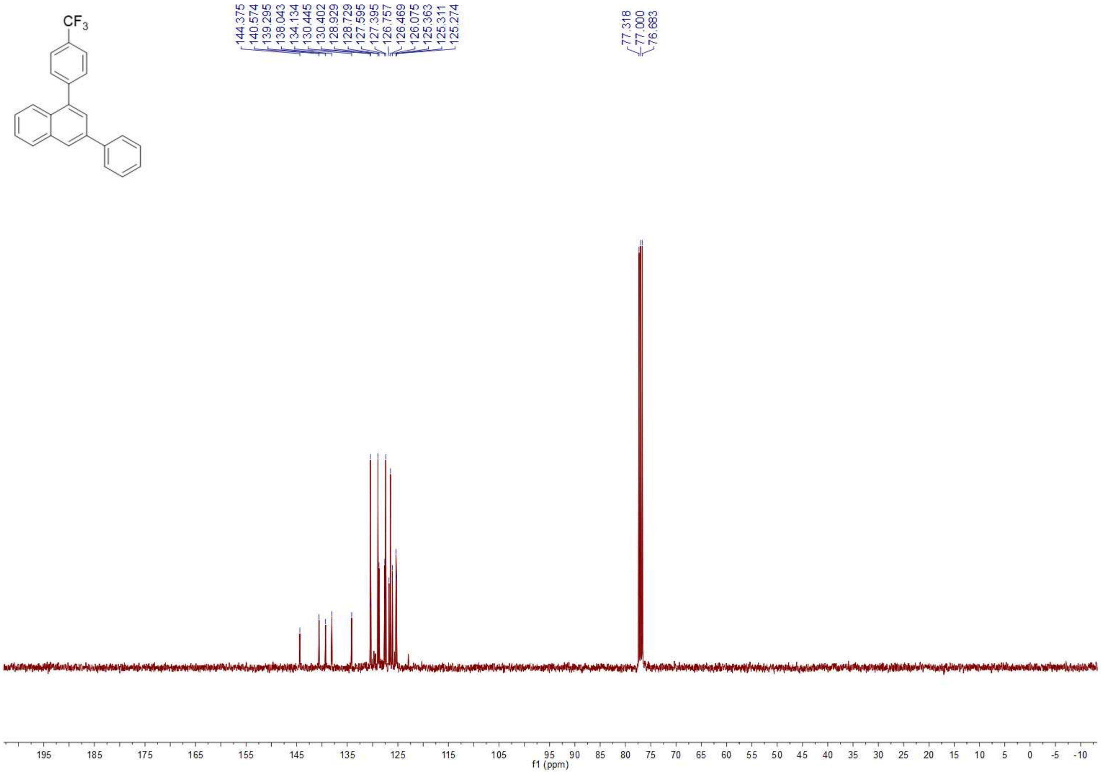

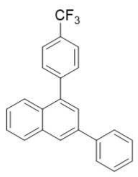
CF3

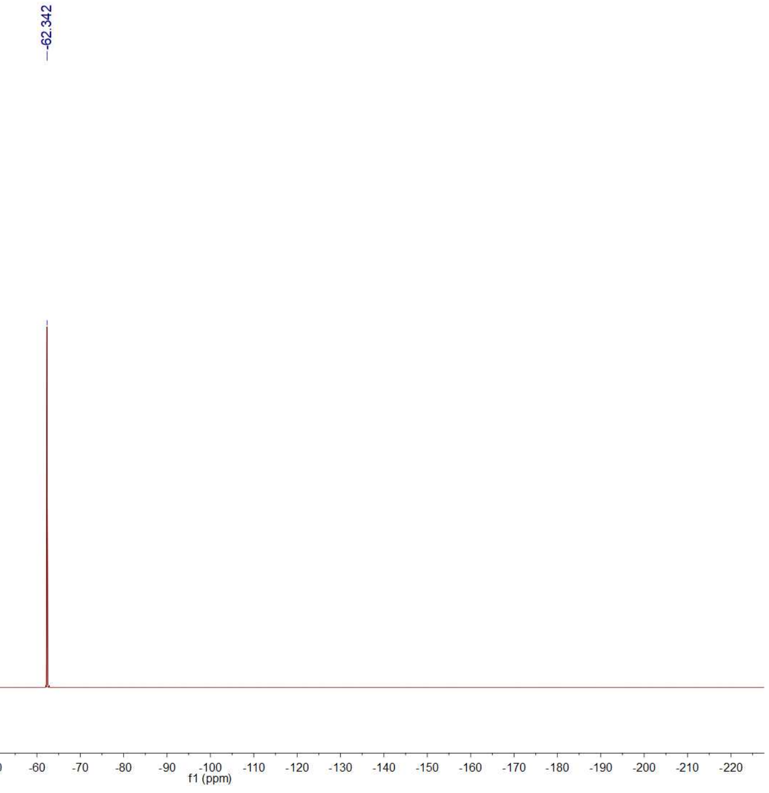
-62.342
20
10
0
-10
-20
-30
-40
-50
-60
-70
-80
-90
-100
-110
-120
-130
-140
-150
-160
-170
-180
-190
-200
-210
-220
f1 (ppm)

^{1}H NMR (400 MHz, $CDCl_3$) and ^{13}C NMR (100 MHz, $CDCl_3$) Spectrum of **3be**.

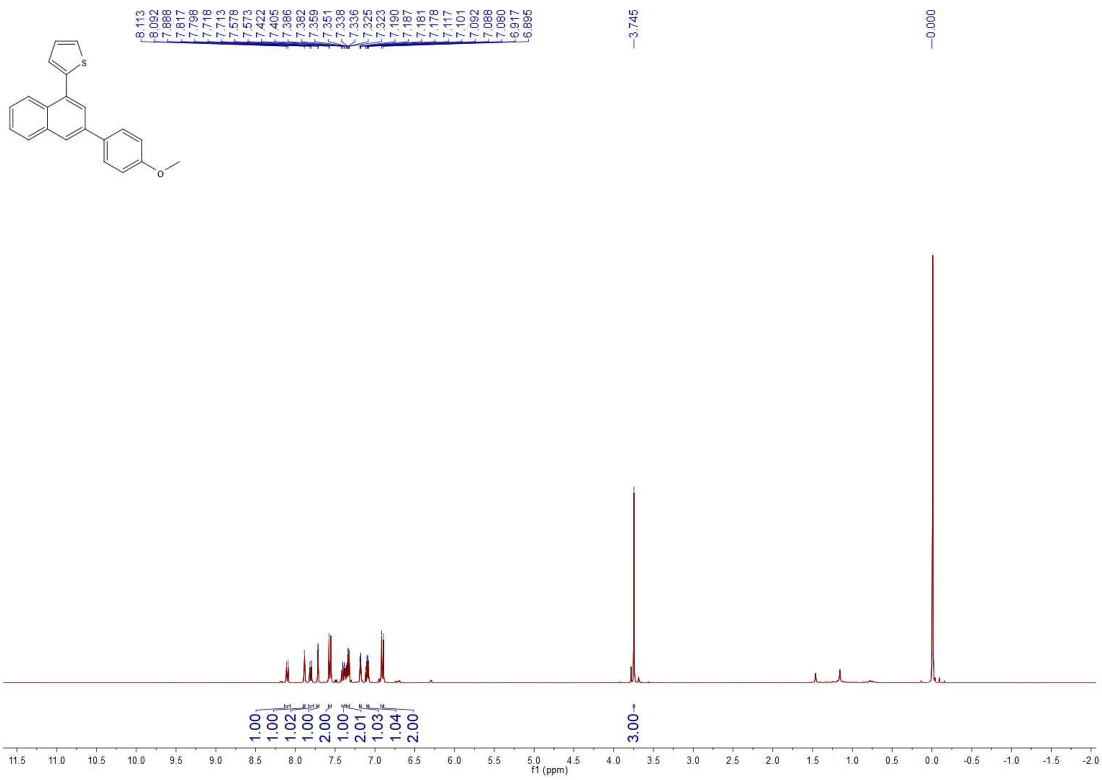

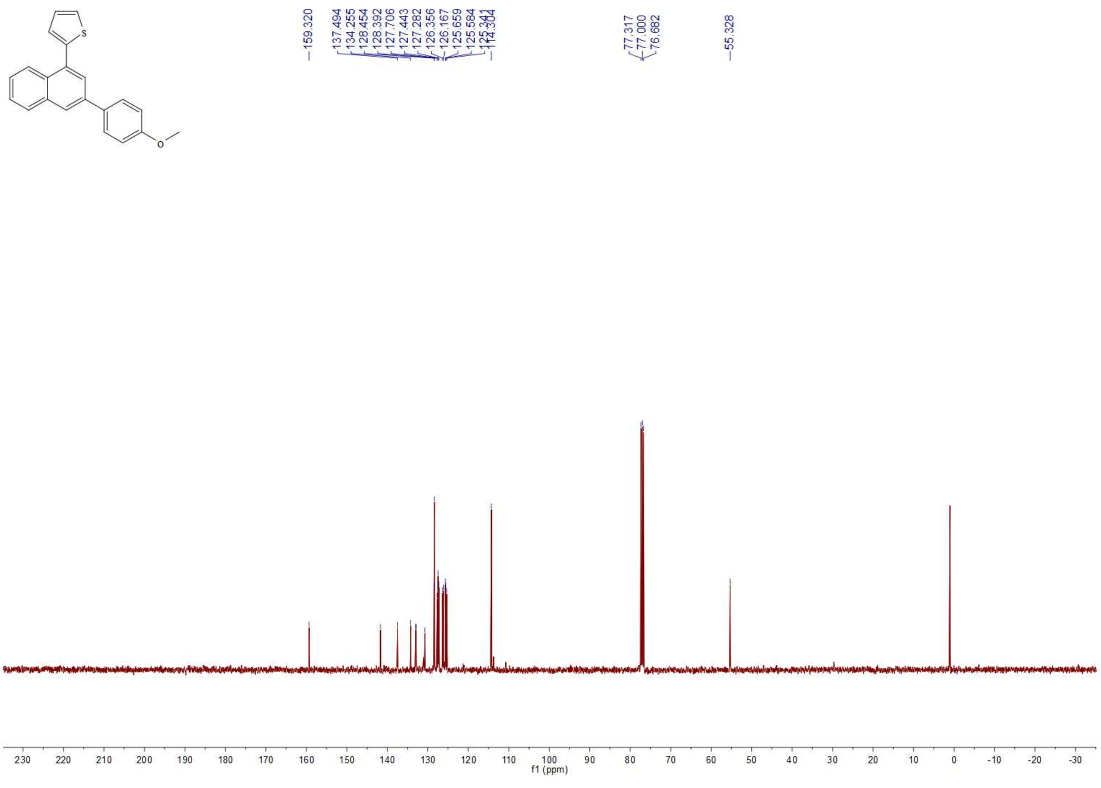

^{1}H NMR (400 MHz, $CDCl_3$) and ^{13}C NMR (100 MHz, $CDCl_3$) Spectrum of **3bf**.

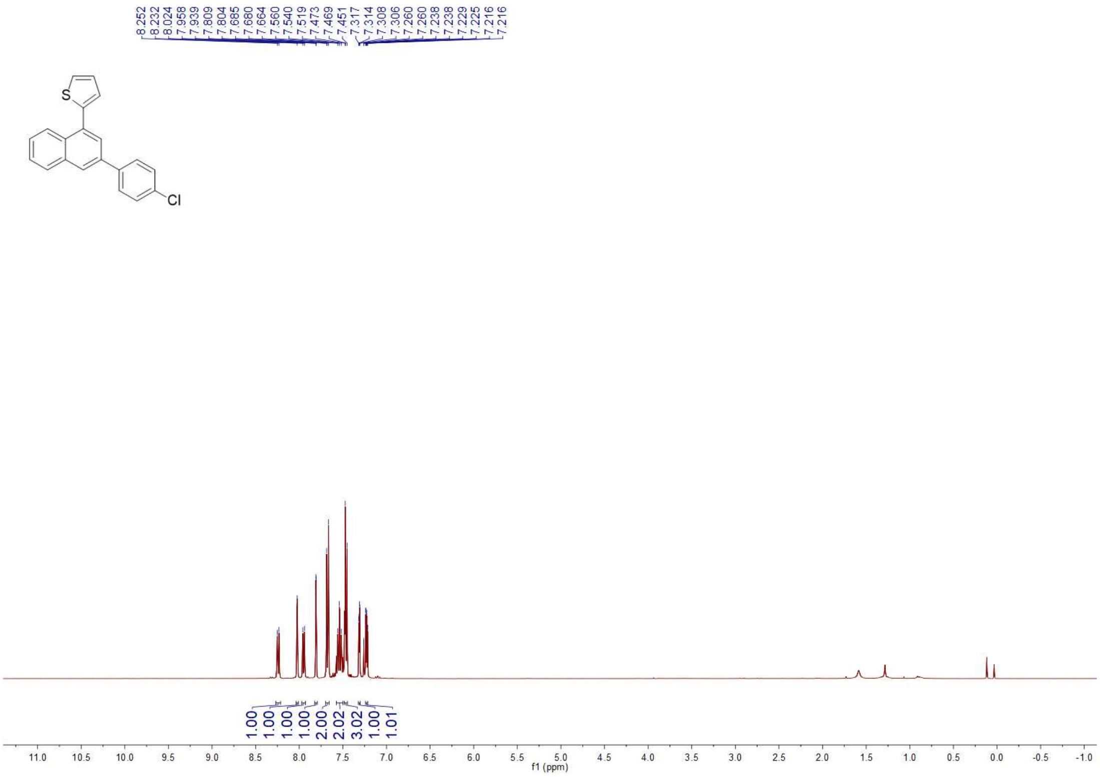

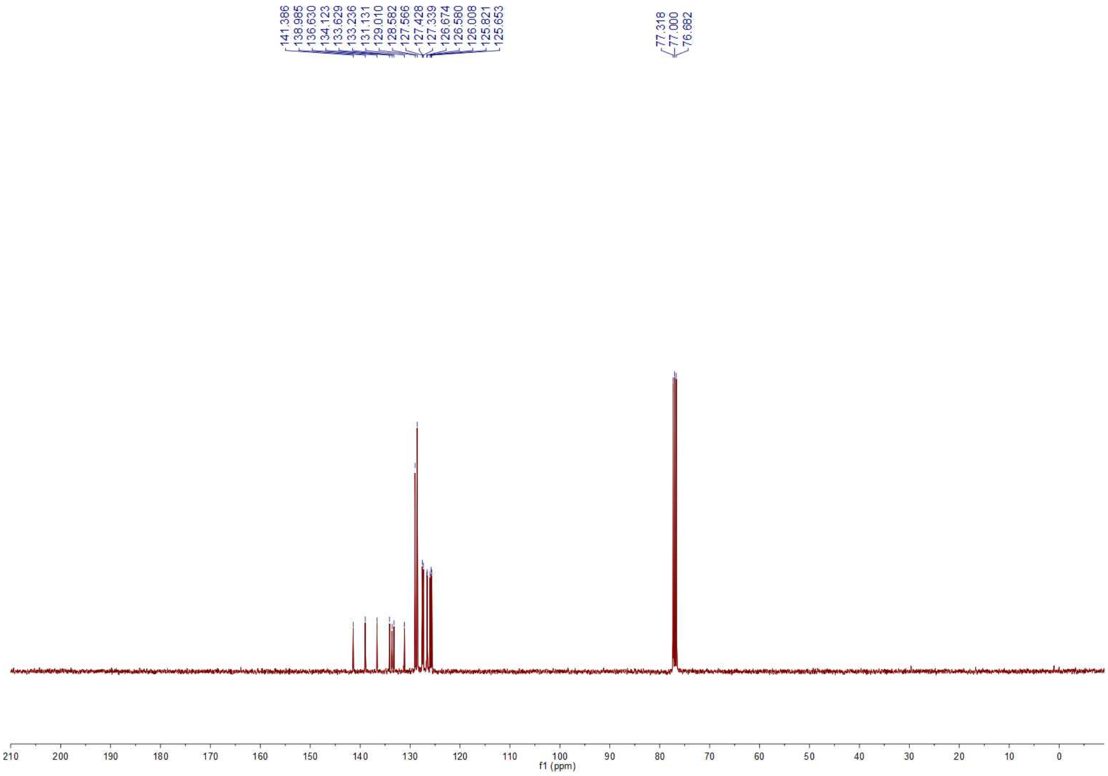

^{1}H NMR (400 MHz, $CDCl_3$) and ^{13}C NMR (100 MHz, $CDCl_3$) Spectrum of **3bg**.

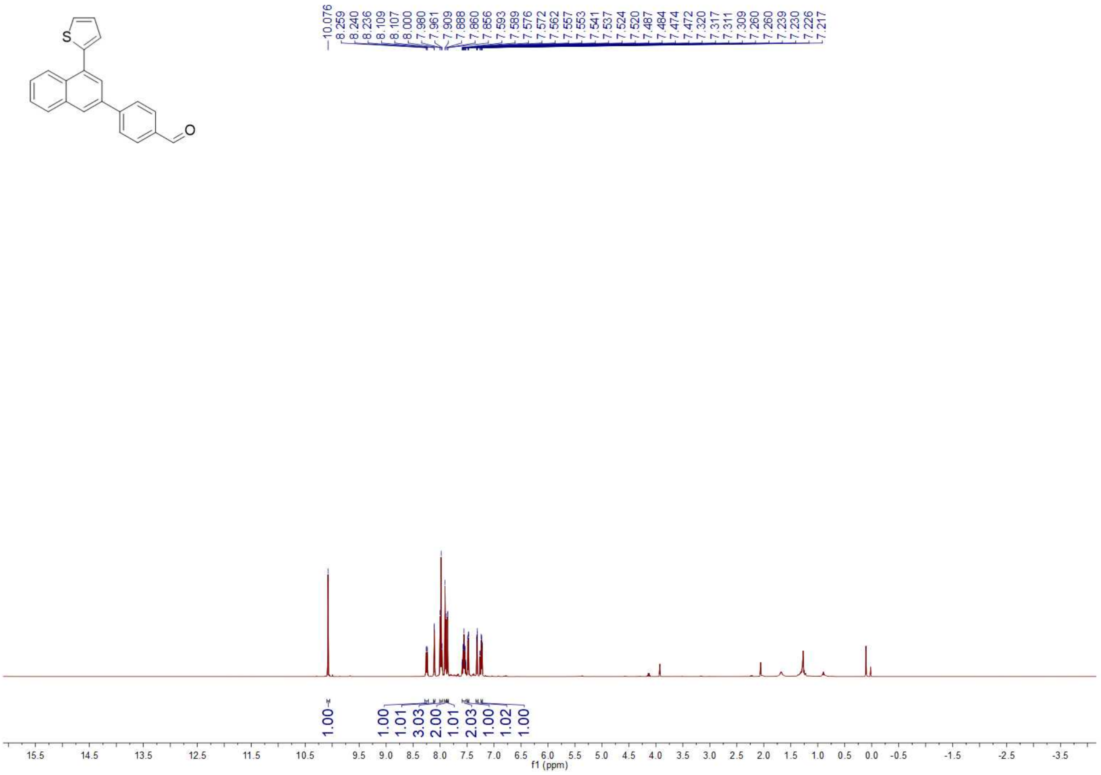

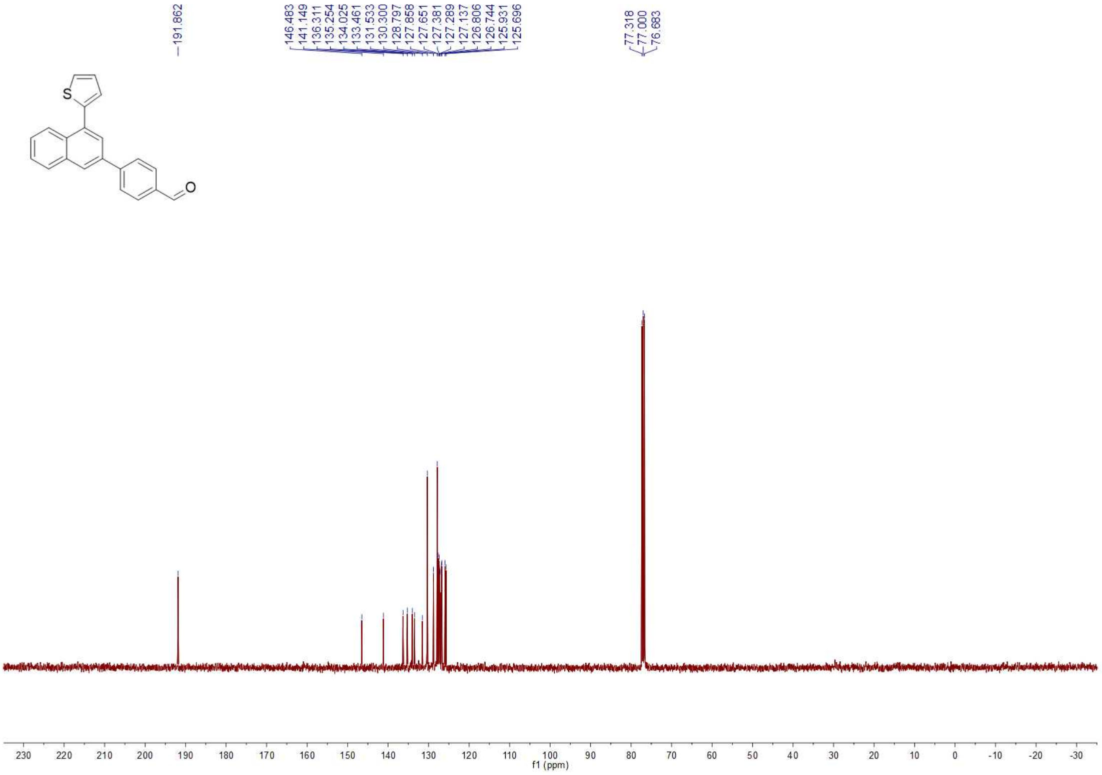

^{1}H NMR (400 MHz, $CDCl_3$) and ^{13}C NMR (100 MHz, $CDCl_3$) Spectrum of **3bh**.

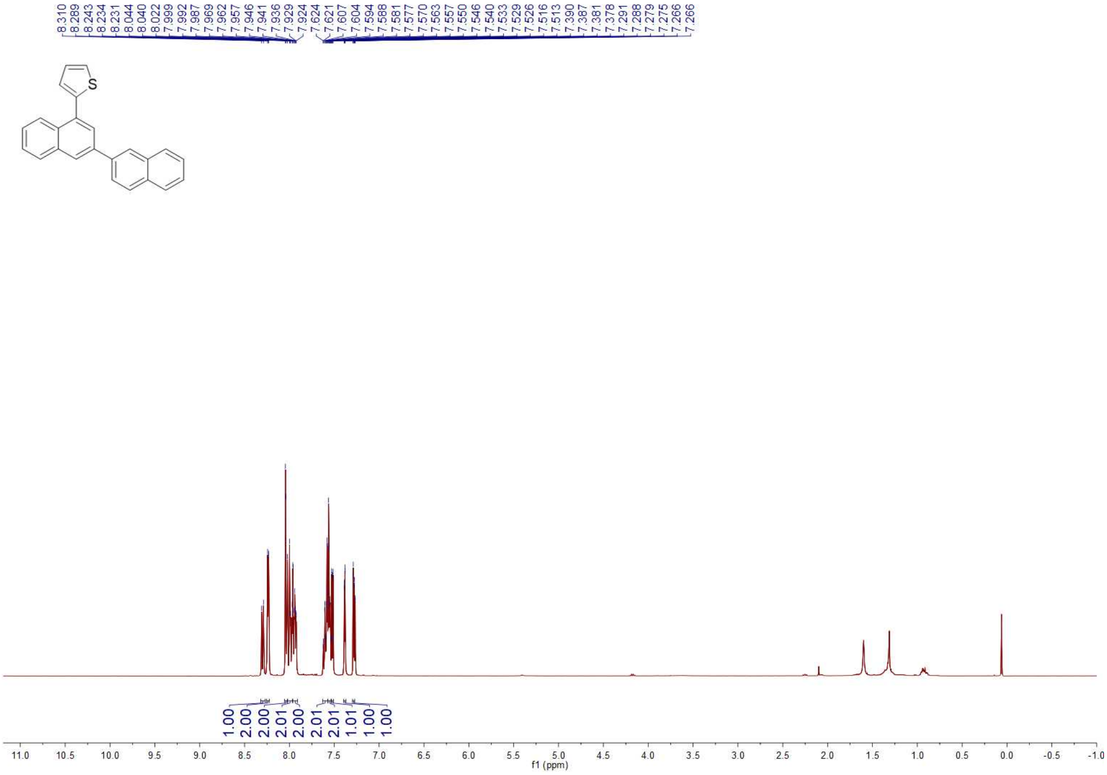

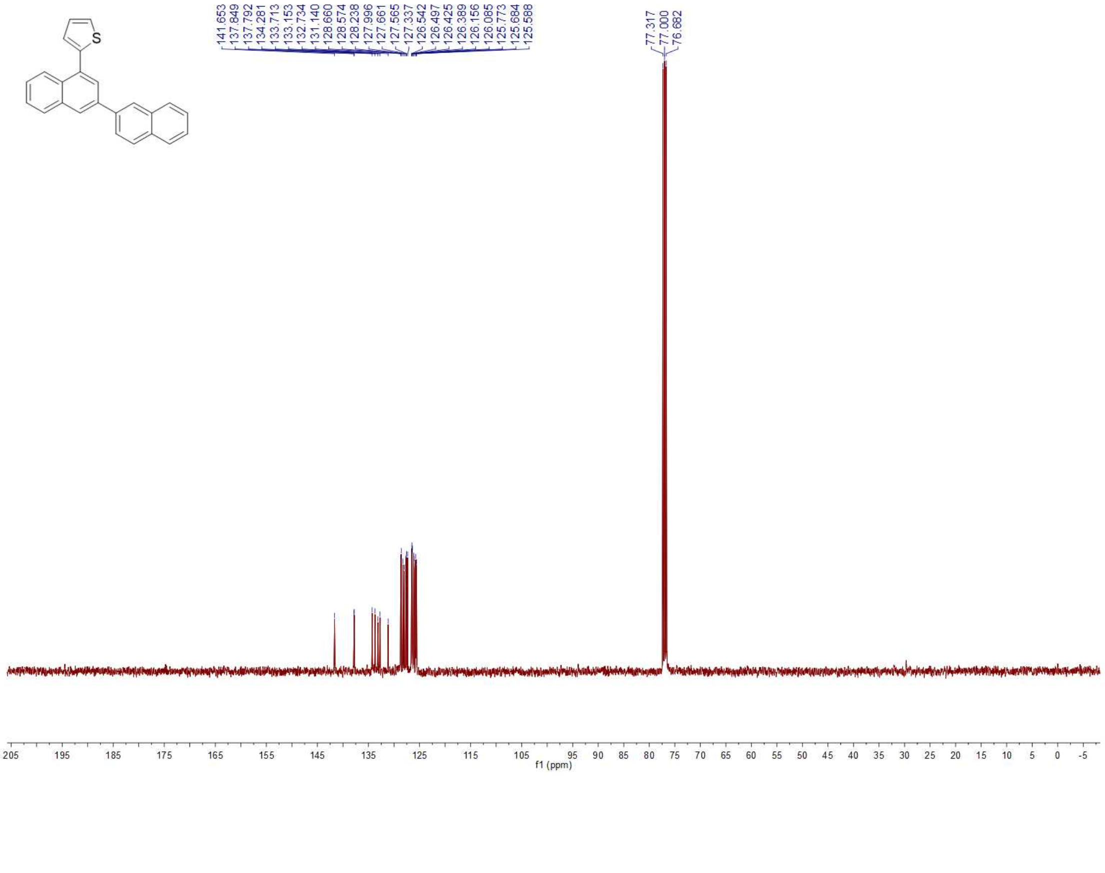

^{1}H NMR (400 MHz, $CDCl_3$) and ^{13}C NMR (100 MHz, $CDCl_3$) Spectrum of **3bi**.

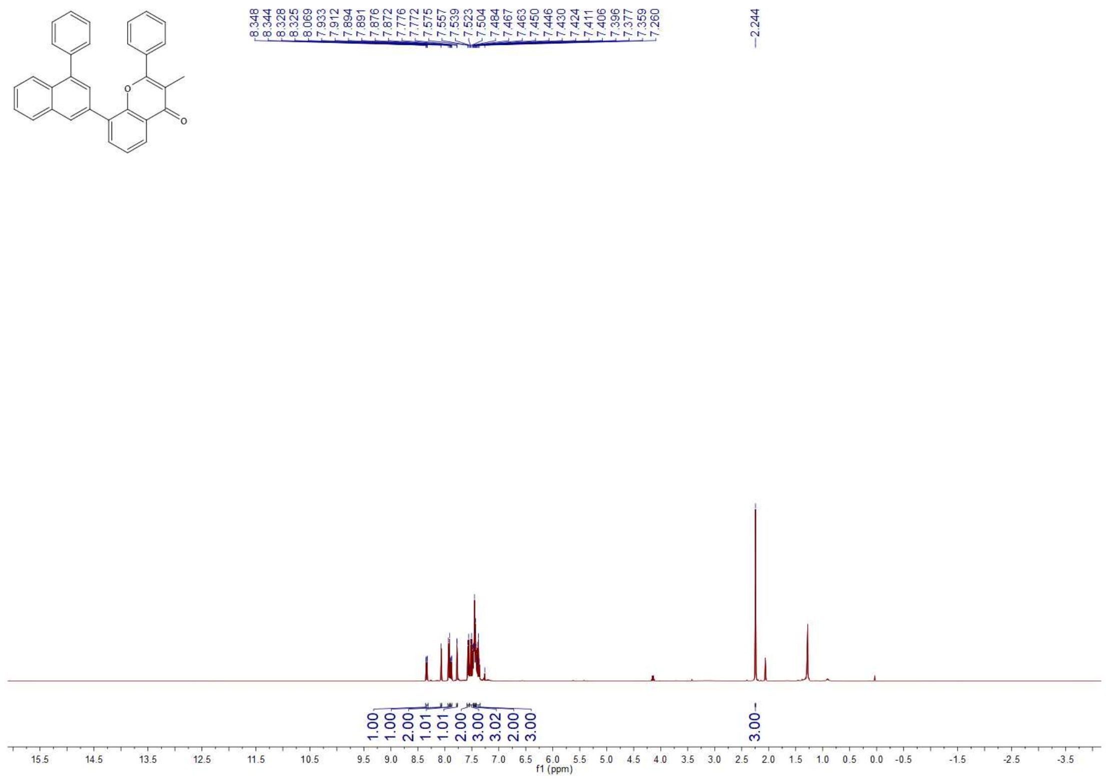

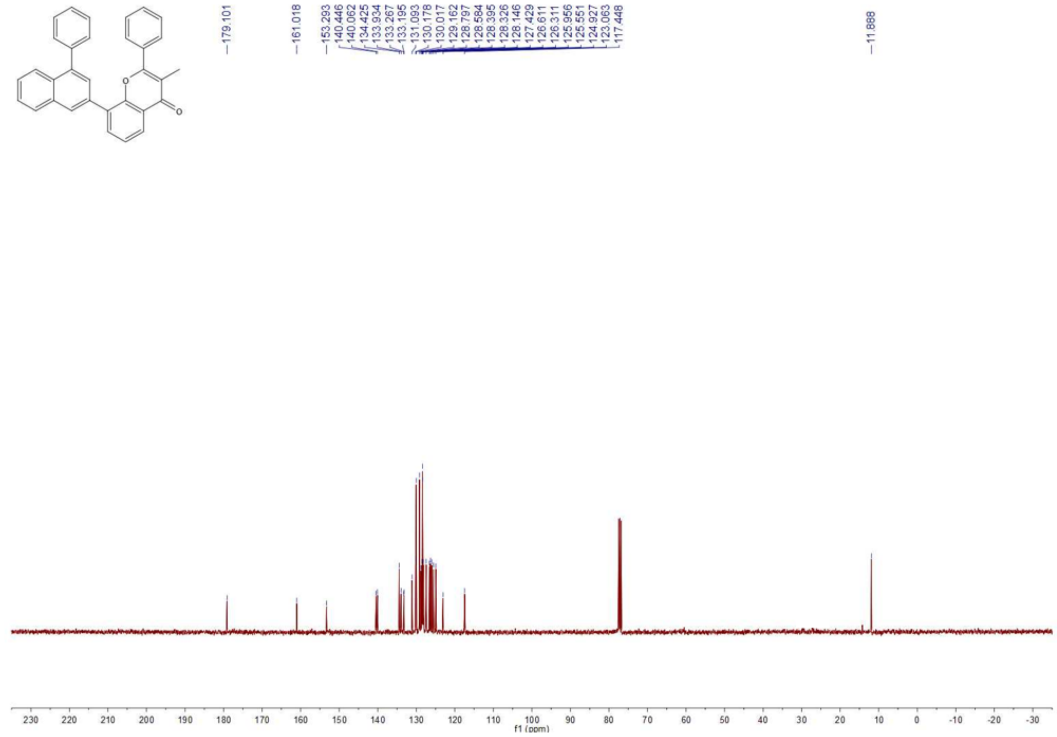

^{1}H NMR (400 MHz, $CDCl_3$) and ^{13}C NMR (100 MHz, $CDCl_3$) Spectrum of **3bj**.

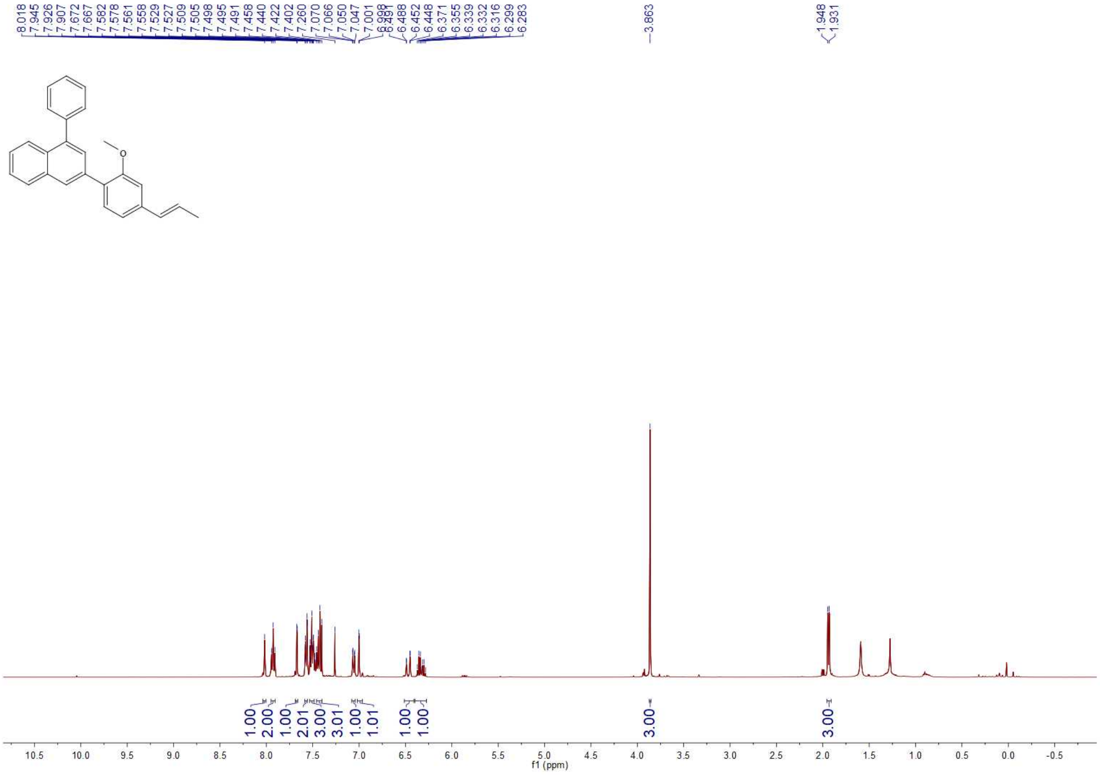

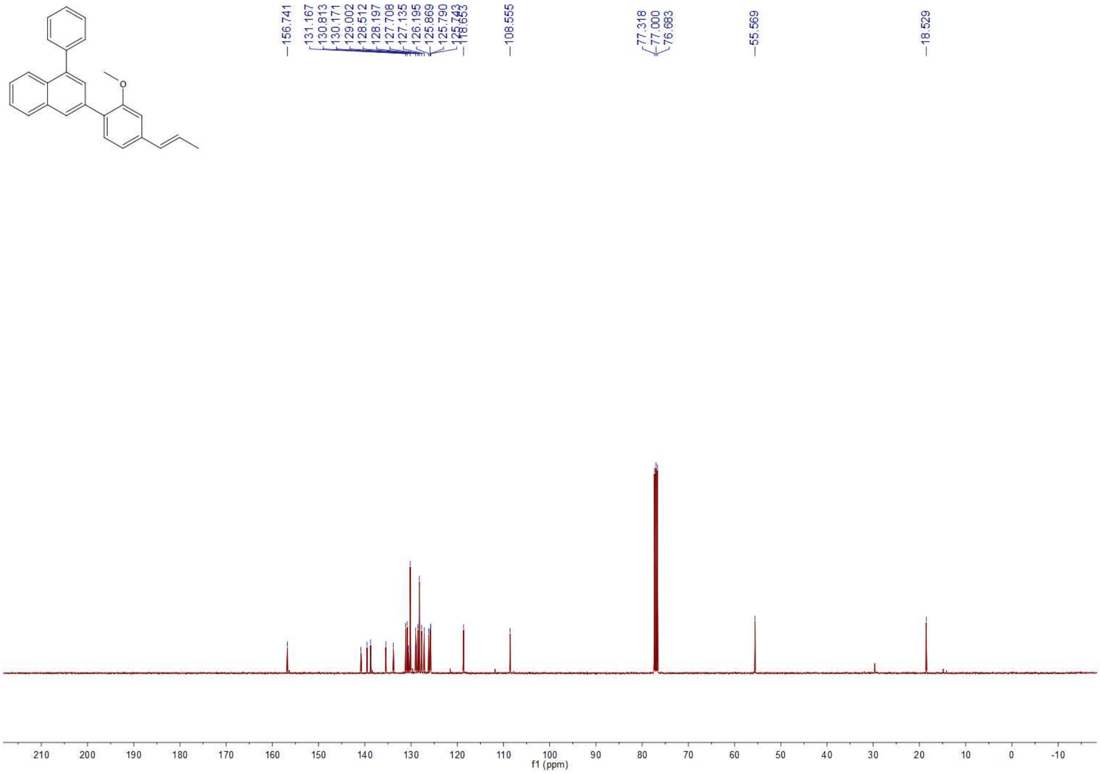

^{1}H NMR (400 MHz, $CDCl_3$) and ^{13}C NMR (100 MHz, $CDCl_3$) Spectrum of **3bk**.

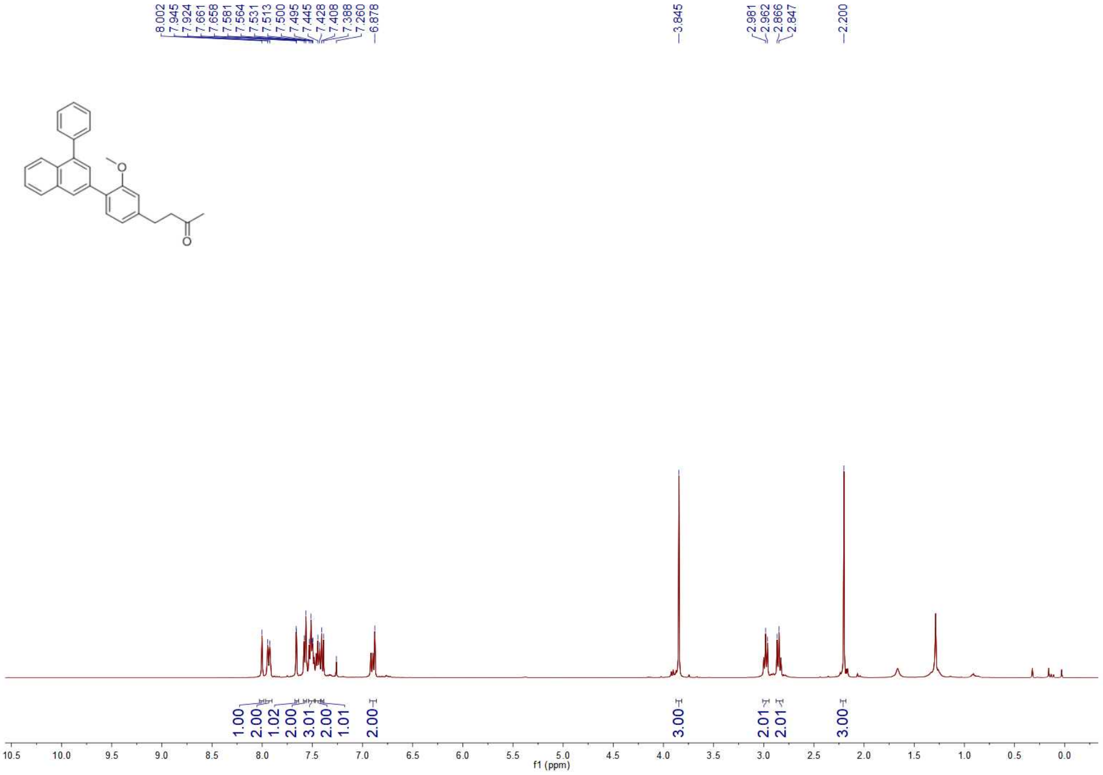

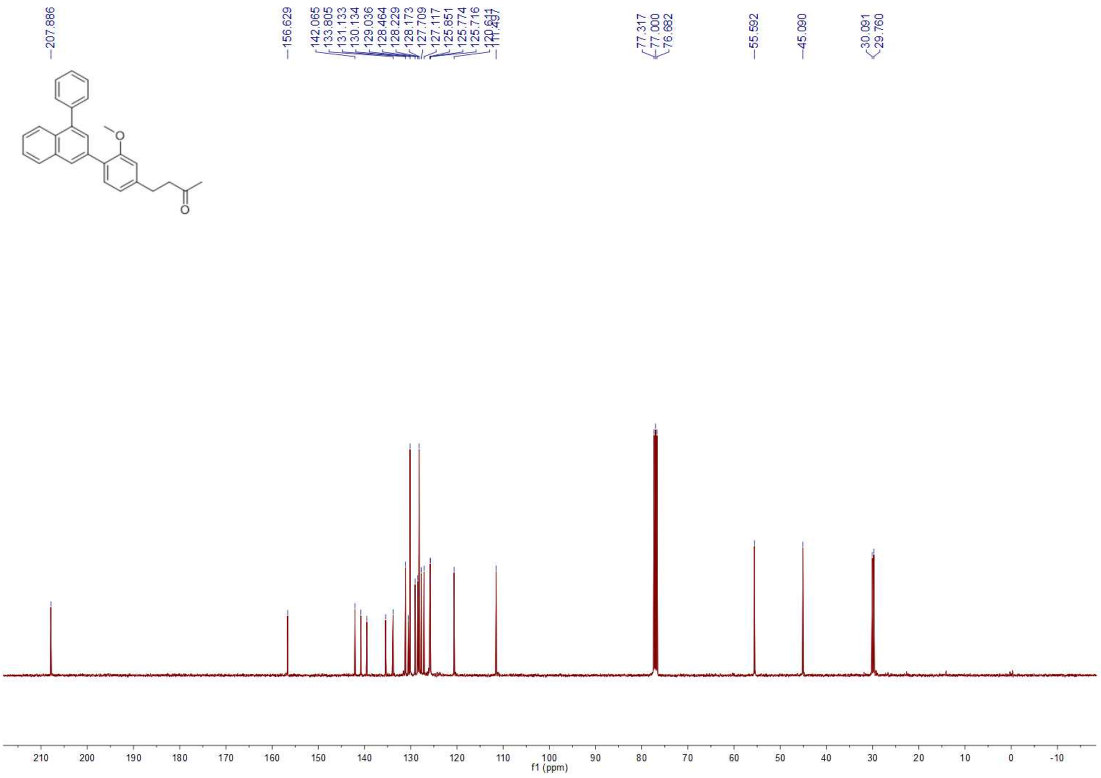

^{1}H NMR (400 MHz, $CDCl_3$) and ^{13}C NMR (100 MHz, $CDCl_3$) Spectrum of **3bl**.

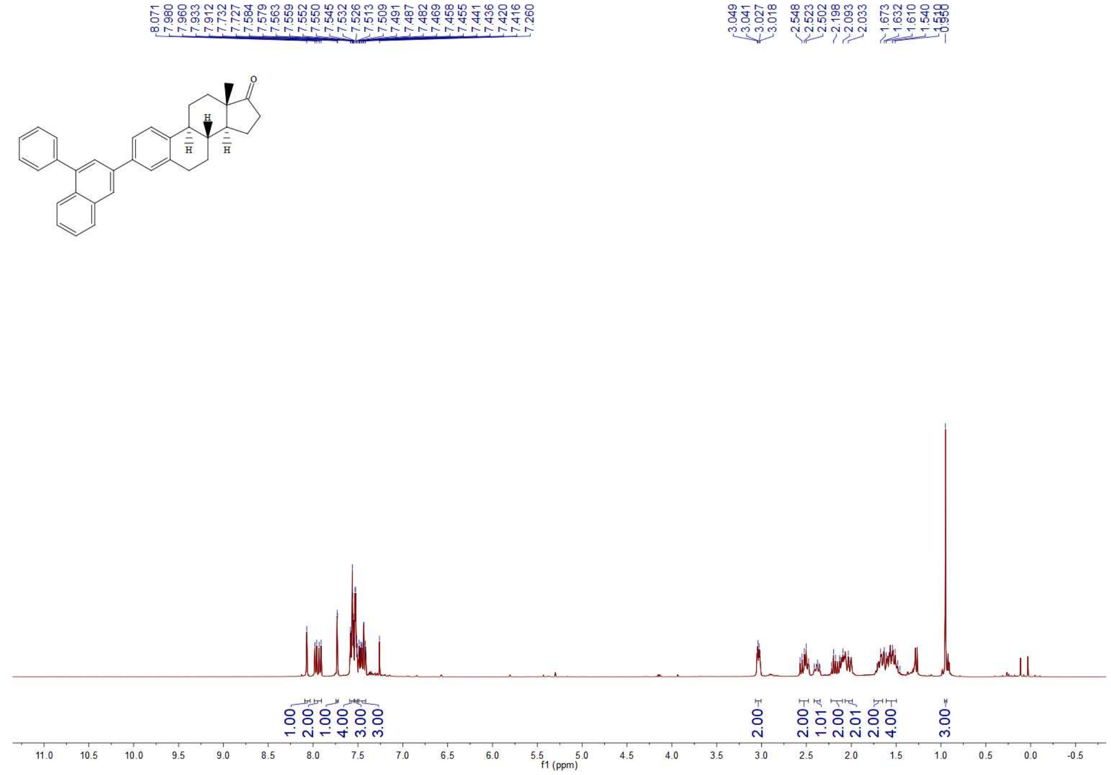

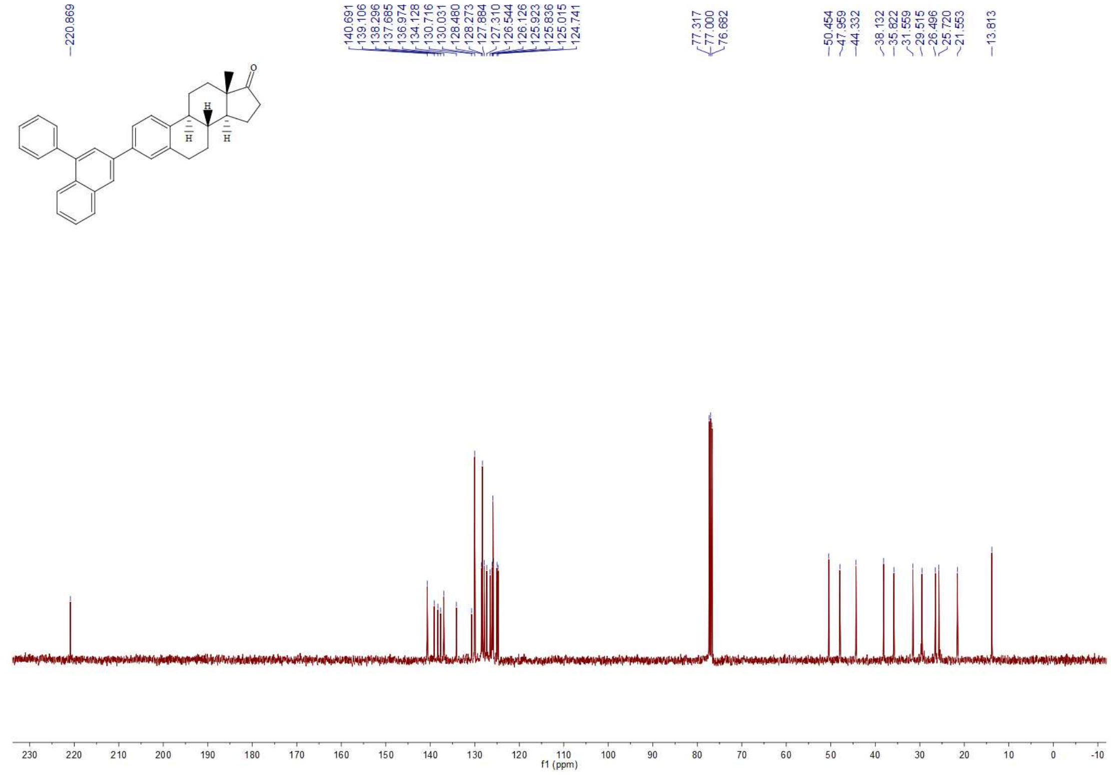

^{1}H NMR (400 MHz, $CDCl_3$) and ^{13}C NMR (100 MHz, $CDCl_3$) Spectrum of **3bm**.

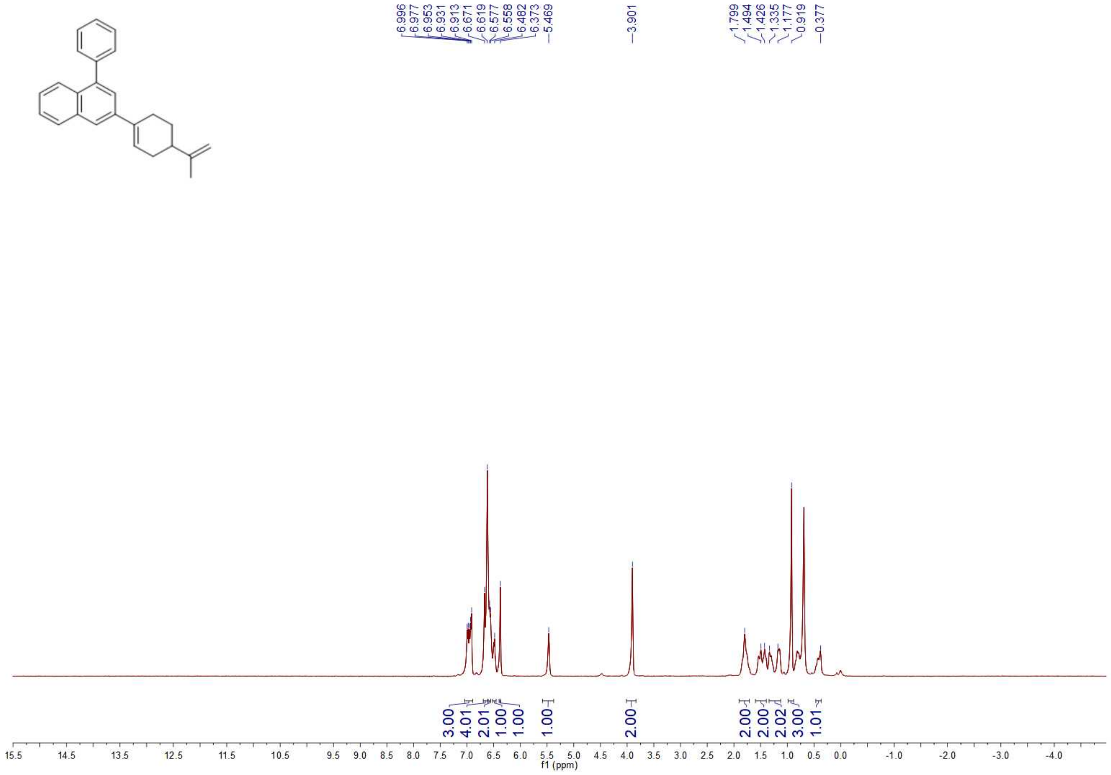

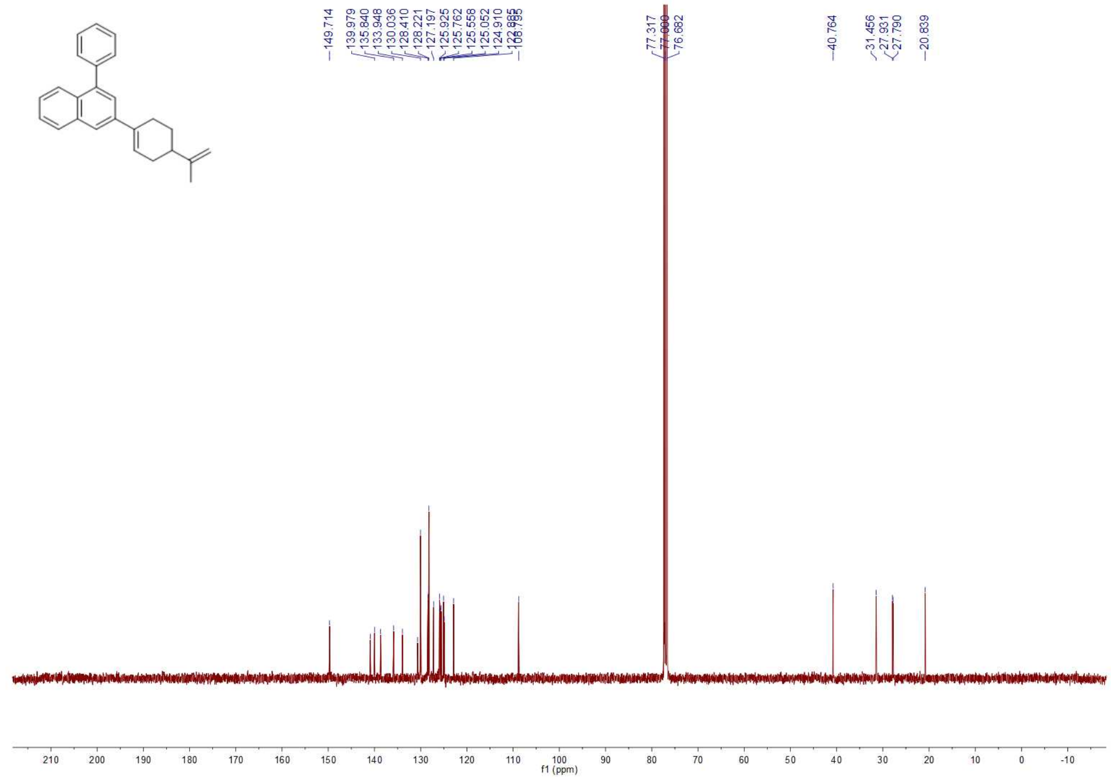

^{1}H NMR (400 MHz, $CDCl_3$) and ^{13}C NMR (100 MHz, $CDCl_3$) Spectrum of **6**.

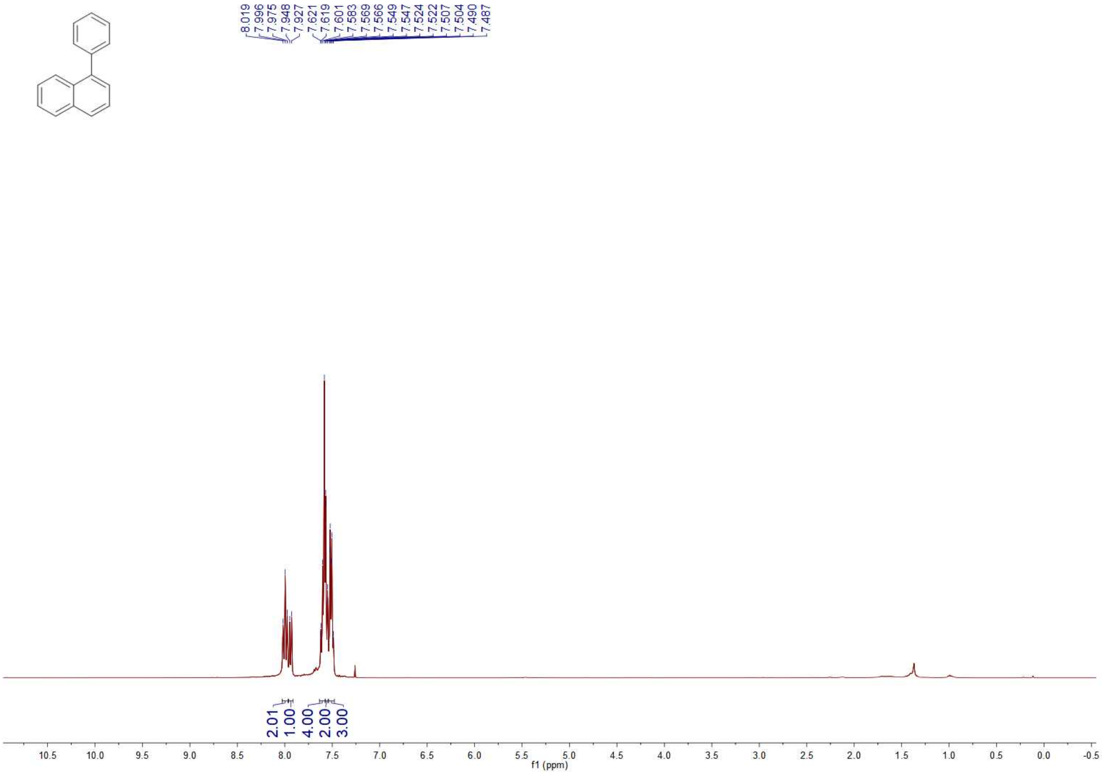

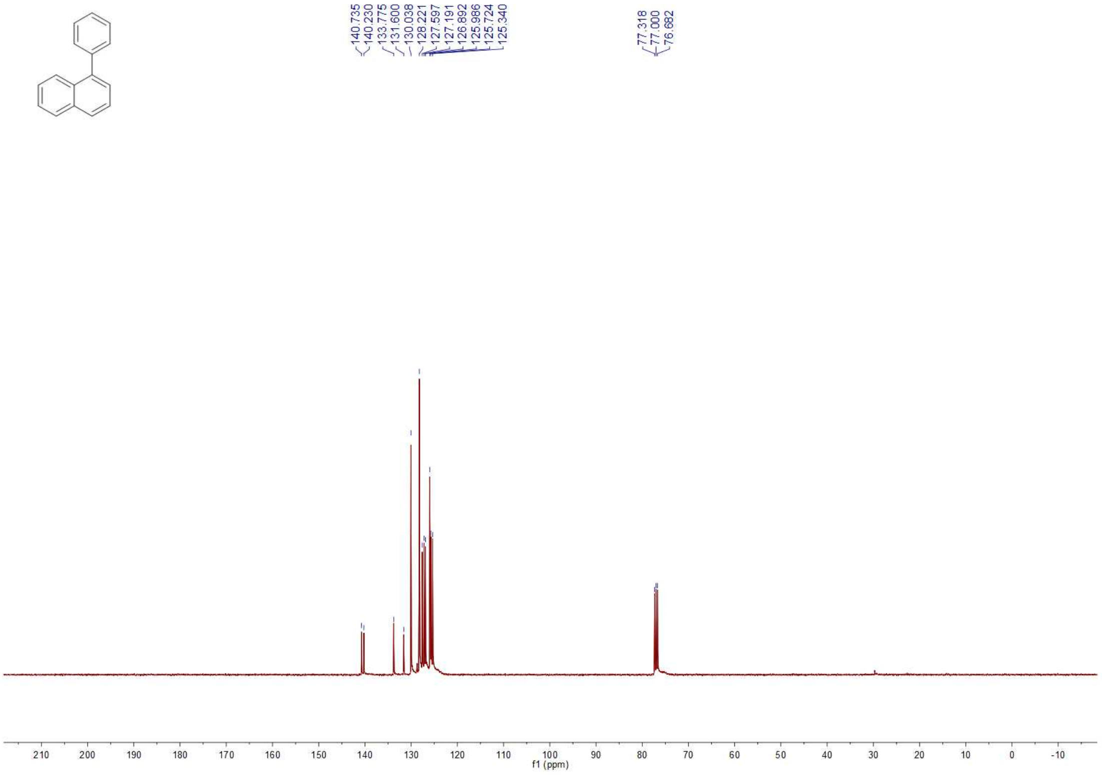

^{1}H NMR (400 MHz, $CDCl_3$) and ^{13}C NMR (100 MHz, $CDCl_3$) Spectrum of **7**.

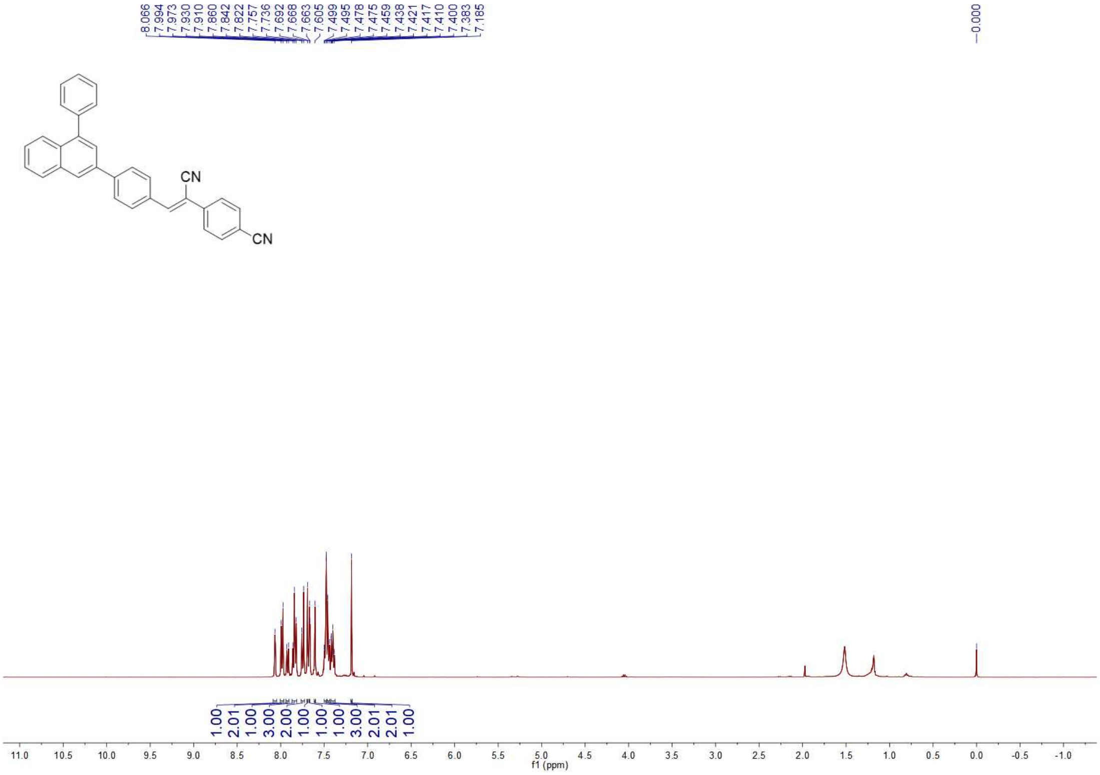

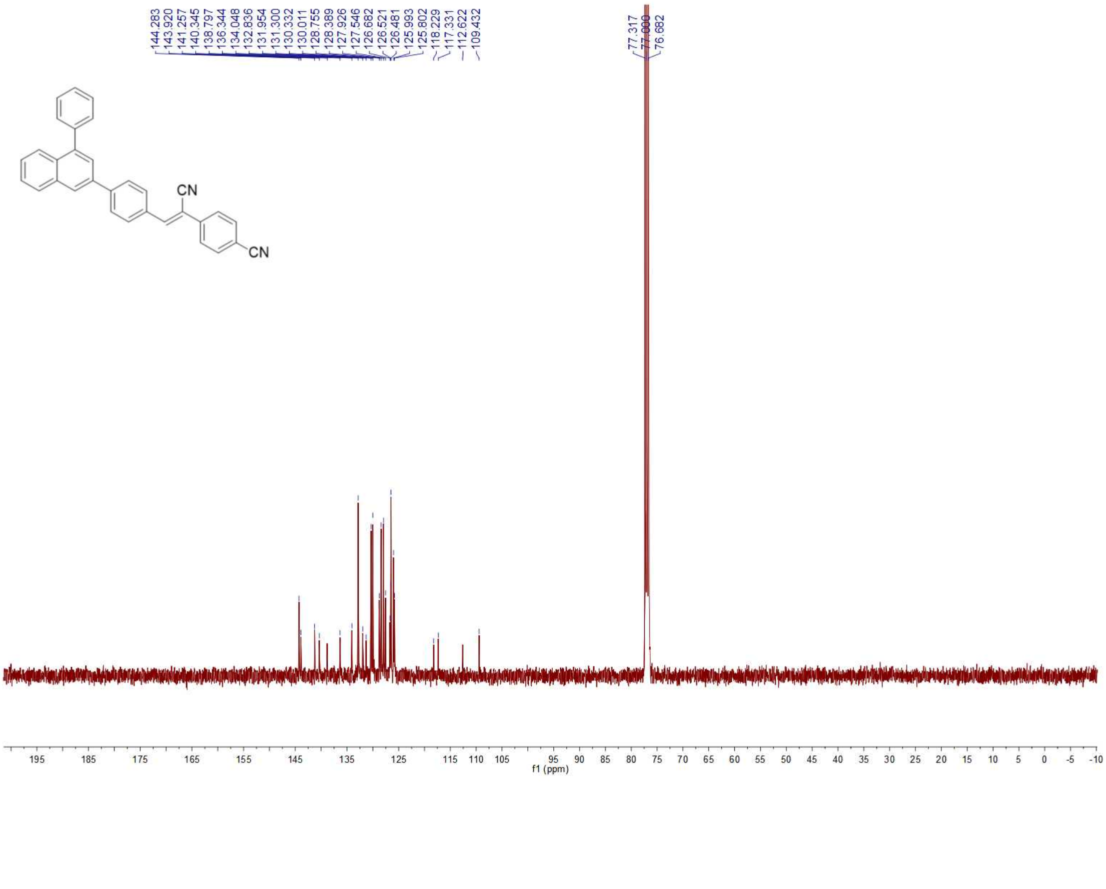

^{1}H NMR (400 MHz, $CDCl_3$) and ^{13}C NMR (100 MHz, $CDCl_3$) Spectrum of **8**.

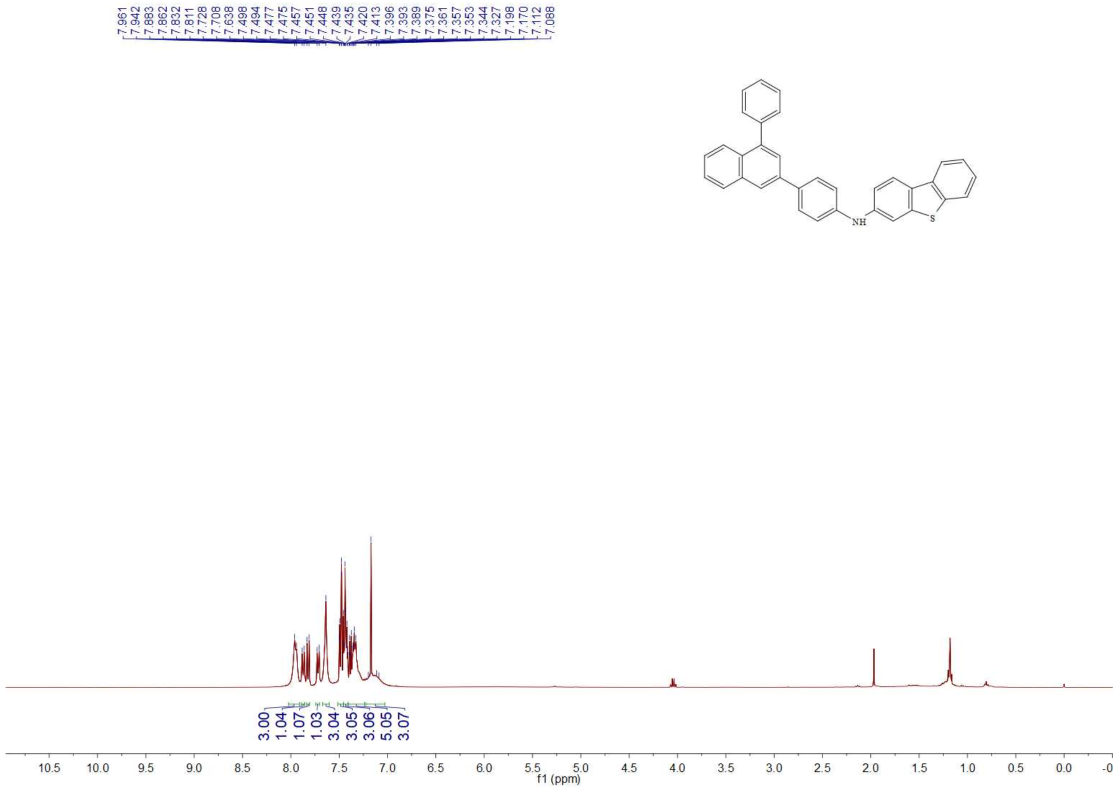

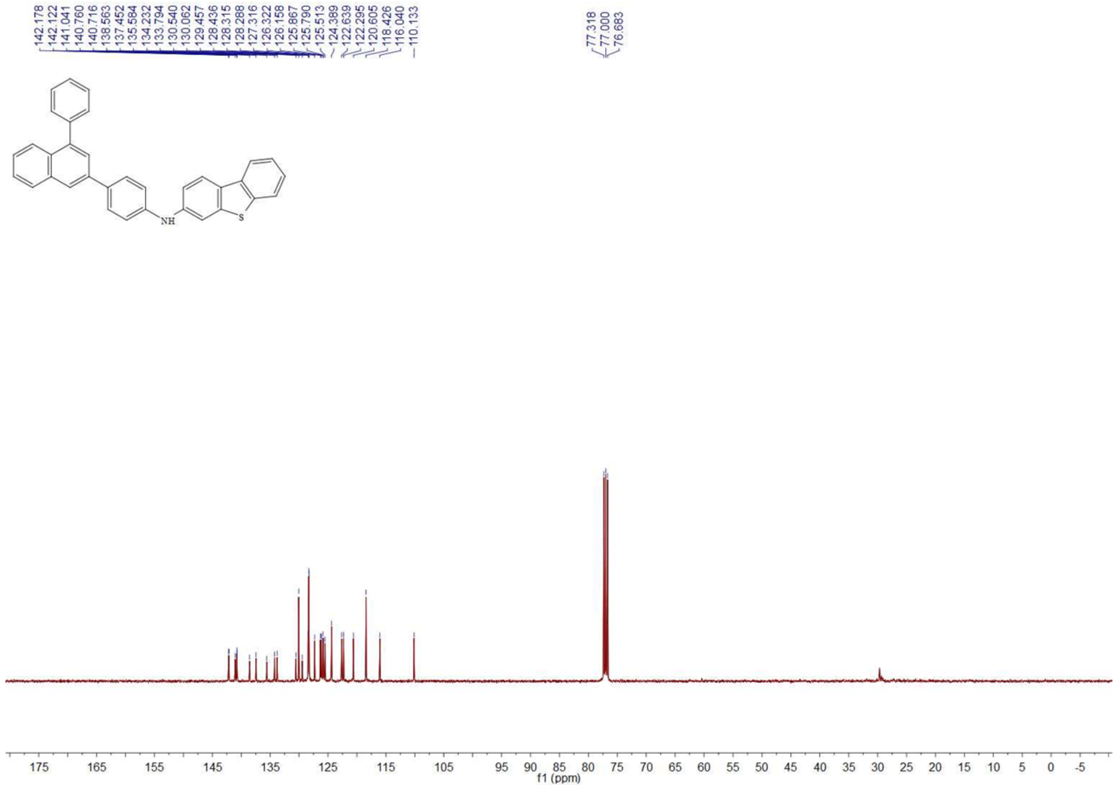

^{1}H NMR (400 MHz, $CDCl_3$) and ^{13}C NMR (100 MHz, $CDCl_3$) Spectrum of **1a-*d***.

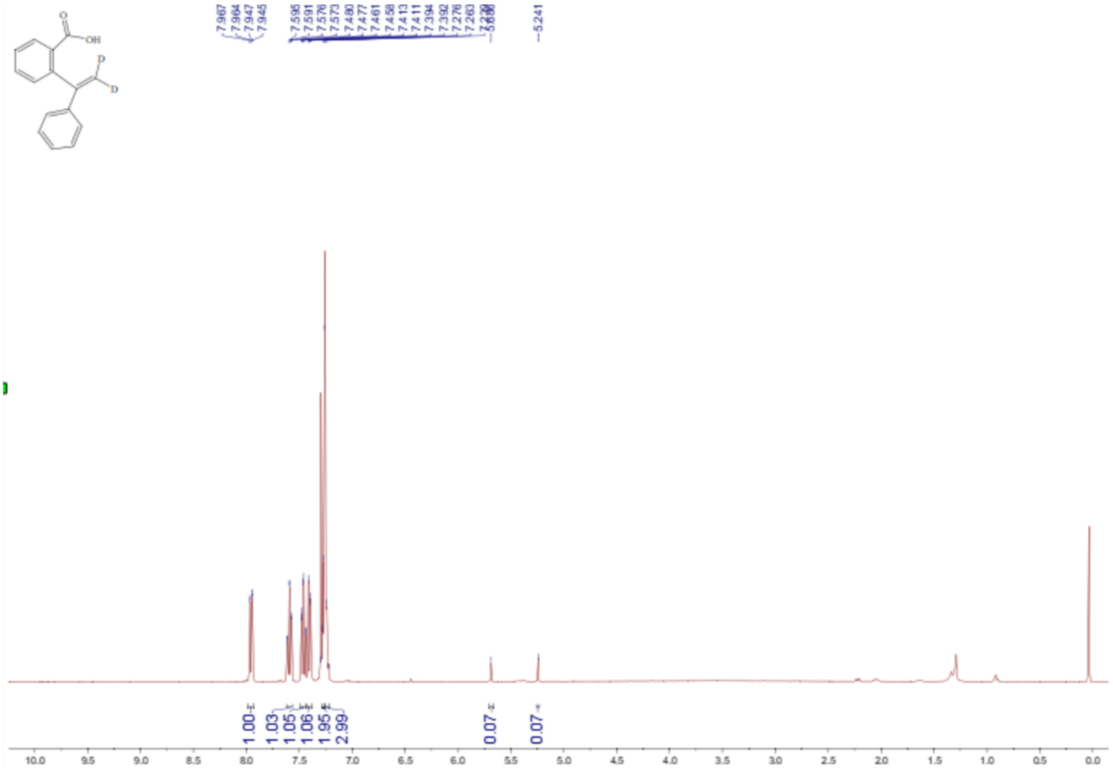

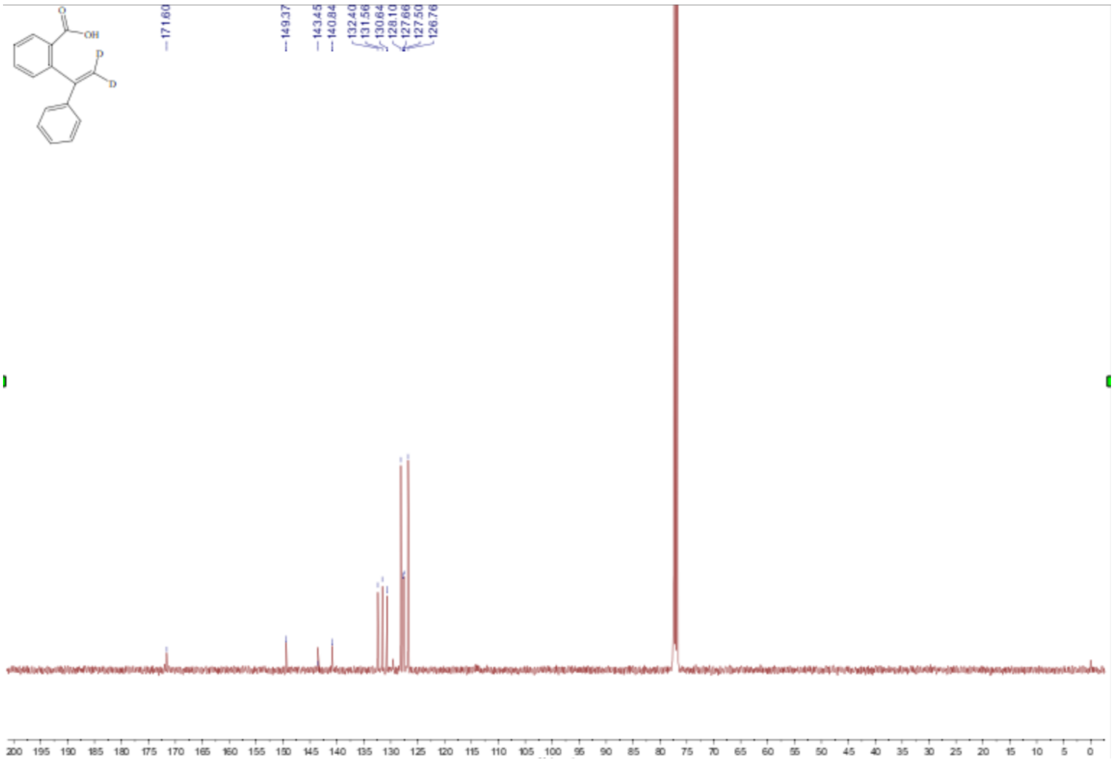

^{1}H NMR (400 MHz, $CDCl_3$) and ^{13}C NMR (100 MHz, $CDCl_3$) Spectrum of **3e-*d***.

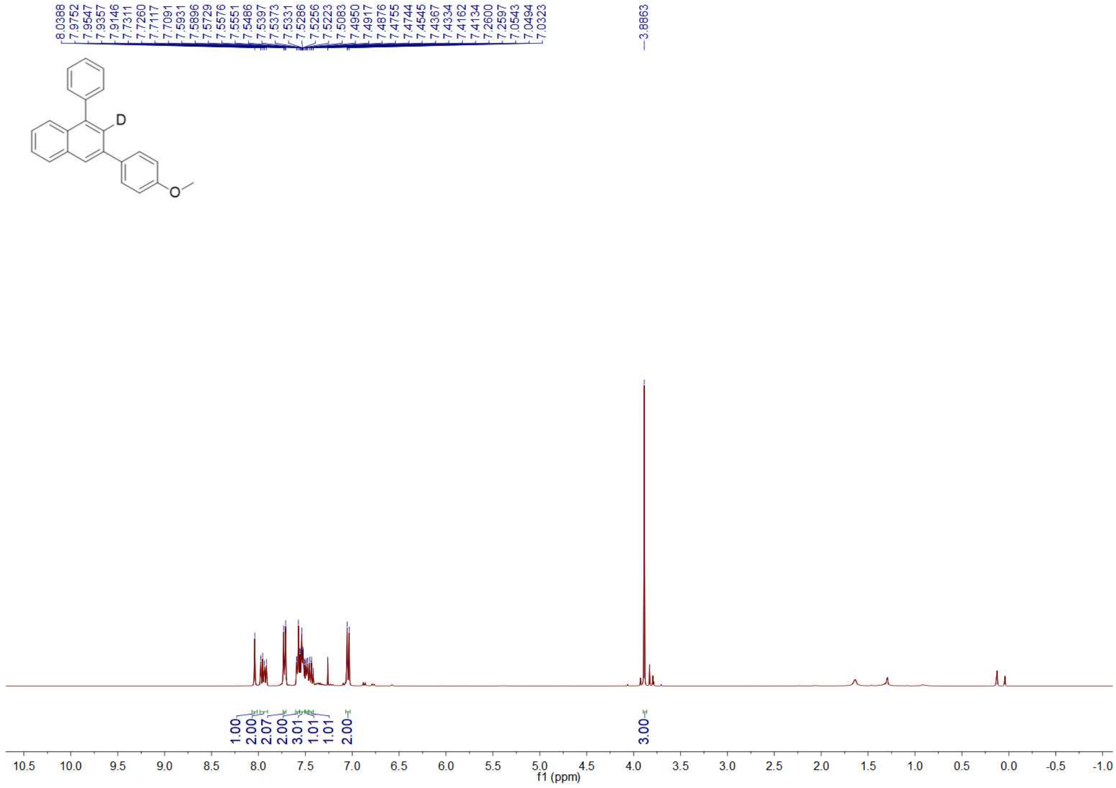

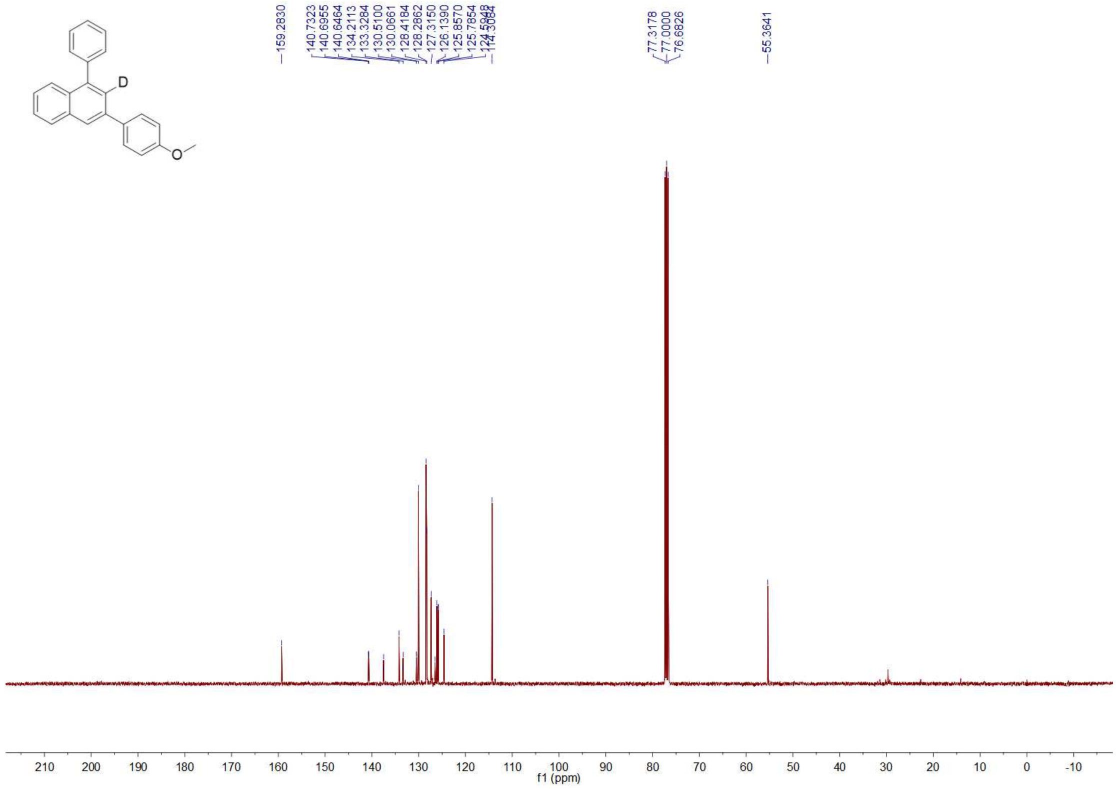

^{1}H NMR (400 MHz, $CDCl_3$) Spectrum of **3a-*d***.

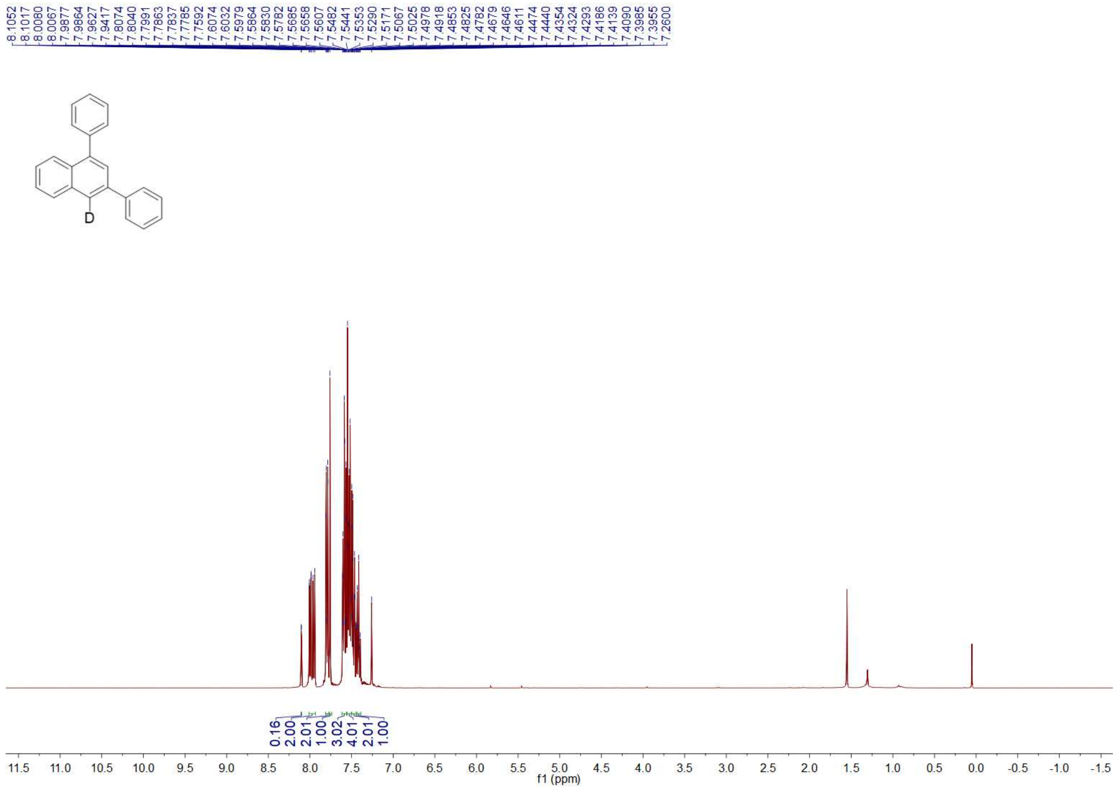

Supplement: SC-015-D4SC05429F-s001 [file SC-015-D4SC05429F-s001.pdf]
